# Topo II inhibition and DNA intercalation by new phthalazine-based derivatives as potent anticancer agents: design, synthesis, anti-proliferative, docking, and *in vivo* studies

**DOI:** 10.1080/14756366.2021.2007905

**Published:** 2021-12-11

**Authors:** Mohamed M. Khalifa, Ahmed A. Al-Karmalawy, Eslam B. Elkaeed, Mohamed S. Nafie, Mohamed A. Tantawy, Ibrahim H. Eissa, Hazem A. Mahdy

**Affiliations:** a Pharmaceutical Medicinal Chemistry & Drug Design Department, Faculty of Pharmacy (Boys), Al-Azhar University, Cairo, Egypt; b Department of Pharmaceutical Medicinal Chemistry, Faculty of Pharmacy, Horus University-Egypt, New Damietta, Egypt; c Department of Pharmaceutical Sciences, College of Pharmacy, AlMaarefa University, Riyadh, Saudi Arabia; d Chemistry Department, Faculty of Science, Suez Canal University, Ismailia, Egypt; e Hormones Department, Medical Research and Clinical Studies Institute, National Research Centre, Dokki, Egypt; f Stem Cells Lab, Center of Excellence for Advanced Sciences, National Research Centre, Dokki, Cairo, Egypt

**Keywords:** Topo II, DNA, antitumer, phthalazine, intercalators

## Abstract

This research presents the design and synthesis of a novel series of phthalazine derivatives as Topo II inhibitors, DNA intercalators, and cytotoxic agents. *In vitro* testing of the new compounds against HepG-2, MCF-7, and HCT-116 cell lines confirmed their potent cytotoxic activity with low IC_50_ values. Topo II inhibition and DNA intercalating activities were evaluated for the most cytotoxic members. IC_50_ values determination demonstrated Topo II inhibitory activities and DNA intercalating affinities of the tested compounds at a micromolar level. Amongst, compound **9d** was the most potent member. It inhibited Topo II enzyme at IC_50_ value of 7.02 ± 0.54 µM with DNA intercalating IC_50_ of 26.19 ± 1.14 µM. Compound **9d** was then subjected to an *in vivo* antitumor examination. It inhibited tumour proliferation reducing solid tumour volume and mass. Additionally, it restored liver enzymes, proteins, and CBC parameters near-normal, indicating a remarkable amelioration in their functions along with histopathological examinations.

## 1. Introduction

Cancer is characterised by uncontrolled cell growth and proliferation following genetic mutation. It represents one of the most important health issues worldwide and is the second leading cause of death^1^^,^^2^. Therefore, it represents one of the greatest challenges to medical researchers, especially with the continued failure of current therapies from one side and the development of drug resistance from the other side^3–5^.

The current search and discovery of new drug candidates with anticancer activities have become one of the most important issues for medicinal chemists nowadays^6–11^. Among the most important chemotherapeutic agents applied for cancer treatment are those that interact with DNA. Anticancer agents in the previously mentioned class belong to either alkylating agents, groove binders, or intercalating agents^12^. DNA intercalating agents got great attention from scientists due to their promising antitumoral activity^13–18^. They are classified into two major groups of compounds that intercalate between DNA base pairs (especially G and C, 70%) without covalent binding: **1)** acridines and related compounds and **2)** anthracyclines and related compounds^19^. These compounds produce local structural changes to the DNA molecule, including the lengthening of the DNA strand following the unwinding of its double helix. So, DNA intercalators are mutagenic due to their retardation or even inhibition of DNA transcription and replication^20^.

Doxorubicin is one of the two first isolated and introduced anthracyclines as antitumor agents. It works through two mechanisms of action; **1)** intercalates into the DNA double helix without covalent binding, and **2)** binds covalently to topoisomerase II (involved in DNA replication and transcription), poisons the cleavable complex of DNA and prevent its re-ligation, and finally results in an apoptotic action^21^^,^^22^.

**Figure 1.**
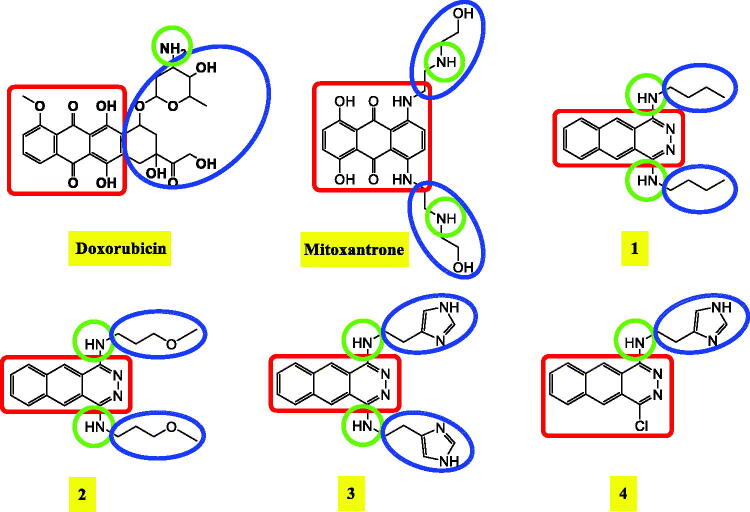
Some reported DNA intercalators and topoisomerase II inhibitors showing their common pharmacophoric features.

Phthalazine moiety was recommended in the area of medicinal chemistry to have promising antitumor activity and primarily to act as DNA intercalator and topoisomerase II inhibitors as well^16^^,^^23^^,^^24^. On the other hand, many other organic moieties like triazoles, hydrazine amides, hydrazine thioacetamides, benzylidene hydrazones, sulphonamides, benzoic acid, and thioacetamides derivatives were identified and introduced as potential antitumor agents^25–29^. Some reported DNA intercalators and topoisomerase II inhibitors showing their common pharmacophoric features were depicted in Figure 1.

### 1.1. The rationale of molecular design

A ligand-based drug design approach^30^^,^^31^ was performed to design a new wave of promising DNA intercalators and topoisomerase II inhibitors taking into consideration the basic pharmacophoric features of doxorubicin. It is worth mentioning that there are three crucial pharmacophoric features present in doxorubicin which guided our rationale. The first one is the planar polyaromatic system (chromophore) inserted in between the DNA base pairs. The second one is the presence of a groove binding side to occupy the minor groove of DNA. The third part is the cationic moiety, or a species having the ability to be protonated in the physiological PH to interact with the negatively charged phosphate group of DNA sugar moiety^32^^,^^33^.

**Figure 2.**
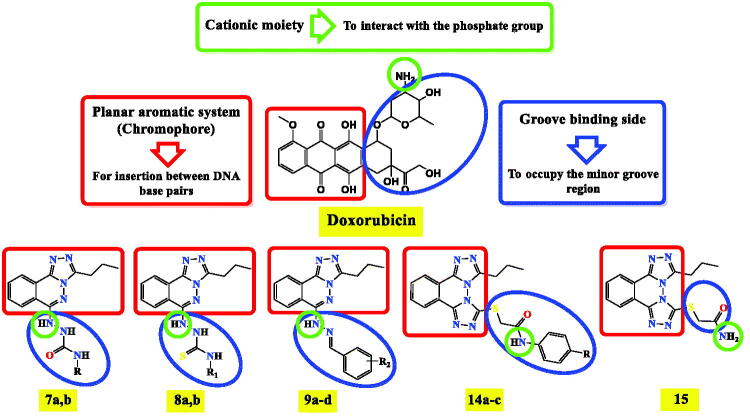
Molecular hybridisation of triazolo phthalazine moieties with different recommended anticancer moieties based on the basic pharmacophoric features of doxorubicin as DNA intercalator and topoisomerase II inhibitors.

Molecular hybridisation of triazolo phthalazine moieties instead of the planar aromatic system of doxorubicin with different recommended anticancer moieties (hydrazine amides, hydrazine thioacetamides, benzylidene hydrazones, sulphonamides, benzoic acid, and thioacetamides derivatives) as the groove binding site with the presence of -NH- or -NH_2_ group to act as a cationic site were designed and synthesised as depicted in Figure 2.

## 2. Results and discussion:

### 2.1. Chemistry

**Scheme 1.**
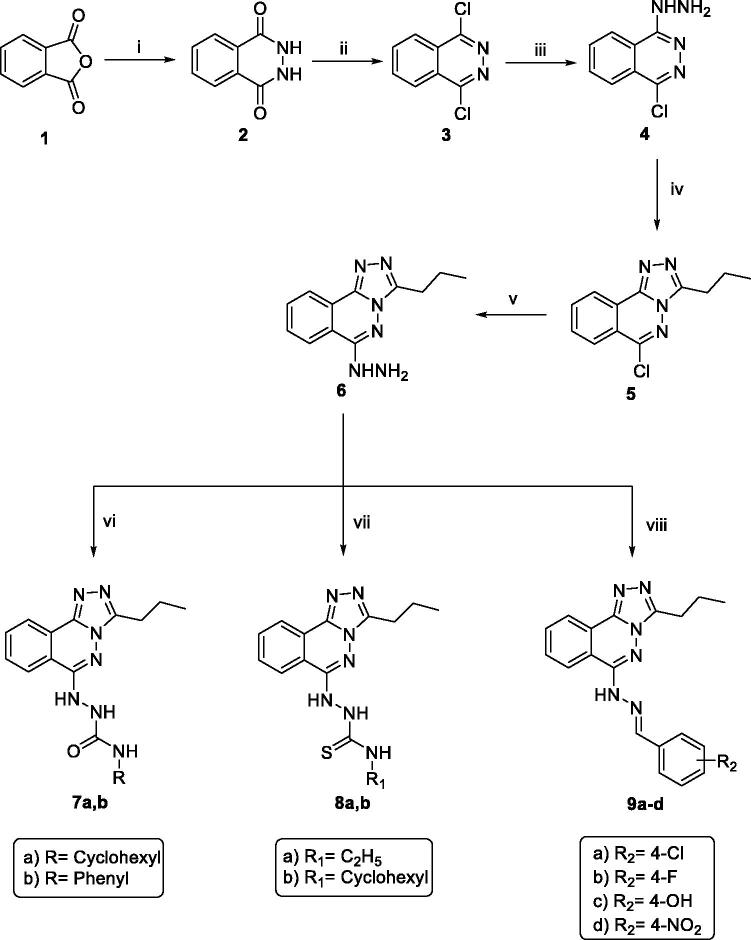
General procedure for synthesis of target compounds **7a,b**, **8a,b** and **9a-d**; Reagents and conditions: (**i**) NH_2_NH_2_.H_2_O / EtOH/reflux/5 h, (**ii**) POCl_3_/heating/1 h, (**iii**) NH_2_NH_2_.H_2_O / EtOH/reflux/0.5 h, (**iv**) Butyric anhydride/reflux/1 h, (**v**) NH_2_NH_2_.H_2_O / EtOH/reflux/0.5 h, (**vi**) The appropriate Isocyanates/EtOH/reflux/3 h, (**vii**) The appropriate Isothiocyanate/EtOH/reflux/3h, (**viii**) The appropriate Aromatic aldehydes / EtOH / gl. acetic acid / reflux/4h.

**Scheme 2.**
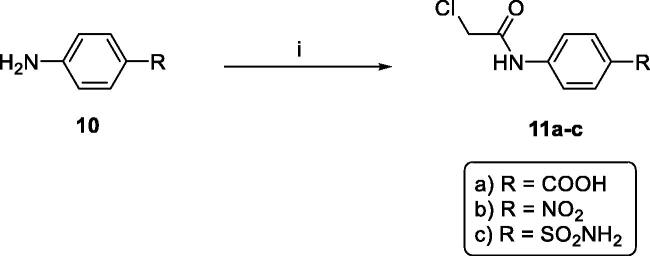
General procedure for synthesis of intermediates **11a-c,** Reagents and conditions: (i) chloroacetyl chloride/DMF/stirring/1.5h.

The new triazolo phthalazine members were synthesised following the reactions outlined in Schemes 1 and 2. 2,3-Dihydrophthalazine-1,4-dione **2** was prepared by reaction of phthalic anhydride **1** with hydrazine hydrate in absolute ethanol^34^. Compound **2** was then chlorinated with phosphorus oxychloride to afford 1,4-dichlorophthalazine **3**^35^, which was then heated with hydrazine hydrate in boiling ethanol^36^ to furnish 1-chloro-4-hydrazinylphthalazine **4**. A solvent-free reaction was performed to cyclize compound **4**; thus, compound **4** was heated with butyric anhydride to give the cyclized member **5**^37^. Reflux of compound **5** with hydrazine hydrate in boiling ethanol afforded the target hydrazinyl triazolo derivative **6**. Compound **6**, however, was allowed to react with different isocyanates and/or isothiocyanates to afford the corresponding semicarbazides **7a,b,** and/or thiosemicarbazides **8a,b,** respectively. Furthermore, treating the hydrazinyl compound **6** with appropriate substituted benzaldehyde derivatives with a catalytic amount of glacial acetic acid afforded the corresponding imines (Schiff's bases) **9a-d**. IR charts of the later compounds revealed the loss of NH_2_ absorption band of compound **6** and the presence of NH absorption bands in the range of 3180 to 3242 cm^−1^. In contrast, ^1^H NMR spectra of members **9a-d** displayed characteristic singlet signals in the range of *δ* 8.12 − 8.72 ppm representing the new benzylidene protons. The ^13 ^C NMR spectra of compounds 9a-d, however, showed a characteristic downfield peak around *δ* 141 ppm corresponding to the new benzyledine carbon (Scheme 1).

**Scheme 3.**
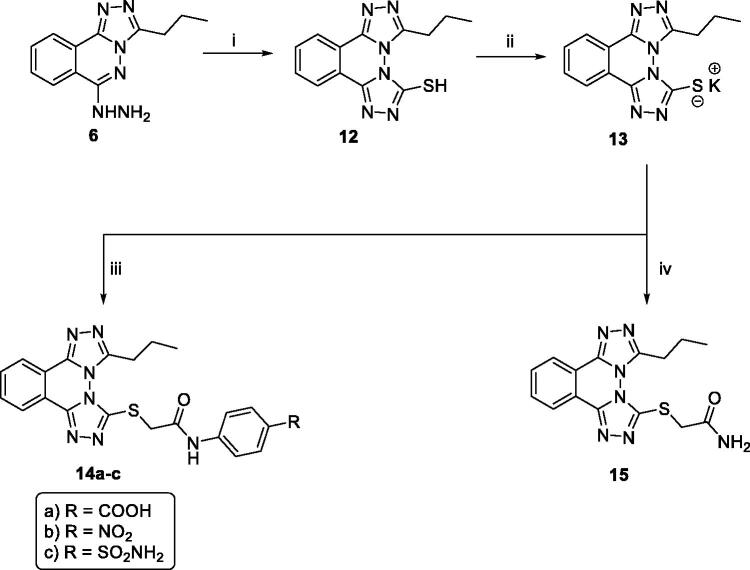
General procedure for synthesis of target compounds **14a-d** and **15**; Reagents and conditions: (**i**) 1) CS_2/_KOH/EtOH/reflux/3 h, 2) HCl, (**ii**) KOH/absolute EtOH/reflux/0.5 h, (**iii**) *N*-Aryl-2-chloroacetamide derivatives **11a-c**/DMF/heating/KI/heating/3 h. (**iv**) Chloroacetamide/DMF/heating over water bath/KI/heating/3 h.

Upon cyclisation of hydrazinyl triazolo derivative **6** with carbon disulphide in alcoholic potassium hydroxide, the corresponding mercaptotriazole derivative **12** was afforded.^16^ ^1^H NMR spectrum of **12** displayed a singlet D_2_O exchangeable signal at *δ* 14.24 ppm corresponding to the SH proton. The potassium salt **13** was then obtained upon heating compound **12** with potassium hydroxide in absolute ethanol^29^. The potassium salt **13** was heated with the appropriate *N*-aryl-2-chloroacetamide derivatives **11a-c** and/or 2-chloroacetamide in the presence of a catalytic amount of potassium iodide in DMF following the reported procedure to afford the corresponding thioacetamide derivatives, **14a-c** and **15,** respectively (Scheme 3).

### 2.2. Biological evaluation

#### 2.2.1. *In vitro* anti-proliferative activities

Anti-proliferative activities of the target compounds were assessed via the standard MTT method^38–40^ against three cancer cell lines, namely, hepatocellular carcinoma (HepG-2), colorectal carcinoma (HCT-116), and human breast adenocarcinoma (MCF-7). Doxorubicin was used in this test as a positive control.

As illustrated in Table 1, the obtained results revealed that most synthesised compounds showed remarkable anti-proliferative activities against the tested cell lines.

**Table 1.** Anti-proliferative activities towards HepG2, HCT-116, and MCF-7 cell lines.

Comp. No.	*In vitro* Cytotoxicity IC_50_ (µM)^a^		
	HepG-2	HCT-116	MCF-7
**7a**	24.92 ± 0.8	23.8 ± 0.81	19.45 ± 0.62
**7b**	44.96 ± 1.1	48.58 ± 1.60	50.69 ± 1.55
**8a**	28.86 ± 0.67	25.04 ± 0.70	16.48 ± 0.43
**8b**	10.92 ± 0.34	13.79 ± 0.44	12.54 ± 0.32
**9a**	25.16 ± 0.70	36.29 ± 1.10	45.52 ± 1.22
**9b**	63.86 ± 1.02	61.71 ± 1.89	35.99 ± 0.98
**9c**	56.32 ± 1.73	78.49 ± 2.04	57.76 ± 1.56
**9d**	5.08 ± 0.17	4.74 ± 0.15	4.95 ± 0.10
**14a**	5.65 ± 0.25	4.35 ± 0.19	4.36 ± 0.20
**14b**	13.40 ± 0.40	12.64 ± 0.45	14.94 ± 0.55
**14c**	24.75 ± 0.88	30.99 ± 0.11	20.64 ± 0.88
**15**	38.63 ± 1.00	53.54 ± 1.55	26.18 ± 0.77
**Doxorubicin**	8.28 ± 0.32	9.62 ± 0.50	7.67 ± 0.37

^a^IC_50_ values are the mean ± SD of three separate experiments.

In general, compounds **9d** and **14a** were found to be more active than the reference drug, doxorubicin, against the three tested cell lines. In particular, compound **9d** was the most potent counterpart with IC_50_ values of 5.08, 4.74, and 4.95 µM as it was 1.63, 2.03, and 1.34 times more active than doxorubicin (IC_50_ = 8.28, 9.62, and 7.67 µM) against HepG2, HCT‐116, and MCF‐7 cell lines, respectively. While, compound **14a** was about 1.46, 2.28, and 1.75 times as active as doxorubicin with IC_50_ values of 5.65, 4.35, and 4.36 µM. Moreover, compounds **8b** and **14b** were found to have satisfactory cytotoxicity against HepG2, HCT-116, and MCF-7 cell lines with IC_50_ values ranging from 10.92 to 14.94 µM. The rest of the compounds exhibited moderate anti-proliferative activities against the three tested cell lines.

#### 2.2.2. Structure activity relationship (SAR)

The biological testing results provided us with a valuable SAR. Regarding the cyclohexyl bearing derivatives, it was noticed that compound **7a** (incorporating *N*-cyclohexylsemicarbazide moiety) was more potent than compound **8b** (incorporating *N*-cyclohexylthiosemicarbazide moiety) in both cytotoxic and Topo II inhibitory effects, as well. However, the later compounds were more active that their counterparts **7b** (bearing a phenylsemicarbazide moiety) and **8a** (bearing an ethylthiosemicarbazide moiety), respectively. For benzylidenehydrazine derivatives (compounds **9a-d**), the effect of the substitution on the aromatic moieties in the order of 4-NO_2_ (**9b**) > 4-Cl (**9a**) > 4-OH (**9c**) > 4-F (**9b**). With regard to bis([1, 2, 4]triazolo)[3,4-a:4′,3′-*c*]phthalazine-3-thiol derivatives (compounds **14a-c**), the activities decreased in the order of the substitution with 4-COOH (**14a**) > 4-NO_2_ (**14b**) > 4-SO_2_NH_2_ (**14c**).

#### 2.2.3. Dna intercalation assay (DNA/methyl green colorimetric assay)

DNA/methyl green assay was carried out for the synthesised derivatives using doxorubicin as a positive control following the reported procedure described by Burre *et al.*^41^, to give extra quantitative data about the binding affinity of the target compounds towards the DNA molecules. DNA‐binding affinities of the target compounds were represented as IC_50_ values and are summarised in Table 2.

**Table 2.** DNA intercalating affinity and IC_50_ values of the tested compounds against DNA and Topo II, respectively.

Comp. No.	DNA/methyl green (IC_50_) (µM)^a,b^	Topoisomerase II (IC_50_) (µM)^a,c^
**7a**	37.14 ± 2.0	NT^d^
**7b**	36.57 ± 1.82	NT^d^
**8a**	29.63 ± 1.41	22.28 ± 2.00
**8b**	34.65 ± 1.10	8.91 ± 0.77
**9a**	43.81 ± 2.22	27.66 ± 2-51
**9b**	49.93 ± 2.53	NT^d^
**9c**	62.18 ± 2 .20	21.39 ± 1.90
**9d**	26.19 ± 1.14	7.02 ± 0.54
**14a**	28.74 ± 1.71	7.64 ± 0.66
**14b**	34.35 ± 2.80	13.66 ± 1.02
**14c**	46.34 ± 2.30	NT^d^
**15**	71.15 ± 3.11	NT^d^
**Doxorubicin**	31.27 ± 1.8	9.65 ± 0.77

^a^Three independent experiments were performed for each concentration.

^b^50% Inhibition concentration values of DNA/methyl green assay.

^c^50% Inhibition of Topo II.

^d^Not tested.

Compounds **8a**, **9d,** and **14a** exhibited excellent DNA binding affinities more than the reference drug with IC_50_ values of 29.63 ± 1.41, 26.19 ± 1.10, and 28.74 ± 1.71 µM, respectively. In addition, compounds **7a**, **7b**, **8b**, and **14b** showed remarkable activities but slightly less than the reference drug with IC_50_ values of 37.14 ± 2.0, 36.57 ± 1.8, 34.65 ± 1.1, and 34.35 ± 2.80 µM, respectively. Moreover, some compounds as **9a**, **9b**, and **14c** showed moderate activities with IC_50_ values ranging from 43.81 ± 2.20 to 49.93 ± 2.53 µM. Finally, compounds **9c** and **15** exhibited weak affinities towards DNA with IC_50_ values ranging from 62.18 ± 2.20 to 71.15 ± 3.11 µM, respectively.

#### 2.2.3. Topoisomerase II inhibitory activity

Seven compounds that exhibited significant DNA binding affinities (**8a**, **8b**, **9a, 9c, 9d, 14a,** and **14b**) were further estimated to determine their inhibitory activities towards topoisomerase II. The activity of topoisomerase II was determined according to the reported procedure described by Patra *et al.*^42^. Doxorubicin was utilised as a positive control in this test. The results were reported as IC_50_ values and summarised in Table 2. Compounds **8b**, **9d**, and **14a** was found to be the most potent derivatives with IC_50_ values of 8.91 ± 0.77, 7.02 ± 0.54, and 7.64 ± 0.66 µM, which were more active than the reference drug, doxorubicin (IC_50_ = 9.65 ± 0.77 µM). The other tested compounds, **8a**, **9a**, **9c**, and **14 b,** exhibited moderate to weak activities with high IC_50_ values ranging from 13.66 ± 1.02 to 13.66 ± 1.02 µM.

#### 2.2.4. *In vivo* antitumor activity

To examine the *in vivo* anticancer activity of compound **9d**, adult female Swiss albino mice (30 mice) inoculated with I.P. injection of Solid Ehrlich Carcinoma (SEC) tumour cell lines in a volume of 0.2 ml physiological saline contains 1 × 10^6^ viable cells for 24 h.

**Figure 3.**
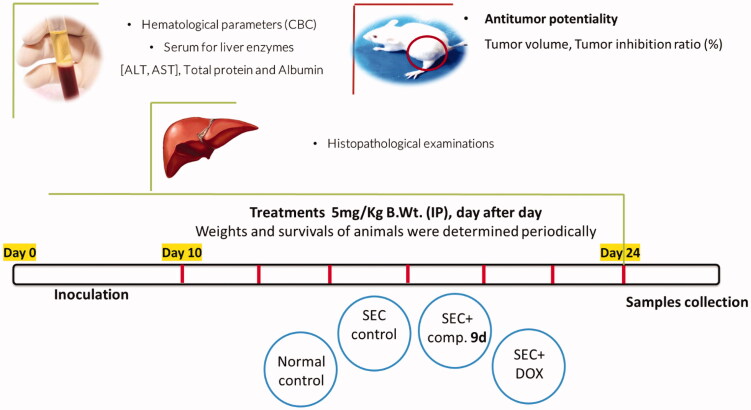
Methodology and Experimental design of the *in vivo* study.

These mice were randomly divided into four groups (7 mice/group). The 1^st^ group (normal saline-control group) was used as a negative control, the 2^nd^ group (the SEC-control group) was injected with the SEC, the 3^rd^ group (compound-treated group) was injected with SEC then with compound **9d**, and the 4^th^ group was injected with the SEC then with a standard anticancer drug, doxorubicin (DOX), as described in the experimental section. Bodyweight and survival were recorded daily until the 24^th^ day in both treated and control groups. At the end of the experiment, the blood of each group was collected under light anaesthesia for the estimation of hematological and biochemical assays. The anaesthetised animals were then sacrificed to evaluate of the antitumor activity and to conduct hematological, biochemical, and histopathological assays, Figure 3.

##### 2.2.4.1. Antitumor potentiality

**Figure 4.**
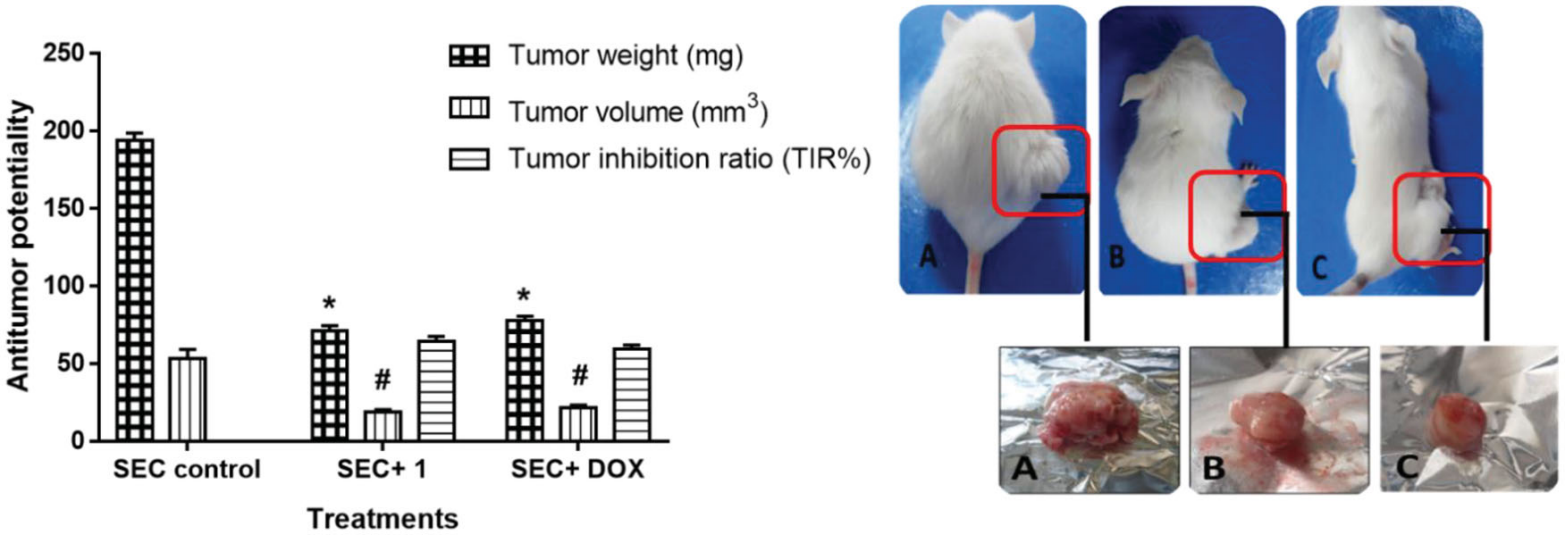
**Left panel:** Bar chart representation of the effect of compound **9d** treatment on the proliferation of solid tumour mass in the SEC-bearing mice. **Right panel:** Morphological representation for the tumour mass volume of **A**: SEC-group, **B**: SEC+ **9d**, and **C**: SEC + DOX. Values are expressed as Mean ± SEM values of mice in each group (*n* = 7). Signs of * and ^#^ are values with significant differences in tumour weight and tumour volume, respectively compared to SEC control using an unpaired t-test (*P* ≤ 0.05) using GraphPad prism.

*In vivo* anticancer activity of the compound, **9d** was estimated against SEC development. At first, the tumour development caused a 194 mg increase in solid tumour weight during the experimental period. During this study, treatment with compound **9d** and doxorubicin significantly reduced the increase in the solid tumour mass by 63.4 (71 mg) and 59.8% (78 mg), compared to control as represented in Figure 3. Treatment with Compound **9d** significantly inhibited tumour inhibition ratio (TIR) % by 64.5 in tumour volume (19 mm^3^) compared to doxorubicin (DOX) treatment with TIR% of 59 (22 mm^3^), compared to control. This indicated that compound **9d** and doxorubicin had a significant antitumor effect, Figure 4.

##### 2.2.4.2. Hematological and biochemical assays (Blood parameters assay)

At the end of the experiment, animals from different groups were sacrificed, and blood samples were collected for hematological parameters, including Hb, RBC’s, and WBC’s levels, and serum for determination of liver enzymes ALT, AST levels, and proteins.

Liver enzymes ALT and AST were significantly increased to 63.4, 64.67 (U/L), respectively, following tumour inoculation as shown in Table 3, compared with normal mice at 45.14 and 53.67 (U/L) because of hepatocellular damage. While liver protein and albumin were decreased to 6.13 and 2.97 (g/dL). Treatment with compound **9d** substantially reduced liver enzymes to 42.9, 55.6 U/L, respectively, and increased liver protein and albumin to 8.04 and 6.25 (g/dL), indicating a remarkable amelioration in the hepatocellular functions.

**Table 3.** Biochemical and hematological parameters in the tested groups.

Parameter/ Treatment	Biochemical parameters				Hematological parameters		
	ALT (U/L)	AST (U/L)	Total Protein (g/dL)	Albumin (g/dL)	Hb (g/dL)	RBCs count (×10^6^/µL)	WBCs count (×10^3^/µL)
Normal control	45.14 ± 2.69	53 ± 2.7	9.88 ± 0.35	5.95 ± 0.44	9.09 ± 0.61	6.08 ± 0.77	4.28 ± 0.44
SEC control	63.4 ± 4.53	64.67 ± 3.6	6.13 ± 0.24	2.97 ± 0.17	5.36 ± 0.41	3.33 ± 0.57	6.21 ± 0.57
SEC + 9d (5 mg/kg BW)	42.9^#^±1.01	55.6^#^±3.1	8.04^#^±0.41	6.25^#^±0.53	8.2^#^±0.31	5.37 ± 0.37	3.72^#^±0.46
SEC + DOX (5 mg/kg BW)	38.67^#^±1.6	48.4^#^±3.3	6.73 ± 0.26	6.01^#^±0.22	7.82 ± 0.27	5.26 ± 0.38	4.11 ± 0.57

Values are expressed as Mean ± SEM (*n* = 7).

^#^Significant difference between treated groups and SEC control using unpaired t-test (*P* ≤ 0.05) using the GraphPad prism7.

In terms of hematological parameters in SEC-bearing mice, all CBC parameters were changed in the SEC control, with Hb content and RBCs significantly decreased to 5.36 (g/dL) and 3.33 (10^6^/µL), respectively. When compared to normal control levels, the WBC count was significantly increased to 6.21 (10^3^/µL). Tumour propagation is routinely associated with decreased haemoglobin, RBC, and WBC counts^43^^,^^44^. After treatment with compound **9d**, CBC levels were nearly restored to normal, where it elevated the Hb (8.2 g/dL), RBC’s (5.37 10^6^/µL) and reduced the WBC’s (3.72 10^3^/µl) levels.

Interestingly our results following previous studies^45^^,^^46^, illustrated the anticancer activity by improving hematological and biochemical parameters after treatment with the tested compound. Taken together, treatment of SEC mice with compound **9d** improved hematological and biochemical parameters, as well as tumour weight and volume.

##### 2.2.4.3. Histopathological examinations

**Figure 5.**
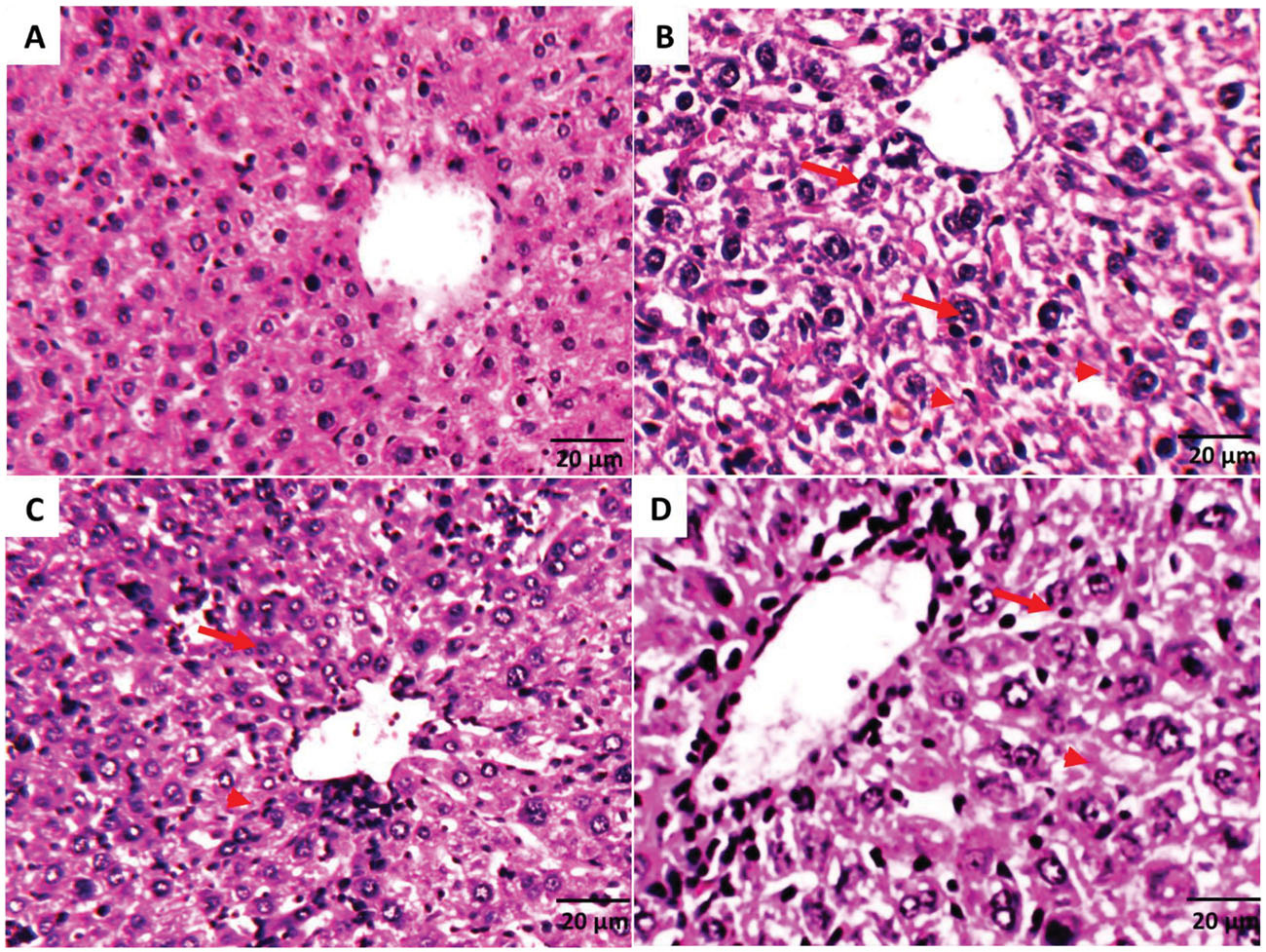
Histopathological examinations of liver tissues of SEC-bearing mice in different treatments (A) Normal control group that shows the normal structure of central vein surrounded with hepatocytes. (B) SEC control group shows pyknosis (arrows) & karyolysis (arrowhead), hydropic degeneration of hepatocytes, and loss of cell boundaries. (C) SEC group treated with **9d** (5 mg/Kg BW) that shows hepatic cells are near normal and show tissue improvement as compared with a little hydropic degeneration. (D) SEC group treated with DOX shows tissue enhancement like normal group, but still, some hydropic degeneration, pyknosis (arrows) and karyolysis (arrowhead) were shown. (H&E stain, magnification ×200).

Histopathological examinations of liver tissues of the SEC-bearing mice in different treatments were illustrated in Figure 5. According to compound **9d** ability to improve liver enzymes and proteins, its treatment was able to keep liver structure close to normal.

### 2.3 *In silico* studies

#### 2.3.1. Docking studies

**Figure 6.**
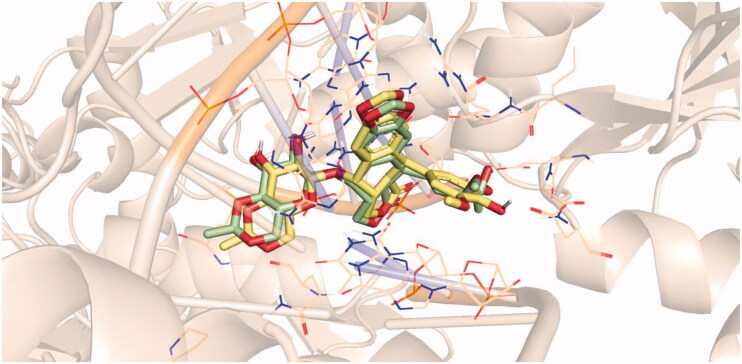
Superimposition of the co-crystallised ligand (light green) and the docking pose (light yellow) of the same molecule.

Molecular docking studies were performed to shed light on the binding modes of the newly synthesised compounds inside the DNA binding site of Topo II (PDB ID: 3qx3). Docking investigation was carried out using Discovery Studio 2.5 software. An X-ray crystallographic structure of Topo II with its co-crystallised ligand, etoposide, was downloaded from the Protein Data Bank (PDB). Re-docking of the co-crystalized ligand was initially performed aiming to validate the used docking protocol. The simulation of the re-docked ligand successfully regenerated the same binding mode of the co-crystalized one inside the DNA binding site of Topo II with RMSD of 0.81 Å, which indicates the validity of the docking process, Figure 6.

**Figure 7.**
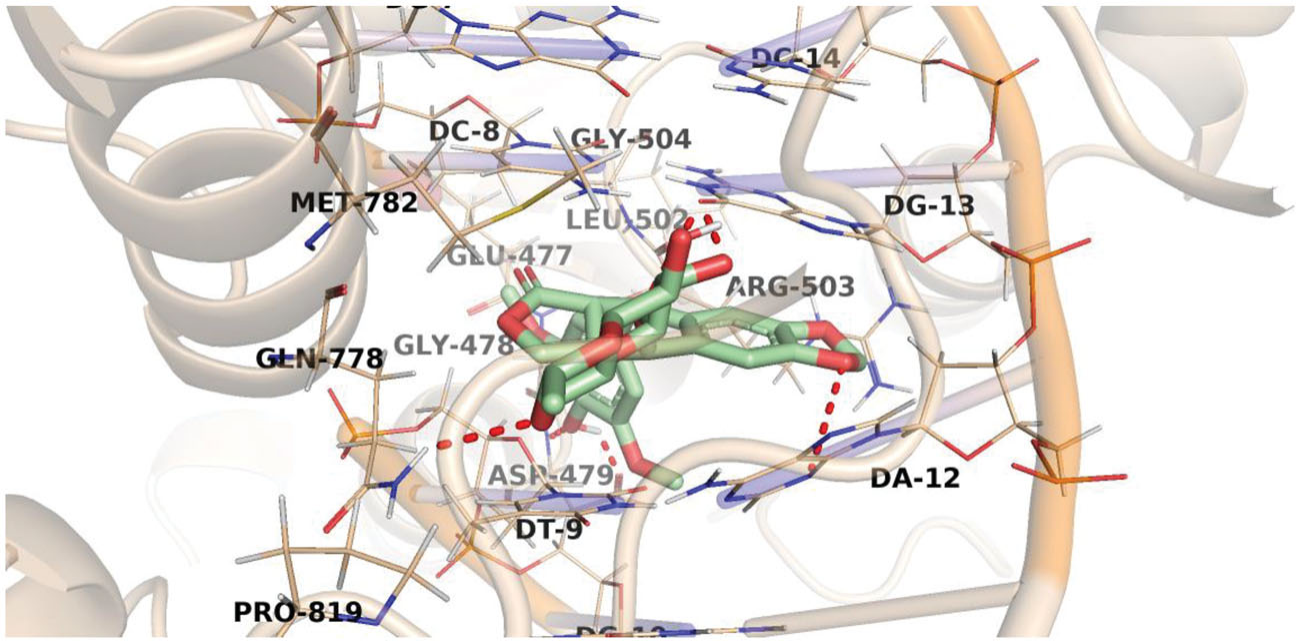
Binding of etoposide with DNA-Topo II, the hydrogen bonds are represented in red dashed lines.

The predicted binding pattern of the co-crystallised ligand, etoposide, revealed an affinity value of −30.13 kcal/mol with the formation of six H-bonds. The planar aromatic system occupied the hydrophobic pocket formed by Glu477, Gly478, Asp479, Leu502, Arg503, Gln778, Met782, and Pro819. It was also stacked between different DNA nucleotides, namely, Cytosine (DC-8 and DC-14), Guanine (DG-7, DG-10, and DG-13), Adenine (DA-12), and Thymine (DT-9). The sugar moiety of etoposide was directed towards the DNA minor groove and stabilised by the formation of two H-bond interactions with Gln778 and DG-13. Similarly, its phenolic OH group formed two H-bond interactions with Asp479. Two H-bonds were also formed between the etoposide oxygen atoms and the DNA nucleotides DG-13 and DA-12 Figure 7.

**Figure 8.**
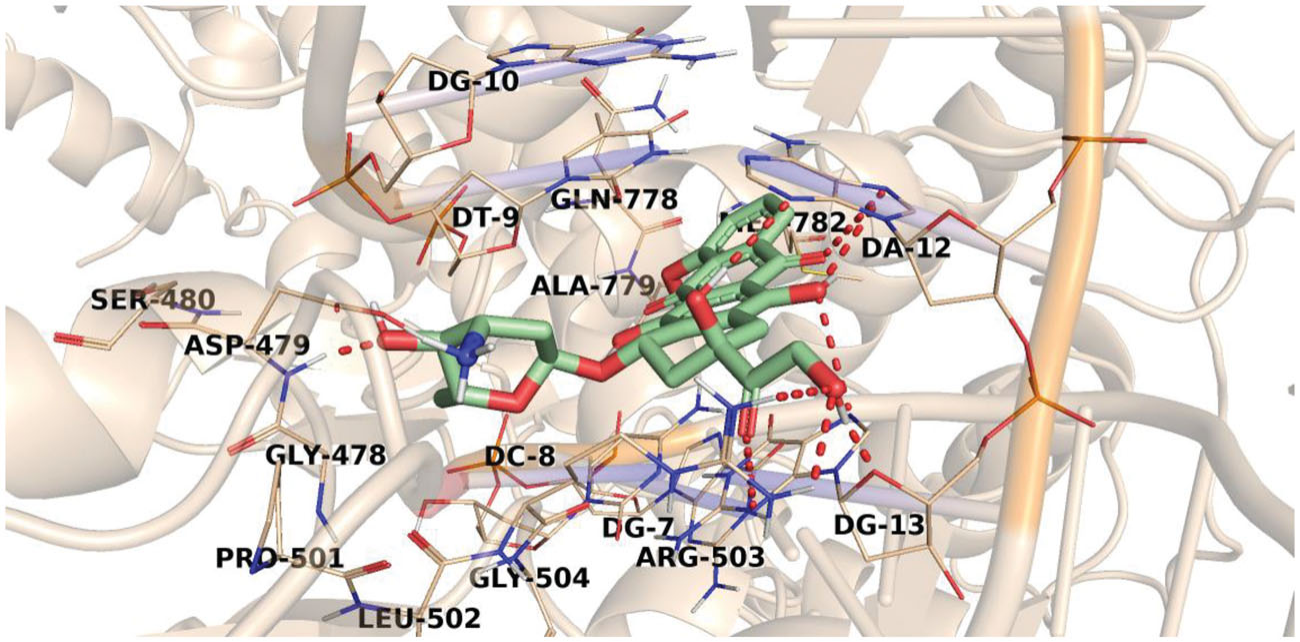
Binding of doxorubicin with DNA-Topo II, the hydrogen bonds are represented in red dashed lines.

The proposed binding mode of doxorubicin, with an affinity value of −33.50 kcal/mol, revealed that the doxorubicin planar aromatic chromophore formed aromatic stacking interactions with the different key residues Glu477, Gly478, Asp479, Leu502, Arg503, Gln778, Met782 in addition to the DNA nucleotides DT-9, DC-8, DC-11, DG-13, and DA-12. The sugar moiety of doxorubicin was oriented into the minor groove of DNA and stabilised by two H-bonds with Asp479. The rest of the compound was involved in several H-bond interactions with Arg503, DG-13, and DA-12, Figure 8.

A general investigation of docking results revealed that the designed compounds displayed a binding pattern comparable to that of the native ligand with predicted binding energy scores ranging from −18.49 to −29.91 kcal/mol.

**Figure 9.**
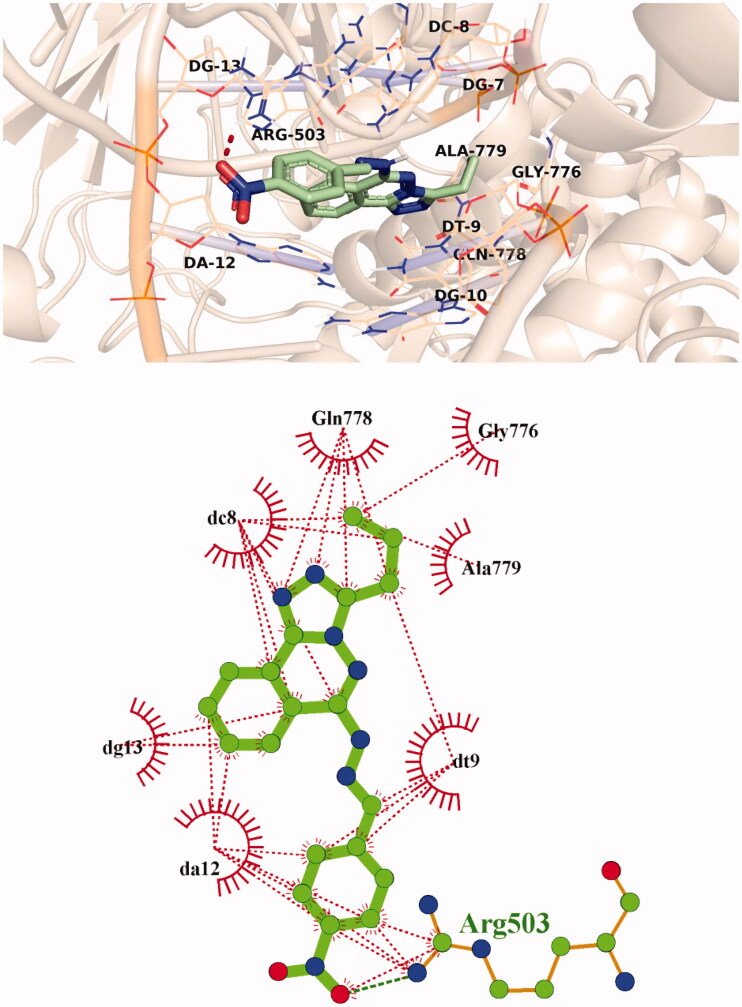
3D and 2D illustration of compound **9d** in the Topo II active site.

The predicted binding mode of compound **9d** as illustrated in Figure 9. Its triazolo phthalazine planner moiety was inserted between the DNA nucleotides with the formation of many hydrophobic interactions with DT-9, DC-8, DG-13, and DA-12 as well as Gly776, Gln778, and Ala779 amino acids. In addition, the 4-nitrophenyl part was oriented in the minor groove of DNA, forming hydrophobic interactions with DA-12 and Arg503. The nitro group of **9d**, however, interacted with Arg503 *via* an H-bond interaction.

**Figure 10.**
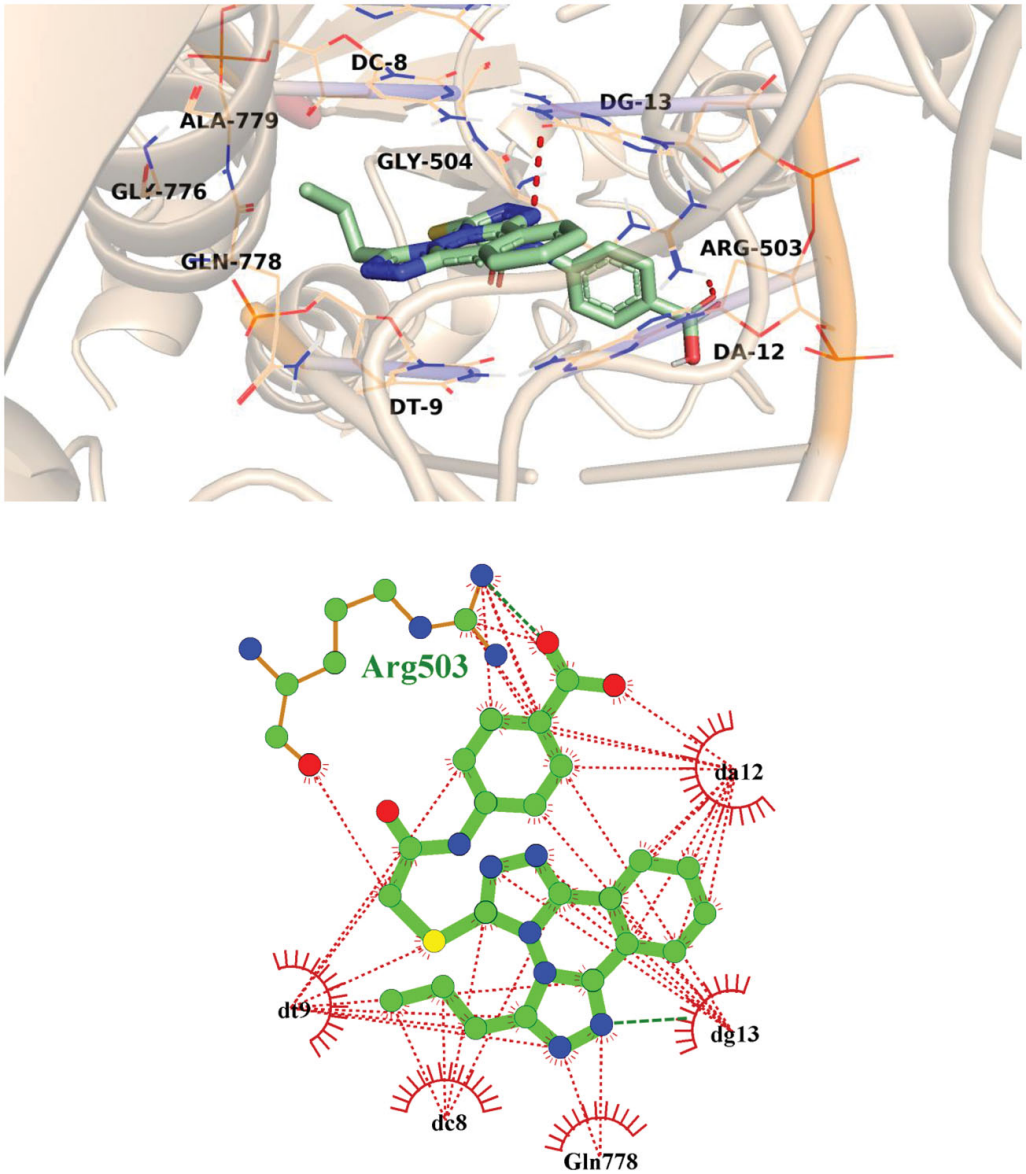
3D and 2D illustration of compound **14a** in the Topo II active site.

Compound **14a** showed an affinity value of −27.36 kcal/mol. The planar aromatic system occupied the hydrophobic pocket formed by DT-9, DC-8, DG-13, and DA-12 nucleotides in addition to Arg503, Gly504, Gly776, Gln778, and Ala779 residues forming several pi-pi interactions. The benzoic acid moiety was directed towards the DNA minor groove with the formation of H-bond interaction with Arg503 residue Figure 10.

#### 2.3.2. *In silico* ADMET analysis

ADMET studies were carried out for the synthesised compounds using doxorubicin as a reference compound. The predicted ADMET parameters were listed in Table 4.

**Table 4.** Predicted ADMET profile for the synthesised compounds

Comp.	BBB level^a^	Solubility level^b^	Absorption level^c^	CYP2D6 prediction^d^	PPB prediction^e^
**7a**	4	2	0	false	true
**7b**	3	2	0	false	true
**8a**	2	2	0	false	true
**8b**	1	1	0	false	true
**9a**	1	1	0	true	true
**9b**	1	1	0	true	true
**9c**	2	2	0	false	false
**9d**	4	1	1	false	true
**14a**	4	2	2	false	false
**14b**	4	1	2	false	false
**14c**	4	1	2	false	true
**15**	3	2	0	false	false
**Doxorubicin**	4	2	3	false	false

^a^BBB level, blood brain barrier level, 0 = very high, 1 = high, 2 = medium, 3 = low, 4 = very low.

^b^Solubility level, 1 = very low, 2 = low, 3 = good, 4 = optimal.

^c^Absorption level, 0 = good, 1 = moderate, 2 = poor, 3 = very poor.

^d^CYP2D6, cytochrome P2D6, TRUE = inhibitor, FALSE = non inhibitor.

^e^PBB, plasma protein binding, FALSE means less than 90%, TRUE means more than 90%.

**Figure 11.**
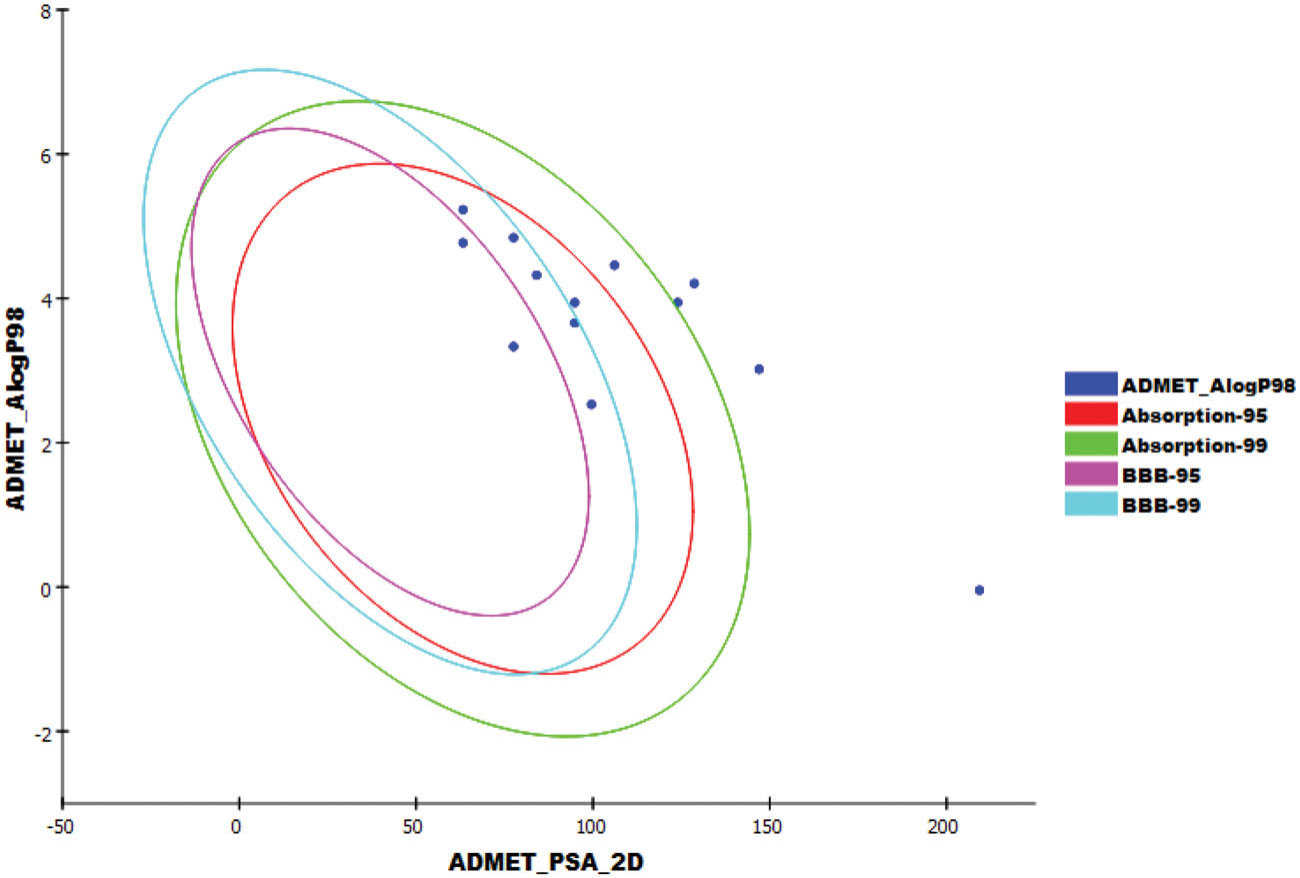
The expected ADMET study of the target compounds.

The results revealed that compounds **7_a_, 9_d_, 14_a_, 14 _b_,** and **14_c_** had very low Blood Brain Barrier penetration power. Accordingly, such compounds were expected to be safe to CNS. Aqueous solubility of the synthesised compounds ranged from low to very low. Compounds **7a**, **7 _b_, 8_a_, 8 _b_, 9_a_, 9 _b_, 9_c_,** and **15** showed good absorption level. Except compounds **9a** and **9b**, all members were predicted as non-inhibitors of CYP2D6. Except compounds **9_c_, 14_a_, 14 _b_,** and **15**, all compounds were expected to bind plasma protein more than 90% (Figure 11).

#### 2.3.3. Toxicity studies

Toxicity prediction was carried out based on the validated and constructed models in Discovery studio software^47^^,^^48^. As shown in Table 5, most compounds showed *in silico* low adverse effects and toxicity against the tested models. Regarding developmental toxicity potential (DTP), all the tested compounds were predicted to be non-toxic. For Carcinogenic Potency TD_50_ (Rat), all compounds showed higher values (from 0.873 to 34.570 mg/kg body weight/day) than that of doxorubicin (0.861 mg/kg body weight/day) except compound **7a** (0.651 mg/kg body weight/day). For rat maximum tolerated dose model, compounds **7 b, 8a, 8 b, 9a, 9 b,** and **9c** showed higher levels than doxorubicin. The tested compounds showed high oral LD_50_ values ranging from 0. 0.229to 12.326 mg/kg body weight/day which were higher than that of doxorubicin **(**0.227 mg/kg body weight/day) Moreover, except compounds **9a** and **9d**, all compounds were predicted to be mild and non-irritant against ocular irritancy and skin irritancy models, respectively.

**Table 5.** Toxicity properties of the synthesised compounds

Comp.	DTP	Carcinogenic Potency TD_50_ (Rat)^a^	Rat Maximum Tolerated Dose (Feed)^b^	Rat Oral LD_50_^b^	Ocular Irritancy	Skin Irritancy
**7a**	Non-Toxic	0.651	0.222	0.861	Mild	Non-Irritant
**7b**	Non-Toxic	19.886	0.369	2.513	Mild	Non-Irritant
**8a**	Non-Toxic	34.570	0.366	0.742	Mild	Non-Irritant
**8b**	Non-Toxic	0.873	0.260	0.297	Mild	Non-Irritant
**9a**	Non-Toxic	2.505	0.290	0.447	Mild	Irritant
**9b**	Non-Toxic	2.668	0.311	0.229	Mild	Non-Irritant
**9c**	Non-Toxic	20.483	0.779	0.501	Mild	Non-Irritant
**9d**	Non-Toxic	1.991	0.177	0.573	Mild	Irritant
**14a**	Non-Toxic	25.171	0.429	2.371	Mild	Non-Irritant
**14b**	Non-Toxic	5.431	0.090	1.863	Mild	Non-Irritant
**14c**	Non-Toxic	22.020	0.095	12.326	Mild	Non-Irritant
**15**	Non-Toxic	23.275	0.118	0.542	Mild	Non-Irritant
**Doxorubicin**	Toxic	0.861	0.277	0.227	Mild	Non-Irritant

^a^Unit: mg/kg body weight/day.

^b^Unit: g/kg body weight.

## 3. Conclusion

A new series of phthalazine derivatives was designed hoping to discover novel Topo II inhibitor and DNA intercalator agents as well. Twelve compounds were synthesised and tested *in vitro* for their anti-proliferative activities against three human cancer cell lines, HepG-2, MCF-7, and HCT-116. The tested members exhibited a promising cytotoxic effect with IC_50_ values ranging from 4.35 ± 0.19 to 78.49 ± 2.04 µM. All compounds were further estimated for their *in vitro* DNA intercalating effects. Amongst, seven compounds were further examined for their *in vitro* inhibitory activity against Topo II enzyme. Three compounds, **8b**, **9d**, and **14a**, out of the seven exhibited potent Topo II inhibitory activities with IC_50_ values of 8.91 ± 0.77, 7.02 ± 0.54, and 7.64 ± 0.66 µM, respectively. Finally, *in vivo* antitumor studies were carried out for compound **9d**. *In vivo* study exhibited that treatment with compound **9d** substantially inhibited tumour proliferation reducing solid tumour volume and mass. Additionally, it restored liver enzymes, proteins, and CBC parameters near-normal, indicating a remarkable amelioration in their functions along with histopathological examinations. Hence, compound **9d** was investigated as a novel anti-cancer agent through Topo II inhibition and DNA-binding affinity. To conclude, compounds presented in the current study were proved to be potent Topo II inhibitors with DNA intercalating efficacy that can be further adopted for hit optimisation and/or lead discovery.

## 4. Experimental

### 4.1. Chemistry

Starting materials and reagents were purchased from Sigma-Aldrich and used without purification. Melting points measurement was carried out by a Gallen lamp melting point apparatus and are uncorrected. Reactions progress was monitored by TLC (Merck, Germany), the spots were detected by exposure to UV lamp at λ 254 nm. IR spectra were recorded by pye Unicam SP 1000 IR spectrophotometer using KBr discs and expressed in wavenumber (cm^−1^). ^1^H and ^13 ^C NMR spectra were recorded with Bruker Advance 400 spectrophotometer operating at 400 MHz and 100 MHz, respectively and the chemical shifts were given in *δ* as parts per million (ppm) downfield from tetramethylsilane (TMS) as internal standard. The mass spectra were recorded on Varian MAT 311-A (70 e.v.).

The previously reported compounds 2,3-dihydrophthalazine-1,4-dione **2**^34^, 1,4-dichlorophthalazine **3**^35^ and 1-chloro-4-hydrazineylphthalazine **4**^35^, 6-chloro-3-propyl-[1, 2, 4]triazolo[3,4-a]phthalazine **5**^49^, and *N*-aryl-2-chloroacetamide **11a-c**^50^ were synthesised following the described procedures.

#### 4.1.1. 6-Hydrazineyl-3-propyl-[1, 2, 4]triazolo[3,4-*a*]phthalazine 6

To a boiling solution of hydrazine hydrate 70% (3.73 ml, 0.074 mol) in ethanol (50 ml), 6-chloro-3-propyl-[1, 2, 4]triazolo[3,4-a]phthalazine **5** (2.46 g, 0.01 mol) was added. The reaction mixture was refluxed for 0.5 h then cooled. The obtained precipitate was filtered, washed with petroleum ether (3 × 20 ml), dried, and recrystallized from ethanol to obtain compound **6**.

White crystals (yield 83%); m.p. 259–261 °C; IR (KBr) *ν* cm-1: 3329, 3143, 3136; ^1^H NMR (DMSO-*d*_6_) *δ* ppm: 0.98 (t, *J* = 7.2 Hz, 3H, CH_3_), 1.36 (m, 2H, CH_2_), 3.01 (t, *J* = 7.2 Hz, 2H, CH_2_), 3.75 (br s, 2H, exchangeable with D_2_O, NH_2_), 7.77 (dd, *J* = 8.4, 7.2 Hz, 1H, Ar-H,), 7.89 (dd, *J* = 7.2, 8.0 Hz, 1H, Ar-H), 8.24 (d, *J* = 8.4 Hz, 1H, Ar-H), 8.36 (d, *J* = 8.0 Hz, 1H, Ar-H), 8.95 (s, 1H, exchangeable with D_2_O, NH); Mass (*m*/*z*): 242.51 (M^+^, 16%), 143.05 (100%).

#### 4.1.2. General procedure for the synthesis of target compounds 7a,b

A mixture of the hydrazinyl compound **6** (0.242 g, 0.001 mol) and the appropriate isocyanate namely, cyclohexyl isocyanate and phenyl isocyanate (0.001 mol) were refluxed in absolute ethanol (25 ml) for 3 h. The solution was cooled. Then, the obtained solid was filtered and recrystallized from ethanol to produce compounds **7a,b**, respectively.

##### 4.1.2.1. *N*-Cyclohexyl-2–(3-propyl-[1, 2, 4]triazolo[3,4-*a*]phthalazin-6-yl)hydrazine-1-carboxamide 7a

White crystals (yield 89%); m.p. 269–271 °C; IR (KBr) *ν* cm^−1^: 3290, 3271, 3147, 1674; ^1^H NMR (DMSO-*d6*) *δ ppm:* 0.95 (t, 3H, CH_3_), 1.06 (m, 2H, CH_2_), 1.14 (m, 2H, CH_2_), 1.19 (m, 3H, CH_3_), 1.53 (m, 2H, CH_2_), 1.65 (m, 2H, CH_2_), 1.75 (m, 2H, CH_2_), 2.94 (t, *J* = 7.6 Hz, 2H, -CH_2_), 3.44 (m, 1H, CH), 6.48 (s, 1H, exchangeable with D_2_O, NH), 7.68 (s, 1H, exchangeable with D_2_O, -NH), 7.84 (dd, *J* = 8.0, 7.6 Hz, 1H, Ar-H), 7.91 (dd, *J* = 7.6, 7.6 Hz, 1H, Ar-H,), 8.35 (d, *J* = 8.0 Hz, 1H, Ar-H), 8.41 (d, *J* = 7.6 Hz, 1H, Ar-H), 9.41 (s, 1H, exchangeable with D_2_O, NH); MS (*m/z*): 367.19 (M^+^, 14.7%), 332 (100%, base peak).

##### 4.1.2.2. *N*-Phenyl-2–(3-propyl-[1, 2, 4]triazolo[3,4-*a*]phthalazin-6-yl)hydrazine-1-carboxamide 7 b

White crystals (yield 82%); mp: 258–260 °C; IR (KBr) ν cm^−1^: 3271, 3217, 3147, 1662; ^1^H NMR (DMSO-d6) *δ* ppm: 0.97 (t, *J* = 7.6 Hz, 3H, CH_3_), 1.72 (m, 2H, CH_2_), 2.95 (t, *J* = 7.6 Hz, 2H, CH_2_), 6.95 (t, 1H, Ar-H), 7.25 (t, 2H, Ar-H), 7.4 (d, 2H, Ar-H), 7.88 (dd, *J* = 7.2, 8.0 Hz, 1H, Ar-H), 7.99 (dd, *J* = 8.0, 7.2 Hz, 1H, Ar-H), 8.40 (d, *J* = 8.0 Hz, 1H, Ar-H), 8.43 (d, *J* = 8.0 Hz, 1H, Ar-H), 8.18 (s, 1H, exchangeable with D_2_O, NH), 8.76 (s, 1H, exchangeable with D_2_O, NH), 9.64 (s, 1H, exchangeable with D_2_O, NH); ^13 ^C NMR (DMSO-*d*6) *δ* ppm: 11.40, 13.61, 17.82, 117.82, 123.06, 124.17, 124.99, 127.20, 129.30 (2 C), 129.33, 130.03, 130.64, 133.60, 135.16, 141.93, 145.59, 148.75, 151.17.

#### 4.1.3. General procedure for the synthesis of target compounds 8a,b

A mixture of compound **6** (0.242 g, 0.001 mol) and appropriate isothiocyanate namely, ethyl isothiocyanate, and cyclohexyl isothiocyanate (0.001 mol) in absolute ethanol (20 ml) were heated under reflux for 3 h. After cooling, the precipitate was collected, dried, and recrystallized from ethanol to afford compounds **8a,b**, respectively.

##### 4.1.3.1. *N*-Ethyl-2–(3-propyl-[1, 2, 4]triazolo[3,4-*a*]phthalazin-6-yl)hydrazine-1-carbothioamide 8a

White crystals (yield 69%); mp: 242–244 °C; IR (KBr) *ν* cm^−1^: 3365, 3273, 3228; ^1^H NMR (DMSO-*d_6_*) *δ ppm:* 0.95 (t, *J* = 6.4 Hz, 3H, CH_3_), 1.02 (t, *J* = 6.0 Hz, 3H, CH_3_), 1.67 (t, *J* = 7.6 Hz, 2H, CH_2_), 2.92 (q, *J* = 7.6 Hz, 2H, -CH_2_), 3.48 (q, *J* = 6.0 Hz, 2H, CH_2_), 7.80 (dd, *J* = 7.6, 7.2 Hz, 1H, Ar-H), 7.91 (dd, *J* = 7.2, 7.2 Hz, 1H, Ar-H), 8.25 (d, *J* = 7.6 Hz, 1H, Ar-H), 8.31 (s, 1H, exchangeable with D_2_O, NH), 8.34 (d, *J* = 7.2 Hz, 1H, Ar-H), 9.27 (s, 1H, exchangeable with D_2_O, NH), 9.68 (s, 1H, exchangeable with D_2_O, NH); ^13 ^C NMR (DMSO-*d*6) *δ* ppm: 11.37, 14.94, 17.78, 19.56, 38.86, 117.66, 122.72, 123.62, 124.85, 130.45, 133.52, 142.02, 150.97, 151.44, 182.20.

##### 4.1.3.2. *N*-Cyclohexyl-2–(3-propyl-[1, 2, 4]triazolo[3,4-*a*]phthalazin-6-yl)hydrazine-1-carbothioamide 8 b

White crystals (yield 80%); mp: 221–223 °C; IR (KBr) *ν* cm^−1^: 3367, 3242, 2924; ^1^H NMR (DMSO-*d_6_*) *δ ppm:* 0.94 (m, 1H, CH), 0.97 (t, 3H, CH_3_), 1.23 (m, 4H, 2CH_2_), 1.49 (m, 1H, CH), 1.62 (m, 2H, CH_2_), 1.71 (m, 2H, -CH_2_), 1.79 (m, 2H, CH_2_), 2.93 (t, *J* = 7.6 Hz, 2H, CH_2_), 4.23 (m, 1H, CH), 7.69 (s, 1H, exchangeable with D_2_O, NH), 7.82 (dd, *J* = 7.7, 7.5 Hz, 1H, Ar-H), 8.02 (dd, *J* = 7.8, 7.7 Hz, 1H, Ar-H), 8.14 (d, *J* = 7.5 Hz, 1H, Ar-H), 8.25 (d, *J* = 7.8 Hz, 1H, Ar-H), 9.22 (s, 1H, exchangeable with D_2_O, NH), 9.63 (s, 1H, exchangeable with D_2_O, NH); MS (*m/z*): 383.21 (M^+^, 14.7%), 301.33 (100%, base peak).

#### 4.1.4. General procedure for the synthesis of target compounds 9a-d

Equimolar amounts of compound **6** (0.242 g, 0.001 mol) and the appropriate aldehyde namely 4-chlorobenzaldehyde, 4-flourobenzaldehyde, 4-hydroxybenzaldehyde, 4-nitrobenzaldehyde, (0.001 mol) were refluxed in absolute ethanol (25 ml) with a catalytic amount of glacial acetic acid for 4 h. The reaction was followed up by TLC. After the completion of the reaction, the mixture was cooled. The formed precipitate was filtered, dried, and recrystallized from ethanol to afford compounds **9a-d**, respectively.

##### 4.1.4.1. 6-[2–(4-Chlorobenzylidene)hydrazineyl]-3-propyl-[1, 2, 4]triazolo[3,4-*a*]phthalazine 9a

White crystals (yield 79%); mp: 251–253 °C; IR (KBr) *ν* cm^−1^: 3242, 3072; ^1^H NMR (DMSO-*d_6_*) *δ ppm:* 0.96 (t, *J* = 7.0 Hz, 3H, CH_3_), 1.45 (m, 2H, CH_2_), 3.09 (t, *J* = 7.6 Hz, 2H, CH_2_), 7.56 (2d, *J* = 8.4 Hz, 2H, Ar-H), 7.81 (2d, *J* = 8.8 Hz, 2H, Ar-H), 7.97 (dd, *J* = 8.0, 7.2 Hz, 1H, Ar-H), 8.02 (dd, *J* = 8.0, 8.8 Hz, 1H, Ar-H), 8.51 (d, *J* = 7.2 Hz, 1H, Ar-H), 8.54 (s, 1H, CH), 8.60 (d, *J* = 8.8 Hz, 1H, Ar-H), 11.56 (s, 1H, exchangeable with D_2_O, NH); ^13 ^C NMR (DMSO-*d*6) *δ* ppm: 11.40, 17.83, 21.61, 117.85, 123.08, 124.16, 124.96, 127.20, 128.75, 129.91, 130.68, 132.42, 133.61, 139.82, 141.94, 145.80, 148.76, 151.19, 161.68.

##### 4.1.4.2. 6-[2–(4-Fluorobenzylidene)hydrazineyl]-3-propyl-[1, 2, 4]triazolo[3,4-*a*]phthalazine 9 b

Yellowish white crystals (yield 71%); mp: 267–269 °C; IR (KBr) *ν* cm^−1^: 3180, 3064, 3031; ^1^H NMR (DMSO-*d_6_*) *δ ppm:* 0.99 (t, 3H, CH_3_), 1.59 (m, 2H, CH_2_), 3.10 (t, *J* = 7.6 Hz, 2H, CH_2_), 7.32 (2d, *J* = 8.8 Hz, 2H, Ar-H),7.83 (dd, *J* = 7.2, 7.6 Hz, 1H, Ar-H), 7.93 (2d, 2H, *J* = 8.8 Hz, Ar-H), 8.01 (dd, *J* = 7.6, 7.6 Hz, 1H, Ar-H), 8.52 (d, *J* = 7.2 Hz, 1H, Ar-H), 8.54 (d, *J* = 7.6 Hz, 1H, Ar-H), 8.72 (s, 1H, CH), 11.41 (s, 1H, exchangeable with D_2_O, NH).

##### 4.1.4.3. 4-[(2–(3-Propyl-[1, 2, 4]triazolo[3,4-a]phthalazin-6-yl)hydrazineylidene)methyl]-phenol 9c

Reddish white crystals (yield 81%); mp: 249–251 °C; IR (KBr) *ν* cm^−1^: 3421, 3213, 3066; ^1^H NMR (DMSO-*d_6_*) *δ ppm:* 0.96 (t, 3H, CH_3_), 1.59 (m, 2H, CH_2_), 3.04 (t, *J* = 7.6 Hz, 2H, CH_2_), 6.86 (2d, *J* = 8.0 Hz, 2H, Ar-H), 7.61 (2d, *J* = 8.0 Hz, 2H, Ar-H), 7.86 (dd, *J* = 7.2, 7.6 Hz, 1H, Ar-H), 7.99 (dd, *J* = 7.2, 7.6 Hz, 1H, Ar-H), 8.39 (s, 1H, CH), 8.44 (d, *J* = 7.6 Hz, 1H, Ar-H), 8.48 (d, *J* = 7.6 Hz, 1H, Ar-H), 9.90 (s, 1H, exchangeable with D_2_O, OH), 11.03 (s, 1H, exchangeable with D_2_O, NH); ^13 ^C NMR (DMSO-*d*6) *δ* ppm: 11.43, 17.84, 21.52, 117.90, 123.13, 124.24, 125.04, 127.23 (2 C), 129.96 (2 C), 130.73, 132.46, 133.68, 139.87, 141.98, 145.83, 148.82, 151.22.

##### 4.1.4.4. 6-[2–(4-Nitrobenzylidene)hydrazineyl]-3-propyl-[1, 2, 4]triazolo[3,4-*a*]phthalazine 9d

Yellow crystals (yield 83%); mp: 251–253 °C; **IR** (KBr) *ν* cm^−1^: 3217, 3078, 3047, 2931; ^1^H NMR (DMSO-*d_6_*) *δ ppm:* 0.98 (t, 3H, CH_3_), 1.59 (m, 2H, CH_2_), 2.62 (t, *J* = 6.8 Hz, 2H, CH_2_), 7.46 (dd, *J* = 8.0, 7.6 Hz, 1H, Ar-H), 7.56 (2d, 2H, *J* = 8.8 Hz, Ar-H), 7.58 (dd, *J* = 8.0, 8.4 Hz, 1H, Ar-H), 7.87 (2d, *J* = 8.8 Hz, 2H, Ar-H), 8.03 (d, *J* = 7.6 Hz, 1H, Ar-H), 8.08 (d, *J* = 8.4 Hz, 1H, Ar-H), 8.12 (s, 1H, CH), 11.90 (s, 1H, exchangeable with D_2_O, NH); ^13 ^C NMR (DMSO-*d*6) *δ* ppm: 11.40, 17.53, 21.55, 117.82, 123.06, 124.17, 124.99, 127.20 (2 C), 129.30 (2 C), 130.03, 130.64, 133.60, 135.16, 141.93, 145.59, 148.75, 151.17.

#### 4.1.5. 6-Propylbis([1, 2, 4]triazolo)[3,4-*a*:4',3'-*c*]phthalazine-3-thiol 12

A mixture of compound **6** (2.42 g, 0.01 mol), carbon disulphide (0.71 ml, 0.01 mol) and potassium hydroxide (0.56 g, 0.01 mol) was refluxed in absolute ethanol (20 ml) for 3 h. The mixture was then cooled to room temperature and poured onto 1 N HCl (l20 ml). The yellow precipitated product was filtered, washed with distilled water, dried, and crystallised from ethanol to give compound **12**.

Yellowish white crystal (yield 72%); mp > 300 °C; **IR** (KBr) *ν* cm^−1^: 3067, 2919, 2563, 1599; **^1^H NMR** (DMSO-*d_6_*) *δ ppm:* 0.96 (t, 3H, CH_3_), 1.60 (m, 2H, CH_2_), 3.40 (t, *J* = 6.8 Hz, 2H, CH_2_), 7.38 (dd, *J* = 6.4, 7.6 Hz, 1H, Ar-H), 7.43 (dd, *J* = 7.6, 6.4 Hz, 1H, Ar-H), 7.77 (d, *J* = 7.6 Hz, 1H, Ar-H), 7.94 (d, *J* = 7.6 Hz, 1H, Ar-H), 14.24 (s, 1H, exchangeable with D_2_O, SH); MS (*m/z*): 284.11 (M^+^, 16.90%), 173.33 (100%, base peak).

#### 4.1.6. Potassium 6-propylbis([1, 2, 4]triazolo)[3,4-*a*:4',3'-*c*]phthalazine-3-thiolate 13

A mixture of 20 (2.84 g, 0.01 mol) and potassium hydroxide (0.56 g, 0.01 mol) in absolute ethanol (20 ml) was heated with continuous stirring for 0.5 h. After cooling, a precipitate was produced. The precipitate was collected and washed with diethyl ether to afford the corresponding potassium salt **13**.

#### 4.1.7. General procedure for the synthesis of target compounds 14a-c and 15

A mixture of the potassium salt **13** (0.322 g, 0.001 mol) and the appropriate chloroacetanilides namely, 4–(2-chloroacetamido)benzoic acid **11a**, 2-chloro-*N*-(4-nitrophenyl)acetamide **11b**, 2-chloro-*N*-(4-sulfamoylphenyl) acetamide **11c,** or 2-chloroacetamide (0.001 mol) in dry DMF (20 ml) with a catalytic amount of potassium iodide was heated over a water bath for 3 h. The reaction mixture was then cooled, poured into ice water (50 ml) and stirred well for 1 h. The separated solid was filtered, washed with water, dried, and crystallised from ethanol to afford the corresponding derivatives **14a-c** and **15**, respectively.

##### 4.1.7.1. 4-[2-((6-Propylbis([1, 2, 4]triazolo)[3,4-*a*:4',3'-*c*]phthalazin-3-yl)thio)acetamido]benzoic acid 14a

White (yield 81%); mp: 244–246 °C; **IR** (KBr) *ν* cm^−1^: 3425, 3248, 3178, 1685, 1673; **^1^H NMR** (DMSO-*d_6_*) *δ ppm:* 0.99 (t, 3H, CH_3_), 1.67 (m, 2H, CH_2_), 3.47 (t, *J* = 6.8 Hz, 2H, CH_2_), 4.51 (s, 2H, SCH_2_), 7.79 (2d, *J* = 6.0 Hz, 2H, Ar-H), 8.03 (m, 4H, Ar-H), 8.56 (2d, *J* = 6.0 Hz, 2H, Ar-H), 10.64 (s, 1H, exchangeable with D_2_O, NH).

##### 4.1.7.2. *N*-(4-Nitrophenyl)-2-[(6-propylbis([1, 2, 4]triazolo)[3,4-*a*:4',3'-*c*]phthalazin-3-yl)thio]acetamide 14 b

Yellowish white crystals (yield 74%); mp: 277–279 °C; **IR** (KBr) *ν* cm^−1^: 3278, 3082, 1701; **^1^H NMR** (DMSO-*d_6_*) *δ ppm:* 0.97 (t, 3H, CH_3_), 1.55 (m, 2H, CH_2_), 3.65 (t, *J* = 6.8 Hz, 2H, CH_2_), 4.38 (s, 1H, SCH_2_), 7.78 (dd, *J* = 8.4 Hz, 2H), 7.93 (m, 2H, Ar-H), 8.24 (dd, *J* = 8.4, 2H, Ar-H), 8.45 (m, 2H, Ar-H), 10.95 (s, 1H, exchangeable with D_2_O, -NH); ^13 ^C NMR (DMSO-*d*6) *δ* ppm: 11.46, 17.84, 23.20, 40.76, 119.20 (2 C), 119.91, 120.39, 123.72, 123.66, 127.22 (2 C), 131.98, 132.29, 139.14, 141.91, 143.67, 145.52, 147.07, 149.78, 166.40.

##### 4.1.7.3. 2-[(6-Propylbis([1, 2, 4]triazolo)[3,4-*a*:4',3'-*c*]phthalazin-3-yl)thio]-*N*-(4-sulfamoyl-phenyl)acetamide 14c

Yellowish white crystals (yield 76%); mp: 245–247 °C; **IR** (KBr) *ν* cm^−1^: 3297, 3243, 3194, 1676; **^1^H NMR** (DMSO-*d_6_*) *δ ppm:* 0.97 (t, 3H, CH_3_), 1.57 (m, 2H, CH_2_), 3.67 (t, *J* = 6.0 Hz, 2H, CH_2_), 4.40 (s, 1H, SCH_2_), 7.39 (s, 2H, exchangeable with D_2_O, NH_2_), 7.73 (dd, *J* = 8.0 Hz, 2H, Ar-H), 7.84 (dd, *J* = 8.0, 2H, Ar-H), 7.91 (m, 2H, Ar-H), 8.39 (m, 2H, Ar-H), 10.74 (s, 1H, exchangeable with D_2_O, NH); **MS** (*m/z*): 496 (M^+^, 12.91%), 320 (100% base peak).

##### 4.1.7.4. 2-[(6-Propylbis([1, 2, 4]triazolo)[3,4-*a*:4',3'-*c*]phthalazin-3-yl)thio]acetamide 15

Yellowish white crystals (yield 73%); mp: 238–240 °C; **IR** (KBr) *ν* cm^−1^: 3194, 3084, 1655; **^1^H NMR** (DMSO-*d_6_*) *δ ppm:* 0.95 (t, 3H, CH_3_), 1.60 (m, 2H, CH_2_), 3.68 (t, *J* = 7.0 Hz, 2H, CH_2_), 4.18 (s, 2H, SCH_2_), 7.41 (s, 1H, exchangeable with D_2_O, **H**-N-H), 7.81 (s, 1H, exchangeable with D_2_O, H-N-**H**), 7.94 (m, 2H, Ar-H), 8.45 (d, 2H, Ar-H).

### 4.2. Biological evaluation

#### 4.2.1. *In vitro* anti-proliferative activity

Anti-proliferative activity of the synthesised compounds was estimated using the MTT assay protocol^8^^,^^38–40^ as shown in Supplementary data.

#### 4.2.2. Dna intercalation assay (DNA/methyl green colorimetric assay)

The DNA/methyl green assay was estimated *in vitro* for all the target derivatives using doxorubicin as a reference drug, adopting the protocol described by Burres *et al.*^41^ as shown in the Supplementary data.

#### 4.2.3. Measurement of topoisomerase II activity

Compounds (**8a**, **8b**, **9a**, **9c**, **9d**, **12a**, and **12b**) that showed the better results in anti-proliferative and DNA/methyl green assay were further evaluated for their *in vitro* inhibitory activities against Topoisomerase II using doxorubicin as a reference drug following to reported procedure described by Patra *et al.*^42^ as shown in the Supplementary data.

#### 4.2.4. *In vivo* antitumor activity

##### 4.2.4.1. Animals and tumour cell line

Adult female Swiss albino mice purchased from Theodor Bilharzia Research Institute, Giza, Egypt, with an average bodyweight of (18–23) g were used. Mice were housed under constant conditions of 12 h light/dark cycle in a temperature under conditions of controlled humidity (22 ± 2 °C), with free access to standard laboratory mice food and water. All procedures related to care and maintenance of the animals were performed according to the international guiding principles for animal research and approved by the Faculty of Science, Suez Canal University bioethics and animal ethics committee (Approval number REC-07–2021).

Solid Ehrlich carcinoma (SEC) was purchased from the National Cancer Institute (Cairo University, Egypt). The tumour cell line was proliferated in mice through serial intraperitoneal (I.P.) transplantation of a volume of 0.2 ml physiological saline contains 1 × 10^6^ viable cells for 24 h. SEC cells were collected 7 days after I.P. implantation. The harvested cells were diluted with saline to obtain a concentration of 5 × 10^6^ viable SEC cells/mL. A volume of 0.2 ml saline contains 1 × 10^6^ SEC cells that were I.P. implanted into each normal mouse. SEC cells (1 × 10^6^ tumour cells/mouse) were implanted subcutaneously into the right thigh of the hind limb.

The experimental animals were randomly divided into four groups. Group 1 served as the normal saline control (5 ml/kg B.Wt., I.P.). Group 2 served as the SEC control (1 × 10^6^ cells/mouse). Group 3 served as the compound-treated group (5 mg/kg B.Wt., I.P.). Group 4 received the standard anticancer drug doxorubicin (5 mg/kg BW, I.P.) and is considered as a reference control. Bodyweight and survival were recorded daily until the 24^th^ day in both treated and control groups. At the end of the experiment, the blood of each group was collected under light anaesthesia to the estimate of hematological and biochemical assays. The anaesthetised animals were then sacrificed for evaluation of the antitumor activity and histopathological examination.

##### 4.2.4.2. Antitumor potentiality

It includes tumour volume, weight, and tumour inhibition ratio (TIR%). Time interval measurements of tumour volume using digital Vernier calliper (Tricle Brand, Shanghai, China). Measure tumour length and width using a clipper and then calculate tumour volume using formulations V = (L × W × W)/2, where V is tumour volume, W is tumour width, L is tumour length. While TIR% was calculated according to the following equation

$$\frac{\mathrm{Tumor}\;\mathrm{volume}\;(\mathrm{Control})-\mathrm{Tumor}\;\mathrm{volume}\;(\mathrm{treated})}{\mathrm{Tumor}\;\mathrm{volume}\;(\mathrm{control})}\times \;100.$$

##### 4.2.4.3. Blood assays

At the end of the experiment, animals from different groups were sacrificed, and blood samples were collected for hematological parameters including, Hb, RBC’s, and WBC’s levels, and serum for determination of liver enzymes ALT, AST levels, and proteins. Complete blood count (CBC) was investigated using the Abbott CELL-DYN®1800 automated haematology analyser (USA) using ready-made kits (Abbott Laboratories, Abbott Park, IL, USA). Activities of aspartate aminotransferase (AST) and alanine aminotransferase (ALT) were evaluated using commercial kits (ELITech clinical systems, France). Serum albumin level was determined by kit purchased from STANBIO Company (USA). Protein content was determined by colorimetric method using ready-made kits produced by Instrumentation Laboratory SpA, Inova diagnostics, Milano, Italy.

##### 4.2.4.4. Histopathological study

Specimens of liver-sacrificed mice were fixed in 10% saline formalin. The fixed liver specimens were dehydrated in ascending series of ethyl alcohol and embedded in paraffin. Sections at 5 mm thicknesses were stained with haematoxylin and eosin and examined under the light microscope.

### 4.3. *In silico* studies

#### 4.3.1. Docking study

Discovery Studio 2.5 software was used to perform docking and visualisation according to the described protocol.^16^

#### 4.3.2. *In silico* ADMET analysis

ADMET studies were performed according to the reported procedure as adescribed in Supplementary data^51–53^.

#### 4.3.3. Toxicity studies

Toxicity studies were performed according to the reported procedure as adescribed in Supplementary data^54–56^

## Supplementary Material

Supplemental Material
