# Supplementary material for: Topo II inhibition and DNA intercalation by new phthalazine-based derivatives as potent anticancer agents: design, synthesis, anti-proliferative, docking, and in vivo studies
Source: J Enzyme Inhib Med Chem. 2021 Dec 11;37(1):299–314. doi: 10.1080/14756366.2021.2007905 (PMC8667898; doi:10.1080/14756366.2021.2007905)
Supplement: Supplemental Material [file IENZ_A_2007905_SM7906.pdf]

TOPKAT_Developmental_Toxicity_Potential

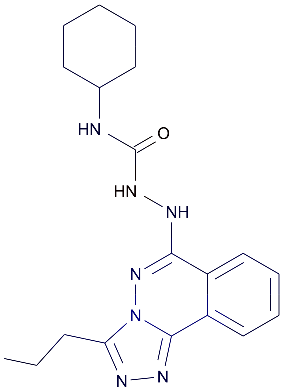

$C_{19}H_{25}N_7O$
Molecular Weight: 367.44809
ALogP: 3.681
Rotatable Bonds: 5
Acceptors: 5
Donors: 3

Model Prediction

Prediction: Non-Toxic
Probability: 0.384
Enrichment: 0.73
Bayesian Score: -4.99
Mahalanobis Distance: 11.9
Mahalanobis Distance p-value: 3.77e-005

Prediction: Positive if the Bayesian score is above the estimated best cutoff value from minimizing the false positive and false negative rate.
Probability: The esimated probability that the sample is in the positive category. This assumes that the Bayesian score follows a normal distribution and is different from the prediction using a cutoff.
Enrichment: An estimate of enrichment, that is, the increased likelihood (versus random) of this sample being in the category.
Bayesian Score: The standard Laplacian-modified Bayesian score.
Mahalanobis Distance: The Mahalanobis distance (MD) is the distance to the center of the training data. The larger the MD, the less trustworthy the prediction.
Mahalanobis Distance p-value: The p-value gives the fraction of training data with an MD greater than or equal to the one for the given sample, assuming normally distributed data. The smaller the p-value, the less trustworthy the prediciton. For highly non-normal X properties (e.g., fingerprints), the MD p-value is wildly inaccurate.

Structural Similar Compounds

Name	Ochratoxin a	Amsacrine	Chenodiol
Structure	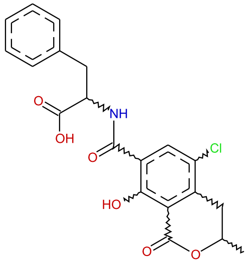	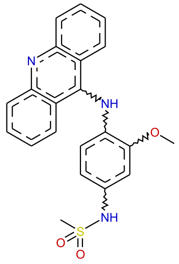	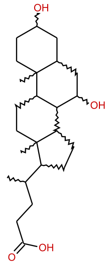
Actual Endpoint	Toxic	Toxic	Toxic
Predicted Endpoint	Toxic	Toxic	Toxic
Distance	0.552	0.588	0.612
Reference	Toxicol Appl Pharmacol 37(2):331-8; 1976	Fundam Appl Toxicol 7(2):214-20; 1986	Arch Int Pharm 246:149-158; 1980

Model Applicability

Unknown features are fingerprint features in the query molecule, but not found or appearing too infrequently in the training set.

1. All properties and OPS components are within expected ranges.

Feature Contribution

Top features for positive contribution				
**Fingerprint**	**Bit/Smiles**	**Feature Structure**	**Score**	**Toxic in training set**
SCFP_6	1256786467	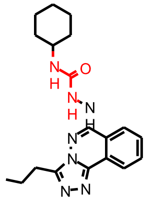 [*]NC(=O)N[*]	0.12	9 out of 15

SCFP_6	9	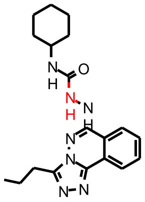 [*]N[*]	0.0928	45 out of 78
SCFP_6	-1272798659	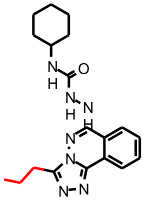 [*]CCC	0.0708	44 out of 78

Top Features for negative contribution

**Fingerprint**	**Bit/Smiles**	**Feature Structure**	**Score**	**Toxic in training set**
SCFP_6	-572674910	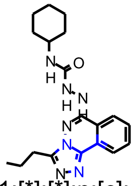 [*]:n1:[*]:[*]:n:[c]:1:[c](:[*]):[*]	-0.945	0 out of 3
SCFP_6	149212520	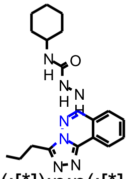 [*][c](:[*]):n:n(:[*]):[*]	-0.448	5 out of 16
SCFP_6	1424144789	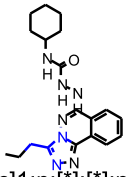 [*]C[c]1:n:[*]:[*]:n:1:[*]	-0.438	1 out of 4

7b TOPKAT_Developmental_Toxicity_Potential

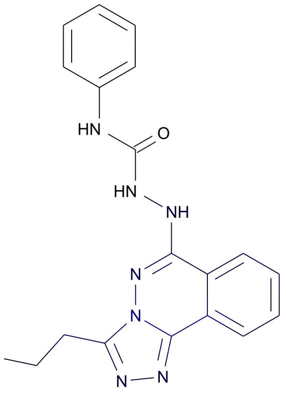

$C_{19}H_{19}N_7O$
Molecular Weight: 361.40046
ALogP: 3.399
Rotatable Bonds: 5
Acceptors: 5
Donors: 3

Model Prediction

Prediction: Non-Toxic
Probability: 0.4
Enrichment: 0.76
Bayesian Score: -4.41
Mahalanobis Distance: 10
Mahalanobis Distance p-value: 0.0194

Prediction: Positive if the Bayesian score is above the estimated best cutoff value from minimizing the false positive and false negative rate.
Probability: The esimated probability that the sample is in the positive category. This assumes that the Bayesian score follows a normal distribution and is different from the prediction using a cutoff.
Enrichment: An estimate of enrichment, that is, the increased likelihood (versus random) of this sample being in the category.
Bayesian Score: The standard Laplacian-modified Bayesian score.
Mahalanobis Distance: The Mahalanobis distance (MD) is the distance to the center of the training data. The larger the MD, the less trustworthy the prediction.
Mahalanobis Distance p-value: The p-value gives the fraction of training data with an MD greater than or equal to the one for the given sample, assuming normally distributed data. The smaller the p-value, the less trustworthy the prediciton. For highly non-normal X properties (e.g., fingerprints), the MD p-value is wildly inaccurate.

Structural Similar Compounds

Name	Ochratoxin a	Amsacrine	Benomyl
Structure	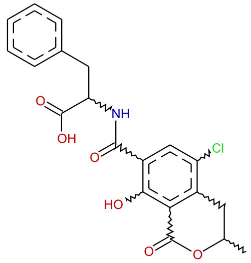	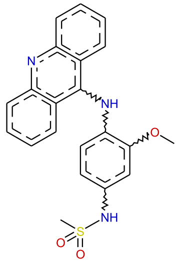	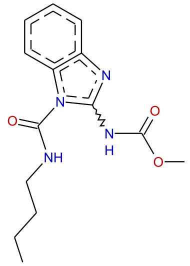
Actual Endpoint	Toxic	Toxic	Toxic
Predicted Endpoint	Toxic	Toxic	Toxic
Distance	0.544	0.587	0.618
Reference	Toxicol Appl Pharmacol 37(2):331-8; 1976	Fundam Appl Toxicol 7(2):214-20; 1986	J Toxicol Environ Health 17:405-417; 1986

Model Applicability

Unknown features are fingerprint features in the query molecule, but not found or appearing too infrequently in the training set.

1. All properties and OPS components are within expected ranges.

Feature Contribution

Top features for positive contribution

Fingerprint	Bit/Smiles	Feature Structure	Score	Toxic in training set
SCFP_6	1631845520	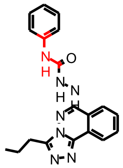 [*]C(=[*])N[c](:[*]):[*]	0.21	8 out of 12

SCFP_6	1205586762	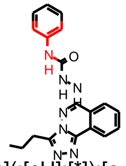 [*]N[c](:[cH]:[*]):[cH]:[*]	0.139	11 out of 18
SCFP_6	1256786467	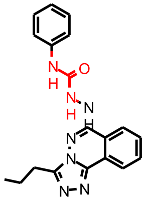 [*]NC(=O)N[*]	0.12	9 out of 15
**Top Features for negative contribution**				
**Fingerprint**	**Bit/Smiles**	**Feature Structure**	**Score**	**Toxic in training set**
SCFP_6	-572674910	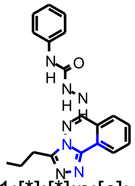 [*]:n1:[*]:[*]:n:[c]:1:[c](:[*]):[*]	-0.945	0 out of 3
SCFP_6	149212520	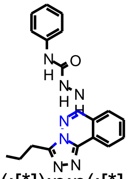 [*][c](:[*]):n:n(:[*]):[*]	-0.448	5 out of 16
SCFP_6	1424144789	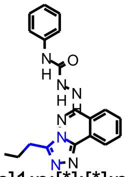 [*]C[c]1:n:[*]:[*]:n:1:[*]	-0.438	1 out of 4

TOPKAT_Developmental_Toxicity_Potential

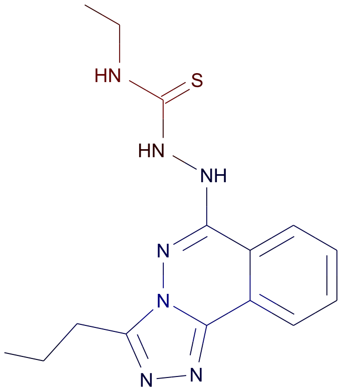

$C_{15}H_{19}N_7S$
Molecular Weight: 329.42325
ALogP: 3.388
Rotatable Bonds: 7
Acceptors: 5
Donors: 3

Model Prediction

Prediction: Non-Toxic
Probability: 0.451
Enrichment: 0.857
Bayesian Score: -2.74
Mahalanobis Distance: 9.32
Mahalanobis Distance p-value: 0.0982

Prediction: Positive if the Bayesian score is above the estimated best cutoff value from minimizing the false positive and false negative rate.
Probability: The esimated probability that the sample is in the positive category. This assumes that the Bayesian score follows a normal distribution and is different from the prediction using a cutoff.
Enrichment: An estimate of enrichment, that is, the increased likelihood (versus random) of this sample being in the category.
Bayesian Score: The standard Laplacian-modified Bayesian score.
Mahalanobis Distance: The Mahalanobis distance (MD) is the distance to the center of the training data. The larger the MD, the less trustworthy the prediction.
Mahalanobis Distance p-value: The p-value gives the fraction of training data with an MD greater than or equal to the one for the given sample, assuming normally distributed data. The smaller the p-value, the less trustworthy the prediciton. For highly non-normal X properties (e.g., fingerprints), the MD p-value is wildly inaccurate.

Structural Similar Compounds

Name	Ochratoxin a	LY171883	Benomyl
Structure	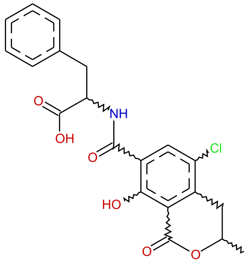	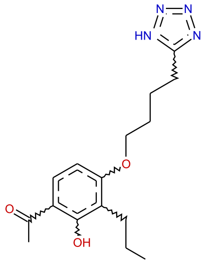	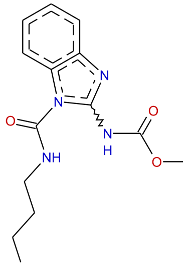
Actual Endpoint	Toxic	Non-Toxic	Toxic
Predicted Endpoint	Toxic	Non-Toxic	Toxic
Distance	0.641	0.645	0.671
Reference	Toxicol Appl Pharmacol 37(2):331-8; 1976	Fundam Appl Toxicol 10(4):672-81; 1988	J Toxicol Environ Health 17:405-417; 1986

Model Applicability

Unknown features are fingerprint features in the query molecule, but not found or appearing too infrequently in the training set.

1. All properties and OPS components are within expected ranges.

Feature Contribution

Top features for positive contribution

Fingerprint	Bit/Smiles	Feature Structure	Score	Toxic in training set
SCFP_6	18058322	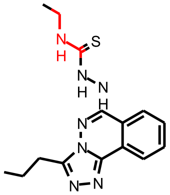 [*]CNC(=[*])[*]	0.478	4 out of 4

SCFP_6	1435188938	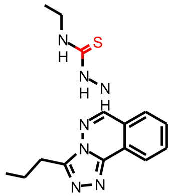 [*]C(=S)[*]	0.478	4 out of 4
SCFP_6	382734644	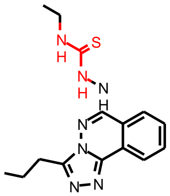 [*]NC(=S)N[*]	0.478	4 out of 4

Top Features for negative contribution

Fingerprint	Bit/Smiles	Feature Structure	Score	Toxic in training set
SCFP_6	-572674910	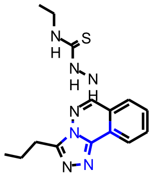 [*]:n1:[*]:[*]:n:[c]:1:[c](:[*]):[*]	-0.945	0 out of 3
SCFP_6	149212520	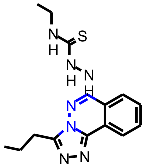 [*][c](:[*]):n:n(:[*]):[*]	-0.448	5 out of 16
SCFP_6	1424144789	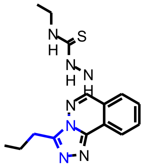 [*]C[c]1:n:[*]:[*]:n:1:[*]	-0.438	1 out of 4

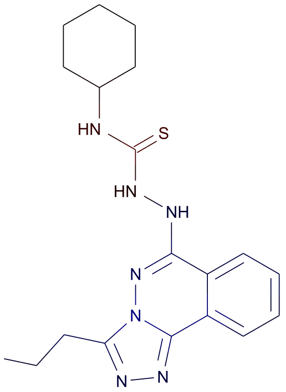

$C_{19}H_{25}N_7S$
Molecular Weight: 383.51369
ALogP: 4.898
Rotatable Bonds: 7
Acceptors: 5
Donors: 3

Structural Similar Compounds

Name	Ochratoxin a	Amsacrine	Dobutamine .HCl (Free base form)
Structure	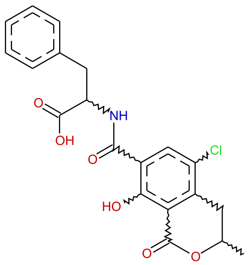	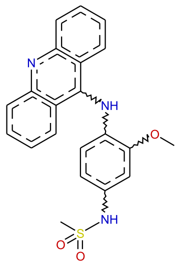	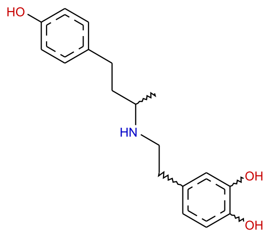
Actual Endpoint	Toxic	Toxic	Toxic
Predicted Endpoint	Toxic	Toxic	Toxic
Distance	0.671	0.674	0.698
Reference	Toxicol Appl Pharmacol 37(2):331-8; 1976	Fundam Appl Toxicol 7(2):214-20; 1986	Yakuri to Chiryo 7:1707-1730; 1979

Model Prediction

Prediction: Non-Toxic
Probability: 0.46
Enrichment: 0.874
Bayesian Score: -2.47
Mahalanobis Distance: 11
Mahalanobis Distance p-value: 0.000957

Prediction: Positive if the Bayesian score is above the estimated best cutoff value from minimizing the false positive and false negative rate.
Probability: The esimated probability that the sample is in the positive category. This assumes that the Bayesian score follows a normal distribution and is different from the prediction using a cutoff.
Enrichment: An estimate of enrichment, that is, the increased likelihood (versus random) of this sample being in the category.
Bayesian Score: The standard Laplacian-modified Bayesian score.
Mahalanobis Distance: The Mahalanobis distance (MD) is the distance to the center of the training data. The larger the MD, the less trustworthy the prediction.
Mahalanobis Distance p-value: The p-value gives the fraction of training data with an MD greater than or equal to the one for the given sample, assuming normally distributed data. The smaller the p-value, the less trustworthy the prediciton. For highly non-normal X properties (e.g., fingerprints), the MD p-value is wildly inaccurate.

Model Applicability

Unknown features are fingerprint features in the query molecule, but not found or appearing too infrequently in the training set.

1. All properties and OPS components are within expected ranges.

Feature Contribution

Top features for positive contribution

Fingerprint	Bit/Smiles	Feature Structure	Score	Toxic in training set
SCFP_6	1435188938	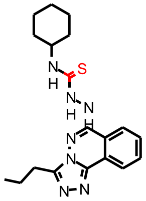 [*]C(=S)[*]	0.478	4 out of 4

SCFP_6	18058322	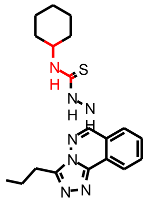 [*]CNC(=[*])[*]	0.478	4 out of 4
SCFP_6	382734644	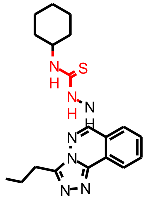 [*]NC(=S)N[*]	0.478	4 out of 4

Top Features for negative contribution

Fingerprint	Bit/Smiles	Feature Structure	Score	Toxic in training set
SCFP_6	-572674910	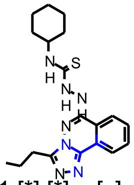 [*]:n1:[*]:[*]:n:[c]:1:[c](:[*]):[*]	-0.945	0 out of 3
SCFP_6	149212520	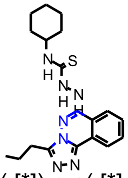 [*][c](:[*]):n:n(:[*]):[*]	-0.448	5 out of 16
SCFP_6	1424144789	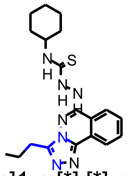 [*]C[c]1:n:[*]:[*]:n:1:[*]	-0.438	1 out of 4

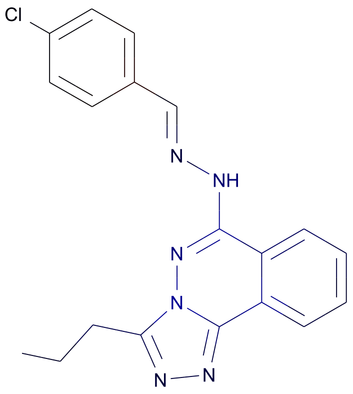

$C_{19}H_{17}ClN_6$
Molecular Weight: 364.83147
ALogP: 4.967
Rotatable Bonds: 5
Acceptors: 5
Donors: 1

Model Prediction

Prediction: Non-Toxic
Probability: 0.371
Enrichment: 0.706
Bayesian Score: -5.47
Mahalanobis Distance: 9.86
Mahalanobis Distance p-value: 0.0301

Prediction: Positive if the Bayesian score is above the estimated best cutoff value from minimizing the false positive and false negative rate.
Probability: The esimated probability that the sample is in the positive category. This assumes that the Bayesian score follows a normal distribution and is different from the prediction using a cutoff.
Enrichment: An estimate of enrichment, that is, the increased likelihood (versus random) of this sample being in the category.
Bayesian Score: The standard Laplacian-modified Bayesian score.
Mahalanobis Distance: The Mahalanobis distance (MD) is the distance to the center of the training data. The larger the MD, the less trustworthy the prediction.
Mahalanobis Distance p-value: The p-value gives the fraction of training data with an MD greater than or equal to the one for the given sample, assuming normally distributed data. The smaller the p-value, the less trustworthy the prediciton. For highly non-normal X properties (e.g., fingerprints), the MD p-value is wildly inaccurate.

Structural Similar Compounds

Name	Perphenazine	Acemetacin	Amsacrine
Structure	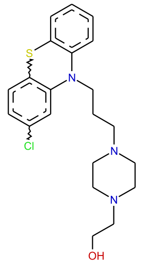	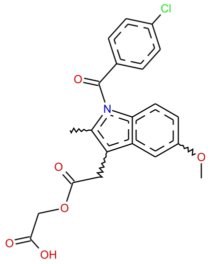	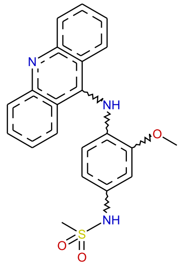
Actual Endpoint	Toxic	Non-Toxic	Toxic
Predicted Endpoint	Toxic	Non-Toxic	Toxic
Distance	0.579	0.618	0.640
Reference	Toxicol Appl Pharmacol 21(2):230-6; 1972	Oyo Yakuri 22(6):777-786; 1981	Fundam Appl Toxicol 7(2):214-20; 1986

Model Applicability

Unknown features are fingerprint features in the query molecule, but not found or appearing too infrequently in the training set.

1. All properties and OPS components are within expected ranges.

Feature Contribution

Top features for positive contribution

Fingerprint	Bit/Smiles	Feature Structure	Score	Toxic in training set
SCFP_6	1390366442	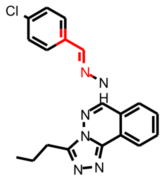 [*]\N=C\[c](:[*]):[*]	0.381	2 out of 2

SCFP_6	-650738059	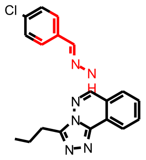 [*]N\N=C\[c](:[cH]:[*]):[cH]:[*]	0.271	1 out of 1
SCFP_6	1905487031	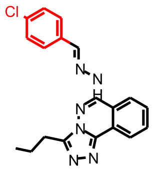 [*][c]1:[cH]:[cH]:[c] (Cl):[cH]:[cH]:1	0.153	2 out of 3

Top Features for negative contribution

Fingerprint	Bit/Smiles	Feature Structure	Score	Toxic in training set
SCFP_6	-572674910	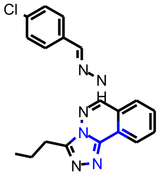 [*]:n1:[*]:[*]:n:[c]: 1:[c](:[*]):[*]	-0.945	0 out of 3
SCFP_6	149212520	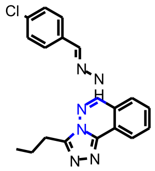 [*][c](:[*]):n:n(:[*]):[*]	-0.448	5 out of 16
SCFP_6	1424144789	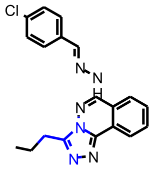 [*]C[c]1:n:[*]:[*]:n: 1:[*]	-0.438	1 out of 4

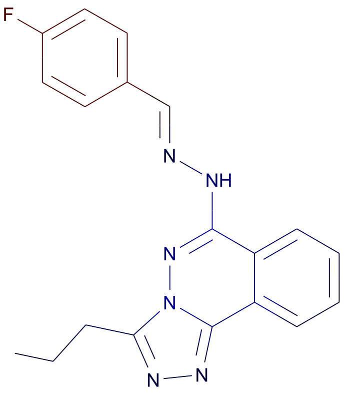

$C_{19}H_{17}FN_6$
Molecular Weight: 348.37688
ALogP: 4.508
Rotatable Bonds: 5
Acceptors: 5
Donors: 1

Model Prediction

Prediction: Non-Toxic
Probability: 0.393
Enrichment: 0.748
Bayesian Score: -4.65
Mahalanobis Distance: 9.95
Mahalanobis Distance p-value: 0.0235

Prediction: Positive if the Bayesian score is above the estimated best cutoff value from minimizing the false positive and false negative rate.
Probability: The esimated probability that the sample is in the positive category. This assumes that the Bayesian score follows a normal distribution and is different from the prediction using a cutoff.
Enrichment: An estimate of enrichment, that is, the increased likelihood (versus random) of this sample being in the category.
Bayesian Score: The standard Laplacian-modified Bayesian score.
Mahalanobis Distance: The Mahalanobis distance (MD) is the distance to the center of the training data. The larger the MD, the less trustworthy the prediction.
Mahalanobis Distance p-value: The p-value gives the fraction of training data with an MD greater than or equal to the one for the given sample, assuming normally distributed data. The smaller the p-value, the less trustworthy the prediciton. For highly non-normal X properties (e.g., fingerprints), the MD p-value is wildly inaccurate.

Structural Similar Compounds

Name	Perphenazine	Amsacrine	Budralazine
Structure	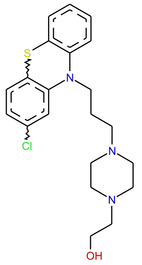	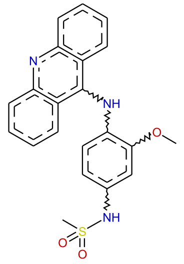	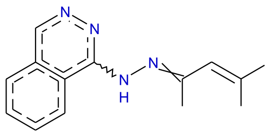
Actual Endpoint	Toxic	Toxic	Non-Toxic
Predicted Endpoint	Toxic	Toxic	Non-Toxic
Distance	0.601	0.622	0.623
Reference	Toxicol Appl Pharmacol 21(2):230-6; 1972	Fundam Appl Toxicol 7(2):214-20; 1986	Oyo Yakuri 21(2):321-330; 1981

Model Applicability

Unknown features are fingerprint features in the query molecule, but not found or appearing too infrequently in the training set.

1. All properties and OPS components are within expected ranges.

Feature Contribution

Top features for positive contribution

Fingerprint	Bit/Smiles	Feature Structure	Score	Toxic in training set
SCFP_6	-1789136446	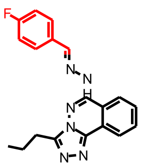 [*]=C[c]1:[cH]:[cH]:[c](F):[cH]:[cH]:1	0.381	2 out of 2

SCFP_6	1390366442	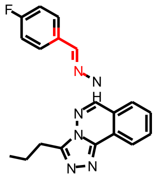 [*]\N=C\[c](:[*]):[*]	0.381	2 out of 2
SCFP_6	-783770208	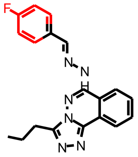 F[c]1:[cH]:[cH]:[*]:[cH]:[cH]:1	0.322	4 out of 5

Top Features for negative contribution

Fingerprint	Bit/Smiles	Feature Structure	Score	Toxic in training set
SCFP_6	-572674910	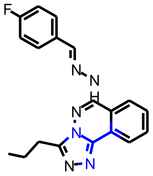 [*]:n1:[*]:[*]:n:[c]:1:[c](:[*]):[*]	-0.945	0 out of 3
SCFP_6	149212520	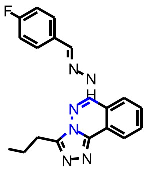 [*][c](:[*]):n:n(:[*]):[*]	-0.448	5 out of 16
SCFP_6	1424144789	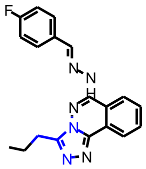 [*]C[c]1:n:[*]:[*]:n:1:[*]	-0.438	1 out of 4

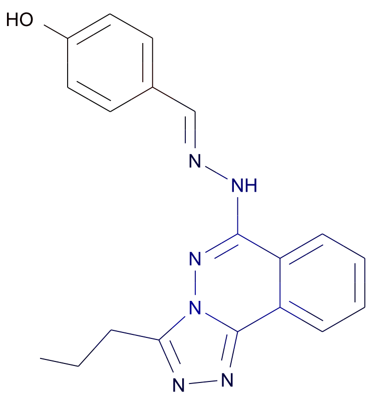

$C_{19}H_{18}N_6O$
Molecular Weight: 346.38582
ALogP: 4.061
Rotatable Bonds: 5
Acceptors: 6
Donors: 2

Model Prediction

Prediction: Non-Toxic
Probability: 0.367
Enrichment: 0.698
Bayesian Score: -5.65
Mahalanobis Distance: 10.2
Mahalanobis Distance p-value: 0.0115

Prediction: Positive if the Bayesian score is above the estimated best cutoff value from minimizing the false positive and false negative rate.
Probability: The esimated probability that the sample is in the positive category. This assumes that the Bayesian score follows a normal distribution and is different from the prediction using a cutoff.
Enrichment: An estimate of enrichment, that is, the increased likelihood (versus random) of this sample being in the category.
Bayesian Score: The standard Laplacian-modified Bayesian score.
Mahalanobis Distance: The Mahalanobis distance (MD) is the distance to the center of the training data. The larger the MD, the less trustworthy the prediction.
Mahalanobis Distance p-value: The p-value gives the fraction of training data with an MD greater than or equal to the one for the given sample, assuming normally distributed data. The smaller the p-value, the less trustworthy the prediciton. For highly non-normal X properties (e.g., fingerprints), the MD p-value is wildly inaccurate.

Structural Similar Compounds

Name	Amsacrine	Ochratoxin a	Benomyl
Structure	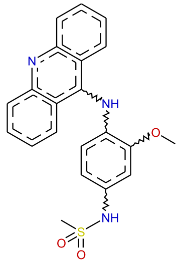	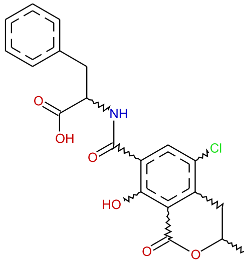	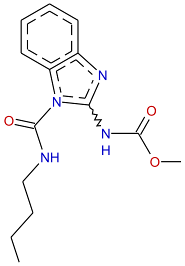
Actual Endpoint	Toxic	Toxic	Toxic
Predicted Endpoint	Toxic	Toxic	Toxic
Distance	0.546	0.625	0.640
Reference	Fundam Appl Toxicol 7(2):214-20; 1986	Toxicol Appl Pharmacol 37(2):331-8; 1976	J Toxicol Environ Health 17:405-417; 1986

Model Applicability

Unknown features are fingerprint features in the query molecule, but not found or appearing too infrequently in the training set.

1. All properties and OPS components are within expected ranges.

Feature Contribution

Top features for positive contribution

Fingerprint	Bit/Smiles	Feature Structure	Score	Toxic in training set
SCFP_6	1390366442	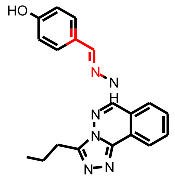 [*]\N=C\[c](:[*]):[*]	0.381	2 out of 2

SCFP_6	-650738059	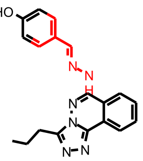 [*]N\N=C\[c](:[cH]:[*]):[cH]:[*]	0.271	1 out of 1
SCFP_6	-1374800107	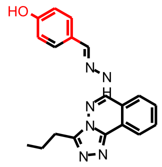 [*][c]1:[*]:[cH]:[c](O):[cH]:[cH]:1	0.205	26 out of 40

Top Features for negative contribution

Fingerprint	Bit/Smiles	Feature Structure	Score	Toxic in training set
SCFP_6	-572674910	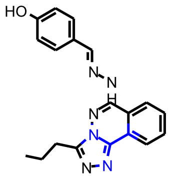 [*]:n1:[*]:[*]:n:[c]:1:[c](:[*]):[*]	-0.945	0 out of 3
SCFP_6	149212520	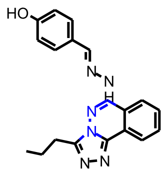 [*][c](:[*]):n:n(:[*]):[*]	-0.448	5 out of 16
SCFP_6	1424144789	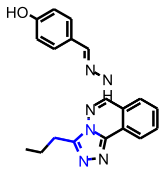 [*]C[c]1:n:[*]:[*]:n:1:[*]	-0.438	1 out of 4

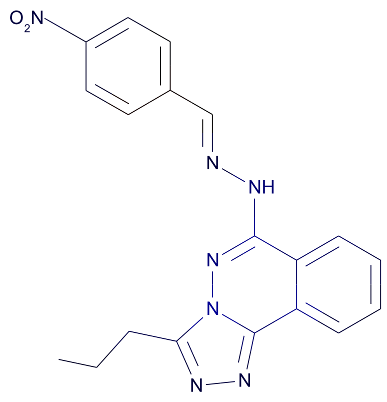

$C_{19}H_{17}N_7O_2$
Molecular Weight: 375.38397
ALogP: 4.197
Rotatable Bonds: 6
Acceptors: 7
Donors: 1

Model Prediction

Prediction: Non-Toxic
Probability: 0.331
Enrichment: 0.629
Bayesian Score: -7.19
Mahalanobis Distance: 10.1
Mahalanobis Distance p-value: 0.0164

Prediction: Positive if the Bayesian score is above the estimated best cutoff value from minimizing the false positive and false negative rate.
Probability: The esimated probability that the sample is in the positive category. This assumes that the Bayesian score follows a normal distribution and is different from the prediction using a cutoff.
Enrichment: An estimate of enrichment, that is, the increased likelihood (versus random) of this sample being in the category.
Bayesian Score: The standard Laplacian-modified Bayesian score.
Mahalanobis Distance: The Mahalanobis distance (MD) is the distance to the center of the training data. The larger the MD, the less trustworthy the prediction.
Mahalanobis Distance p-value: The p-value gives the fraction of training data with an MD greater than or equal to the one for the given sample, assuming normally distributed data. The smaller the p-value, the less trustworthy the prediciton. For highly non-normal X properties (e.g., fingerprints), the MD p-value is wildly inaccurate.

Structural Similar Compounds

Name	Acemetacin	Amsacrine	Bunazosin .HCl (Free base form)
Structure	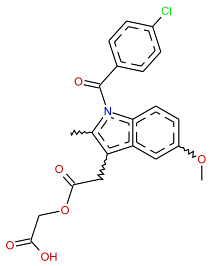	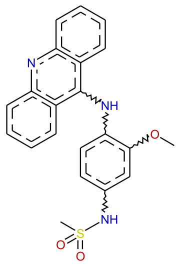	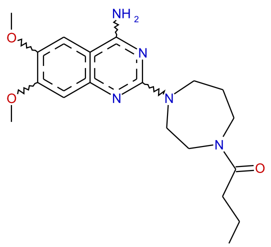
Actual Endpoint	Non-Toxic	Toxic	Toxic
Predicted Endpoint	Non-Toxic	Toxic	Toxic
Distance	0.608	0.665	0.671
Reference	Oyo Yakuri 22(6):777-786; 1981	Fundam Appl Toxicol 7(2):214-20; 1986	Kiso to Rinsho 17:914-924; 1983

Model Applicability

Unknown features are fingerprint features in the query molecule, but not found or appearing too infrequently in the training set.

1. All properties and OPS components are within expected ranges.

Feature Contribution

Top features for positive contribution				
**Fingerprint**	**Bit/Smiles**	**Feature Structure**	**Score**	**Toxic in training set**
SCFP_6	1390366442	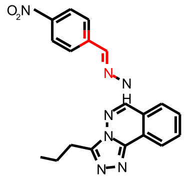 [*]\N=C\[c](:[*]):[*]	0.381	2 out of 2

SCFP_6	-650738059	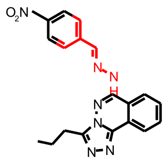 [*]N\N=C\[c](:[cH]:[*]):[cH]:[*]	0.271	1 out of 1
SCFP_6	-2056718782	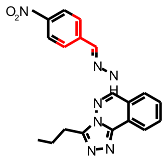 [*]=C[c](:[cH]:[*]):[cH]:[*]	0.0786	33 out of 58

Top Features for negative contribution

**Fingerprint**	**Bit/Smiles**	**Feature Structure**	**Score**	**Toxic in training set**
SCFP_6	-572674910	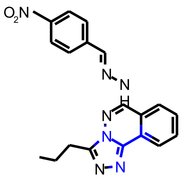 [*]:n1:[*]:[*]:n:[c]:1:[c](:[*]):[*]	-0.945	0 out of 3
SCFP_6	-1380909229	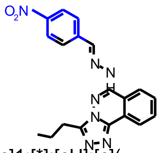 [*][c]1:[*]:[cH]:[c](:[cH]:[cH]:1)[N+](=[*])[*]	-0.449	6 out of 19
SCFP_6	149212520	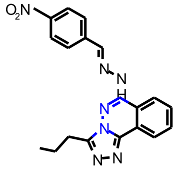 [*][c](:[*]):n:n(:[*]):[*]	-0.448	5 out of 16

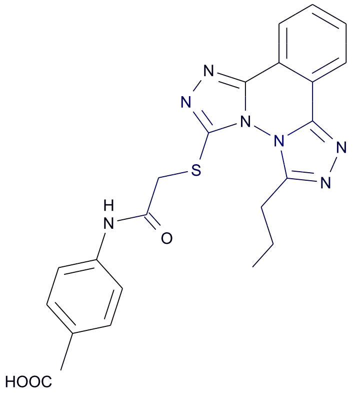

$C_{22}H_{19}N_{7}O_{3}S$
Molecular Weight: 461.49635
ALogP: 3.42
Rotatable Bonds: 7
Acceptors: 8
Donors: 2

Model Prediction

Prediction: Non-Toxic
Probability: 0.368
Enrichment: 0.699
Bayesian Score: -5.62
Mahalanobis Distance: 10.7
Mahalanobis Distance p-value: 0.00292

Prediction: Positive if the Bayesian score is above the estimated best cutoff value from minimizing the false positive and false negative rate.
Probability: The esimated probability that the sample is in the positive category. This assumes that the Bayesian score follows a normal distribution and is different from the prediction using a cutoff.
Enrichment: An estimate of enrichment, that is, the increased likelihood (versus random) of this sample being in the category.
Bayesian Score: The standard Laplacian-modified Bayesian score.
Mahalanobis Distance: The Mahalanobis distance (MD) is the distance to the center of the training data. The larger the MD, the less trustworthy the prediction.
Mahalanobis Distance p-value: The p-value gives the fraction of training data with an MD greater than or equal to the one for the given sample, assuming normally distributed data. The smaller the p-value, the less trustworthy the prediciton. For highly non-normal X properties (e.g., fingerprints), the MD p-value is wildly inaccurate.

Structural Similar Compounds

Name	Ochratoxin a	LY171883	Lenampicillin .HCl (Free base form)
Structure	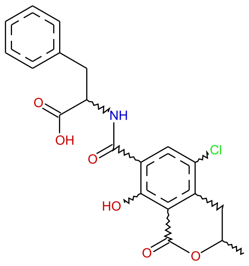	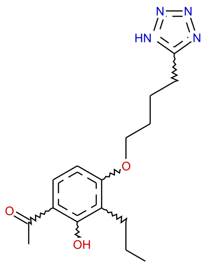	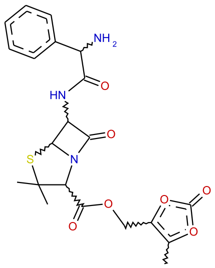
Actual Endpoint	Toxic	Non-Toxic	Non-Toxic
Predicted Endpoint	Toxic	Non-Toxic	Non-Toxic
Distance	0.682	0.698	0.703
Reference	Toxicol Appl Pharmacol 37(2):331-8; 1976	Fundam Appl Toxicol 10(4):672-81; 1988	Chemotherapy 32:130-145; 1984

Model Applicability

Unknown features are fingerprint features in the query molecule, but not found or appearing too infrequently in the training set.

1. All properties and OPS components are within expected ranges.

Feature Contribution

Top features for positive contribution

Fingerprint	Bit/Smiles	Feature Structure	Score	Toxic in training set
SCFP_6	1623050981	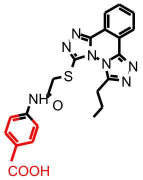 OC(=O)[c]1:[cH]:[cH]:[*]:[cH]:[cH]:1	0.271	1 out of 1

SCFP_6	1631845520	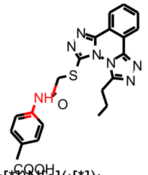 [*]C(=[*])N[c](:[*]):[*]	0.21	8 out of 12
SCFP_6	1205586762	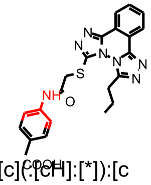 [*]N[c](:[cH]:[*]):[cH]:[*]	0.139	11 out of 18

Top Features for negative contribution

Fingerprint	Bit/Smiles	Feature Structure	Score	Toxic in training set
SCFP_6	-572674910	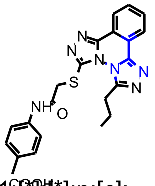 [*]:n1:[*]:[*]:n:[c]:1:[c](:[*]):[*]	-0.945	0 out of 3
SCFP_6	-299471172	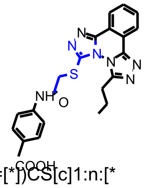 [*]C(=[*])CS[c]1:n:[*]:[*]:n:1:[*]	-0.594	1 out of 5
SCFP_6	149212520	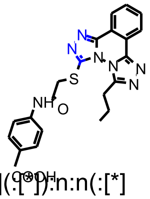 [*][c](:[*]):n:n(:[*]):[*]	-0.448	5 out of 16

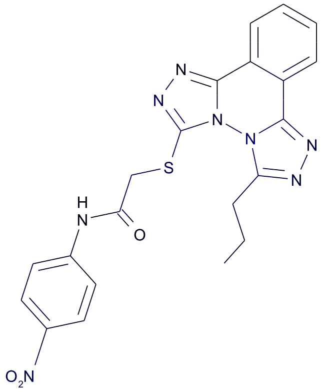

$C_{21}H_{18}N_8O_3S$
Molecular Weight: 462.48441
ALogP: 3.685
Rotatable Bonds: 7
Acceptors: 8
Donors: 1

Model Prediction

Prediction: Non-Toxic
Probability: 0.344
Enrichment: 0.654
Bayesian Score: -6.6
Mahalanobis Distance: 11.1
Mahalanobis Distance p-value: 0.000823

Prediction: Positive if the Bayesian score is above the estimated best cutoff value from minimizing the false positive and false negative rate.
Probability: The esimated probability that the sample is in the positive category. This assumes that the Bayesian score follows a normal distribution and is different from the prediction using a cutoff.
Enrichment: An estimate of enrichment, that is, the increased likelihood (versus random) of this sample being in the category.
Bayesian Score: The standard Laplacian-modified Bayesian score.
Mahalanobis Distance: The Mahalanobis distance (MD) is the distance to the center of the training data. The larger the MD, the less trustworthy the prediction.
Mahalanobis Distance p-value: The p-value gives the fraction of training data with an MD greater than or equal to the one for the given sample, assuming normally distributed data. The smaller the p-value, the less trustworthy the prediciton. For highly non-normal X properties (e.g., fingerprints), the MD p-value is wildly inaccurate.

Structural Similar Compounds

Name	Acemetacin	Beclomethasone Dipropionate	Hydrocortisone-17-butyrate-21-propionate
Structure	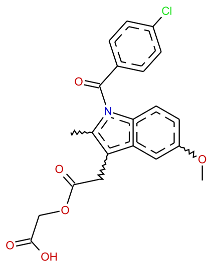	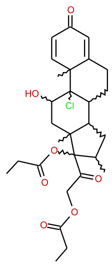	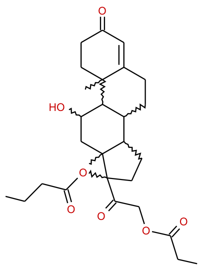
Actual Endpoint	Non-Toxic	Toxic	Toxic
Predicted Endpoint	Non-Toxic	Toxic	Toxic
Distance	0.701	0.730	0.731
Reference	Oyo Yakuri 22(6):777-786; 1981	Oyo Yakuri 18(6):1021-1038; 1979	Oyo Yakuri 21:441-466; 1981

Model Applicability

Unknown features are fingerprint features in the query molecule, but not found or appearing too infrequently in the training set.

1. All properties and OPS components are within expected ranges.

Feature Contribution

Top features for positive contribution

Fingerprint	Bit/Smiles	Feature Structure	Score	Toxic in training set
SCFP_6	1631845520	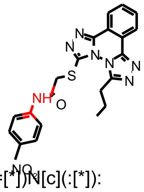 [*]C(=[*])N[c](:[*]):[*]	0.21	8 out of 12

SCFP_6	1205586762	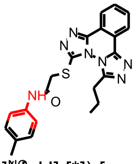 [*]N[c](:[cH]:[*]):[c H]:[*]	0.139	11 out of 18
SCFP_6	9	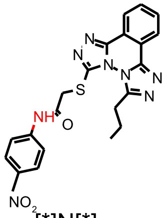 [*]N[*]	0.0928	45 out of 78
**Top Features for negative contribution**				
**Fingerprint**	**Bit/Smiles**	**Feature Structure**	**Score**	**Toxic in training set**
SCFP_6	-572674910	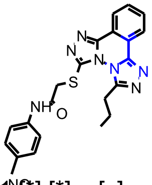 [*]:n1:[*]:[*]:n:[c]: 1:[c](:[*]):[*]	-0.945	0 out of 3
SCFP_6	-299471172	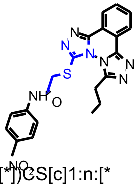 [*]C(=[*])CS[c]1:n:[*]:[*]:n:1:[*]	-0.594	1 out of 5
SCFP_6	-1380909229	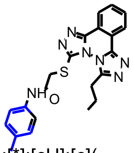 [*][c]1:[*]:[cH]:[c](:[cH]:[cH]:1)[N+](=[*])[*]	-0.449	6 out of 19

TOPKAT_Developmental_Toxicity_Potential

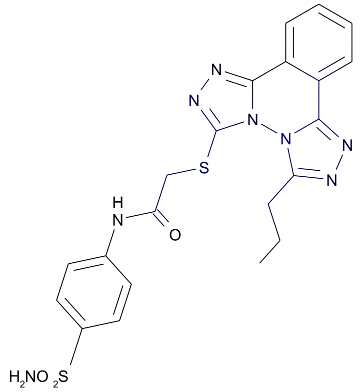

$C_{21}H_{20}N_8O_3S_2$
Molecular Weight: 496.56529
ALogP: 2.496
Rotatable Bonds: 7
Acceptors: 8
Donors: 2

Model Prediction

Prediction: Non-Toxic
Probability: 0.367
Enrichment: 0.698
Bayesian Score: -5.64
Mahalanobis Distance: 9.96
Mahalanobis Distance p-value: 0.0234

Prediction: Positive if the Bayesian score is above the estimated best cutoff value from minimizing the false positive and false negative rate.
Probability: The esimated probability that the sample is in the positive category. This assumes that the Bayesian score follows a normal distribution and is different from the prediction using a cutoff.
Enrichment: An estimate of enrichment, that is, the increased likelihood (versus random) of this sample being in the category.
Bayesian Score: The standard Laplacian-modified Bayesian score.
Mahalanobis Distance: The Mahalanobis distance (MD) is the distance to the center of the training data. The larger the MD, the less trustworthy the prediction.
Mahalanobis Distance p-value: The p-value gives the fraction of training data with an MD greater than or equal to the one for the given sample, assuming normally distributed data. The smaller the p-value, the less trustworthy the prediciton. For highly non-normal X properties (e.g., fingerprints), the MD p-value is wildly inaccurate.

Structural Similar Compounds

Name	Lenampicillin .HCl (Free base form)	Bacampicillin .HCl (Free base form)	Cyclic AMP Bucladesine
Structure	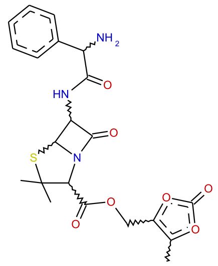	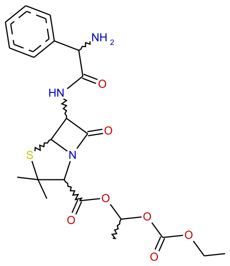	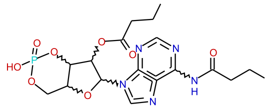
Actual Endpoint	Non-Toxic	Toxic	Non-Toxic
Predicted Endpoint	Non-Toxic	Non-Toxic	Non-Toxic
Distance	0.640	0.686	0.711
Reference	Chemotherapy 32:130-145; 1984	Chemotherapy 27:30-35; 1979	Oyo Yakuri 27(3):585-597; 1984

Model Applicability

Unknown features are fingerprint features in the query molecule, but not found or appearing too infrequently in the training set.

1. All properties and OPS components are within expected ranges.

Feature Contribution

Top features for positive contribution

Fingerprint	Bit/Smiles	Feature Structure	Score	Toxic in training set
SCFP_6	-1247518081	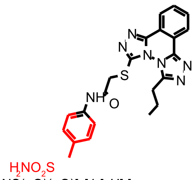 NS(=O)(=O)[c]1:[cH]:[cH]:[*]:[cH]:[cH]:1	0.271	1 out of 1

SCFP_6	-1380395165	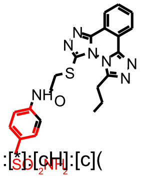<br>[*][c]1:[*]:[cH]:[c](:[cH]:[cH]:1)S(=[*])(=[*])[*]	0.255	3 out of 4
SCFP_6	1631845520	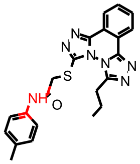<br>[*]C(=[*])N[c](:[*]):[*]	0.21	8 out of 12
**Top Features for negative contribution**				
**Fingerprint**	**Bit/Smiles**	**Feature Structure**	**Score**	**Toxic in training set**
SCFP_6	-572674910	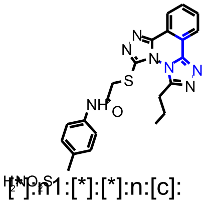<br>[*]:n1:[*]:[*]:n:[c]:1:[c](:[*]):[*]	-0.945	0 out of 3
SCFP_6	-1463646519	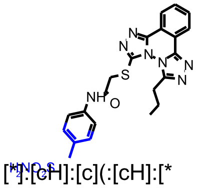<br>[*]:[cH]:[c](:[cH]:[*])S(=O)(=O)N	-0.718	0 out of 2
SCFP_6	-299471172	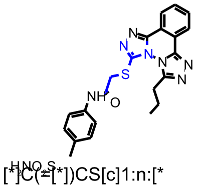<br>[*]C(=[*])CS[c]1:n:[*]:[*]:n:1:[*]	-0.594	1 out of 5

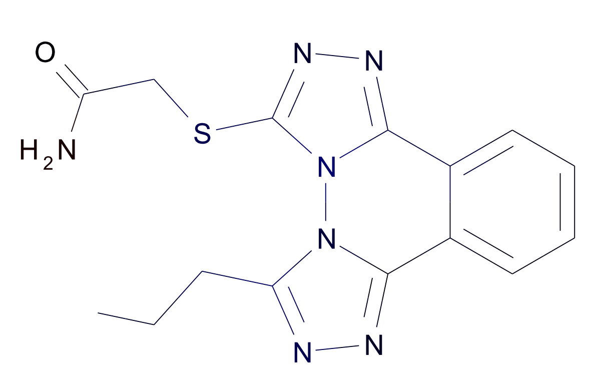

$C_{15}H_{15}N_7OS$
Molecular Weight: 341.39089
ALogP: 2.008
Rotatable Bonds: 5
Acceptors: 6
Donors: 1

Structural Similar Compounds

Name	Guthion	Prazosin .HCl (Free base form)	Dinoseb
Structure	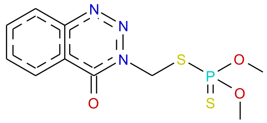	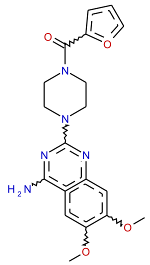	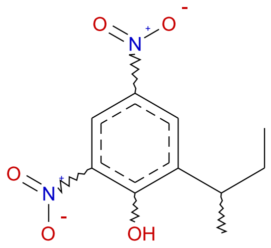
Actual Endpoint	Non-Toxic	Toxic	Toxic
Predicted Endpoint	Non-Toxic	Toxic	Toxic
Distance	0.630	0.653	0.657
Reference	Arch Toxicol 43:177-186; 1980	Oyo Yakuri 17:57-62; 1979	Arch Environ Contam Toxicol 15(4):377-84; 1986

Model Prediction

Prediction: Non-Toxic
Probability: 0.382
Enrichment: 0.725
Bayesian Score: -5.08
Mahalanobis Distance: 9.46
Mahalanobis Distance p-value: 0.0743

Prediction: Positive if the Bayesian score is above the estimated best cutoff value from minimizing the false positive and false negative rate.
Probability: The esimated probability that the sample is in the positive category. This assumes that the Bayesian score follows a normal distribution and is different from the prediction using a cutoff.
Enrichment: An estimate of enrichment, that is, the increased likelihood (versus random) of this sample being in the category.
Bayesian Score: The standard Laplacian-modified Bayesian score.
Mahalanobis Distance: The Mahalanobis distance (MD) is the distance to the center of the training data. The larger the MD, the less trustworthy the prediction.
Mahalanobis Distance p-value: The p-value gives the fraction of training data with an MD greater than or equal to the one for the given sample, assuming normally distributed data. The smaller the p-value, the less trustworthy the prediciton. For highly non-normal X properties (e.g., fingerprints), the MD p-value is wildly inaccurate.

Model Applicability

Unknown features are fingerprint features in the query molecule, but not found or appearing too infrequently in the training set.

1. All properties and OPS components are within expected ranges.

Feature Contribution

Top features for positive contribution

Fingerprint	Bit/Smiles	Feature Structure	Score	Toxic in training set
SCFP_6	-1357949052	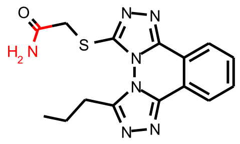 [*]C(=[*])N	0.453	8 out of 9

SCFP_6	9	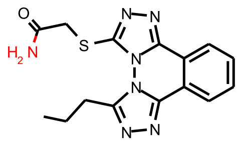 [*]N[*]	0.0928	45 out of 78
SCFP_6	-1272798659	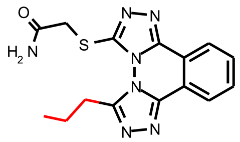 [*]CCC	0.0708	44 out of 78

Top Features for negative contribution

Fingerprint	Bit/Smiles	Feature Structure	Score	Toxic in training set
SCFP_6	-572674910	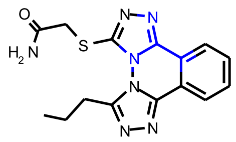 [*]:n1:[*]:[*]:n:[c]:1:[c](:[*]):[*]	-0.945	0 out of 3
SCFP_6	-299471172	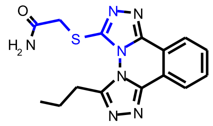 [*]C(=[*])CS[c]1:n:[*]:[*]:n:1:[*]	-0.594	1 out of 5
SCFP_6	149212520	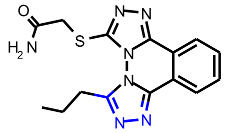 [*][c](:[*]):n:n(:[*]):[*]	-0.448	5 out of 16

Doxorubicin

TOPKAT_Developmental_Toxicity_Potential

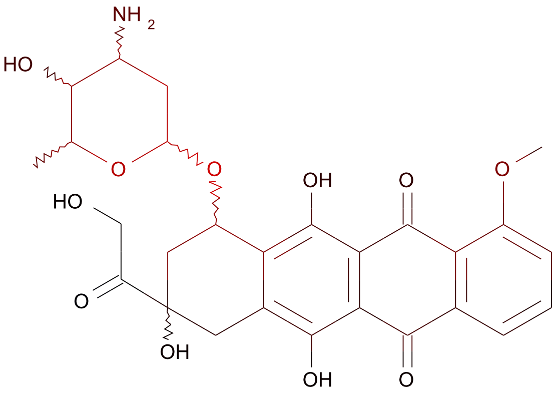

$C_{27}H_{29}NO_{11}$
Molecular Weight: 543.51925
ALogP: -4.4e-002
Rotatable Bonds: 5
Acceptors: 12
Donors: 6

Model Prediction

Prediction: Toxic

Probability: 0.882

Enrichment: 1.68

Bayesian Score: 9.61

Mahalanobis Distance: 11.3

Mahalanobis Distance p-value: 0.000349

Prediction: Positive if the Bayesian score is above the estimated best cutoff value from minimizing the false positive and false negative rate.
Probability: The esimated probability that the sample is in the positive category. This assumes that the Bayesian score follows a normal distribution and is different from the prediction using a cutoff.
Enrichment: An estimate of enrichment, that is, the increased likelihood (versus random) of this sample being in the category.
Bayesian Score: The standard Laplacian-modified Bayesian score.
Mahalanobis Distance: The Mahalanobis distance (MD) is the distance to the center of the training data. The larger the MD, the less trustworthy the prediction.
Mahalanobis Distance p-value: The p-value gives the fraction of training data with an MD greater than or equal to the one for the given sample, assuming normally distributed data. The smaller the p-value, the less trustworthy the prediciton. For highly non-normal X properties (e.g., fingerprints), the MD p-value is wildly inaccurate.

Structural Similar Compounds

Name	Cefpiramide Sodium (Free acid form)	Latomoxef	Cefoperazone
Structure	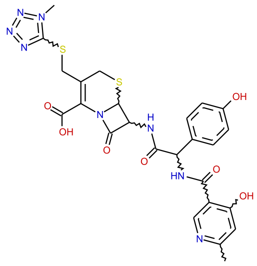	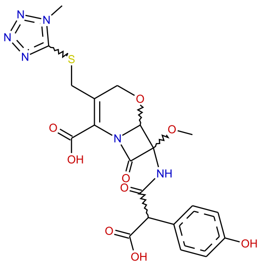	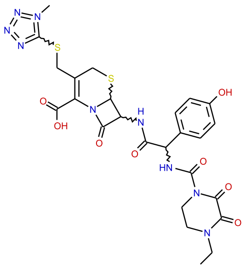
Actual Endpoint	Non-Toxic	Toxic	Non-Toxic
Predicted Endpoint	Non-Toxic	Non-Toxic	Non-Toxic
Distance	0.760	0.821	0.850
Reference	Kiso to Rinsho 17:1000-1004; 1983	Chemotherapy 28:1119-1141; 1980	Chemotherapy 28:268-291; 1980

Model Applicability

Unknown features are fingerprint features in the query molecule, but not found or appearing too infrequently in the training set.

1. All properties and OPS components are within expected ranges.

Feature Contribution

Top features for positive contribution

Fingerprint	Bit/Smiles	Feature Structure	Score	Toxic in training set
SCFP_6	1702724181	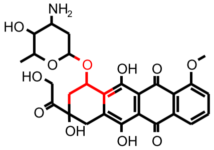 [*]CC(O[*])[c](:[*]):[*]	0.558	9 out of 9

SCFP_6	-2031220028	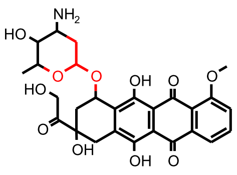 [*]CC(O[*])O[*]	0.538	7 out of 7
SCFP_6	-37268149	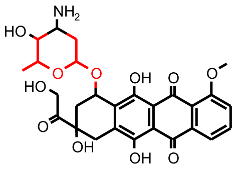 [*]OC1C[*]C([*])C(C)O 1	0.523	6 out of 6
**Top Features for negative contribution**				
**Fingerprint**	**Bit/Smiles**	**Feature Structure**	**Score**	**Toxic in training set**
SCFP_6	-1379591900	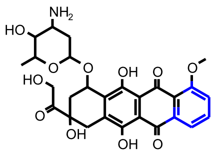 [*]:[c]1:[*]:[cH]:[cH]:[cH]:[cH]:1	-0.282	33 out of 84
SCFP_6	834949365	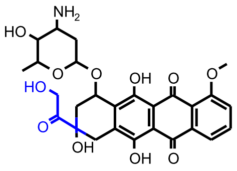 [*]C([*])([*])C(=O)CO	-0.252	1 out of 3
SCFP_6	1505318543	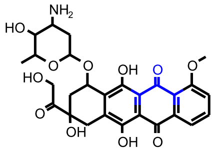 [*]:[c](:[*])C(=O)[c] (:[*]):[*]	-0.189	2 out of 5

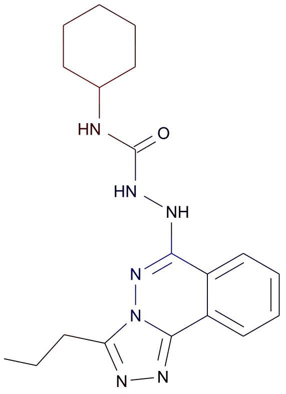

$C_{19}H_{25}N_7O$

Molecular Weight: 367.44809

ALogP: 3.681

Rotatable Bonds: 5

Acceptors: 5

Donors: 3

Model Prediction

Prediction: Mild

Probability: 0.785

Enrichment: 1.14

Bayesian Score: -1.52

Mahalanobis Distance: 10.2

Mahalanobis Distance p-value: 0.0511

Prediction: Positive if the Bayesian score is above the estimated best cutoff value from minimizing the false positive and false negative rate.
Probability: The esimated probability that the sample is in the positive category. This assumes that the Bayesian score follows a normal distribution and is different from the prediction using a cutoff.
Enrichment: An estimate of enrichment, that is, the increased likelihood (versus random) of this sample being in the category.
Bayesian Score: The standard Laplacian-modified Bayesian score.
Mahalanobis Distance: The Mahalanobis distance (MD) is the distance to the center of the training data. The larger the MD, the less trustworthy the prediction.
Mahalanobis Distance p-value: The p-value gives the fraction of training data with an MD greater than or equal to the one for the given sample, assuming normally distributed data. The smaller the p-value, the less trustworthy the prediciton. For highly non-normal X properties (e.g., fingerprints), the MD p-value is wildly inaccurate.

Structural Similar Compounds

Name	ANTHRAQUINONE; 1-((2-HYDROXYETHYL)AMINO)-4-(METHYLAMINO)-	ANTHRAQUINONE; 1-AMINO-4-HYDROXY-2-PHENOXY-	METHANE;TRIS(4-AMINOPHENYL)-
Structure	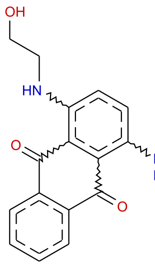	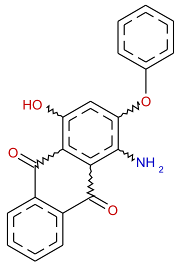	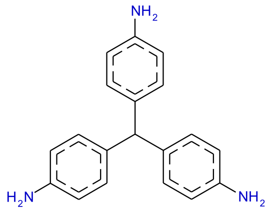
Actual Endpoint	Mild	Mild	Moderate_Severe
Predicted Endpoint	Mild	Mild	Moderate_Severe
Distance	0.695	0.733	0.733
Reference	28ZPAK 245;72	28ZPAK 239;72	28ZPAK-;73;72

Model Applicability

Unknown features are fingerprint features in the query molecule, but not found or appearing too infrequently in the training set.

1. All properties and OPS components are within expected ranges.
2. Unknown FCFP_2 feature: -1151884458: [*]N[c](:n:[*]):[c](:[*]):[*]
3. Unknown FCFP_2 feature: -1564473960: [*]:n1:[*]:[*]:n:[c]:1:[c](:[*]):[*]
4. Unknown FCFP_2 feature: -885461129: [*]NNC(=[*])[*]

Feature Contribution

Top features for positive contribution

Fingerprint	Bit/Smiles	Feature Structure	Score	Moderate_Severe in training set
FCFP_10	-796673622	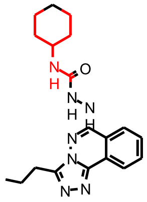 [*]C(=[*])NC1CC[*]CC1	0.317	4 out of 4

FCFP_10	-1539162406	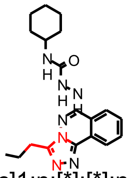 [*]C[c]1:n:[*]:[*]:n:1:[*]	0.294	3 out of 3
FCFP_10	654530535	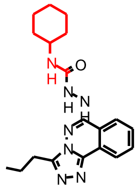 [*]C(=[*])NC1CCCCC1	0.294	3 out of 3

Top Features for negative contribution

Fingerprint	Bit/Smiles	Feature Structure	Score	Moderate_Severe in training set
FCFP_10	4427049	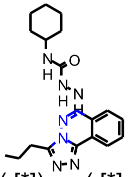 [*][c](:[*]):n:n(:[*]):[*]	-1.29	0 out of 4
FCFP_10	-332197802	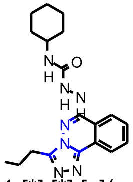 [*][c]1:[*]:[*]:[c](:[*]):n:1:n:[*]	-0.507	0 out of 1
FCFP_10	1294344583	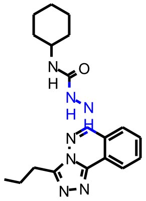 [*]NN[c](:[*]):[*]	-0.507	0 out of 1

7b

TOPKAT_Ocular_Irritancy_Mild_vs_Moderate_Severe

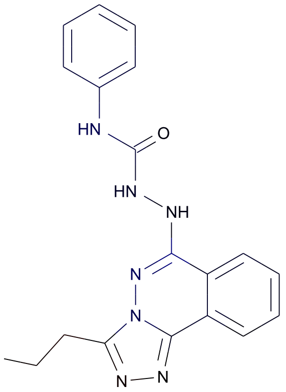

$C_{19}H_{19}N_7O$
Molecular Weight: 361.40046
ALogP: 3.399
Rotatable Bonds: 5
Acceptors: 5
Donors: 3

Model Prediction

Prediction: Mild
Probability: 0.643
Enrichment: 0.933
Bayesian Score: -4.44
Mahalanobis Distance: 6.86
Mahalanobis Distance p-value: 0.999

Prediction: Positive if the Bayesian score is above the estimated best cutoff value from minimizing the false positive and false negative rate.
Probability: The esimated probability that the sample is in the positive category. This assumes that the Bayesian score follows a normal distribution and is different from the prediction using a cutoff.
Enrichment: An estimate of enrichment, that is, the increased likelihood (versus random) of this sample being in the category.
Bayesian Score: The standard Laplacian-modified Bayesian score.
Mahalanobis Distance: The Mahalanobis distance (MD) is the distance to the center of the training data. The larger the MD, the less trustworthy the prediction.
Mahalanobis Distance p-value: The p-value gives the fraction of training data with an MD greater than or equal to the one for the given sample, assuming normally distributed data. The smaller the p-value, the less trustworthy the prediciton. For highly non-normal X properties (e.g., fingerprints), the MD p-value is wildly inaccurate.

Structural Similar Compounds

Name	ANTHRAQUINONE; 1-((2-HYDROXYETHYL)AMINO)-4-(METHYLAMINO)-	METHANE;TRIS(4-AMINOPHENYL)-	ANTHRAQUINONE; 1-AMINO-4-HYDROXY-2-PHENOXY-
Structure	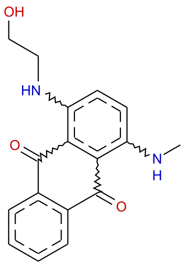	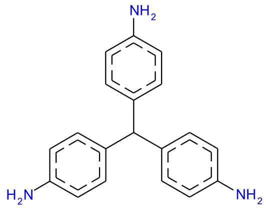	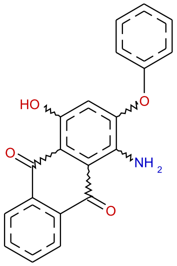
Actual Endpoint	Mild	Moderate_Severe	Mild
Predicted Endpoint	Mild	Moderate_Severe	Mild
Distance	0.653	0.709	0.719
Reference	28ZPAK 245;72	28ZPAK-;73;72	28ZPAK 239;72

Model Applicability

Unknown features are fingerprint features in the query molecule, but not found or appearing too infrequently in the training set.

1. All properties and OPS components are within expected ranges.
2. Unknown FCFP_2 feature: -1151884458: [*]N[c](:n:[*]):[c](:[*]):[*]
3. Unknown FCFP_2 feature: -1564473960: [*]:n1:[*]:[*]:n:[c]:1:[c](:[*]):[*]
4. Unknown FCFP_2 feature: -885461129: [*]NNC(=[*])[*]

Feature Contribution

Top features for positive contribution

Fingerprint	Bit/Smiles	Feature Structure	Score	Moderate_Severe in training set
FCFP_10	-1539162406	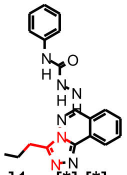 [*]C[c]1:n:[*]:[*]:n:1:[*]	0.294	3 out of 3

FCFP_10	-402549409	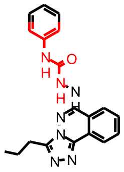 [*]NC(=O)N[c](:[cH]:[*]):[cH]:[*]	0.186	1 out of 1
FCFP_10	3	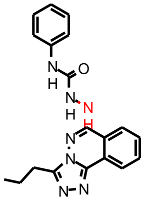 [*]N[*]	0.165	383 out of 491
**Top Features for negative contribution**				
**Fingerprint**	**Bit/Smiles**	**Feature Structure**	**Score**	**Moderate_Severe in training set**
FCFP_10	4427049	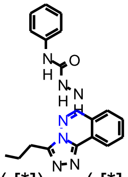 [*][c](:[*]):n:n(:[*]):[*]	-1.29	0 out of 4
FCFP_10	1294344583	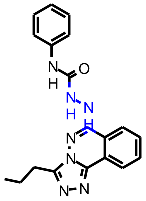 [*]NN[c](:[*]):[*]	-0.507	0 out of 1
FCFP_10	-332197802	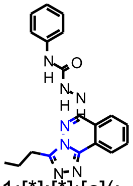 [*][c]1:[*]:[*]:[c](:[*]):n:1:n:[*]	-0.507	0 out of 1

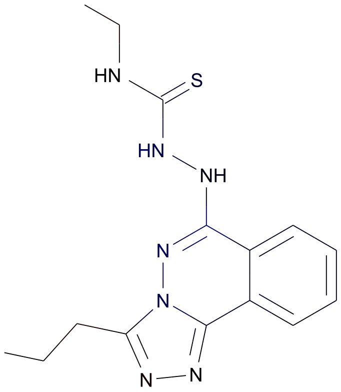

$C_{15}H_{19}N_7S$
Molecular Weight: 329.42325
ALogP: 3.388
Rotatable Bonds: 7
Acceptors: 5
Donors: 3

Structural Similar Compounds

Name	ANTHRAQUINONE; 1-((2-HYDROXYETHYL)AMINO)-4-(METHYLAMINO)-	METHANE;TRIS(4-AMINOPHENYL)-	2-NAPHTHALENESULFONIC ACID; 4-HYDROXY-7-(METHYLAMINO)-
Structure	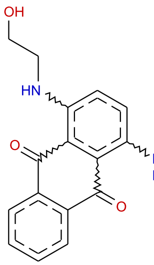	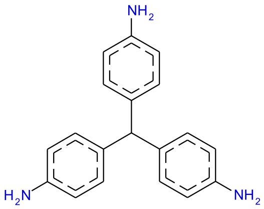	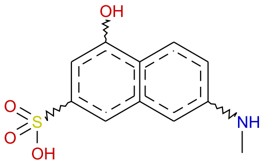
Actual Endpoint	Mild	Moderate_Severe	Mild
Predicted Endpoint	Mild	Moderate_Severe	Mild
Distance	0.673	0.737	0.747
Reference	28ZPAK 245;72	28ZPAK-;73;72	28ZPAK 190;72

Model Prediction

Prediction: Mild
Probability: 0.734
Enrichment: 1.07
Bayesian Score: -2.85
Mahalanobis Distance: 7.55
Mahalanobis Distance p-value: 0.981

Prediction: Positive if the Bayesian score is above the estimated best cutoff value from minimizing the false positive and false negative rate.
Probability: The esimated probability that the sample is in the positive category. This assumes that the Bayesian score follows a normal distribution and is different from the prediction using a cutoff.
Enrichment: An estimate of enrichment, that is, the increased likelihood (versus random) of this sample being in the category.
Bayesian Score: The standard Laplacian-modified Bayesian score.
Mahalanobis Distance: The Mahalanobis distance (MD) is the distance to the center of the training data. The larger the MD, the less trustworthy the prediction.
Mahalanobis Distance p-value: The p-value gives the fraction of training data with an MD greater than or equal to the one for the given sample, assuming normally distributed data. The smaller the p-value, the less trustworthy the prediciton. For highly non-normal X properties (e.g., fingerprints), the MD p-value is wildly inaccurate.

Model Applicability

Unknown features are fingerprint features in the query molecule, but not found or appearing too infrequently in the training set.

1. All properties and OPS components are within expected ranges.
2. Unknown FCFP_2 feature: -1151884458: [*]N[c](:n:[*]):[c](:[*]):[*]
3. Unknown FCFP_2 feature: -1564473960: [*]:n1:[*]:[*]:n:[c]:1:[c](:[*]):[*]
4. Unknown FCFP_2 feature: -885461129: [*]NNC(=[*])[*]

Feature Contribution

Top features for positive contribution				
**Fingerprint**	**Bit/Smiles**	**Feature Structure**	**Score**	**Moderate_Severe in training set**
FCFP_10	-1939253119	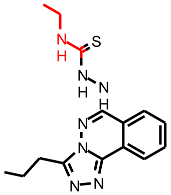 [*]C(=[*])NCC	0.332	5 out of 5

FCFP_10	-1539162406	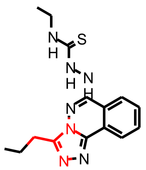 [*]C[c]1:n:[*]:[*]:n:1:[*]	0.294	3 out of 3
FCFP_10	-1272709286	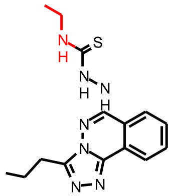 [*]NCC	0.285	234 out of 266

Top Features for negative contribution

**Fingerprint**	**Bit/Smiles**	**Feature Structure**	**Score**	**Moderate_Severe in training set**
FCFP_10	4427049	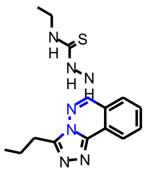 [*][c](:[*]):n:n(:[*]):[*]	-1.29	0 out of 4
FCFP_10	-332197802	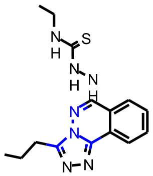 [*][c]1:[*]:[*]:[*]:[c](:[*]):n:1:n:[*]	-0.507	0 out of 1
FCFP_10	1934560662	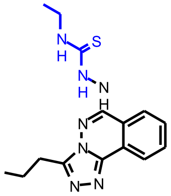 [*]NC(=S)NCC	-0.507	0 out of 1

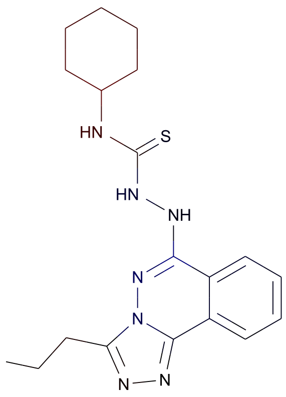

$C_{19}H_{25}N_7S$
Molecular Weight: 383.51369
ALogP: 4.898
Rotatable Bonds: 7
Acceptors: 5
Donors: 3

Model Prediction

Prediction: Mild
Probability: 0.79
Enrichment: 1.15
Bayesian Score: -1.36
Mahalanobis Distance: 10.2
Mahalanobis Distance p-value: 0.0543

Prediction: Positive if the Bayesian score is above the estimated best cutoff value from minimizing the false positive and false negative rate.
Probability: The esimated probability that the sample is in the positive category. This assumes that the Bayesian score follows a normal distribution and is different from the prediction using a cutoff.
Enrichment: An estimate of enrichment, that is, the increased likelihood (versus random) of this sample being in the category.
Bayesian Score: The standard Laplacian-modified Bayesian score.
Mahalanobis Distance: The Mahalanobis distance (MD) is the distance to the center of the training data. The larger the MD, the less trustworthy the prediction.
Mahalanobis Distance p-value: The p-value gives the fraction of training data with an MD greater than or equal to the one for the given sample, assuming normally distributed data. The smaller the p-value, the less trustworthy the prediciton. For highly non-normal X properties (e.g., fingerprints), the MD p-value is wildly inaccurate.

Structural Similar Compounds

Name	4;4'-DIAMINO-1;1'-DIANTHRIMIDE	ANTHRAQUINONE; 1-AMINO-4-HYDROXY-2-PHENOXY-	ANTHRAQUINONE; 1-((2-HYDROXYETHYL)AMINO)-4-(METHYLAMINO)-
Structure	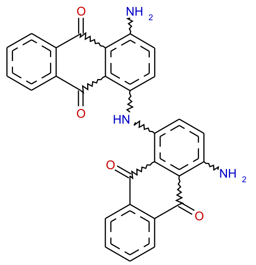	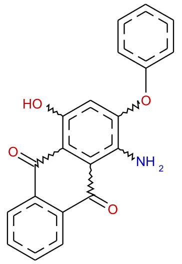	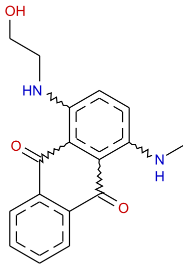
Actual Endpoint	Mild	Mild	Mild
Predicted Endpoint	Mild	Mild	Mild
Distance	0.764	0.800	0.828
Reference	28ZPAK-;125;72	28ZPAK 239;72	28ZPAK 245;72

Model Applicability

Unknown features are fingerprint features in the query molecule, but not found or appearing too infrequently in the training set.

1. All properties and OPS components are within expected ranges.
2. Unknown FCFP_2 feature: -1151884458: [*]N[c](:n:[*]):[c](:[*]):[*]
3. Unknown FCFP_2 feature: -1564473960: [*]:n1:[*]:[*]:n:[c]:1:[c](:[*]):[*]
4. Unknown FCFP_2 feature: -885461129: [*]NNC(=[*])[*]

Feature Contribution

Top features for positive contribution				
Fingerprint	Bit/Smiles	Feature Structure	Score	Moderate_Severe in training set
FCFP_10	-796673622	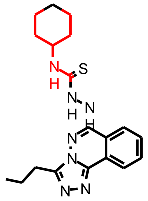 [*]C(=[*])NC1CC[*]CC1	0.317	4 out of 4

FCFP_10	-1539162406	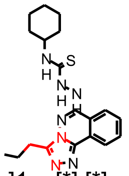 [*]C[c]1:n:[*]:[*]:n:1:[*]	0.294	3 out of 3
FCFP_10	654530535	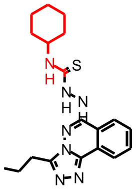 [*]C(=[*])NC1CCCCC1	0.294	3 out of 3
**Top Features for negative contribution**				
**Fingerprint**	**Bit/Smiles**	**Feature Structure**	**Score**	**Moderate_Severe in training set**
FCFP_10	4427049	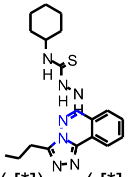 [*][c](:[*]):n:n(:[*]):[*]	-1.29	0 out of 4
FCFP_10	1294344583	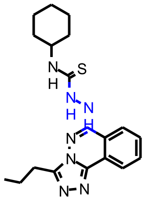 [*]NN[c](:[*]):[*]	-0.507	0 out of 1
FCFP_10	-332197802	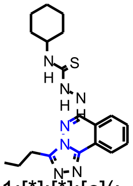 [*][c]1:[*]:[*]:[c](:[*]):n:1:n:[*]	-0.507	0 out of 1

TOPKAT_Ocular_Irritancy_Mild_vs_Moderate_Severe

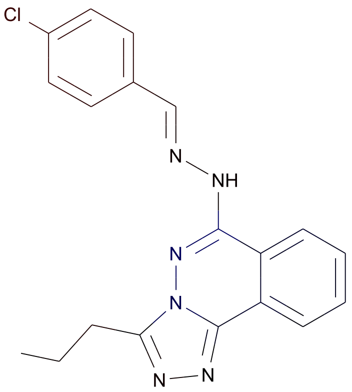

$C_{19}H_{17}ClN_6$

Molecular Weight: 364.83147

ALogP: 4.967

Rotatable Bonds: 5

Acceptors: 5

Donors: 1

Model Prediction

Prediction: Mild

Probability: 0.802

Enrichment: 1.16

Bayesian Score: -0.913

Mahalanobis Distance: 7.84

Mahalanobis Distance p-value: 0.951

Prediction: Positive if the Bayesian score is above the estimated best cutoff value from minimizing the false positive and false negative rate.
Probability: The esimated probability that the sample is in the positive category. This assumes that the Bayesian score follows a normal distribution and is different from the prediction using a cutoff.
Enrichment: An estimate of enrichment, that is, the increased likelihood (versus random) of this sample being in the category.
Bayesian Score: The standard Laplacian-modified Bayesian score.
Mahalanobis Distance: The Mahalanobis distance (MD) is the distance to the center of the training data. The larger the MD, the less trustworthy the prediction.
Mahalanobis Distance p-value: The p-value gives the fraction of training data with an MD greater than or equal to the one for the given sample, assuming normally distributed data. The smaller the p-value, the less trustworthy the prediciton. For highly non-normal X properties (e.g., fingerprints), the MD p-value is wildly inaccurate.

Structural Similar Compounds

Name	1-BENZOYLAMINO-4-METHOXY-5-CHLORANTHRAQUINONE	ANTHRAQUINONE; 1;1'-IMINODI-	BENZILIC ACID; 4;4'-DICHLORO-; ISOPROPYL ESTER
Structure	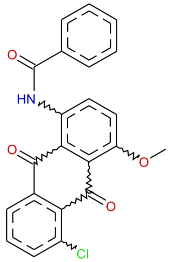	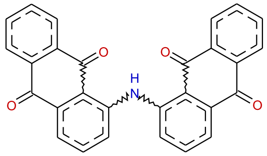	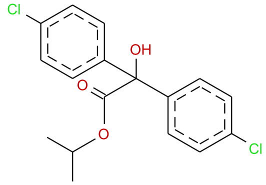
Actual Endpoint	Mild	Mild	Moderate_Severe
Predicted Endpoint	Mild	Mild	Moderate_Severe
Distance	0.601	0.616	0.637
Reference	28ZPAK-;90;72	28ZPAK-;125;72	CIGET* -;-;77

Model Applicability

Unknown features are fingerprint features in the query molecule, but not found or appearing too infrequently in the training set.

1. All properties and OPS components are within expected ranges.
2. Unknown FCFP_2 feature: -1151884458: [*]N[c](:n:[*]):[c](:[*]):[*]
3. Unknown FCFP_2 feature: -1564473960: [*]:n1:[*]:[*]:n:[c]:1:[c](:[*]):[*]
4. Unknown FCFP_2 feature: 1294285001: [*]=NN[c](:[*]):[*]
5. Unknown FCFP_2 feature: 581019816: [*]N\N=C\[*]

Feature Contribution

Top features for positive contribution				
**Fingerprint**	**Bit/Smiles**	**Feature Structure**	**Score**	**Moderate_Severe in training set**
FCFP_10	-149636017	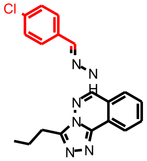 [*]=C[c]1:[cH]:[cH]:[c](Cl):[cH]:[cH]:1	0.352	7 out of 7

FCFP_10	-1508180856	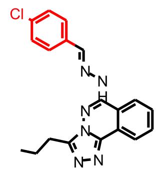 [*][c]1:[cH]:[cH]:[c](Cl):[cH]:[cH]:1	0.329	16 out of 17
FCFP_10	-745491832	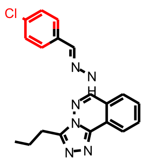 Cl[c]1:[cH]:[cH]:[*]:[cH]:[cH]:1	0.304	29 out of 32
**Top Features for negative contribution**				
**Fingerprint**	**Bit/Smiles**	**Feature Structure**	**Score**	**Moderate_Severe in training set**
FCFP_10	4427049	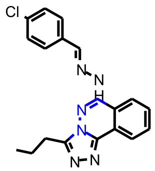 [*][c](:[*]):n:n(:[*]):[*]	-1.29	0 out of 4
FCFP_10	-332197802	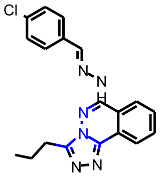 [*][c]1:[*]:[*]:[c](:[*]):n:1:n:[*]	-0.507	0 out of 1
FCFP_10	-1320007763	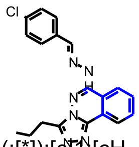 [*][c](:[*]):[c]1:[cH]:[cH]:[cH]:[*]:[c]:1:[*]	-0.316	19 out of 40

9b TOPKAT_Ocular_Irritancy_Mild_vs_Moderate_Severe

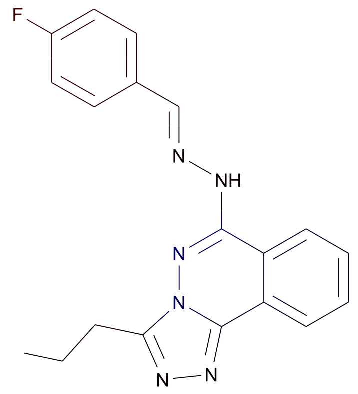

$C_{19}H_{17}FN_6$
Molecular Weight: 348.37688
ALogP: 4.508
Rotatable Bonds: 5
Acceptors: 5
Donors: 1

Model Prediction

Prediction: Mild
Probability: 0.802
Enrichment: 1.16
Bayesian Score: -0.913
Mahalanobis Distance: 7.8
Mahalanobis Distance p-value: 0.957

Prediction: Positive if the Bayesian score is above the estimated best cutoff value from minimizing the false positive and false negative rate.
Probability: The esimated probability that the sample is in the positive category. This assumes that the Bayesian score follows a normal distribution and is different from the prediction using a cutoff.
Enrichment: An estimate of enrichment, that is, the increased likelihood (versus random) of this sample being in the category.
Bayesian Score: The standard Laplacian-modified Bayesian score.
Mahalanobis Distance: The Mahalanobis distance (MD) is the distance to the center of the training data. The larger the MD, the less trustworthy the prediction.
Mahalanobis Distance p-value: The p-value gives the fraction of training data with an MD greater than or equal to the one for the given sample, assuming normally distributed data. The smaller the p-value, the less trustworthy the prediciton. For highly non-normal X properties (e.g., fingerprints), the MD p-value is wildly inaccurate.

Structural Similar Compounds

Name	1-BENZOYLAMINO-4-METHOXY-5-CHLORANTHRAQUINONE	BENZILIC ACID; 4;4'-DICHLORO-; ISOPROPYL ESTER	ANTHRAQUINONE; 1;1'-IMINODI-
Structure	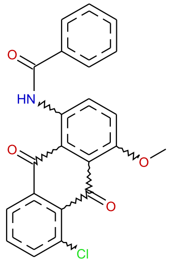	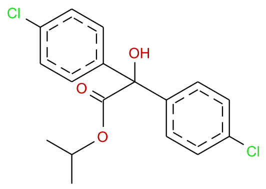	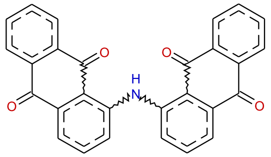
Actual Endpoint	Mild	Moderate_Severe	Mild
Predicted Endpoint	Mild	Moderate_Severe	Mild
Distance	0.595	0.634	0.653
Reference	28ZPAK-;90;72	CIGET* -;-;77	28ZPAK-;125;72

Model Applicability

Unknown features are fingerprint features in the query molecule, but not found or appearing too infrequently in the training set.

1. All properties and OPS components are within expected ranges.
2. Unknown FCFP_2 feature: -1151884458: [*]N[c](:n:[*]):[c](:[*]):[*]
3. Unknown FCFP_2 feature: -1564473960: [*]:n1:[*]:[*]:n:[c]:1:[c](:[*]):[*]
4. Unknown FCFP_2 feature: 1294285001: [*]=NN[c](:[*]):[*]
5. Unknown FCFP_2 feature: 581019816: [*]N\N=C\[*]

Feature Contribution

Top features for positive contribution

Fingerprint	Bit/Smiles	Feature Structure	Score	Moderate_Severe in training set
FCFP_10	-149636017	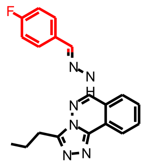 [*]=C[c]1:[cH]:[cH]:[c](Cl):[cH]:[cH]:1	0.352	7 out of 7

FCFP_10	-1508180856	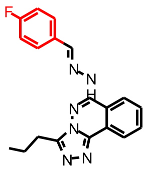 [*][c]1:[cH]:[cH]:[c](Cl):[cH]:[cH]:1	0.329	16 out of 17
FCFP_10	-745491832	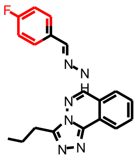 Cl[c]1:[cH]:[cH]:[*]:[cH]:[cH]:1	0.304	29 out of 32
**Top Features for negative contribution**				
**Fingerprint**	**Bit/Smiles**	**Feature Structure**	**Score**	**Moderate_Severe in training set**
FCFP_10	4427049	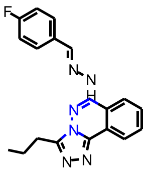 [*][c](:[*]):n:n(:[*]):[*]	-1.29	0 out of 4
FCFP_10	-332197802	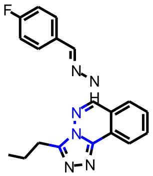 [*][c]1:[*]:[*]:[c](:[*]):n:1:n:[*]	-0.507	0 out of 1
FCFP_10	-1320007763	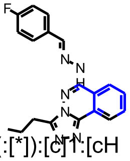 [*][c](:[*]):[c]1:[cH]:[cH]:[cH]:[*]:[c]:1:[*]	-0.316	19 out of 40

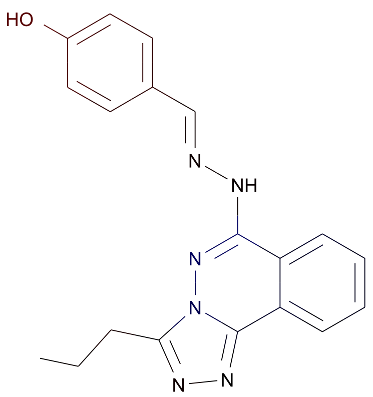

$C_{19}H_{18}N_6O$
Molecular Weight: 346.38582
ALogP: 4.061
Rotatable Bonds: 5
Acceptors: 6
Donors: 2

Structural Similar Compounds

Name	ANTHRAQUINONE; 1-AMINO-4-HYDROXY-2-PHENOXY-	s-TRIAZINE; 2;4-BIS(ISOPROPYLAMINO0-6-(METHYLTHIO)-	s-TRIAZINE; 2-(tert-BUTYLAMINO)-4-(ETHYLAMINO)-6-(METHYLTHIO)-
Structure	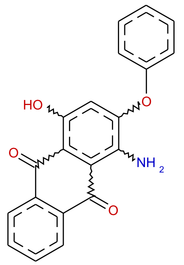	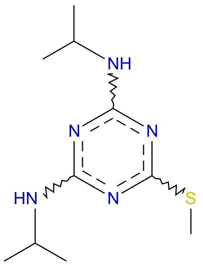	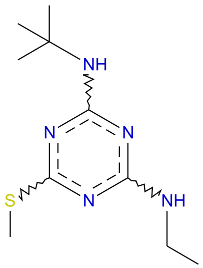
Actual Endpoint	Mild	Mild	Moderate_Severe
Predicted Endpoint	Mild	Mild	Moderate_Severe
Distance	0.615	0.691	0.696
Reference	28ZPAK 239;72	CIGET* -;-;77	CIGET* -;-;77

Model Prediction

Prediction: Mild
Probability: 0.811
Enrichment: 1.18
Bayesian Score: -0.525
Mahalanobis Distance: 8.13
Mahalanobis Distance p-value: 0.893

Prediction: Positive if the Bayesian score is above the estimated best cutoff value from minimizing the false positive and false negative rate.
Probability: The esimated probability that the sample is in the positive category. This assumes that the Bayesian score follows a normal distribution and is different from the prediction using a cutoff.
Enrichment: An estimate of enrichment, that is, the increased likelihood (versus random) of this sample being in the category.
Bayesian Score: The standard Laplacian-modified Bayesian score.
Mahalanobis Distance: The Mahalanobis distance (MD) is the distance to the center of the training data. The larger the MD, the less trustworthy the prediction.
Mahalanobis Distance p-value: The p-value gives the fraction of training data with an MD greater than or equal to the one for the given sample, assuming normally distributed data. The smaller the p-value, the less trustworthy the prediciton. For highly non-normal X properties (e.g., fingerprints), the MD p-value is wildly inaccurate.

Model Applicability

Unknown features are fingerprint features in the query molecule, but not found or appearing too infrequently in the training set.

1. All properties and OPS components are within expected ranges.
2. Unknown FCFP_2 feature: -1151884458: [*]N[c](:n:[*]):[c](:[*]):[*]
3. Unknown FCFP_2 feature: -1564473960: [*]:n1:[*]:[*]:n:[c]:1:[c](:[*]):[*]
4. Unknown FCFP_2 feature: 1294285001: [*]=NN[c](:[*]):[*]
5. Unknown FCFP_2 feature: 581019816: [*]N\N=C\[*]

Feature Contribution

Top features for positive contribution

Fingerprint	Bit/Smiles	Feature Structure	Score	Moderate_Severe in training set
FCFP_10	-1066794953	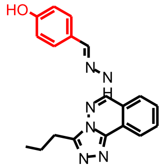 [*][c]1:[cH]:[cH]:[c](O):[cH]:[cH]:1	0.378	13 out of 13

FCFP_10	-158888774	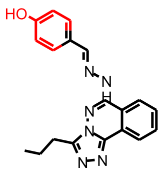 O[c]1:[cH]:[cH]:[*]:[cH]:[cH]:1	0.356	24 out of 25
FCFP_10	-1539162406	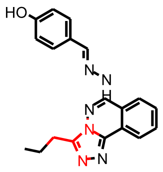 [*]C[c]1:n:[*]:[*]:n:1:[*]	0.294	3 out of 3
**Top Features for negative contribution**				
**Fingerprint**	**Bit/Smiles**	**Feature Structure**	**Score**	**Moderate_Severe in training set**
FCFP_10	4427049	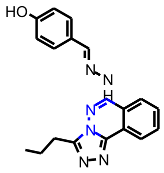 [*][c](:[*]):n:n(:[*]):[*]	-1.29	0 out of 4
FCFP_10	-332197802	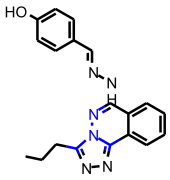 [*][c]1:[*]:[*]:[c](:[*]):n:1:n:[*]	-0.507	0 out of 1
FCFP_10	-1320007763	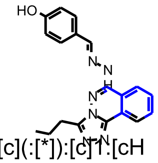 [*][c](:[*]):[c]1:[cH]:[cH]:[cH]:[*]:[c]:1:[*]	-0.316	19 out of 40

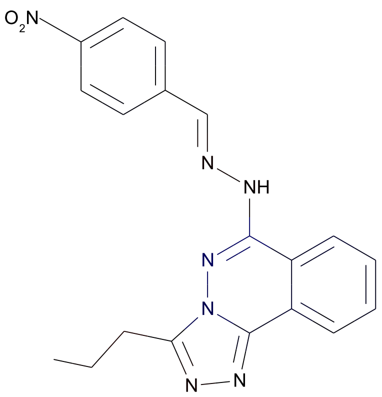

$C_{19}H_{17}N_7O_2$
Molecular Weight: 375.38397
ALogP: 4.197
Rotatable Bonds: 6
Acceptors: 7
Donors: 1

Structural Similar Compounds

Name	COLCHICINE	1;4-PENTADIENE-3-ONE;1;5-BIS(4-(2;3-DIDEHYDROTRIAZIRIDINYL)PHENYL)-	ANTHRAQUINONE; 1;1'-IMINODI-
Structure	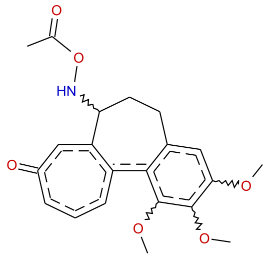	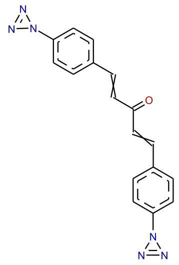	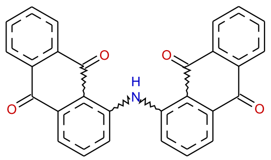
Actual Endpoint	Moderate_Severe	Mild	Mild
Predicted Endpoint	Moderate_Severe	Mild	Mild
Distance	0.709	0.741	0.766
Reference	AJOPAA 31;837;48	28ZPAK-;123;72	28ZPAK-;125;72

Model Prediction

Prediction: Mild
Probability: 0.772
Enrichment: 1.12
Bayesian Score: -1.94
Mahalanobis Distance: 7.74
Mahalanobis Distance p-value: 0.965

Prediction: Positive if the Bayesian score is above the estimated best cutoff value from minimizing the false positive and false negative rate.
Probability: The esimated probability that the sample is in the positive category. This assumes that the Bayesian score follows a normal distribution and is different from the prediction using a cutoff.
Enrichment: An estimate of enrichment, that is, the increased likelihood (versus random) of this sample being in the category.
Bayesian Score: The standard Laplacian-modified Bayesian score.
Mahalanobis Distance: The Mahalanobis distance (MD) is the distance to the center of the training data. The larger the MD, the less trustworthy the prediction.
Mahalanobis Distance p-value: The p-value gives the fraction of training data with an MD greater than or equal to the one for the given sample, assuming normally distributed data. The smaller the p-value, the less trustworthy the prediciton. For highly non-normal X properties (e.g., fingerprints), the MD p-value is wildly inaccurate.

Model Applicability

Unknown features are fingerprint features in the query molecule, but not found or appearing too infrequently in the training set.

1. All properties and OPS components are within expected ranges.
2. Unknown FCFP_2 feature: -1151884458: [*]N[c](:n:[*]):[c](:[*]):[*]
3. Unknown FCFP_2 feature: -1564473960: [*]:n1:[*]:[*]:n:[c]:1:[c](:[*]):[*]
4. Unknown FCFP_2 feature: 1294285001: [*]=NN[c](:[*]):[*]
5. Unknown FCFP_2 feature: 581019816: [*]N\N=C\[*]
6. Unknown FCFP_2 feature: -828984032: [*][N+](=[*])[c](:[cH]:[*]):[cH]:[*]
7. Unknown FCFP_2 feature: -1338588315: [*]:[c](:[*])[N+](=O)[O-]
8. Unknown FCFP_2 feature: 1872392852: [*][N+](=O)[*]
9. Unknown FCFP_2 feature: 260476081: [*][N+](=[*])[O-]

Feature Contribution

Top features for positive contribution

Fingerprint	Bit/Smiles	Feature Structure	Score	Moderate_Severe in training set

FCFP_10	-1539162406	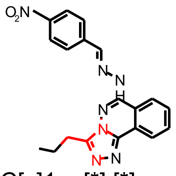 [*]C[c]1:n:[*]:[*]:n:1:[*]	0.294	3 out of 3
FCFP_10	-2100785893	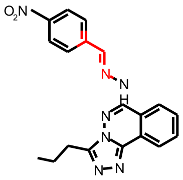 [*]\N=C\[c](:[*]):[*]	0.256	2 out of 2
FCFP_10	3	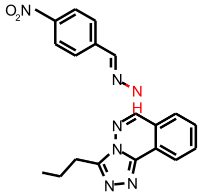 [*]N[*]	0.165	383 out of 491
**Top Features for negative contribution**				
**Fingerprint**	**Bit/Smiles**	**Feature Structure**	**Score**	**Moderate_Severe in training set**
FCFP_10	4427049	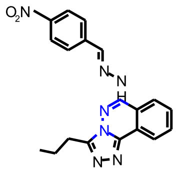 [*][c](:[*]):n:n(:[*]):[*]	-1.29	0 out of 4
FCFP_10	-332197802	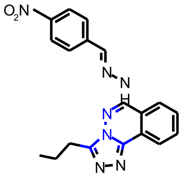 [*][c]1:[*]:[*]:[c](:[*]):n:1:n:[*]	-0.507	0 out of 1

FCFP_10	-1320007763	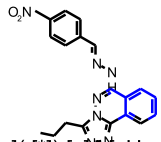 [*][c](:[*]):[c]1:[cH]:[cH]:[cH]:[*]:[c]:1:[*]	-0.316	19 out of 40

TOPKAT_Ocular_Irritancy_Mild_vs_Moderate_Severe

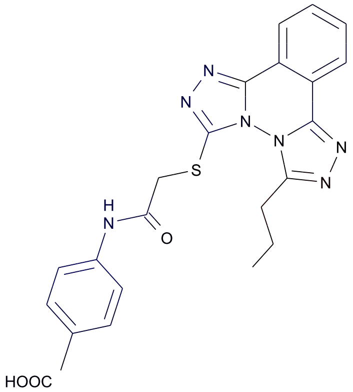

$C_{22}H_{19}N_7O_3S$
Molecular Weight: 461.49635
ALogP: 3.42
Rotatable Bonds: 7
Acceptors: 8
Donors: 2

Structural Similar Compounds

Name	4;4'-DIAMINO-1;1'-DIANTHRIMIDE	COLCHICINE	2;7-NAPHTHALENE DISULFONIC ACID;4-AMINO-5-HYDROXY-;P-TOLUENE SULFONATE (ESTER)
Structure	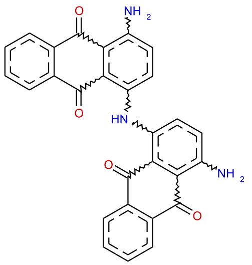	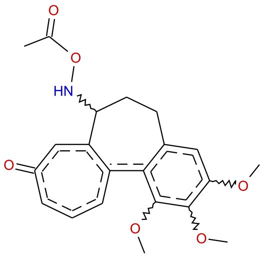	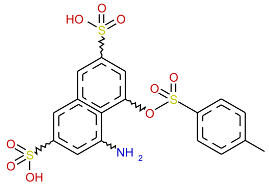
Actual Endpoint	Mild	Moderate_Severe	Moderate_Severe
Predicted Endpoint	Mild	Moderate_Severe	Moderate_Severe
Distance	0.753	0.829	0.852
Reference	28ZPAK-;125;72	AJOPAA 31;837;48	28ZPAK-;194;72

Model Prediction

Prediction: Mild

Probability: 0.715

Enrichment: 1.04

Bayesian Score: -3.24

Mahalanobis Distance: 12.2

Mahalanobis Distance p-value: 2.02e-005

Prediction: Positive if the Bayesian score is above the estimated best cutoff value from minimizing the false positive and false negative rate.
Probability: The esimated probability that the sample is in the positive category. This assumes that the Bayesian score follows a normal distribution and is different from the prediction using a cutoff.
Enrichment: An estimate of enrichment, that is, the increased likelihood (versus random) of this sample being in the category.
Bayesian Score: The standard Laplacian-modified Bayesian score.
Mahalanobis Distance: The Mahalanobis distance (MD) is the distance to the center of the training data. The larger the MD, the less trustworthy the prediction.
Mahalanobis Distance p-value: The p-value gives the fraction of training data with an MD greater than or equal to the one for the given sample, assuming normally distributed data. The smaller the p-value, the less trustworthy the prediciton. For highly non-normal X properties (e.g., fingerprints), the MD p-value is wildly inaccurate.

Model Applicability

Unknown features are fingerprint features in the query molecule, but not found or appearing too infrequently in the training set.

1. All properties and OPS components are within expected ranges.
2. Unknown FCFP_2 feature: -1564473960: [*]:n1:[*]:[*]:n:[c]:1:[c](:[*]):[*]

Feature Contribution

Top features for positive contribution

Fingerprint	Bit/Smiles	Feature Structure	Score	Moderate_Severe in training set
FCFP_10	-548632217	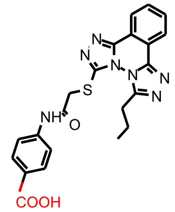 [*]C(=[*])O	0.319	54 out of 59

FCFP_10	-1539162406	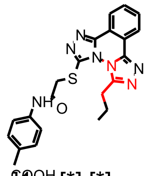 [*]C[c]1:n:[*]:[*]:n:1:[*]	0.294	3 out of 3
FCFP_10	7	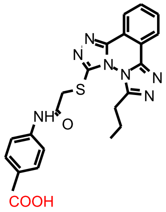 [*]O	0.219	117 out of 142

Top Features for negative contribution

Fingerprint	Bit/Smiles	Feature Structure	Score	Moderate_Severe in training set
FCFP_10	4427049	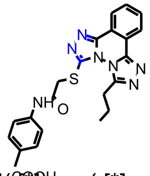 [*][c](:[*]):n:n(:[*]):[*]	-1.29	0 out of 4
FCFP_10	-332197802	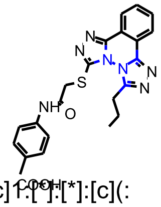 [*][c]1:[*]:[*]:[c](:[*]):n:1:n:[*]	-0.507	0 out of 1
FCFP_10	-790336137	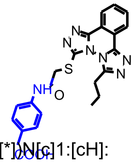 [*]C(=[*])N[c]1:[cH]:[cH]:[c](:[cH]:[cH]:1)C(=[*])[*]	-0.507	0 out of 1

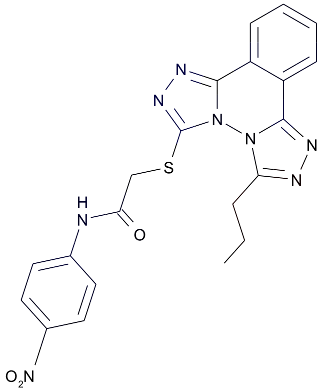

$C_{21}H_{18}N_8O_3S$
Molecular Weight: 462.48441
ALogP: 3.685
Rotatable Bonds: 7
Acceptors: 8
Donors: 1

Structural Similar Compounds

Name	COLCHICINE	1;8;9-ANTHRACENETRIOL; TRIACETATE	1;4-PENTADIENE-3-ONE;1;5-BIS(4-(2;3-DIDEHYDROTRIAZIRIDINYL)PHENYL)-
Structure	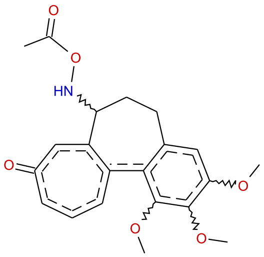	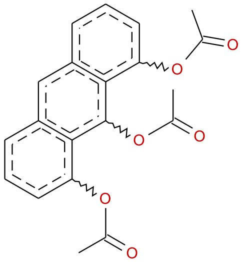	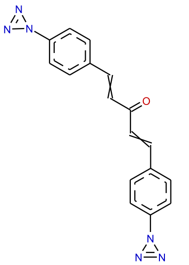
Actual Endpoint	Moderate_Severe	Moderate_Severe	Mild
Predicted Endpoint	Moderate_Severe	Moderate_Severe	Mild
Distance	0.780	0.872	0.881
Reference	AJOPAA 31;837;48	BJOPAL 53;819;69	28ZPAK-;123;72

Model Prediction

Prediction: Mild
Probability: 0.726
Enrichment: 1.05
Bayesian Score: -3.03
Mahalanobis Distance: 8.28
Mahalanobis Distance p-value: 0.85

Prediction: Positive if the Bayesian score is above the estimated best cutoff value from minimizing the false positive and false negative rate.
Probability: The esimated probability that the sample is in the positive category. This assumes that the Bayesian score follows a normal distribution and is different from the prediction using a cutoff.
Enrichment: An estimate of enrichment, that is, the increased likelihood (versus random) of this sample being in the category.
Bayesian Score: The standard Laplacian-modified Bayesian score.
Mahalanobis Distance: The Mahalanobis distance (MD) is the distance to the center of the training data. The larger the MD, the less trustworthy the prediction.
Mahalanobis Distance p-value: The p-value gives the fraction of training data with an MD greater than or equal to the one for the given sample, assuming normally distributed data. The smaller the p-value, the less trustworthy the prediciton. For highly non-normal X properties (e.g., fingerprints), the MD p-value is wildly inaccurate.

Model Applicability

Unknown features are fingerprint features in the query molecule, but not found or appearing too infrequently in the training set.

1. All properties and OPS components are within expected ranges.
2. Unknown FCFP_2 feature: -1564473960: [*]:n1:[*]:[*]:n:[c]:1:[c](:[*]):[*]
3. Unknown FCFP_2 feature: -828984032: [*][N+](=[*])[c](:[cH]:[*]):[cH]:[*]
4. Unknown FCFP_2 feature: -1338588315: [*]:[c](:[*])[N+](=O)[O-]
5. Unknown FCFP_2 feature: 1872392852: [*][N+](=O)[*]
6. Unknown FCFP_2 feature: 260476081: [*][N+](=[*])[O-]

Feature Contribution

Top features for positive contribution

Fingerprint	Bit/Smiles	Feature Structure	Score	Moderate_Severe in training set

FCFP_10	-1539162406	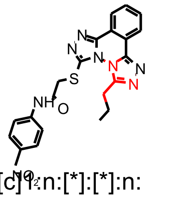 [*]C[c]1:n:[*]:[*]:n:1:[*]	0.294	3 out of 3
FCFP_10	3	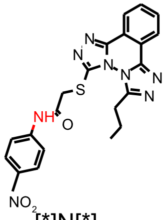 [*]N[*]	0.165	383 out of 491
FCFP_10	907007053	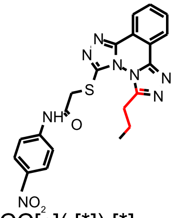 [*]CC[c](:[*]):[*]	0.138	32 out of 42

Top Features for negative contribution

**Fingerprint**	**Bit/Smiles**	**Feature Structure**	**Score**	**Moderate_Severe in training set**
FCFP_10	4427049	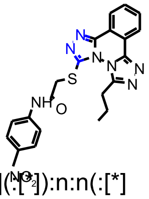 [*][c](:[*]):n:n(:[*]):[*]	-1.29	0 out of 4
FCFP_10	-332197802	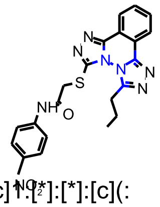 [*][c]1:[*]:[*]:[c](:[*]):n:1:n:[*]	-0.507	0 out of 1

FCFP_10	-1320007763	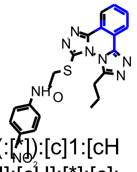 [*][c](:[*]):[c]1:[cH]:[cH]:[cH]:[*]:[c]:1:[*]	-0.316	19 out of 40

TOPKAT_Ocular_Irritancy_Mild_vs_Moderate_Severe

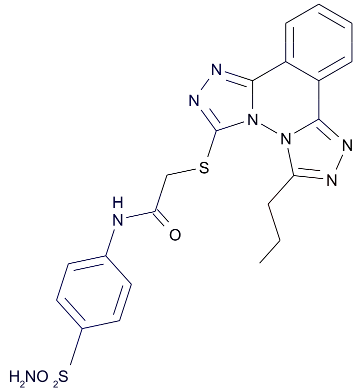

$C_{21}H_{20}N_8O_3S_2$
Molecular Weight: 496.56529
ALogP: 2.496
Rotatable Bonds: 7
Acceptors: 8
Donors: 2

Model Prediction

Prediction: Mild

Probability: 0.659

Enrichment: 0.956

Bayesian Score: -4.2

Mahalanobis Distance: 8.56

Mahalanobis Distance p-value: 0.737

Prediction: Positive if the Bayesian score is above the estimated best cutoff value from minimizing the false positive and false negative rate.
Probability: The esimated probability that the sample is in the positive category. This assumes that the Bayesian score follows a normal distribution and is different from the prediction using a cutoff.
Enrichment: An estimate of enrichment, that is, the increased likelihood (versus random) of this sample being in the category.
Bayesian Score: The standard Laplacian-modified Bayesian score.
Mahalanobis Distance: The Mahalanobis distance (MD) is the distance to the center of the training data. The larger the MD, the less trustworthy the prediction.
Mahalanobis Distance p-value: The p-value gives the fraction of training data with an MD greater than or equal to the one for the given sample, assuming normally distributed data. The smaller the p-value, the less trustworthy the prediciton. For highly non-normal X properties (e.g., fingerprints), the MD p-value is wildly inaccurate.

Structural Similar Compounds

Name	2;2'-Stilbenedisulfonic acid; 4;4'-dinitro-	2;7-NAPHTHALENE DISULFONIC ACID;4-AMINO-5-HYDROXY-;P-TOLUENE SULFONATE (ESTER)	4;4'-DIAMINO-1;1'-DIANTHRIMIDE
Structure	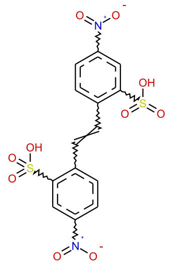	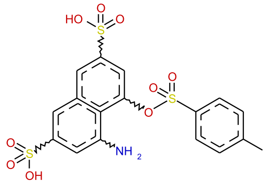	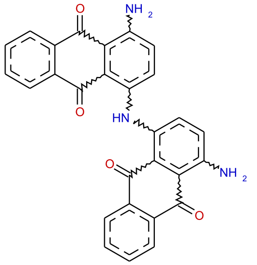
Actual Endpoint	Mild	Moderate_Severe	Mild
Predicted Endpoint	Mild	Moderate_Severe	Mild
Distance	0.783	0.787	0.803
Reference	Prehled Prumyslove Toxikologie; Organicke Latky; Marhold; J. pp 1062;86	28ZPAK-;194;72	28ZPAK-;125;72

Model Applicability

Unknown features are fingerprint features in the query molecule, but not found or appearing too infrequently in the training set.

1. All properties and OPS components are within expected ranges.
2. Unknown FCFP_2 feature: -1564473960: [*]:n1:[*]:[*]:n:[c]:1:[c](:[*]):[*]

Feature Contribution

Top features for positive contribution				
Fingerprint	Bit/Smiles	Feature Structure	Score	Moderate_Severe in training set
FCFP_10	-1539162406	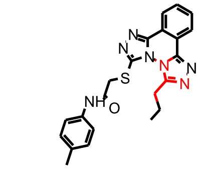 [*]C[c]1:n:[*]:[*]:n:1:[*]	0.294	3 out of 3

FCFP_10	1070061035	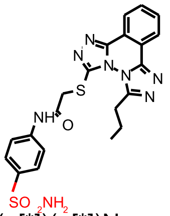 [*]S(=[*])(=[*])N	0.239	284 out of 338
FCFP_10	1763397430	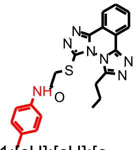 [*]N[c]1:[cH]:[cH]:[c](:[cH]:[cH]:1)S(=O) (=O)N	0.186	1 out of 1

Top Features for negative contribution

Fingerprint	Bit/Smiles	Feature Structure	Score	Moderate_Severe in training set
FCFP_10	4427049	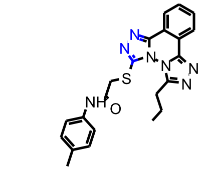 [*][c](:[*]):n:n(:[*]):[*]	-1.29	0 out of 4
FCFP_10	-332197802	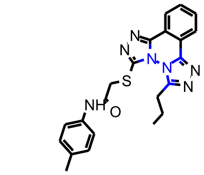 [*][c]1:[*]:[*]:[c](: [*]):n:1:n:[*]	-0.507	0 out of 1
FCFP_10	-790336137	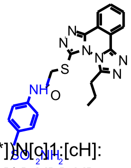 [*]C(=[*])N[c]1:[cH]: [cH]:[c](:[cH]:[cH]: 1)C(=[*])[*]	-0.507	0 out of 1

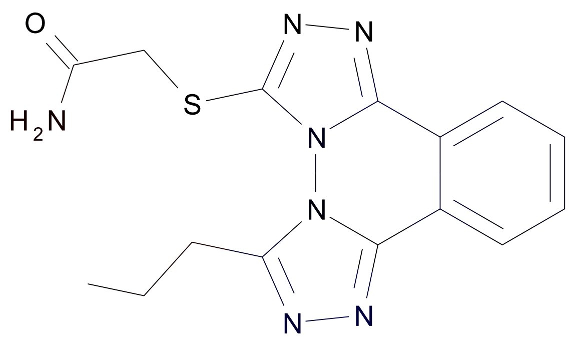

$C_{15}H_{15}N_7OS$

Molecular Weight: 341.39089

ALogP: 2.008

Rotatable Bonds: 5

Acceptors: 6

Donors: 1

Structural Similar Compounds

Name	2;5-DICHLORO-4(3'-METHYL-5' PYRAZOLON-1'-YL)BENZENE SULFONIC ACID	Benzenesulfonic acid; 3;5-bis(methoxycarbonyl)-; sodium salt	Benzenesulfonic acid; 2-anilino-5-nitro-
Structure	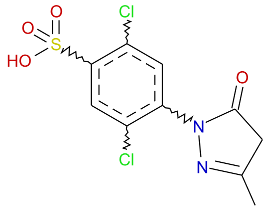	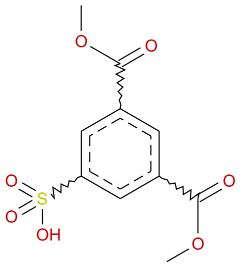	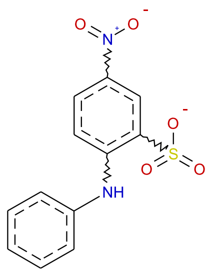
Actual Endpoint	Mild	Moderate_Severe	Moderate_Severe
Predicted Endpoint	Mild	Mild	Mild
Distance	0.607	0.662	0.678
Reference	28ZPAK-;186;72	Prehled Prumyslove Toxikologie; Organicke Latky; Marhold; J. -;1059;86	Prehled Prumyslove Toxikologie; Organicke Latky; Marhold; J. -;1061;86

Model Prediction

Prediction: Mild

Probability: 0.777

Enrichment: 1.13

Bayesian Score: -1.78

Mahalanobis Distance: 7.91

Mahalanobis Distance p-value: 0.941

Prediction: Positive if the Bayesian score is above the estimated best cutoff value from minimizing the false positive and false negative rate.
Probability: The esimated probability that the sample is in the positive category. This assumes that the Bayesian score follows a normal distribution and is different from the prediction using a cutoff.
Enrichment: An estimate of enrichment, that is, the increased likelihood (versus random) of this sample being in the category.
Bayesian Score: The standard Laplacian-modified Bayesian score.
Mahalanobis Distance: The Mahalanobis distance (MD) is the distance to the center of the training data. The larger the MD, the less trustworthy the prediction.
Mahalanobis Distance p-value: The p-value gives the fraction of training data with an MD greater than or equal to the one for the given sample, assuming normally distributed data. The smaller the p-value, the less trustworthy the prediciton. For highly non-normal X properties (e.g., fingerprints), the MD p-value is wildly inaccurate.

Model Applicability

Unknown features are fingerprint features in the query molecule, but not found or appearing too infrequently in the training set.

1. All properties and OPS components are within expected ranges.
2. Unknown FCFP_2 feature: -1564473960: [*]:n1:[*]:[*]:n:[c]:1:[c](:[*]):[*]

Feature Contribution

Top features for positive contribution

Fingerprint	Bit/Smiles	Feature Structure	Score	Moderate_Severe in training set
FCFP_10	-1539162406	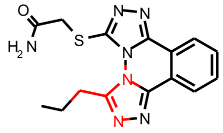 [*]C[c]1:n:[*]:[*]:n: 1:[*]	0.294	3 out of 3

FCFP_10	1070061035	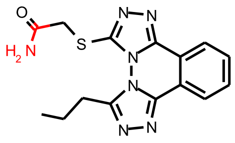 [*]S(=[*])(=[*])N	0.239	284 out of 338
FCFP_10	3	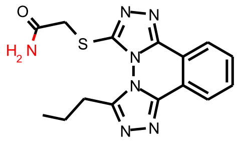 [*]N[*]	0.165	383 out of 491

Top Features for negative contribution

Fingerprint	Bit/Smiles	Feature Structure	Score	Moderate_Severe in training set
FCFP_10	4427049	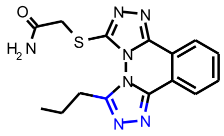 [*][c](:[*]):n:n(:[*]):[*]	-1.29	0 out of 4
FCFP_10	-332197802	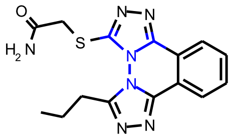 [*][c]1:[*]:[*]:[c](: [*]):n:1:n:[*]	-0.507	0 out of 1
FCFP_10	-1320007763	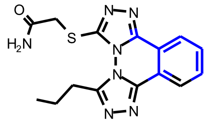 [*][c](:[*]):[c]1:[cH]:[cH]:[cH]:[*]:[c]: 1:[*]	-0.316	19 out of 40

Doxorubicin

TOPKAT_Ocular_Irritancy_Mild_vs_Moderate_Severe

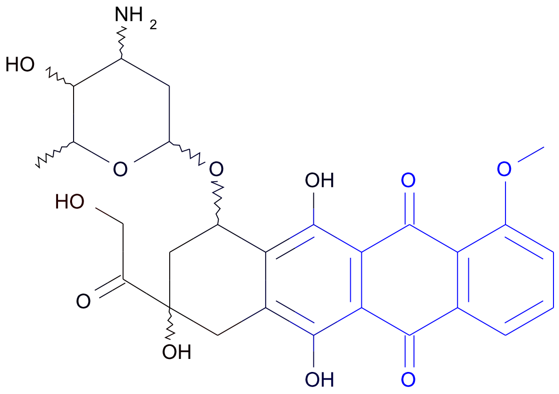

$C_{27}H_{29}NO_{11}$

Molecular Weight: 543.51925

ALogP: -4.4e-002

Rotatable Bonds: 5

Acceptors: 12

Donors: 6

Structural Similar Compounds

Name	Methanol; (s-triazine-2;4;6-triyltrinitrilo)hexa-	2-Naphthalenesulfonic acid; 5;6'-iminobis(1-hydroxy-	1;3;6-NAPHTHALENE TRISULFONIC ACID;7-AMINO-
Structure	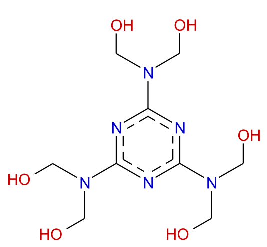	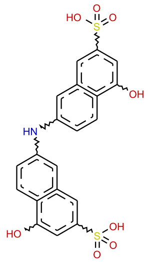	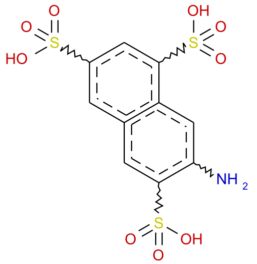
Actual Endpoint	Moderate_Severe	Mild	Mild
Predicted Endpoint	Moderate_Severe	Mild	Mild
Distance	0.947	0.991	1.168
Reference	Prehled Prumyslove Toxikologie; Organicke Latky; Marhold; J. -;876;86	Prehled Prumyslove Toxikologie; Organicke Latky; Marhold; J. pp 1065;86	28ZPAK-;190;72

Model Prediction

Prediction: Mild

Probability: 0.00731

Enrichment: 0.0106

Bayesian Score: -15

Mahalanobis Distance: 16.1

Mahalanobis Distance p-value: 1.56e-017

Prediction: Positive if the Bayesian score is above the estimated best cutoff value from minimizing the false positive and false negative rate.
Probability: The esimated probability that the sample is in the positive category. This assumes that the Bayesian score follows a normal distribution and is different from the prediction using a cutoff.
Enrichment: An estimate of enrichment, that is, the increased likelihood (versus random) of this sample being in the category.
Bayesian Score: The standard Laplacian-modified Bayesian score.
Mahalanobis Distance: The Mahalanobis distance (MD) is the distance to the center of the training data. The larger the MD, the less trustworthy the prediction.
Mahalanobis Distance p-value: The p-value gives the fraction of training data with an MD greater than or equal to the one for the given sample, assuming normally distributed data. The smaller the p-value, the less trustworthy the prediciton. For highly non-normal X properties (e.g., fingerprints), the MD p-value is wildly inaccurate.

Model Applicability

Unknown features are fingerprint features in the query molecule, but not found or appearing too infrequently in the training set.

1. OPS PC18 out of range. Value: -4.3032. Training min, max, SD, explained variance: -4.1566, 3.8733, 1.069, 0.0139.
2. OPS PC20 out of range. Value: -3.1531. Training min, max, SD, explained variance: -3.1297, 4.2414, 1.03, 0.0129.

Feature Contribution

Top features for positive contribution				
Fingerprint	Bit/Smiles	Feature Structure	Score	Moderate_Severe in training set

FCFP_10	-1601875224	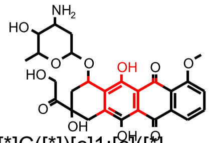 [*]C([*])[c]1:[c]([*]):[*]:[c]([*]):[c](C(=[*])[*]):[c]:1O	0.352	7 out of 7
FCFP_10	-1272709286	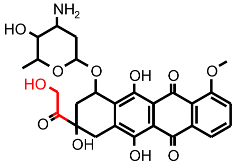 [*]NCC	0.285	234 out of 266
FCFP_10	1070061035	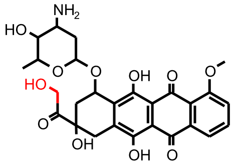 [*]S(=[*])(=[*])N	0.239	284 out of 338

Top Features for negative contribution

Fingerprint	Bit/Smiles	Feature Structure	Score	Moderate_Severe in training set
FCFP_10	979221427	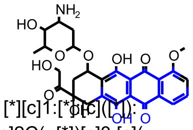 [*][c]1:[*]:[c]([*]):[c]2C(=[*])[c]3:[c]([*]):[*]:[cH]:[cH]:[c]:3C(=O)[c]:2:[c]:1O	-1.6	0 out of 6
FCFP_10	-22686407	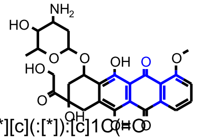 [*][c](:[*]):[c]1C(=O)[c](:[c]([*]):[*]):[c](:[*])[*][c]:1:[*]	-1.5	7 out of 53

FCFP_10	-1549669478	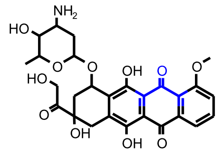 [*]:[c](:[*])C(=O)[c](:[*]):[*]	-1.5	7 out of 53

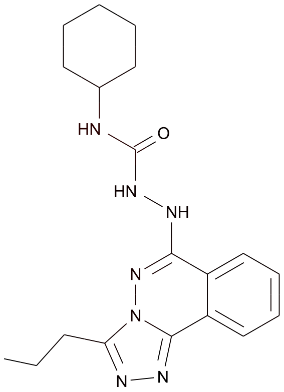

$C_{19}H_{25}N_7O$
Molecular Weight: 367.44809
ALogP: 3.681
Rotatable Bonds: 5
Acceptors: 5
Donors: 3

Model Prediction

Prediction: Irritant
Probability: 1
Enrichment: 1.18
Bayesian Score: 1.39
Mahalanobis Distance: 9.4
Mahalanobis Distance p-value: 0.302

Prediction: Positive if the Bayesian score is above the estimated best cutoff value from minimizing the false positive and false negative rate.
Probability: The esimated probability that the sample is in the positive category. This assumes that the Bayesian score follows a normal distribution and is different from the prediction using a cutoff.
Enrichment: An estimate of enrichment, that is, the increased likelihood (versus random) of this sample being in the category.
Bayesian Score: The standard Laplacian-modified Bayesian score.
Mahalanobis Distance: The Mahalanobis distance (MD) is the distance to the center of the training data. The larger the MD, the less trustworthy the prediction.
Mahalanobis Distance p-value: The p-value gives the fraction of training data with an MD greater than or equal to the one for the given sample, assuming normally distributed data. The smaller the p-value, the less trustworthy the prediciton. For highly non-normal X properties (e.g., fingerprints), the MD p-value is wildly inaccurate.

Structural Similar Compounds

Name	Disperse Black 9	ANTHRAQUINONE; 1-((2-HYDROXYETHYL)AMINO)-4-(METHYLAMINO)-	METHANE;TRIS(4-AMINOPHENYL)-
Structure	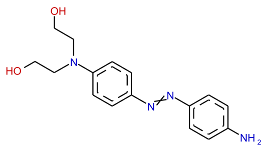	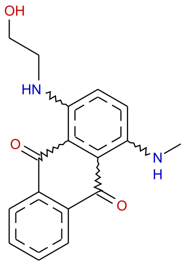	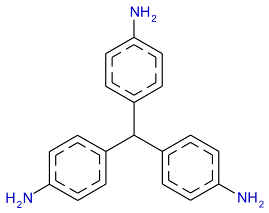
Actual Endpoint	Non-Irritant	Irritant	Irritant
Predicted Endpoint	Non-Irritant	Irritant	Irritant
Distance	0.668	0.673	0.720
Reference	J. Am. Coll. Toxicol. 5(3):205;1986	28ZPAK 245;72	28ZPAK-;73;72

Model Applicability

Unknown features are fingerprint features in the query molecule, but not found or appearing too infrequently in the training set.

1. All properties and OPS components are within expected ranges.
2. Unknown FCFP_2 feature: -1151884458: [*]N[c](:n:[*]):[c](:[*]):[*]
3. Unknown FCFP_2 feature: -1564473960: [*]:n1:[*]:[*]:n:[c]:1:[c](:[*]):[*]
4. Unknown FCFP_2 feature: -885461129: [*]NNC(=[*])[*]

Feature Contribution

Top features for positive contribution

Fingerprint	Bit/Smiles	Feature Structure	Score	Irritant in training set
FCFP_12	17	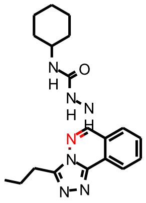 [*]:n:[*]	0.189	48 out of 49

FCFP_12	-885550502	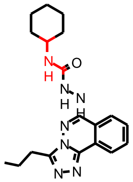 [*]C([*])NC(=[*])[*]	0.18	64 out of 66
FCFP_12	4427049	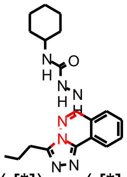 [*][c](:[*]):n:n(:[*]):[*]	0.167	4 out of 4
**Top Features for negative contribution**				
**Fingerprint**	**Bit/Smiles**	**Feature Structure**	**Score**	**Irritant in training set**
FCFP_12	1175638033	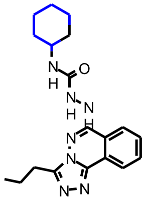 [*]C1[*]CCCC1	-0.133	207 out of 293
FCFP_12	-1525101452	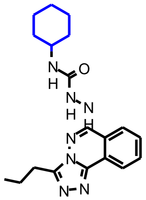 [*]C1CCCCC1	-0.127	108 out of 152
FCFP_12	1	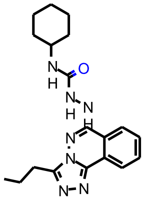 [*]=O	0	872 out of 1051

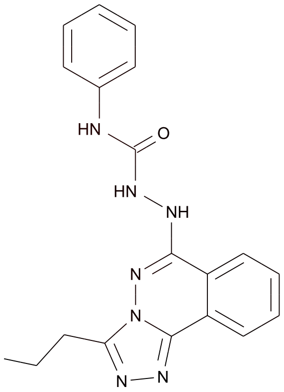

$C_{19}H_{19}N_7O$
Molecular Weight: 361.40046
ALogP: 3.399
Rotatable Bonds: 5
Acceptors: 5
Donors: 3

Structural Similar Compounds

Name	ANTHRAQUINONE; 1-((2-HYDROXYETHYL)AMINO)-4-(METHYLAMINO)-	Disperse Black 9	METHANE;TRIS(4-AMINOPHENYL)-
Structure	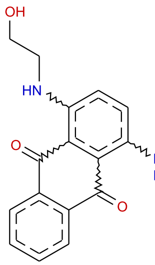	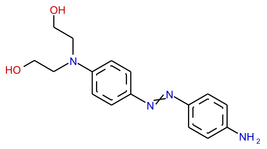	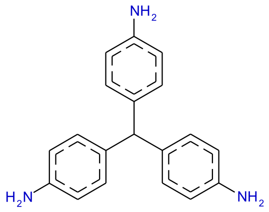
Actual Endpoint	Irritant	Non-Irritant	Irritant
Predicted Endpoint	Irritant	Non-Irritant	Irritant
Distance	0.636	0.639	0.699
Reference	28ZPAK 245;72	J. Am. Coll. Toxicol. 5(3):205;1986	28ZPAK-;73;72

Model Prediction

Prediction: Irritant
Probability: 1
Enrichment: 1.18
Bayesian Score: 1.51
Mahalanobis Distance: 6.14
Mahalanobis Distance p-value: 1

Prediction: Positive if the Bayesian score is above the estimated best cutoff value from minimizing the false positive and false negative rate.
Probability: The esimated probability that the sample is in the positive category. This assumes that the Bayesian score follows a normal distribution and is different from the prediction using a cutoff.
Enrichment: An estimate of enrichment, that is, the increased likelihood (versus random) of this sample being in the category.
Bayesian Score: The standard Laplacian-modified Bayesian score.
Mahalanobis Distance: The Mahalanobis distance (MD) is the distance to the center of the training data. The larger the MD, the less trustworthy the prediction.
Mahalanobis Distance p-value: The p-value gives the fraction of training data with an MD greater than or equal to the one for the given sample, assuming normally distributed data. The smaller the p-value, the less trustworthy the prediciton. For highly non-normal X properties (e.g., fingerprints), the MD p-value is wildly inaccurate.

Model Applicability

Unknown features are fingerprint features in the query molecule, but not found or appearing too infrequently in the training set.

1. All properties and OPS components are within expected ranges.
2. Unknown FCFP_2 feature: -1151884458: [*]N[c](:n:[*]):[c](:[*]):[*]
3. Unknown FCFP_2 feature: -1564473960: [*]:n1:[*]:[*]:n:[c]:1:[c](:[*]):[*]
4. Unknown FCFP_2 feature: -885461129: [*]NNC(=[*])[*]

Feature Contribution

Top features for positive contribution

Fingerprint	Bit/Smiles	Feature Structure	Score	Irritant in training set
FCFP_12	17	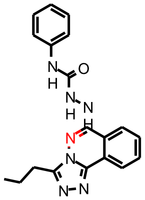 [*]:n:[*]	0.189	48 out of 49

FCFP_12	4427049	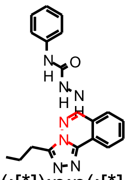 [*][c](:[*]):n:n(:[*]):[*]	0.167	4 out of 4
FCFP_12	-1539162406	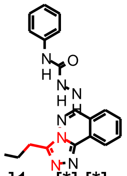 [*]C[c]1:n:[*]:[*]:n:1:[*]	0.156	3 out of 3
**Top Features for negative contribution**				
**Fingerprint**	**Bit/Smiles**	**Feature Structure**	**Score**	**Irritant in training set**
FCFP_12	-2093839777	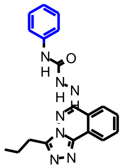 [*][c]1:[cH]:[cH]:[cH]:[cH]:[cH]:1	0	94 out of 121
FCFP_12	-792685140	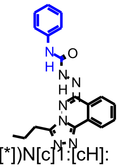 [*]C(=[*])N[c]1:[cH]:[cH]:[cH]:[cH]:[cH]:1	0	5 out of 6
FCFP_12	0	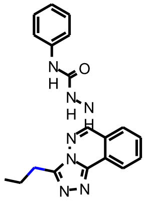 [*]C[*]	0	1184 out of 1397

TOPKAT_Ocular_Irritancy_None_vs_Irritant

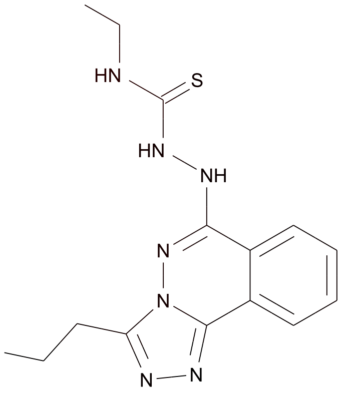

$C_{15}H_{19}N_7S$
Molecular Weight: 329.42325
ALogP: 3.388
Rotatable Bonds: 7
Acceptors: 5
Donors: 3

Structural Similar Compounds

Name	Disperse Black 9	ANTHRAQUINONE; 1-((2-HYDROXYETHYL)AMINO)-4-(METHYLAMINO)-	2-NAPHTHALENESULFONIC ACID; 4-HYDROXY-7-(METHYLAMINO)-
Structure	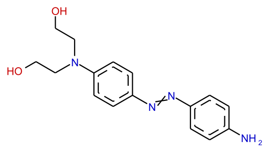	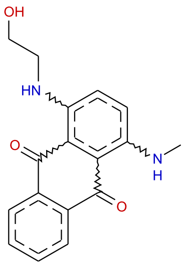	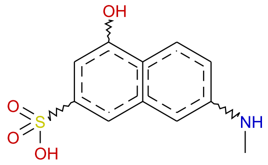
Actual Endpoint	Non-Irritant	Irritant	Irritant
Predicted Endpoint	Non-Irritant	Irritant	Irritant
Distance	0.616	0.656	0.724
Reference	J. Am. Coll. Toxicol. 5(3):205;1986	28ZPAK 245;72	28ZPAK 190;72

Model Prediction

Prediction: Irritant
Probability: 1
Enrichment: 1.18
Bayesian Score: 1.45
Mahalanobis Distance: 6.86
Mahalanobis Distance p-value: 0.999

Prediction: Positive if the Bayesian score is above the estimated best cutoff value from minimizing the false positive and false negative rate.
Probability: The esimated probability that the sample is in the positive category. This assumes that the Bayesian score follows a normal distribution and is different from the prediction using a cutoff.
Enrichment: An estimate of enrichment, that is, the increased likelihood (versus random) of this sample being in the category.
Bayesian Score: The standard Laplacian-modified Bayesian score.
Mahalanobis Distance: The Mahalanobis distance (MD) is the distance to the center of the training data. The larger the MD, the less trustworthy the prediction.
Mahalanobis Distance p-value: The p-value gives the fraction of training data with an MD greater than or equal to the one for the given sample, assuming normally distributed data. The smaller the p-value, the less trustworthy the prediciton. For highly non-normal X properties (e.g., fingerprints), the MD p-value is wildly inaccurate.

Model Applicability

Unknown features are fingerprint features in the query molecule, but not found or appearing too infrequently in the training set.

1. All properties and OPS components are within expected ranges.
2. Unknown FCFP_2 feature: -1151884458: [*]N[c](:n:[*]):[c](:[*]):[*]
3. Unknown FCFP_2 feature: -1564473960: [*]:n1:[*]:[*]:n:[c]:1:[c](:[*]):[*]
4. Unknown FCFP_2 feature: -885461129: [*]NNC(=[*])[*]

Feature Contribution

Top features for positive contribution

Fingerprint	Bit/Smiles	Feature Structure	Score	Irritant in training set
FCFP_12	17	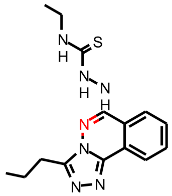 [*]:n:[*]	0.189	48 out of 49

FCFP_12	-885550502	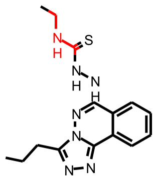 [*]C([*])NC(=[*])[*]	0.18	64 out of 66
FCFP_12	-1939253119	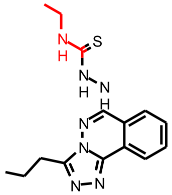 [*]C(=[*])NCC	0.175	5 out of 5

Top Features for negative contribution

Fingerprint	Bit/Smiles	Feature Structure	Score	Irritant in training set
FCFP_12	-1272798659	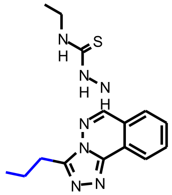 [*]CCC	0	517 out of 643
FCFP_12	307419094	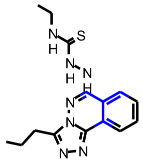 [*][c](:[*]):[c](:[cH]:[*]):[c](:[*]):[*]	0	43 out of 52
FCFP_12	1	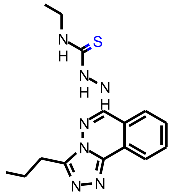 [*]=O	0	872 out of 1051

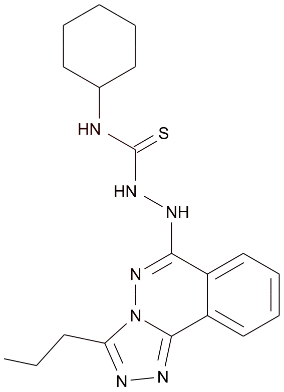

$C_{19}H_{25}N_7S$
Molecular Weight: 383.51369
ALogP: 4.898
Rotatable Bonds: 7
Acceptors: 5
Donors: 3

Model Prediction

Prediction: Irritant
Probability: 1
Enrichment: 1.18
Bayesian Score: 1.32
Mahalanobis Distance: 9.32
Mahalanobis Distance p-value: 0.342

Prediction: Positive if the Bayesian score is above the estimated best cutoff value from minimizing the false positive and false negative rate.
Probability: The esimated probability that the sample is in the positive category. This assumes that the Bayesian score follows a normal distribution and is different from the prediction using a cutoff.
Enrichment: An estimate of enrichment, that is, the increased likelihood (versus random) of this sample being in the category.
Bayesian Score: The standard Laplacian-modified Bayesian score.
Mahalanobis Distance: The Mahalanobis distance (MD) is the distance to the center of the training data. The larger the MD, the less trustworthy the prediction.
Mahalanobis Distance p-value: The p-value gives the fraction of training data with an MD greater than or equal to the one for the given sample, assuming normally distributed data. The smaller the p-value, the less trustworthy the prediciton. For highly non-normal X properties (e.g., fingerprints), the MD p-value is wildly inaccurate.

Structural Similar Compounds

Name	4;4'-DIAMINO-1;1'-DIANTHRIMIDE	Disperse Black 9	BENZANILIDE;2';2'''-DITHIOBIS-
Structure	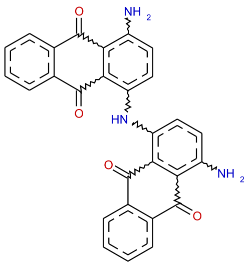	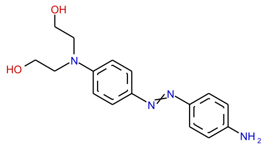	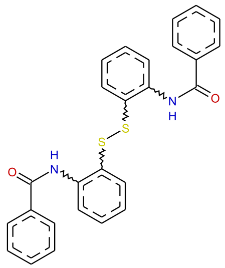
Actual Endpoint	Irritant	Non-Irritant	Non-Irritant
Predicted Endpoint	Irritant	Non-Irritant	Non-Irritant
Distance	0.747	0.748	0.774
Reference	28ZPAK-;125;72	J. Am. Coll. Toxicol. 5(3):205;1986	28ZPAK-;173;72

Model Applicability

Unknown features are fingerprint features in the query molecule, but not found or appearing too infrequently in the training set.

1. All properties and OPS components are within expected ranges.
2. Unknown FCFP_2 feature: -1151884458: [*]N[c](:n:[*]):[c](:[*]):[*]
3. Unknown FCFP_2 feature: -1564473960: [*]:n1:[*]:[*]:n:[c]:1:[c](:[*]):[*]
4. Unknown FCFP_2 feature: -885461129: [*]NNC(=[*])[*]

Feature Contribution

Top features for positive contribution				
Fingerprint	Bit/Smiles	Feature Structure	Score	Irritant in training set
FCFP_12	17	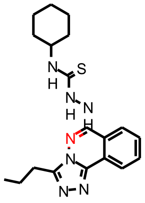 [*]:n:[*]	0.189	48 out of 49

FCFP_12	-885550502	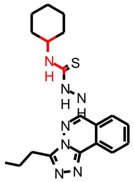 [*]C([*])NC(=[*])[*]	0.18	64 out of 66
FCFP_12	4427049	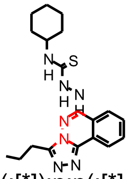 [*][c](:[*]):n:n(:[*]):[*]	0.167	4 out of 4
**Top Features for negative contribution**				
**Fingerprint**	**Bit/Smiles**	**Feature Structure**	**Score**	**Irritant in training set**
FCFP_12	1175638033	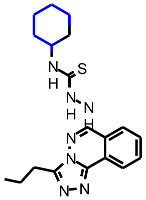 [*]C1[*]CCCC1	-0.133	207 out of 293
FCFP_12	-1525101452	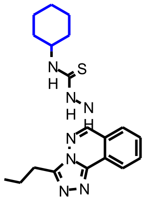 [*]C1CCCCC1	-0.127	108 out of 152
FCFP_12	1	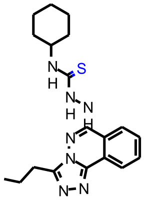 [*]=O	0	872 out of 1051

9a TOPKAT_Ocular_Irritancy_None_vs_Irritant

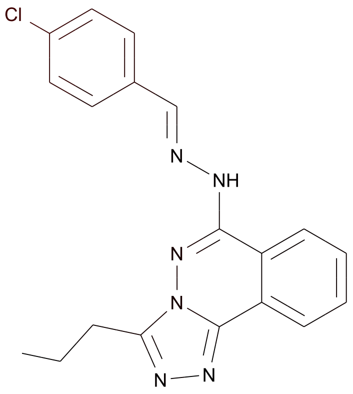

$C_{19}H_{17}ClN_6$
Molecular Weight: 364.83147
ALogP: 4.967
Rotatable Bonds: 5
Acceptors: 5
Donors: 1

Model Prediction

Prediction: Irritant
Probability: 1
Enrichment: 1.18
Bayesian Score: 1.81
Mahalanobis Distance: 6.68
Mahalanobis Distance p-value: 1

Prediction: Positive if the Bayesian score is above the estimated best cutoff value from minimizing the false positive and false negative rate.
Probability: The esimated probability that the sample is in the positive category. This assumes that the Bayesian score follows a normal distribution and is different from the prediction using a cutoff.
Enrichment: An estimate of enrichment, that is, the increased likelihood (versus random) of this sample being in the category.
Bayesian Score: The standard Laplacian-modified Bayesian score.
Mahalanobis Distance: The Mahalanobis distance (MD) is the distance to the center of the training data. The larger the MD, the less trustworthy the prediction.
Mahalanobis Distance p-value: The p-value gives the fraction of training data with an MD greater than or equal to the one for the given sample, assuming normally distributed data. The smaller the p-value, the less trustworthy the prediciton. For highly non-normal X properties (e.g., fingerprints), the MD p-value is wildly inaccurate.

Structural Similar Compounds

Name	1-BENZOYLAMINO-4-METHOXY-5-CHLORANTHRAQUINONE	ANTHRAQUINONE; 1;1'-IMINODI-	BENZILIC ACID; 4;4'-DICHLORO-; ISOPROPYL ESTER
Structure	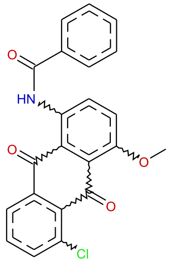	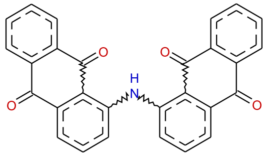	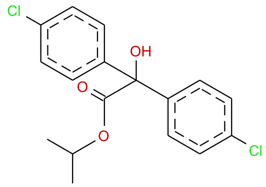
Actual Endpoint	Irritant	Irritant	Irritant
Predicted Endpoint	Irritant	Irritant	Irritant
Distance	0.593	0.607	0.633
Reference	28ZPAK-;90;72	28ZPAK-;125;72	CIGET* -;-;77

Model Applicability

Unknown features are fingerprint features in the query molecule, but not found or appearing too infrequently in the training set.

1. All properties and OPS components are within expected ranges.
2. Unknown FCFP_2 feature: -1151884458: [*]N[c](:n:[*]):[c](:[*]):[*]
3. Unknown FCFP_2 feature: -1564473960: [*]:n1:[*]:[*]:n:[c]:1:[c](:[*]):[*]
4. Unknown FCFP_2 feature: 1294285001: [*]=NN[c](:[*]):[*]
5. Unknown FCFP_2 feature: 581019816: [*]N\N=C\[*]

Feature Contribution

Top features for positive contribution				
**Fingerprint**	**Bit/Smiles**	**Feature Structure**	**Score**	**Irritant in training set**
FCFP_12	-1508180856	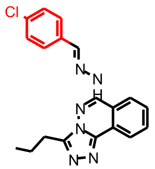 [*][c]1:[cH]:[cH]:[c](Cl):[cH]:[cH]:1	0.2	17 out of 17

FCFP_12	17	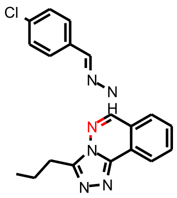 [*]:n:[*]	0.189	48 out of 49
FCFP_12	-149636017	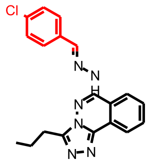 [*]=C[c]1:[cH]:[cH]:[c](Cl):[cH]:[cH]:1	0.184	7 out of 7

Top Features for negative contribution

Fingerprint	Bit/Smiles	Feature Structure	Score	Irritant in training set
FCFP_12	136597326	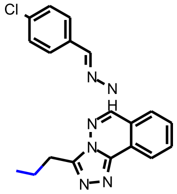 [*]CC	0	612 out of 753
FCFP_12	203677720	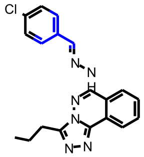 [*]=C[c](:[cH]:[*]):[cH]:[*]	0	319 out of 382
FCFP_12	-1272798659	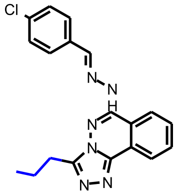 [*]CCC	0	517 out of 643

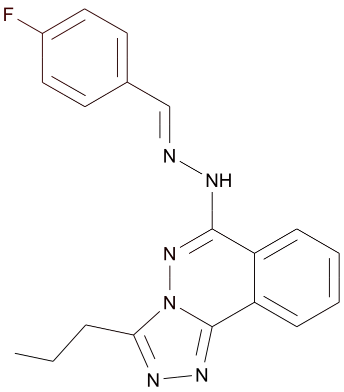

$C_{19}H_{17}FN_6$
Molecular Weight: 348.37688
ALogP: 4.508
Rotatable Bonds: 5
Acceptors: 5
Donors: 1

Model Prediction

Prediction: Irritant
Probability: 1
Enrichment: 1.18
Bayesian Score: 2.2
Mahalanobis Distance: 6.6
Mahalanobis Distance p-value: 1

Prediction: Positive if the Bayesian score is above the estimated best cutoff value from minimizing the false positive and false negative rate.
Probability: The esimated probability that the sample is in the positive category. This assumes that the Bayesian score follows a normal distribution and is different from the prediction using a cutoff.
Enrichment: An estimate of enrichment, that is, the increased likelihood (versus random) of this sample being in the category.
Bayesian Score: The standard Laplacian-modified Bayesian score.
Mahalanobis Distance: The Mahalanobis distance (MD) is the distance to the center of the training data. The larger the MD, the less trustworthy the prediction.
Mahalanobis Distance p-value: The p-value gives the fraction of training data with an MD greater than or equal to the one for the given sample, assuming normally distributed data. The smaller the p-value, the less trustworthy the prediciton. For highly non-normal X properties (e.g., fingerprints), the MD p-value is wildly inaccurate.

Structural Similar Compounds

Name	1-BENZOYLAMINO-4-METHOXY-5-CHLORANTHRAQUINONE	BENZILIC ACID; 4;4'-DICHLORO-; ISOPROPYL ESTER	ANTHRAQUINONE; 1;1'-IMINODI-
Structure	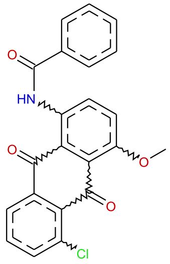	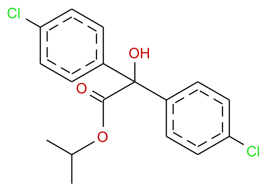	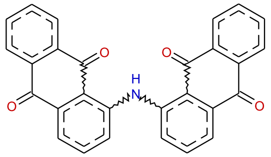
Actual Endpoint	Irritant	Irritant	Irritant
Predicted Endpoint	Irritant	Irritant	Irritant
Distance	0.590	0.631	0.641
Reference	28ZPAK-;90;72	CIGET* -;-;77	28ZPAK-;125;72

Model Applicability

Unknown features are fingerprint features in the query molecule, but not found or appearing too infrequently in the training set.

1. All properties and OPS components are within expected ranges.
2. Unknown FCFP_2 feature: -1151884458: [*]N[c](:n:[*]):[c](:[*]):[*]
3. Unknown FCFP_2 feature: -1564473960: [*]:n1:[*]:[*]:n:[c]:1:[c](:[*]):[*]
4. Unknown FCFP_2 feature: 1294285001: [*]=NN[c](:[*]):[*]
5. Unknown FCFP_2 feature: 581019816: [*]N\N=C\[*]

Feature Contribution

Top features for positive contribution

Fingerprint	Bit/Smiles	Feature Structure	Score	Irritant in training set
FCFP_12	-1508180856	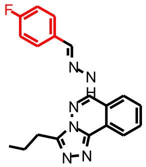 [*][c]1:[cH]:[cH]:[c](Cl):[cH]:[cH]:1	0.2	17 out of 17

FCFP_12	17	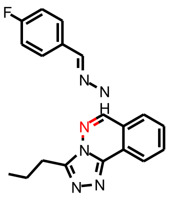 [*]:n:[*]	0.189	48 out of 49
FCFP_12	-149636017	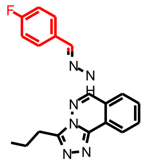 [*]=C[c]1:[cH]:[cH]:[c](Cl):[cH]:[cH]:1	0.184	7 out of 7

Top Features for negative contribution

Fingerprint	Bit/Smiles	Feature Structure	Score	Irritant in training set
FCFP_12	-453677277	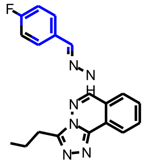 [*][c]1:[*]:[cH]:[c](C=[*]):[cH]:[cH]:1	0	264 out of 323
FCFP_12	991735244	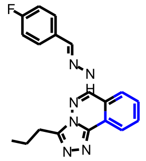 [*]:[c]1:[*]:[cH]:[cH]:[cH]:[cH]:1	0	237 out of 291
FCFP_12	136597326	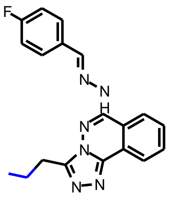 [*]CC	0	612 out of 753

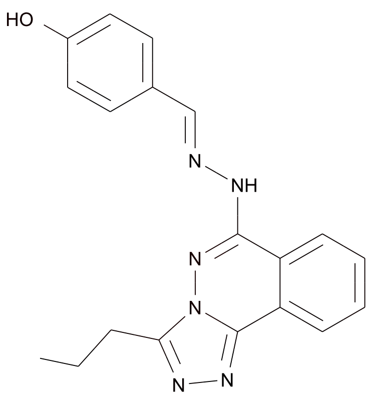

$C_{19}H_{18}N_6O$

Molecular Weight: 346.38582

ALogP: 4.061

Rotatable Bonds: 5

Acceptors: 6

Donors: 2

Structural Similar Compounds

Name	ANTHRAQUINONE; 1-AMINO-4-HYDROXY-2-PHENOXY-	2;2';-Dihydroxy-4;4'-dimethoxybenzophenone	s-TRIAZINE; 2;4-BIS(ISOPROPYLAMINO0-6-(METHYLTHIO)-
Structure	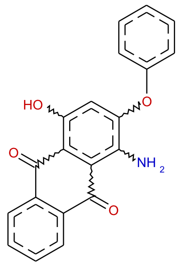	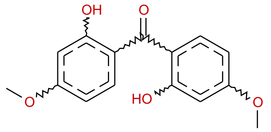	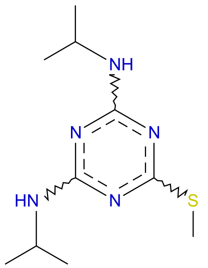
Actual Endpoint	Irritant	Non-Irritant	Irritant
Predicted Endpoint	Irritant	Non-Irritant	Irritant
Distance	0.607	0.655	0.679
Reference	28ZPAK 239;72	J. Am. Coll. Toxicol. 2(5):35;1983	CIGET* -;-;77

Model Prediction

Prediction: Irritant

Probability: 1

Enrichment: 1.18

Bayesian Score: 1.27

Mahalanobis Distance: 6.78

Mahalanobis Distance p-value: 0.999

Prediction: Positive if the Bayesian score is above the estimated best cutoff value from minimizing the false positive and false negative rate.
Probability: The esimated probability that the sample is in the positive category. This assumes that the Bayesian score follows a normal distribution and is different from the prediction using a cutoff.
Enrichment: An estimate of enrichment, that is, the increased likelihood (versus random) of this sample being in the category.
Bayesian Score: The standard Laplacian-modified Bayesian score.
Mahalanobis Distance: The Mahalanobis distance (MD) is the distance to the center of the training data. The larger the MD, the less trustworthy the prediction.
Mahalanobis Distance p-value: The p-value gives the fraction of training data with an MD greater than or equal to the one for the given sample, assuming normally distributed data. The smaller the p-value, the less trustworthy the prediciton. For highly non-normal X properties (e.g., fingerprints), the MD p-value is wildly inaccurate.

Model Applicability

Unknown features are fingerprint features in the query molecule, but not found or appearing too infrequently in the training set.

1. All properties and OPS components are within expected ranges.
2. Unknown FCFP_2 feature: -1151884458: [*]N[c](:n:[*]):[c](:[*]):[*]
3. Unknown FCFP_2 feature: -1564473960: [*]:n1:[*]:[*]:n:[c]:1:[c](:[*]):[*]
4. Unknown FCFP_2 feature: 1294285001: [*]=NN[c](:[*]):[*]
5. Unknown FCFP_2 feature: 581019816: [*]N\N=C\[*]

Feature Contribution

Top features for positive contribution

Fingerprint	Bit/Smiles	Feature Structure	Score	Irritant in training set
FCFP_12	17	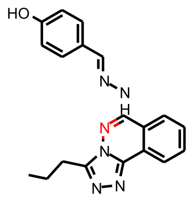 [*]:n:[*]	0.189	48 out of 49

FCFP_12	4427049	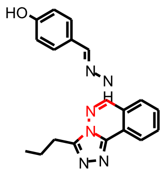 [*][c](:[*]):n:n(:[*]):[*]	0.167	4 out of 4
FCFP_12	-1539162406	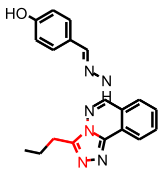 [*]C[c]1:n:[*]:[*]:n:1:[*]	0.156	3 out of 3
**Top Features for negative contribution**				
**Fingerprint**	**Bit/Smiles**	**Feature Structure**	**Score**	**Irritant in training set**
FCFP_12	-1066794953	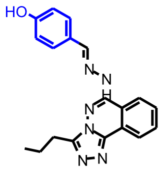 [*][c]1:[cH]:[cH]:[c](O):[cH]:[cH]:1	-0.0509	13 out of 17
FCFP_12	-158888774	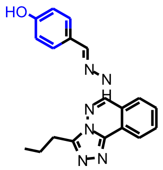 O[c]1:[cH]:[cH]:[*]:[cH]:[cH]:1	0	25 out of 30
FCFP_12	-453677277	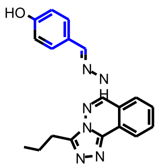 [*][c]1:[*]:[cH]:[c](C=[*]):[cH]:[cH]:1	0	264 out of 323

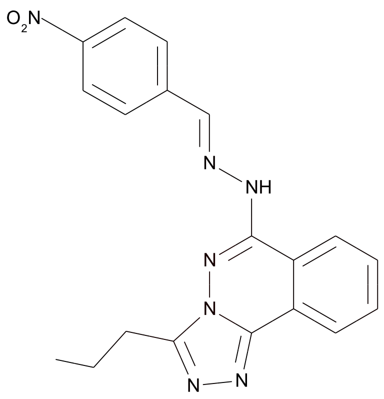

$C_{19}H_{17}N_7O_2$

Molecular Weight: 375.38397

ALogP: 4.197

Rotatable Bonds: 6

Acceptors: 7

Donors: 1

Structural Similar Compounds

Name	COLCHICINE	1;4-PENTADIENE-3-ONE;1;5-BIS(4-(2;3-DIDEHYDROTRIAZIRIDINYL)PHENYL)-	ANTHRAQUINONE; 1;1'-IMINODI-
Structure	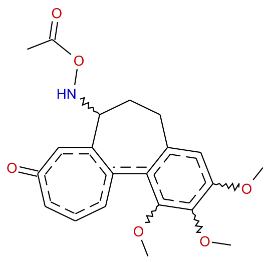	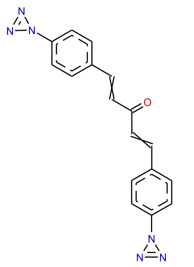	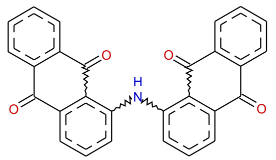
Actual Endpoint	Irritant	Irritant	Irritant
Predicted Endpoint	Irritant	Irritant	Irritant
Distance	0.692	0.741	0.753
Reference	AJOPAA 31;837;48	28ZPAK-;123;72	28ZPAK-;125;72

Model Prediction

Prediction: Irritant

Probability: 1

Enrichment: 1.18

Bayesian Score: 1.09

Mahalanobis Distance: 7.36

Mahalanobis Distance p-value: 0.991

Prediction: Positive if the Bayesian score is above the estimated best cutoff value from minimizing the false positive and false negative rate.
Probability: The esimated probability that the sample is in the positive category. This assumes that the Bayesian score follows a normal distribution and is different from the prediction using a cutoff.
Enrichment: An estimate of enrichment, that is, the increased likelihood (versus random) of this sample being in the category.
Bayesian Score: The standard Laplacian-modified Bayesian score.
Mahalanobis Distance: The Mahalanobis distance (MD) is the distance to the center of the training data. The larger the MD, the less trustworthy the prediction.
Mahalanobis Distance p-value: The p-value gives the fraction of training data with an MD greater than or equal to the one for the given sample, assuming normally distributed data. The smaller the p-value, the less trustworthy the prediciton. For highly non-normal X properties (e.g., fingerprints), the MD p-value is wildly inaccurate.

Model Applicability

Unknown features are fingerprint features in the query molecule, but not found or appearing too infrequently in the training set.

1. All properties and OPS components are within expected ranges.
2. Unknown FCFP_2 feature: -1151884458: [*]N[c](:n:[*]):[c](:[*]):[*]
3. Unknown FCFP_2 feature: -1564473960: [*]:n1:[*]:[*]:n:[c]:1:[c](:[*]):[*]
4. Unknown FCFP_2 feature: 1294285001: [*]=NN[c](:[*]):[*]
5. Unknown FCFP_2 feature: 581019816: [*]N\N=C\[*]
6. Unknown FCFP_2 feature: -828984032: [*][N+](=[*])[c](:[cH]:[*]):[cH]:[*]
7. Unknown FCFP_2 feature: -1338588315: [*]:[c](:[*])[N+](=O)[O-]
8. Unknown FCFP_2 feature: 1872392852: [*][N+](=O)[*]
9. Unknown FCFP_2 feature: 260476081: [*][N+](=[*])[O-]

Feature Contribution

Top features for positive contribution

Fingerprint	Bit/Smiles	Feature Structure	Score	Irritant in training set

FCFP_12	17	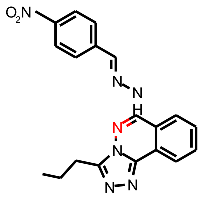 [*]:n:[*]	0.189	48 out of 49
FCFP_12	4427049	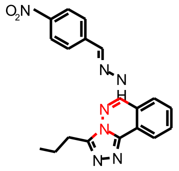 [*][c](:[*]):n:n(:[*]):[*]	0.167	4 out of 4
FCFP_12	-1539162406	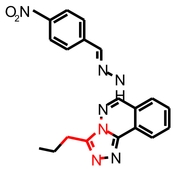 [*]C[c]1:n:[*]:[*]:n: 1:[*]	0.156	3 out of 3
**Top Features for negative contribution**				
**Fingerprint**	**Bit/Smiles**	**Feature Structure**	**Score**	**Irritant in training set**
FCFP_12	8	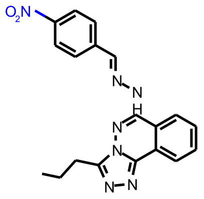 [*][N+](=[*])[*]	-0.0561	3 out of 4
FCFP_12	991735244	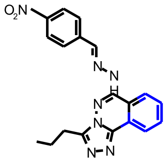 [*]:[c]1:[*]:[cH]:[cH]:[cH]:[cH]:1	0	237 out of 291

FCFP_12	136597326	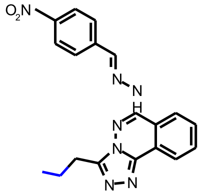 [*]CC	0	612 out of 753

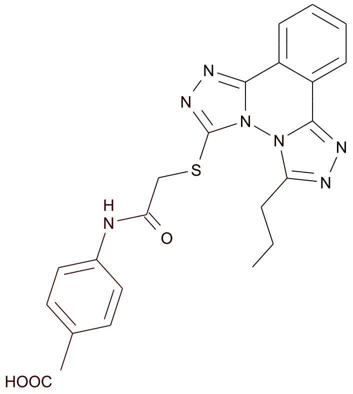

$C_{22}H_{19}N_7O_3S$

Molecular Weight: 461.49635

ALogP: 3.42

Rotatable Bonds: 7

Acceptors: 8

Donors: 2

Structural Similar Compounds

Name	4;4'-DIAMINO-1;1'-DIANTHRIMIDE	COLCHICINE	ANTHRAQUINONE; 1-AMINO-4-HYDROXY-2-PHENOXY-
Structure	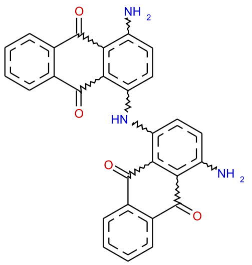	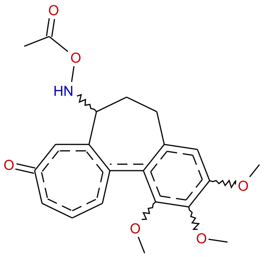	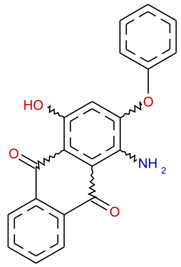
Actual Endpoint	Irritant	Irritant	Irritant
Predicted Endpoint	Irritant	Irritant	Irritant
Distance	0.748	0.825	0.843
Reference	28ZPAK-;125;72	AJOPAA 31;837;48	28ZPAK 239;72

Model Applicability

Unknown features are fingerprint features in the query molecule, but not found or appearing too infrequently in the training set.

1. All properties and OPS components are within expected ranges.
2. Unknown FCFP_2 feature: -1564473960: [*]:n1:[*]:[*]:n:[c]:1:[c](:[*]):[*]

Model Prediction

Prediction: Irritant

Probability: 1

Enrichment: 1.18

Bayesian Score: 2.93

Mahalanobis Distance: 7.78

Mahalanobis Distance p-value: 0.961

Prediction: Positive if the Bayesian score is above the estimated best cutoff value from minimizing the false positive and false negative rate.
Probability: The esimated probability that the sample is in the positive category. This assumes that the Bayesian score follows a normal distribution and is different from the prediction using a cutoff.
Enrichment: An estimate of enrichment, that is, the increased likelihood (versus random) of this sample being in the category.
Bayesian Score: The standard Laplacian-modified Bayesian score.
Mahalanobis Distance: The Mahalanobis distance (MD) is the distance to the center of the training data. The larger the MD, the less trustworthy the prediction.
Mahalanobis Distance p-value: The p-value gives the fraction of training data with an MD greater than or equal to the one for the given sample, assuming normally distributed data. The smaller the p-value, the less trustworthy the prediciton. For highly non-normal X properties (e.g., fingerprints), the MD p-value is wildly inaccurate.

Feature Contribution

Top features for positive contribution

Fingerprint	Bit/Smiles	Feature Structure	Score	Irritant in training set
FCFP_12	-1549222613	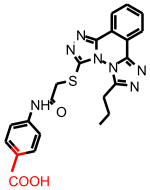 [*]:[c](:[*])C(=O)O	0.198	14 out of 14

FCFP_12	1175665944	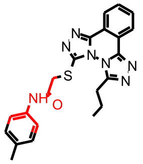<br><br>[*]CC(=O)N[c](:[cH]:[*]):[cH]:[*]	0.198	14 out of 14
FCFP_12	937923569	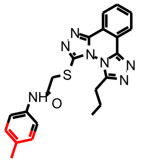<br>[*]:[cH]:[c](:[cH]:[*])C(=O)O	0.198	14 out of 14

Top Features for negative contribution

Fingerprint	Bit/Smiles	Feature Structure	Score	Irritant in training set
FCFP_12	-773983804	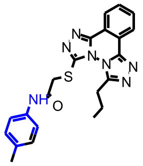<br>[*]N[c]1:[cH]:[*]:[cH]:[cH]:[cH]:1	0	102 out of 121
FCFP_12	-453677277	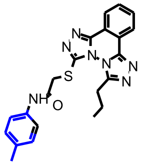<br>[*][c]1:[*]:[cH]:[c](C=[*]):[cH]:[cH]:1	0	264 out of 323
FCFP_12	1872154524	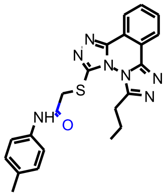<br><br>[*]C(=O)[*]	0	563 out of 690

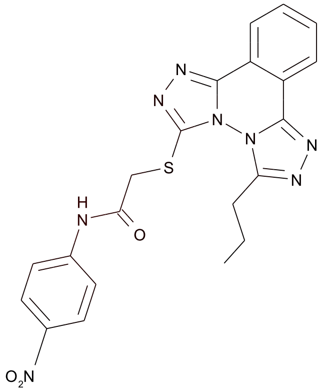

$C_{21}H_{18}N_8O_3S$
Molecular Weight: 462.48441
ALogP: 3.685
Rotatable Bonds: 7
Acceptors: 8
Donors: 1

Structural Similar Compounds

Name	COLCHICINE	1;8;9-ANTHRACENETRIOL; TRIACETATE	1;4-PENTADIENE-3-ONE;1;5-BIS(4-(2;3-DIDEHYDROTRIAZIRIDINYL)PHENYL)-
Structure	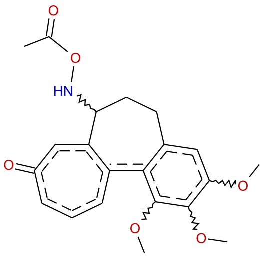	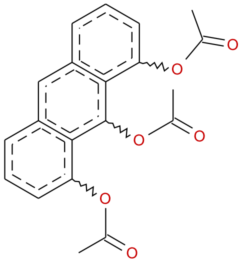	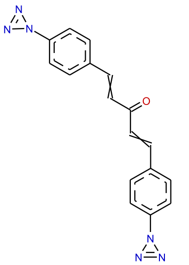
Actual Endpoint	Irritant	Irritant	Irritant
Predicted Endpoint	Irritant	Irritant	Irritant
Distance	0.772	0.866	0.872
Reference	AJOPAA 31;837;48	BJOPAL 53;819;69	28ZPAK-;123;72

Model Prediction

Prediction: Irritant

Probability: 1

Enrichment: 1.18

Bayesian Score: 1.94

Mahalanobis Distance: 8.14

Mahalanobis Distance p-value: 0.892

Prediction: Positive if the Bayesian score is above the estimated best cutoff value from minimizing the false positive and false negative rate.
Probability: The esimated probability that the sample is in the positive category. This assumes that the Bayesian score follows a normal distribution and is different from the prediction using a cutoff.
Enrichment: An estimate of enrichment, that is, the increased likelihood (versus random) of this sample being in the category.
Bayesian Score: The standard Laplacian-modified Bayesian score.
Mahalanobis Distance: The Mahalanobis distance (MD) is the distance to the center of the training data. The larger the MD, the less trustworthy the prediction.
Mahalanobis Distance p-value: The p-value gives the fraction of training data with an MD greater than or equal to the one for the given sample, assuming normally distributed data. The smaller the p-value, the less trustworthy the prediciton. For highly non-normal X properties (e.g., fingerprints), the MD p-value is wildly inaccurate.

Model Applicability

Unknown features are fingerprint features in the query molecule, but not found or appearing too infrequently in the training set.

1. All properties and OPS components are within expected ranges.
2. Unknown FCFP_2 feature: -1564473960: [*]:n1:[*]:[*]:n:[c]:1:[c](:[*]):[*]
3. Unknown FCFP_2 feature: -828984032: [*][N+](=[*])[c](:[cH]:[*]):[cH]:[*]
4. Unknown FCFP_2 feature: -1338588315: [*]:[c](:[*])[N+](=O)[O-]
5. Unknown FCFP_2 feature: 1872392852: [*][N+](=O)[*]
6. Unknown FCFP_2 feature: 260476081: [*][N+](=[*])[O-]

Feature Contribution

Top features for positive contribution				
Fingerprint	Bit/Smiles	Feature Structure	Score	Irritant in training set

FCFP_12	1175665944	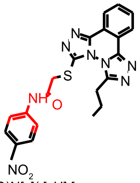 [*]CC(=O)N[c](:[cH]:[*]):[cH]:[*]	0.198	14 out of 14
FCFP_12	17	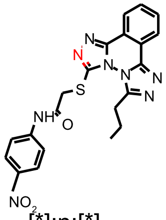 [*]:n:[*]	0.189	48 out of 49
FCFP_12	-1410079687	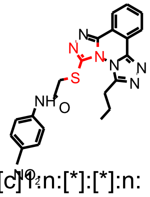 [*]S[c]1:n:[*]:[*]:n:1:[*]	0.187	8 out of 8
**Top Features for negative contribution**				
**Fingerprint**	**Bit/Smiles**	**Feature Structure**	**Score**	**Irritant in training set**
FCFP_12	8	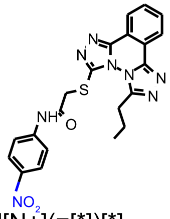 [*][N+](=[*])[*]	-0.0561	3 out of 4
FCFP_12	1	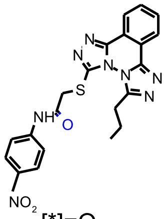 [*]=O	0	872 out of 1051

FCFP_12	0	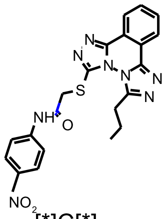 [*]C[*]	0	1184 out of 1397

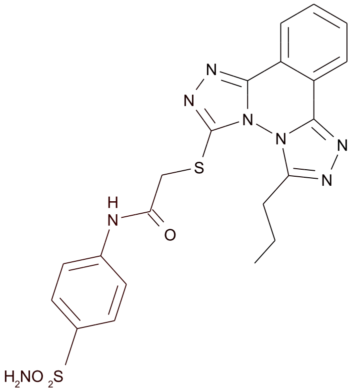

$C_{21}H_{20}N_8O_3S_2$

Molecular Weight: 496.56529

ALogP: 2.496

Rotatable Bonds: 7

Acceptors: 8

Donors: 2

Model Prediction

Prediction: Irritant

Probability: 1

Enrichment: 1.18

Bayesian Score: 2.72

Mahalanobis Distance: 8.46

Mahalanobis Distance p-value: 0.781

Prediction: Positive if the Bayesian score is above the estimated best cutoff value from minimizing the false positive and false negative rate.
Probability: The esimated probability that the sample is in the positive category. This assumes that the Bayesian score follows a normal distribution and is different from the prediction using a cutoff.
Enrichment: An estimate of enrichment, that is, the increased likelihood (versus random) of this sample being in the category.
Bayesian Score: The standard Laplacian-modified Bayesian score.
Mahalanobis Distance: The Mahalanobis distance (MD) is the distance to the center of the training data. The larger the MD, the less trustworthy the prediction.
Mahalanobis Distance p-value: The p-value gives the fraction of training data with an MD greater than or equal to the one for the given sample, assuming normally distributed data. The smaller the p-value, the less trustworthy the prediciton. For highly non-normal X properties (e.g., fingerprints), the MD p-value is wildly inaccurate.

Structural Similar Compounds

Name	2;2'-Stilbenedisulfonic acid; 4;4'-dinitro-	2;7-NAPHTHALENE DISULFONIC ACID;4-AMINO-5-HYDROXY-;P-TOLUENE SULFONATE (ESTER)	4;4'-DIAMINO-1;1'-DIANTHRIMIDE
Structure	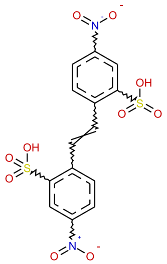	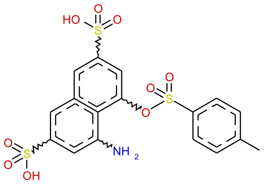	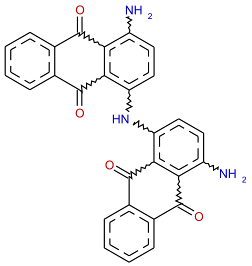
Actual Endpoint	Irritant	Irritant	Irritant
Predicted Endpoint	Irritant	Irritant	Irritant
Distance	0.783	0.786	0.793
Reference	Prehled Prumyslove Toxikologie; Organicke Latky; Marhold; J. pp 1062;86	28ZPAK-;194;72	28ZPAK-;125;72

Model Applicability

Unknown features are fingerprint features in the query molecule, but not found or appearing too infrequently in the training set.

1. All properties and OPS components are within expected ranges.
2. Unknown FCFP_2 feature: -1564473960: [*]:n1:[*]:[*]:n:[c]:1:[c](:[*]):[*]

Feature Contribution

Top features for positive contribution

Fingerprint	Bit/Smiles	Feature Structure	Score	Irritant in training set
FCFP_12	1175665944	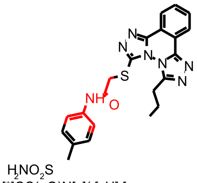 [*]CC(=O)N[c](:[cH]:[*]):[cH]:[*]	0.198	14 out of 14

FCFP_12	17	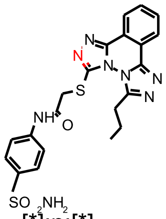 [*]:n:[*]	0.189	48 out of 49
FCFP_12	-1410079687	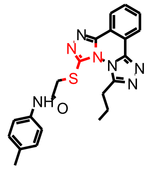 [*]S[c]1:n:[*]:[*]:n:1:[*]	0.187	8 out of 8
**Top Features for negative contribution**				
**Fingerprint**	**Bit/Smiles**	**Feature Structure**	**Score**	**Irritant in training set**
FCFP_12	1872154524	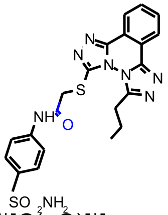 [*]C(=O)[*]	0	563 out of 690
FCFP_12	-1272768868	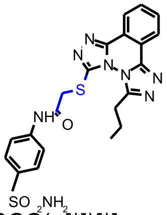 [*]SCC(=[*])[*]	0	396 out of 514
FCFP_12	-1272798659	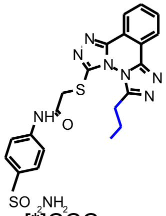 [*]CCC	0	517 out of 643

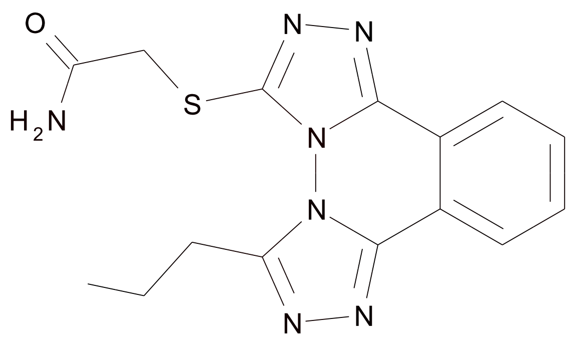

$C_{15}H_{15}N_7OS$
Molecular Weight: 341.39089
ALogP: 2.008
Rotatable Bonds: 5
Acceptors: 6
Donors: 1

Model Prediction

Prediction: Irritant
Probability: 1
Enrichment: 1.18
Bayesian Score: 1.62
Mahalanobis Distance: 7.73
Mahalanobis Distance p-value: 0.966

Prediction: Positive if the Bayesian score is above the estimated best cutoff value from minimizing the false positive and false negative rate.
Probability: The esimated probability that the sample is in the positive category. This assumes that the Bayesian score follows a normal distribution and is different from the prediction using a cutoff.
Enrichment: An estimate of enrichment, that is, the increased likelihood (versus random) of this sample being in the category.
Bayesian Score: The standard Laplacian-modified Bayesian score.
Mahalanobis Distance: The Mahalanobis distance (MD) is the distance to the center of the training data. The larger the MD, the less trustworthy the prediction.
Mahalanobis Distance p-value: The p-value gives the fraction of training data with an MD greater than or equal to the one for the given sample, assuming normally distributed data. The smaller the p-value, the less trustworthy the prediciton. For highly non-normal X properties (e.g., fingerprints), the MD p-value is wildly inaccurate.

Structural Similar Compounds

Name	2;5-DICHLORO-4(3'-METHYL-5' PYRAZOLON-1'-YL)BENZENE SULFONIC ACID	Benzenesulfonic acid; 3;5-bis(methoxycarbonyl)-; sodium salt	Benzenesulfonic acid; 2-anilino-5-nitro-
Structure	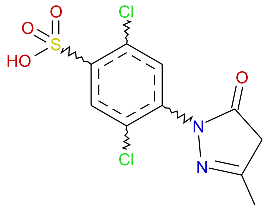	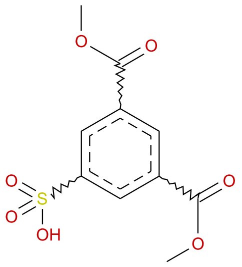	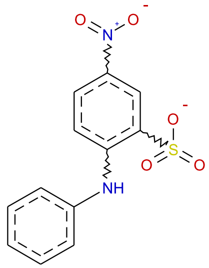
Actual Endpoint	Irritant	Irritant	Irritant
Predicted Endpoint	Irritant	Non-Irritant	Irritant
Distance	0.600	0.649	0.660
Reference	28ZPAK-;186;72	Prehled Prumyslove Toxikologie; Organicke Latky; Marhold; J. - ;1059;86	Prehled Prumyslove Toxikologie; Organicke Latky; Marhold; J. - ;1061;86

Model Applicability

Unknown features are fingerprint features in the query molecule, but not found or appearing too infrequently in the training set.

1. All properties and OPS components are within expected ranges.
2. Unknown FCFP_2 feature: -1564473960: [*]:n1:[*]:[*]:n:[c]:1:[c](:[*]):[*]

Feature Contribution

Top features for positive contribution				
Fingerprint	Bit/Smiles	Feature Structure	Score	Irritant in training set
FCFP_12	17	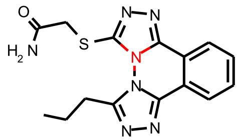 [*]:n:[*]	0.189	48 out of 49

FCFP_12	-1410079687	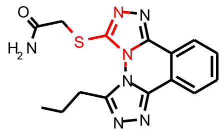 [*]S[c]1:n:[*]:[*]:n:1:[*]	0.187	8 out of 8
FCFP_12	4427049	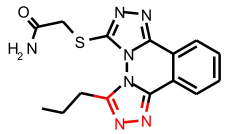 [*][c](:[*]):n:n(:[*]):[*]	0.167	4 out of 4

Top Features for negative contribution

**Fingerprint**	**Bit/Smiles**	**Feature Structure**	**Score**	**Irritant in training set**
FCFP_12	-1272798659	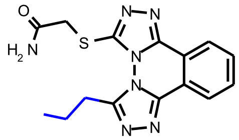 [*]CCC	0	517 out of 643
FCFP_12	-1272768868	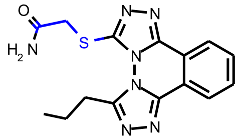 [*]SCC(=[*])[*]	0	396 out of 514
FCFP_12	307419094	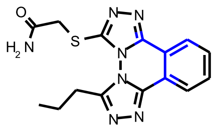 [*][c](:[*]):[c](:[cH]:[*]):[c](:[*]):[*]	0	43 out of 52

Doxorubicin

TOPKAT_Ocular_Irritancy_None_vs_Irritant

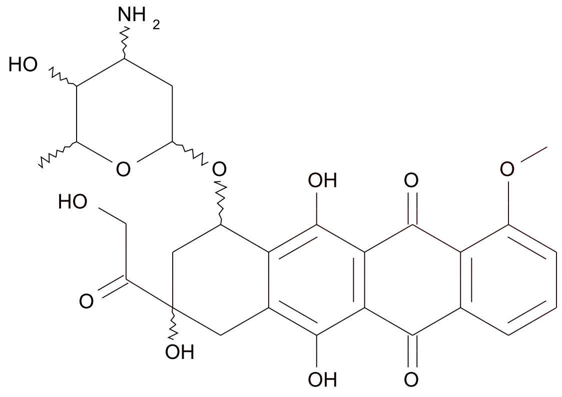

$C_{27}H_{29}NO_{11}$

Molecular Weight: 543.51925

ALogP: -4.4e-002

Rotatable Bonds: 5

Acceptors: 12

Donors: 6

Structural Similar Compounds

Name	Methanol; (s-triazine-2;4;6-triyltrinitrilo)hexa-	2-Naphthalenesulfonic acid; 5;6'-iminobis(1-hydroxy-	1;3;6-NAPHTHALENE TRISULFONIC ACID;7-AMINO-
Structure	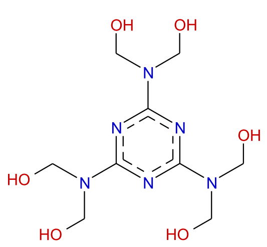	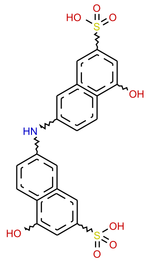	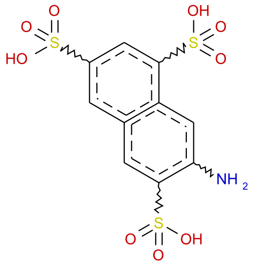
Actual Endpoint	Irritant	Irritant	Irritant
Predicted Endpoint	Irritant	Irritant	Irritant
Distance	0.923	0.958	1.164
Reference	Prehled Prumyslove Toxikologie; Organicke Latky; Marhold; J. -;876;86	Prehled Prumyslove Toxikologie; Organicke Latky; Marhold; J. pp 1065;86	28ZPAK-;190;72

Model Prediction

Prediction: Irritant

Probability: 1

Enrichment: 1.18

Bayesian Score: 1.88

Mahalanobis Distance: 14.4

Mahalanobis Distance p-value: 1.33e-011

Prediction: Positive if the Bayesian score is above the estimated best cutoff value from minimizing the false positive and false negative rate.
Probability: The esimated probability that the sample is in the positive category. This assumes that the Bayesian score follows a normal distribution and is different from the prediction using a cutoff.
Enrichment: An estimate of enrichment, that is, the increased likelihood (versus random) of this sample being in the category.
Bayesian Score: The standard Laplacian-modified Bayesian score.
Mahalanobis Distance: The Mahalanobis distance (MD) is the distance to the center of the training data. The larger the MD, the less trustworthy the prediction.
Mahalanobis Distance p-value: The p-value gives the fraction of training data with an MD greater than or equal to the one for the given sample, assuming normally distributed data. The smaller the p-value, the less trustworthy the prediciton. For highly non-normal X properties (e.g., fingerprints), the MD p-value is wildly inaccurate.

Model Applicability

Unknown features are fingerprint features in the query molecule, but not found or appearing too infrequently in the training set.

1. OPS PC15 out of range. Value: 4.2823. Training min, max, SD, explained variance: -4.3422, 4.1885, 1.08, 0.0142.

Feature Contribution

Top features for positive contribution				
**Fingerprint**	**Bit/Smiles**	**Feature Structure**	**Score**	**Irritant in training set**
FCFP_12	-415156552	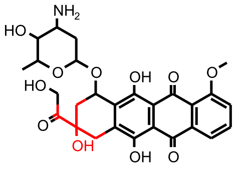 [*]CC(O)(C[*])C(=[*]) [*]	0.184	7 out of 7

FCFP_12	-1601875224	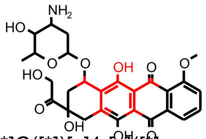 [*]C([*])[c]1:[c]([*]):[*]:[c]([*]):[c](C(=[*])[*]):[c]:1O	0.184	7 out of 7
FCFP_12	-1099193755	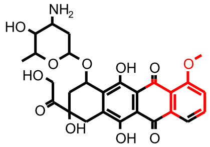 [*]C(=[*])[c]1:[c]([*]):[*]:[cH]:[cH]:[c]:1OC	0.175	5 out of 5

Top Features for negative contribution

Fingerprint	Bit/Smiles	Feature Structure	Score	Irritant in training set
FCFP_12	1186333723	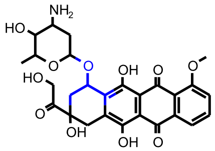 [*]CC(O[*])[c](:[*]):[*]	-0.344	2 out of 4
FCFP_12	1312974458	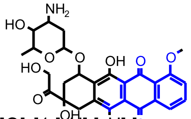 [*]O[c]1:[cH]:[cH]:[cH]:[c]2C(=O)[c](:[c]([*]):[*]):[c](:[*])C(=O)[c]:1:2	-0.268	1 out of 2
FCFP_12	-880844584	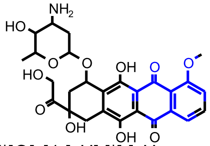 [*]O[c]1:[cH]:[*]:[cH]:[c]2C(=[*])[*]:[c](:[*])C(=O)[c]:1:2	-0.132	2 out of 3

TOPKAT_Skin_Irritancy_Mild_vs_Moderate_Severe

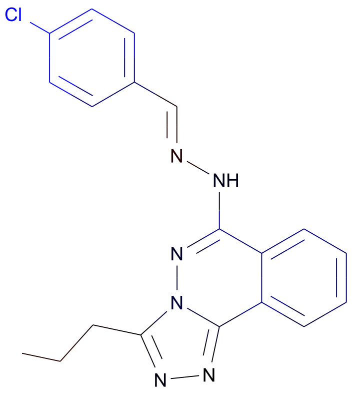

$C_{19}H_{17}ClN_6$

Molecular Weight: 364.83147

ALogP: 4.967

Rotatable Bonds: 5

Acceptors: 5

Donors: 1

Structural Similar Compounds

Name	Anthraquinone, 1,1'-iminodi-	Phosphorothioic acid, O-ethyl S-propyl O-(2,4,6-trichlorophenyl) es ter	Aniline, 2,4-bis(o-methylphenoxy)-
Structure	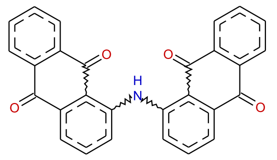	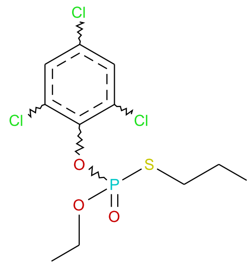	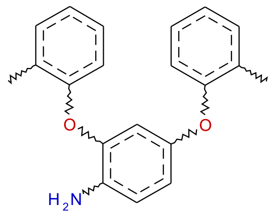
Actual Endpoint	Mild	Moderate_Severe	Mild
Predicted Endpoint	Mild	Mild	Mild
Distance	0.669	0.711	0.733
Reference	85JCAE "Prehled Prumyslove Toxikologie; Organicke Latky," Marhold, J., Prague , Czechoslovakia, Avicenum, 1986 Volume(issue)/page/year: -,735,1986	NTIS** National Technical Information Service. (Springfield, VA 22161) Forme rly U.S. Clearinghouse for Scientific & Technical Information. Volume(issue)/pag e/year: OTS0535844	85JCAE "Prehled Prumyslove Toxikologie; Organicke Latky," Marhold, J., Prague , Czechoslovakia, Avicenum, 1986 Volume(issue)/page/year: -,725,1986

Model Prediction

Prediction: Mild

Probability: 0.0319

Enrichment: 0.0866

Bayesian Score: -12.4

Mahalanobis Distance: 8.52

Mahalanobis Distance p-value: 0.527

Prediction: Positive if the Bayesian score is above the estimated best cutoff value from minimizing the false positive and false negative rate.
Probability: The esimated probability that the sample is in the positive category. This assumes that the Bayesian score follows a normal distribution and is different from the prediction using a cutoff.
Enrichment: An estimate of enrichment, that is, the increased likelihood (versus random) of this sample being in the category.
Bayesian Score: The standard Laplacian-modified Bayesian score.
Mahalanobis Distance: The Mahalanobis distance (MD) is the distance to the center of the training data. The larger the MD, the less trustworthy the prediction.
Mahalanobis Distance p-value: The p-value gives the fraction of training data with an MD greater than or equal to the one for the given sample, assuming normally distributed data. The smaller the p-value, the less trustworthy the prediciton. For highly non-normal X properties (e.g., fingerprints), the MD p-value is wildly inaccurate.

Model Applicability

Unknown features are fingerprint features in the query molecule, but not found or appearing too infrequently in the training set.

1. All properties and OPS components are within expected ranges.
2. Unknown FCFP_2 feature: -332197802: [*][c]1:[*]:[*]:[c](:[*]):n:1:n:[*]
3. Unknown FCFP_2 feature: -1564473960: [*]:n1:[*]:[*]:n:[c]:1:[c](:[*]):[*]

Feature Contribution

Top features for positive contribution				
**Fingerprint**	**Bit/Smiles**	**Feature Structure**	**Score**	**Moderate_Severe in training set**

FCFP_12	-577707014	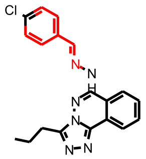 [*]\N=C\[c]1:[cH]:[cH]:[*]:[cH]:[cH]:1	0.717	4 out of 4
FCFP_12	-2100785893	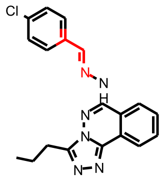 [*]\N=C\[c](:[*]):[*]	0.552	7 out of 10
FCFP_12	-1151884458	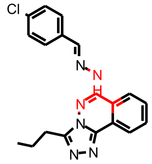 [*]N[c](:n:[*]):[c](:[*]):[*]	0.385	1 out of 1
**Top Features for negative contribution**				
**Fingerprint**	**Bit/Smiles**	**Feature Structure**	**Score**	**Moderate_Severe in training set**
FCFP_12	367998008	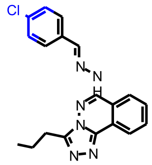 [*]:[cH]:[c](Cl):[cH]:[*]	-1.34	4 out of 50
FCFP_12	551850122	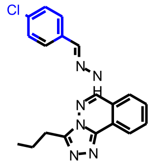 [*][c]1:[*]:[cH]:[c](Cl):[cH]:[cH]:1	-1.27	2 out of 27

FCFP_12	-1508180856	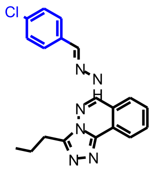<br>[*][c]1:[cH]:[cH]:[c](Cl):[cH]:[cH]:1	-1.26	0 out of 7

TOPKAT_Skin_Irritancy_Mild_vs_Moderate_Severe

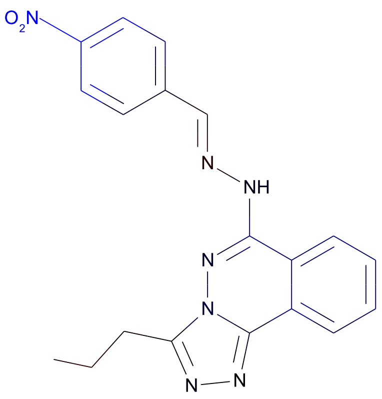

$C_{19}H_{17}N_7O_2$

Molecular Weight: 375.38397

ALogP: 4.197

Rotatable Bonds: 6

Acceptors: 7

Donors: 1

Model Prediction

Prediction: Mild

Probability: 0.00857

Enrichment: 0.0233

Bayesian Score: -16.3

Mahalanobis Distance: 7.75

Mahalanobis Distance p-value: 0.871

Prediction: Positive if the Bayesian score is above the estimated best cutoff value from minimizing the false positive and false negative rate.
Probability: The esimated probability that the sample is in the positive category. This assumes that the Bayesian score follows a normal distribution and is different from the prediction using a cutoff.
Enrichment: An estimate of enrichment, that is, the increased likelihood (versus random) of this sample being in the category.
Bayesian Score: The standard Laplacian-modified Bayesian score.
Mahalanobis Distance: The Mahalanobis distance (MD) is the distance to the center of the training data. The larger the MD, the less trustworthy the prediction.
Mahalanobis Distance p-value: The p-value gives the fraction of training data with an MD greater than or equal to the one for the given sample, assuming normally distributed data. The smaller the p-value, the less trustworthy the prediciton. For highly non-normal X properties (e.g., fingerprints), the MD p-value is wildly inaccurate.

Structural Similar Compounds

Name	Pregna-1,4-diene-3,20-dione, 21-(acetyloxy)-11-hydroxy-6-methyl-17- (1-oxopropoxy)-, (6-alpha,11-beta)-	1,4-Pentadien-3-one, 1,5-bis(p-azidophenyl)-	Acetic acid, 2-(sec-butyl)-4,6-dinitrophenyl ester
Structure	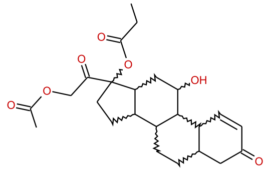	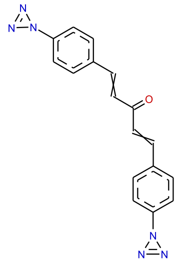	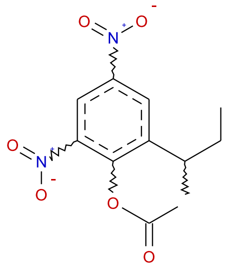
Actual Endpoint	Mild	Mild	Mild
Predicted Endpoint	Mild	Mild	Mild
Distance	0.730	0.808	0.809
Reference	YACHDS Yakuri to Chiryo. Pharmacology and Therapeutics. (Raifu Saiensu Shup pan K.K., 2-5-13, Yaesu, Chuo-ku, Tokyo 104, Japan) V.1- 1972- Volume(issue) /page/year: 19,3103,1991	85JCAE "Prehled Prumyslove Toxikologie; Organicke Latky," Marhold, J., Prague , Czechoslovakia, Avicenum, 1986 Volume(issue)/page/year: -,733,1986	85JCAE "Prehled Prumyslove Toxikologie; Organicke Latky," Marhold, J., Prague , Czechoslovakia, Avicenum, 1986 Volume(issue)/page/year: -,750,1986

Model Applicability

Unknown features are fingerprint features in the query molecule, but not found or appearing too infrequently in the training set.

1. All properties and OPS components are within expected ranges.
2. Unknown FCFP_2 feature: -332197802: [*][c]1:[*]:[*]:[c](:[*]):n:1:n:[*]
3. Unknown FCFP_2 feature: -1564473960: [*]:n1:[*]:[*]:n:[c]:1:[c](:[*]):[*]

Feature Contribution

Top features for positive contribution

Fingerprint	Bit/Smiles	Feature Structure	Score	Moderate_Severe in training set

FCFP_12	-577707014	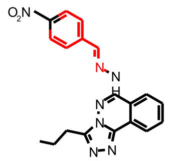 [*]\N=C\[c]1:[cH]:[cH]:[*]:[cH]:[cH]:1	0.717	4 out of 4
FCFP_12	-2100785893	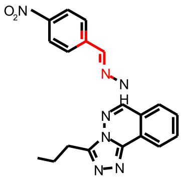 [*]\N=C\[c](:[*]):[*]	0.552	7 out of 10
FCFP_12	-1151884458	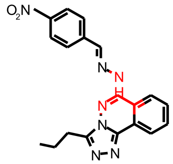 [*]N[c](:n:[*]):[c](:[*]):[*]	0.385	1 out of 1
**Top Features for negative contribution**				
**Fingerprint**	**Bit/Smiles**	**Feature Structure**	**Score**	**Moderate_Severe in training set**
FCFP_12	8	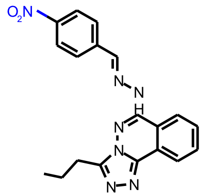 [*][N+](=[*])[*]	-1.6	0 out of 11
FCFP_12	-828984032	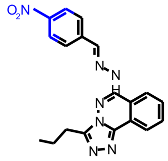 [*][N+](=[*])[c](:[cH]:[*]):[cH]:[*]	-1.36	0 out of 8

FCFP_12	1872392852	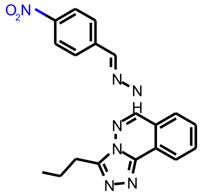 [*][N+](=O)[*]	-1.26	0 out of 7

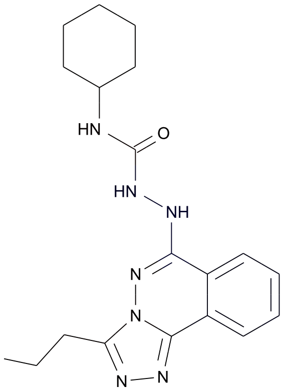

$C_{19}H_{25}N_7O$

Molecular Weight: 367.44809

ALogP: 3.681

Rotatable Bonds: 5

Acceptors: 5

Donors: 3

Model Prediction

Prediction: Non-Irritant

Probability: 0.968

Enrichment: 1.05

Bayesian Score: -1.11

Mahalanobis Distance: 10

Mahalanobis Distance p-value: 0.0583

Prediction: Positive if the Bayesian score is above the estimated best cutoff value from minimizing the false positive and false negative rate.
Probability: The esimated probability that the sample is in the positive category. This assumes that the Bayesian score follows a normal distribution and is different from the prediction using a cutoff.
Enrichment: An estimate of enrichment, that is, the increased likelihood (versus random) of this sample being in the category.
Bayesian Score: The standard Laplacian-modified Bayesian score.
Mahalanobis Distance: The Mahalanobis distance (MD) is the distance to the center of the training data. The larger the MD, the less trustworthy the prediction.
Mahalanobis Distance p-value: The p-value gives the fraction of training data with an MD greater than or equal to the one for the given sample, assuming normally distributed data. The smaller the p-value, the less trustworthy the prediciton. For highly non-normal X properties (e.g., fingerprints), the MD p-value is wildly inaccurate.

Structural Similar Compounds

Name	5-Norbornene-2,3-dicarboxylic acid, 1,4,5,6,7,7-hexachloro-	1-Amino-2-bromo-4-hydroxyanthraquinone	8-Methylamino-4-hydroxy-2-naphthalene sulfonic acid
Structure	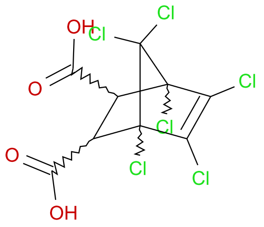	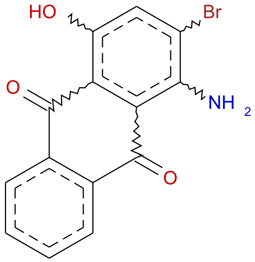	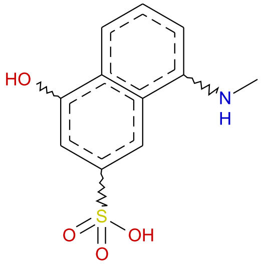
Actual Endpoint	Irritant	Non-Irritant	Non-Irritant
Predicted Endpoint	Irritant	Non-Irritant	Non-Irritant
Distance	0.796	0.840	0.844
Reference	85JCAE "Prehled Prumyslove Toxikologie; Organicke Latky," Marhold, J., Prague , Czechoslovakia, Avicenum, 1986 Volume(issue)/page/year: -,581,1986	28ZPAK -,83,72	28ZPAK -,190,72

Model Applicability

Unknown features are fingerprint features in the query molecule, but not found or appearing too infrequently in the training set.

1. All properties and OPS components are within expected ranges.
2. Unknown FCFP_2 feature: -332197802: [*][c]1:[*]:[*]:[c](:[*]):n:1:n:[*]

Feature Contribution

Top features for positive contribution

Fingerprint	Bit/Smiles	Feature Structure	Score	Irritant in training set

FCFP_12	4427049	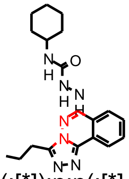 [*][c](:[*]):n:n(:[*]):[*]	0.0734	5 out of 5
FCFP_12	-796673622	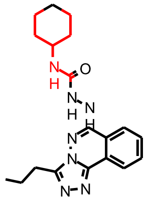 [*]C(=[*])NC1CC[*]CC1	0.0658	3 out of 3
FCFP_12	1499521844	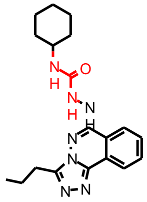 [*]NC(=O)N[*]	0.0658	3 out of 3

Top Features for negative contribution

**Fingerprint**	**Bit/Smiles**	**Feature Structure**	**Score**	**Irritant in training set**
FCFP_12	1294344583	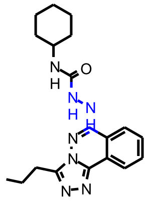 [*]NN[c](:[*]):[*]	-0.65	0 out of 1
FCFP_12	-1320007763	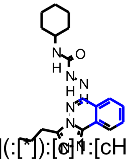 [*][c](:[*]):[c]1:[cH]:[cH]:[cH]:[*]:[c]:1:[*]	-0.0893	20 out of 24

FCFP_12	1618154665	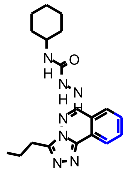 [*]:[cH]:[cH]:[cH]:[*]	-0.0845	412 out of 490

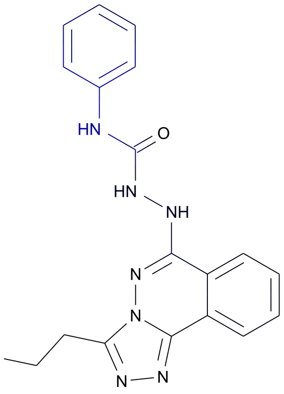

$C_{19}H_{19}N_7O$
Molecular Weight: 361.40046
ALogP: 3.399
Rotatable Bonds: 5
Acceptors: 5
Donors: 3

Model Prediction

Prediction: Non-Irritant
Probability: 0.576
Enrichment: 0.625
Bayesian Score: -4.34
Mahalanobis Distance: 7.03
Mahalanobis Distance p-value: 0.996

Prediction: Positive if the Bayesian score is above the estimated best cutoff value from minimizing the false positive and false negative rate.
Probability: The esimated probability that the sample is in the positive category. This assumes that the Bayesian score follows a normal distribution and is different from the prediction using a cutoff.
Enrichment: An estimate of enrichment, that is, the increased likelihood (versus random) of this sample being in the category.
Bayesian Score: The standard Laplacian-modified Bayesian score.
Mahalanobis Distance: The Mahalanobis distance (MD) is the distance to the center of the training data. The larger the MD, the less trustworthy the prediction.
Mahalanobis Distance p-value: The p-value gives the fraction of training data with an MD greater than or equal to the one for the given sample, assuming normally distributed data. The smaller the p-value, the less trustworthy the prediciton. For highly non-normal X properties (e.g., fingerprints), the MD p-value is wildly inaccurate.

Structural Similar Compounds

Name	5-Norbornene-2,3-dicarboxylic acid, 1,4,5,6,7,7-hexachloro-	8-Methylamino-4-hydroxy-2-naphthalene sulfonic acid	p-Acetophenetidide, 3'-(bis(2-hydroxyethyl)amino)-
Structure	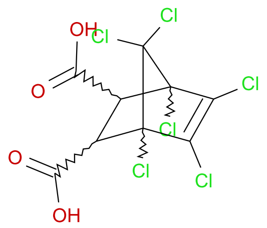	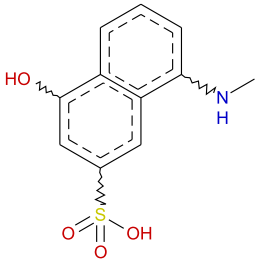	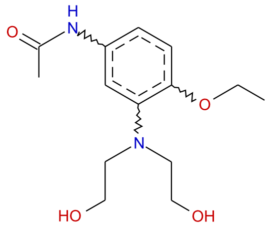
Actual Endpoint	Irritant	Non-Irritant	Non-Irritant
Predicted Endpoint	Irritant	Non-Irritant	Non-Irritant
Distance	0.790	0.805	0.817
Reference	85JCAE "Prehled Prumyslove Toxikologie; Organicke Latky," Marhold, J., Prague , Czechoslovakia, Avicenum, 1986 Volume(issue)/page/year: -,581,1986	28ZPAK -,190,72	28ZPAK -,100,72

Model Applicability

Unknown features are fingerprint features in the query molecule, but not found or appearing too infrequently in the training set.

1. All properties and OPS components are within expected ranges.
2. Unknown FCFP_2 feature: -332197802: [*][c]1:[*]:[*]:[c](:[*]):n:1:n:[*]

Feature Contribution

Top features for positive contribution				
Fingerprint	Bit/Smiles	Feature Structure	Score	Irritant in training set

FCFP_12	4427049	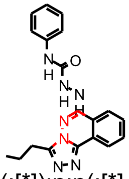 [*][c](:[*]):n:n(:[*]):[*]	0.0734	5 out of 5
FCFP_12	1499521844	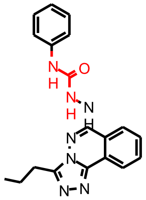 [*]NC(=O)N[*]	0.0658	3 out of 3
FCFP_12	-1578026142	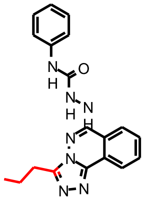 [*]:[c](:[*])CCC	0.0583	2 out of 2

Top Features for negative contribution

Fingerprint	Bit/Smiles	Feature Structure	Score	Irritant in training set
FCFP_12	-1838187238	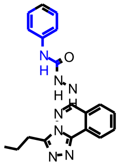 [*]C(=[*])N[c]1:[cH]:[cH]:[*]:[cH]:[cH]:1	-0.692	5 out of 12
FCFP_12	1294344583	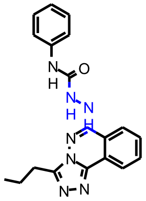 [*]NN[c](:[*]):[*]	-0.65	0 out of 1

FCFP_12	-792685140	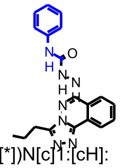 [*]C(=[*])N[c]1:[cH]:[cH]:[cH]:[cH]:[cH]:1	-0.65	0 out of 1

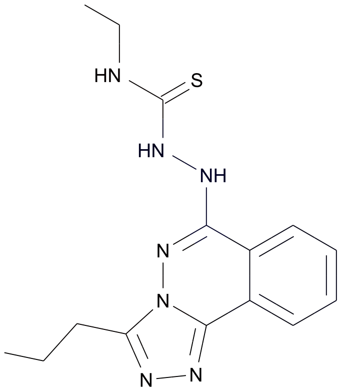

$C_{15}H_{19}N_7S$
Molecular Weight: 329.42325
ALogP: 3.388
Rotatable Bonds: 7
Acceptors: 5
Donors: 3

Model Prediction

Prediction: Non-Irritant
Probability: 0.967
Enrichment: 1.05
Bayesian Score: -1.2
Mahalanobis Distance: 8.05
Mahalanobis Distance p-value: 0.87

Prediction: Positive if the Bayesian score is above the estimated best cutoff value from minimizing the false positive and false negative rate.
Probability: The esimated probability that the sample is in the positive category. This assumes that the Bayesian score follows a normal distribution and is different from the prediction using a cutoff.
Enrichment: An estimate of enrichment, that is, the increased likelihood (versus random) of this sample being in the category.
Bayesian Score: The standard Laplacian-modified Bayesian score.
Mahalanobis Distance: The Mahalanobis distance (MD) is the distance to the center of the training data. The larger the MD, the less trustworthy the prediction.
Mahalanobis Distance p-value: The p-value gives the fraction of training data with an MD greater than or equal to the one for the given sample, assuming normally distributed data. The smaller the p-value, the less trustworthy the prediciton. For highly non-normal X properties (e.g., fingerprints), the MD p-value is wildly inaccurate.

Structural Similar Compounds

Name	Benzenesulfonic acid, 2-anilino-5-nitro-	8-Methylamino-4-hydroxy-2-naphthalene sulfonic acid	C.I. Fluorescent Brightening Agent 24
Structure	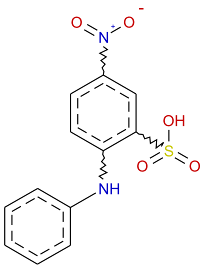	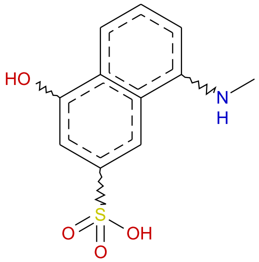	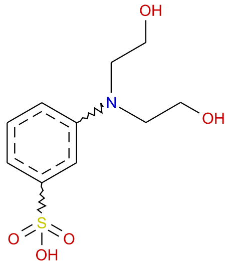
Actual Endpoint	Irritant	Non-Irritant	Irritant
Predicted Endpoint	Non-Irritant	Non-Irritant	Non-Irritant
Distance	0.793	0.802	0.817
Reference	85JCAE "Prehled Prumyslove Toxikologie; Organicke Latky," Marhold, J., Prague , Czechoslovakia, Avicenum, 1986 Volume(issue)/page/year: -,1061,1986	28ZPAK -,190,72	MVCRB3 MVC-Report. (Stockholm, Sweden) No.1-2, 1972-73. Discontinued. Volu me(issue)/page/year: 2,193,1973

Model Applicability

Unknown features are fingerprint features in the query molecule, but not found or appearing too infrequently in the training set.

1. All properties and OPS components are within expected ranges.
2. Unknown FCFP_2 feature: -332197802: [*][c]1:[*]:[*]:[c](:[*]):n:1:n:[*]

Feature Contribution

Top features for positive contribution

Fingerprint	Bit/Smiles	Feature Structure	Score	Irritant in training set

FCFP_12	4427049	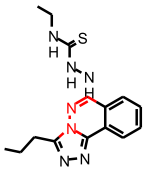 [*][c](:[*]):n:n(:[*]):[*]	0.0734	5 out of 5
FCFP_12	-1939253119	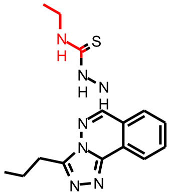 [*]C(=[*])NCC	0.0658	3 out of 3
FCFP_12	1499521844	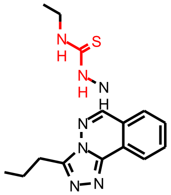 [*]NC(=O)N[*]	0.0658	3 out of 3

Top Features for negative contribution

Fingerprint	Bit/Smiles	Feature Structure	Score	Irritant in training set
FCFP_12	1294344583	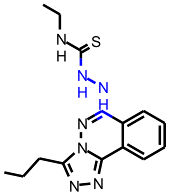 [*]NN[c](:[*]):[*]	-0.65	0 out of 1
FCFP_12	-1320007763	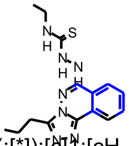 [*][c](:[*]):[c]1:[cH]:[cH]:[cH]:[*]:[c]: 1:[*]	-0.0893	20 out of 24

FCFP_12	1618154665	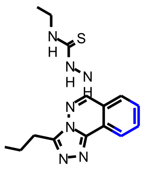 [*]:[cH]:[cH]:[cH]:[*]	-0.0845	412 out of 490

TOPKAT_Skin_Irritancy_None_vs_Irritant

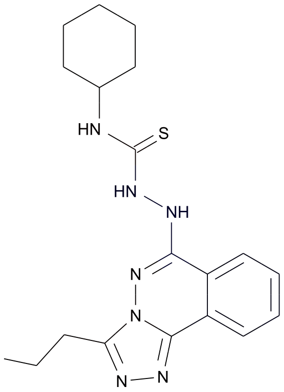

$C_{19}H_{25}N_7S$
Molecular Weight: 383.51369
ALogP: 4.898
Rotatable Bonds: 7
Acceptors: 5
Donors: 3

Model Prediction

Prediction: Non-Irritant
Probability: 0.968
Enrichment: 1.05
Bayesian Score: -1.16
Mahalanobis Distance: 10.1
Mahalanobis Distance p-value: 0.0523

Prediction: Positive if the Bayesian score is above the estimated best cutoff value from minimizing the false positive and false negative rate.
Probability: The esimated probability that the sample is in the positive category. This assumes that the Bayesian score follows a normal distribution and is different from the prediction using a cutoff.
Enrichment: An estimate of enrichment, that is, the increased likelihood (versus random) of this sample being in the category.
Bayesian Score: The standard Laplacian-modified Bayesian score.
Mahalanobis Distance: The Mahalanobis distance (MD) is the distance to the center of the training data. The larger the MD, the less trustworthy the prediction.
Mahalanobis Distance p-value: The p-value gives the fraction of training data with an MD greater than or equal to the one for the given sample, assuming normally distributed data. The smaller the p-value, the less trustworthy the prediciton. For highly non-normal X properties (e.g., fingerprints), the MD p-value is wildly inaccurate.

Structural Similar Compounds

Name	Benzenesulfonic acid, 2,2'-(4,4'-biphenylylenedivinylene)di-, disod ium salt	Urea, 1,3-bis(2-benzothiazolylthiomethyl)-	Benzenesulfonic acid, 2-anilino-5-nitro-
Structure	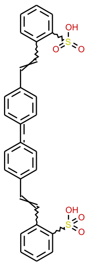	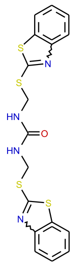	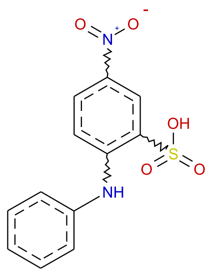
Actual Endpoint	Irritant	Irritant	Irritant
Predicted Endpoint	Non-Irritant	Irritant	Non-Irritant
Distance	0.858	0.910	0.911
Reference	MVCRB3 MVC-Report. (Stockholm, Sweden) No.1-2, 1972-73. Discontinued. Volu me(issue)/page/year: 2,193,1973	AMIHBC AMA Archives of Industrial Hygiene and Occupational Medicine. (Chicago , IL) V.2-10, 1950-54. For publisher information, see AEHLAU. Volume(issue)/pag e/year: 5,311,1952	85JCAE "Prehled Prumyslove Toxikologie; Organicke Latky," Marhold, J., Prague , Czechoslovakia, Avicenum, 1986 Volume(issue)/page/year: -,1061,1986

Model Applicability

Unknown features are fingerprint features in the query molecule, but not found or appearing too infrequently in the training set.

1. All properties and OPS components are within expected ranges.
2. Unknown FCFP_2 feature: -332197802: [*][c]1:[*]:[*]:[c](:[*]):n:1:n:[*]

Feature Contribution

Top features for positive contribution				
Fingerprint	Bit/Smiles	Feature Structure	Score	Irritant in training set

FCFP_12	4427049	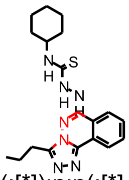 [*][c](:[*]):n:n(:[*]):[*]	0.0734	5 out of 5
FCFP_12	-796673622	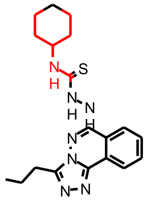 [*]C(=[*])NC1CC[*]CC1	0.0658	3 out of 3
FCFP_12	1499521844	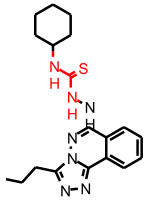 [*]NC(=O)N[*]	0.0658	3 out of 3

Top Features for negative contribution

**Fingerprint**	**Bit/Smiles**	**Feature Structure**	**Score**	**Irritant in training set**
FCFP_12	1294344583	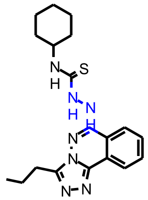 [*]NN[c](:[*]):[*]	-0.65	0 out of 1
FCFP_12	-1320007763	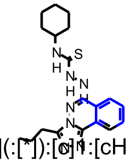 [*][c](:[*]):[c]1:[cH]:[cH]:[cH]:[*]:[c]: 1:[*]	-0.0893	20 out of 24

FCFP_12	1618154665	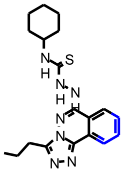 [*]:[cH]:[cH]:[cH]:[*]	-0.0845	412 out of 490

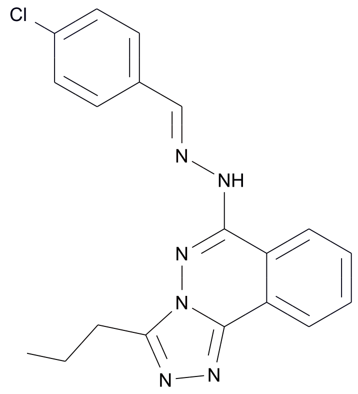

$C_{19}H_{17}ClN_6$
Molecular Weight: 364.83147
ALogP: 4.967
Rotatable Bonds: 5
Acceptors: 5
Donors: 1

Model Prediction

Prediction: Irritant
Probability: 0.974
Enrichment: 1.06
Bayesian Score: -0.717
Mahalanobis Distance: 8.11
Mahalanobis Distance p-value: 0.849

Prediction: Positive if the Bayesian score is above the estimated best cutoff value from minimizing the false positive and false negative rate.
Probability: The esimated probability that the sample is in the positive category. This assumes that the Bayesian score follows a normal distribution and is different from the prediction using a cutoff.
Enrichment: An estimate of enrichment, that is, the increased likelihood (versus random) of this sample being in the category.
Bayesian Score: The standard Laplacian-modified Bayesian score.
Mahalanobis Distance: The Mahalanobis distance (MD) is the distance to the center of the training data. The larger the MD, the less trustworthy the prediction.
Mahalanobis Distance p-value: The p-value gives the fraction of training data with an MD greater than or equal to the one for the given sample, assuming normally distributed data. The smaller the p-value, the less trustworthy the prediciton. For highly non-normal X properties (e.g., fingerprints), the MD p-value is wildly inaccurate.

Structural Similar Compounds

Name	1-Piperazineacetic acid, 4-(2-hydroxyethyl)-alpha-phenyl-, 2,6-xyly l ester, monohydrochloride	Anthraquinone, 1,1'-iminodi-	Phosphorothioic acid, O-ethyl S-propyl O-(2,4,6-trichlorophenyl) es ter
Structure	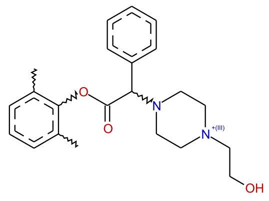	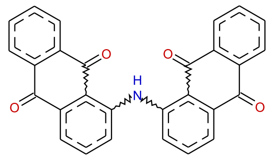	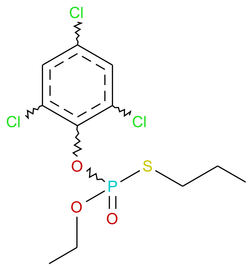
Actual Endpoint	Irritant	Irritant	Irritant
Predicted Endpoint	Irritant	Non-Irritant	Irritant
Distance	0.674	0.675	0.707
Reference	BCFAAI Bollettino Chimico Farmaceutico. (Societa Editoriale Farmaceutica, Vi a Ausonio 12, 20123 Milan, Italy) V.33- 1894- Volume(issue)/page/year: 107,3 10,1968	85JCAE "Prehled Prumyslove Toxikologie; Organicke Latky," Marhold, J., Prague , Czechoslovakia, Avicenum, 1986 Volume(issue)/page/year: -,735,1986	NTIS** National Technical Information Service. (Springfield, VA 22161) Forme rly U.S. Clearinghouse for Scientific & Technical Information. Volume(issue)/pag e/year: OTS0535844

Model Applicability

Unknown features are fingerprint features in the query molecule, but not found or appearing too infrequently in the training set.

1. All properties and OPS components are within expected ranges.
2. Unknown FCFP_2 feature: -332197802: [*][c]1:[*]:[*]:[c](:[*]):n:1:n:[*]

Feature Contribution

Top features for positive contribution				
Fingerprint	Bit/Smiles	Feature Structure	Score	Irritant in training set

FCFP_12	-2100785893	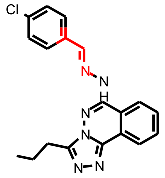 [*]\N=C\[c](:[*]):[*]	0.081	11 out of 11
FCFP_12	-577707014	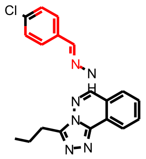 [*]\N=C\[c]1:[cH]:[cH]:[*]:[cH]:[cH]:1	0.0734	5 out of 5
FCFP_12	4427049	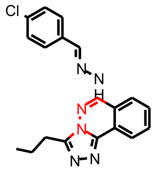 [*][c](:[*]):n:n(:[*]):[*]	0.0734	5 out of 5

Top Features for negative contribution

Fingerprint	Bit/Smiles	Feature Structure	Score	Irritant in training set
FCFP_12	367998008	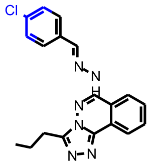 [*]:[cH]:[c](Cl):[cH]:[*]	-0.129	61 out of 76
FCFP_12	-1508180856	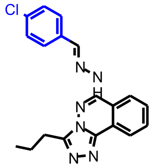 [*][c]1:[cH]:[cH]:[c](Cl):[cH]:[cH]:1	-0.125	12 out of 15

FCFP_12	71476542	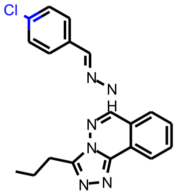<br>[*]:[c](:[*])Cl	-0.12	64 out of 79

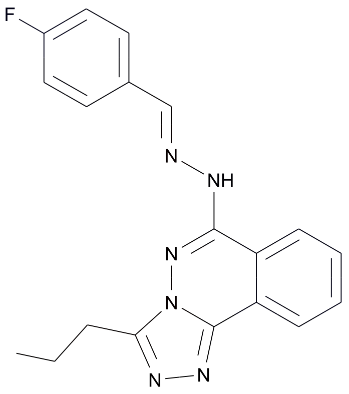

$C_{19}H_{17}FN_6$

Molecular Weight: 348.37688

ALogP: 4.508

Rotatable Bonds: 5

Acceptors: 5

Donors: 1

Model Prediction

Prediction: Non-Irritant

Probability: 0.973

Enrichment: 1.06

Bayesian Score: -0.774

Mahalanobis Distance: 8.1

Mahalanobis Distance p-value: 0.855

Prediction: Positive if the Bayesian score is above the estimated best cutoff value from minimizing the false positive and false negative rate.
Probability: The esimated probability that the sample is in the positive category. This assumes that the Bayesian score follows a normal distribution and is different from the prediction using a cutoff.
Enrichment: An estimate of enrichment, that is, the increased likelihood (versus random) of this sample being in the category.
Bayesian Score: The standard Laplacian-modified Bayesian score.
Mahalanobis Distance: The Mahalanobis distance (MD) is the distance to the center of the training data. The larger the MD, the less trustworthy the prediction.
Mahalanobis Distance p-value: The p-value gives the fraction of training data with an MD greater than or equal to the one for the given sample, assuming normally distributed data. The smaller the p-value, the less trustworthy the prediciton. For highly non-normal X properties (e.g., fingerprints), the MD p-value is wildly inaccurate.

Structural Similar Compounds

Name	1-Piperazineacetic acid, 4-(2-hydroxyethyl)-alpha-phenyl-, 2,6-xyly l ester, monohydrochloride	s-Triazine, 2,4-dichloro-6-(o-chloroanilino)-	Anthraquinone, 1,1'-iminodi-
Structure	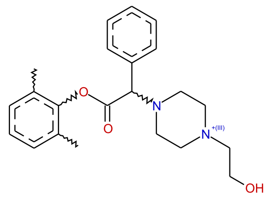	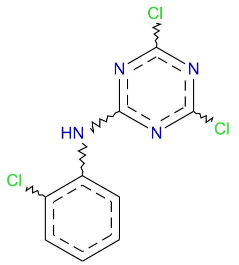	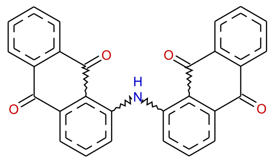
Actual Endpoint	Irritant	Irritant	Irritant
Predicted Endpoint	Irritant	Non-Irritant	Non-Irritant
Distance	0.660	0.705	0.708
Reference	BCFAAI Bollettino Chimico Farmaceutico. (Societa Editoriale Farmaceutica, Vi a Ausonio 12, 20123 Milan, Italy) V.33- 1894- Volume(issue)/page/year: 107,3 10,1968	34ZIAG* -,235,69	85JCAE "Prehled Prumyslove Toxikologie; Organicke Latky," Marhold, J., Prague , Czechoslovakia, Avicenum, 1986 Volume(issue)/page/year: -,735,1986

Model Applicability

Unknown features are fingerprint features in the query molecule, but not found or appearing too infrequently in the training set.

1. All properties and OPS components are within expected ranges.
2. Unknown FCFP_2 feature: -332197802: [*][c]1:[*]:[*]:[c](:[*]):n:1:n:[*]

Feature Contribution

Top features for positive contribution				
**Fingerprint**	**Bit/Smiles**	**Feature Structure**	**Score**	**Irritant in training set**

FCFP_12	-2100785893	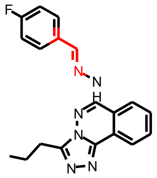 [*]\N=C\[c](:[*]):[*]	0.081	11 out of 11
FCFP_12	4427049	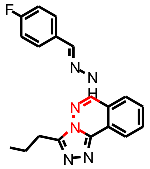 [*][c](:[*]):n:n(:[*]):[*]	0.0734	5 out of 5
FCFP_12	-577707014	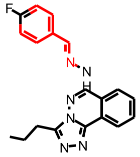 [*]\N=C\[c]1:[cH]:[cH]:[*]:[cH]:[cH]:1	0.0734	5 out of 5

Top Features for negative contribution

Fingerprint	Bit/Smiles	Feature Structure	Score	Irritant in training set
FCFP_12	367998008	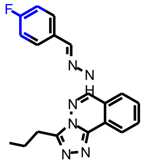 [*]:[cH]:[c](Cl):[cH] :[*]	-0.129	61 out of 76
FCFP_12	-1508180856	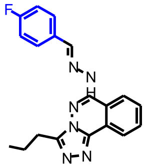 [*][c]1:[cH]:[cH]:[c] (Cl):[cH]:[cH]:1	-0.125	12 out of 15

FCFP_12	71476542	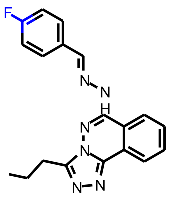 [*]:[c](:[*])Cl	-0.12	64 out of 79

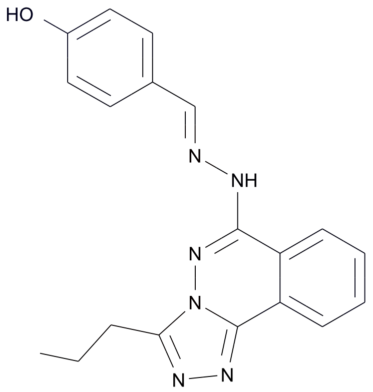

$C_{19}H_{18}N_6O$

Molecular Weight: 346.38582

ALogP: 4.061

Rotatable Bonds: 5

Acceptors: 6

Donors: 2

Model Prediction

Prediction: Non-Irritant

Probability: 0.966

Enrichment: 1.05

Bayesian Score: -1.24

Mahalanobis Distance: 8.51

Mahalanobis Distance p-value: 0.685

Prediction: Positive if the Bayesian score is above the estimated best cutoff value from minimizing the false positive and false negative rate.
Probability: The esimated probability that the sample is in the positive category. This assumes that the Bayesian score follows a normal distribution and is different from the prediction using a cutoff.
Enrichment: An estimate of enrichment, that is, the increased likelihood (versus random) of this sample being in the category.
Bayesian Score: The standard Laplacian-modified Bayesian score.
Mahalanobis Distance: The Mahalanobis distance (MD) is the distance to the center of the training data. The larger the MD, the less trustworthy the prediction.
Mahalanobis Distance p-value: The p-value gives the fraction of training data with an MD greater than or equal to the one for the given sample, assuming normally distributed data. The smaller the p-value, the less trustworthy the prediciton. For highly non-normal X properties (e.g., fingerprints), the MD p-value is wildly inaccurate.

Structural Similar Compounds

Name	5-Norbornene-2,3-dicarboxylic acid, 1,4,5,6,7,7-hexachloro-	1-Amino-2-bromo-4-hydroxyanthraquinone	Phenol, 4,4'-sulfonyldi-
Structure	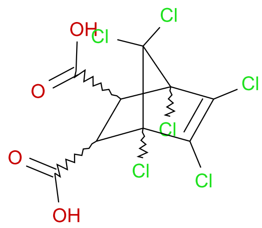	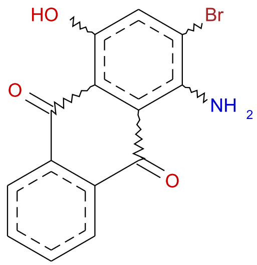	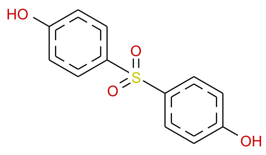
Actual Endpoint	Irritant	Non-Irritant	Irritant
Predicted Endpoint	Irritant	Non-Irritant	Non-Irritant
Distance	0.786	0.802	0.809
Reference	85JCAE "Prehled Prumyslove Toxikologie; Organicke Latky," Marhold, J., Prague , Czechoslovakia, Avicenum, 1986 Volume(issue)/page/year: -,581,1986	28ZPAK -,83,72	BIOFX* BIOFAX Industrial Bio-Test Laboratories, Inc., Data Sheets. (1810 Fro ntage Rd., Northbrook, IL 60062) Volume(issue)/page/year: 601-05501,1974

Model Applicability

Unknown features are fingerprint features in the query molecule, but not found or appearing too infrequently in the training set.

1. All properties and OPS components are within expected ranges.
2. Unknown FCFP_2 feature: -332197802: [*][c]1:[*]:[*]:[c](:[*]):n:1:n:[*]

Feature Contribution

Top features for positive contribution

Fingerprint	Bit/Smiles	Feature Structure	Score	Irritant in training set

FCFP_12	-2100785893	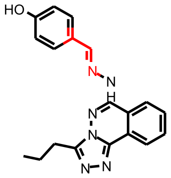<br>[*]\N=C\[c](:[*]):[*]	0.081	11 out of 11
FCFP_12	4427049	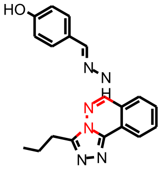<br>[*][c](:[*]):n:n(:[*]<br>):[*]	0.0734	5 out of 5
FCFP_12	-577707014	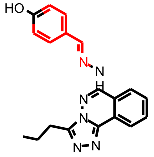<br>[*]\N=C\[c]1:[cH]:[cH<br>]:[*]:[cH]:[cH]:1	0.0734	5 out of 5
**Top Features for negative contribution**				
**Fingerprint**	**Bit/Smiles**	**Feature Structure**	**Score**	**Irritant in training set**
FCFP_12	7	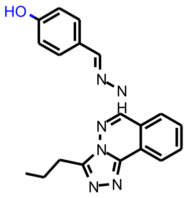<br>[*]O	-0.118	104 out of 128
FCFP_12	-549108873	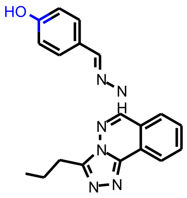<br>[*]:[c](:[*])O	-0.11	54 out of 66

FCFP_12	74595001	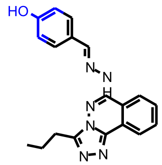 [*]:[cH]:[c](O):[cH]:[*]	-0.11	54 out of 66

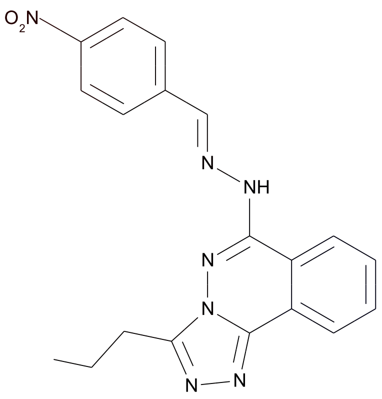

$C_{19}H_{17}N_{7}O_{2}$
Molecular Weight: 375.38397
ALogP: 4.197
Rotatable Bonds: 6
Acceptors: 7
Donors: 1

Structural Similar Compounds

Name	Pregna-1,4-diene-3,20-dione, 21-(acetyloxy)-11-hydroxy-6-methyl-17- (1-oxopropoxy)-, (6-alpha,11-beta)-	Acetic acid, 2-(sec-butyl)-4,6-dinitrophenyl ester	1,4-Pentadien-3-one, 1,5-bis(p-azidophenyl)-
Structure	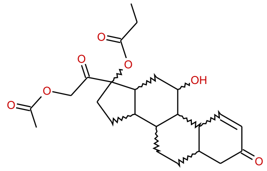	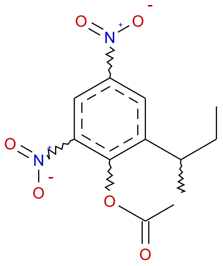	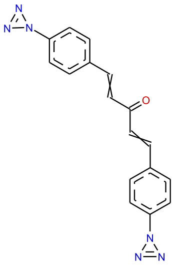
Actual Endpoint	Irritant	Irritant	Irritant
Predicted Endpoint	Irritant	Non-Irritant	Irritant
Distance	0.735	0.809	0.810
Reference	YACHDS Yakuri to Chiryo. Pharmacology and Therapeutics. (Raifu Saiensu Shup pan K.K., 2-5-13, Yaesu, Chuo-ku, Tokyo 104, Japan) V.1-1972- Volume(issue) /page/year: 19,3103,1991	85JCAE "Prehled Prumyslove Toxikologie; Organicke Latky," Marhold, J., Prague , Czechoslovakia, Avicenum, 1986 Volume(issue)/page/year: -,750,1986	85JCAE "Prehled Prumyslove Toxikologie; Organicke Latky," Marhold, J., Prague , Czechoslovakia, Avicenum, 1986 Volume(issue)/page/year: -,733,1986

Model Prediction

Prediction: Irritant
Probability: 0.996
Enrichment: 1.08
Bayesian Score: 0.207
Mahalanobis Distance: 7.65
Mahalanobis Distance p-value: 0.955

Prediction: Positive if the Bayesian score is above the estimated best cutoff value from minimizing the false positive and false negative rate.
Probability: The esimated probability that the sample is in the positive category. This assumes that the Bayesian score follows a normal distribution and is different from the prediction using a cutoff.
Enrichment: An estimate of enrichment, that is, the increased likelihood (versus random) of this sample being in the category.
Bayesian Score: The standard Laplacian-modified Bayesian score.
Mahalanobis Distance: The Mahalanobis distance (MD) is the distance to the center of the training data. The larger the MD, the less trustworthy the prediction.
Mahalanobis Distance p-value: The p-value gives the fraction of training data with an MD greater than or equal to the one for the given sample, assuming normally distributed data. The smaller the p-value, the less trustworthy the prediciton. For highly non-normal X properties (e.g., fingerprints), the MD p-value is wildly inaccurate.

Model Applicability

Unknown features are fingerprint features in the query molecule, but not found or appearing too infrequently in the training set.

1. All properties and OPS components are within expected ranges.
2. Unknown FCFP_2 feature: -332197802: [*][c]1:[*]:[*]:[c](:[*]):n:1:n:[*]

Feature Contribution

Top features for positive contribution

Fingerprint	Bit/Smiles	Feature Structure	Score	Irritant in training set

FCFP_12	5	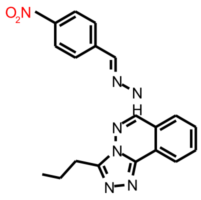 [*][O-]	0.0854	27 out of 27
FCFP_12	8	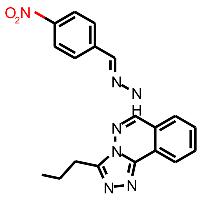 [*][N+](=[*])[*]	0.0843	20 out of 20
FCFP_12	-2100785893	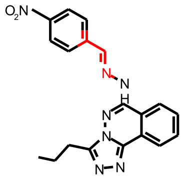 [*]\N=C\[c](:[*]):[*]	0.081	11 out of 11
**Top Features for negative contribution**				
**Fingerprint**	**Bit/Smiles**	**Feature Structure**	**Score**	**Irritant in training set**
FCFP_12	-1320007763	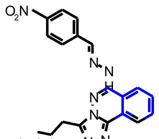 [*][c](:[*]):[c]1:[cH]:[cH]:[cH]:[*]:[c]: 1:[*]	-0.0893	20 out of 24
FCFP_12	1618154665	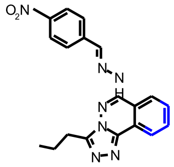 [*]:[cH]:[cH]:[cH]:[*]	-0.0845	412 out of 490

FCFP_12	16	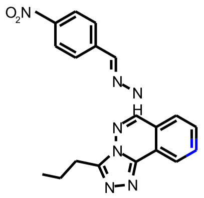 [*]:[cH]:[*]	-0.0843	423 out of 503

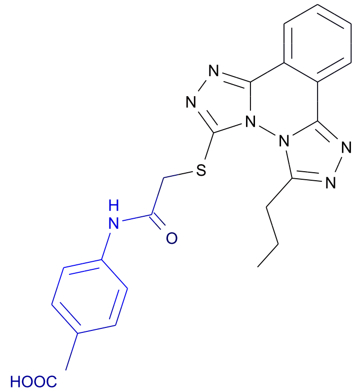

$C_{22}H_{19}N_7O_3S$
Molecular Weight: 461.49635
ALogP: 3.42
Rotatable Bonds: 7
Acceptors: 8
Donors: 2

Model Prediction

Prediction: Non-Irritant
Probability: 0.00137
Enrichment: 0.00149
Bayesian Score: -7.93
Mahalanobis Distance: 13.3
Mahalanobis Distance p-value: 8.82e-009

Prediction: Positive if the Bayesian score is above the estimated best cutoff value from minimizing the false positive and false negative rate.
Probability: The esimated probability that the sample is in the positive category. This assumes that the Bayesian score follows a normal distribution and is different from the prediction using a cutoff.
Enrichment: An estimate of enrichment, that is, the increased likelihood (versus random) of this sample being in the category.
Bayesian Score: The standard Laplacian-modified Bayesian score.
Mahalanobis Distance: The Mahalanobis distance (MD) is the distance to the center of the training data. The larger the MD, the less trustworthy the prediction.
Mahalanobis Distance p-value: The p-value gives the fraction of training data with an MD greater than or equal to the one for the given sample, assuming normally distributed data. The smaller the p-value, the less trustworthy the prediciton. For highly non-normal X properties (e.g., fingerprints), the MD p-value is wildly inaccurate.

Structural Similar Compounds

Name	2-Anthracenesulfonic acid, 1-amino-9,10-dihydro-9,10-dioxo-4-(2,4,6 -trimethylanilino)-, monosodium salt	2,2'-Stilbenedisulfonic acid, 4,4'-dinitro-	Benzenesulfonic acid, 2,2'-(4,4'-biphenylylenedivinylene)di-, disod ium salt
Structure	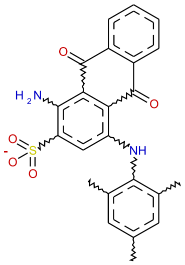	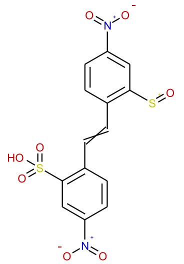	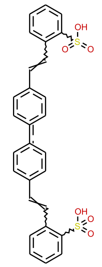
Actual Endpoint	Irritant	Irritant	Irritant
Predicted Endpoint	Non-Irritant	Irritant	Non-Irritant
Distance	0.724	0.804	0.847
Reference	85JCAE "Prehled Prumyslove Toxikologie; Organicke Latky," Marhold, J., Prague , Czechoslovakia, Avicenum, 1986 Volume(issue)/page/year: -,1327,1986	85JCAE "Prehled Prumyslove Toxikologie; Organicke Latky," Marhold, J., Prague , Czechoslovakia, Avicenum, 1986 Volume(issue)/page/year: -,1062,1986	MVCRB3 MVC-Report. (Stockholm, Sweden) No.1-2, 1972-73. Discontinued. Volu me(issue)/page/year: 2,193,1973

Model Applicability

Unknown features are fingerprint features in the query molecule, but not found or appearing too infrequently in the training set.

1. All properties and OPS components are within expected ranges.
2. Unknown FCFP_2 feature: -332197802: [*][c]1:[*]:[*]:[c](:[*]):n:1:n:[*]

Feature Contribution

Top features for positive contribution

Fingerprint	Bit/Smiles	Feature Structure	Score	Irritant in training set

FCFP_12	-1410079687	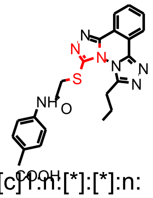<br>[*]S[c]1:n:[*]:[*]:n:1:[*]	0.0756	6 out of 6
FCFP_12	4427049	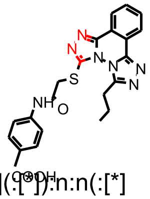<br>[*][c](:[*]):n:n(:[*]):[*]	0.0734	5 out of 5
FCFP_12	-1539162406	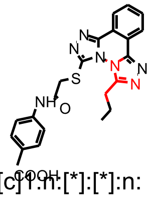<br>[*]C[c]1:n:[*]:[*]:n:1:[*]	0.0583	2 out of 2

Top Features for negative contribution

Fingerprint	Bit/Smiles	Feature Structure	Score	Irritant in training set
FCFP_12	1175665944	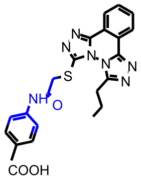<br>[*]CC(=O)N[c](:[cH]:[*]):[cH]:[*]	-1.02	2 out of 8
FCFP_12	-1838187238	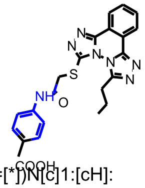<br>[*]C(=[*])N[c]1:[cH]:[cH]:[*]:[cH]:[cH]:1	-0.692	5 out of 12

FCFP_12	-451043714	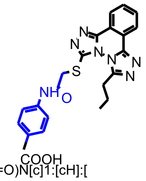 [*]CC(=O)N[c]1:[cH]:[cH]:[c]([*]):[cH]:[cH]:1	-0.65	0 out of 1

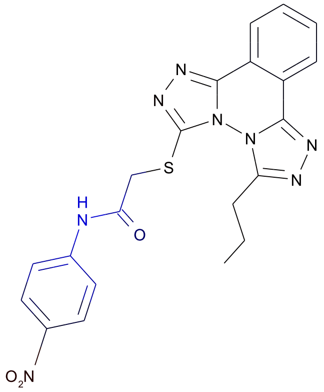

$C_{21}H_{18}N_8O_3S$

Molecular Weight: 462.48441

ALogP: 3.685

Rotatable Bonds: 7

Acceptors: 8

Donors: 1

Structural Similar Compounds

Name	2,2'-Stilbenedisulfonic acid, 4,4'-dinitro-	2-Anthracenesulfonic acid, 1-amino-9,10-dihydro-9,10-dioxo-4-(2,4,6 -trimethylanilino)-, monosodium salt	Pregna-1,4-diene-3,20-dione, 21-(acetyloxy)-11-hydroxy-6-methyl-17- (1-oxopropoxy)-, (6-alpha,11-beta)-
Structure	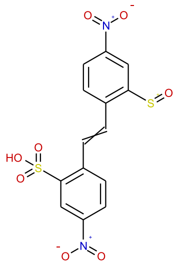	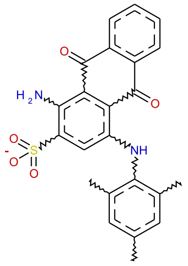	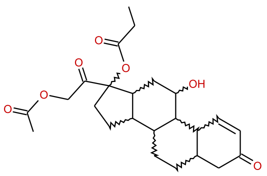
Actual Endpoint	Irritant	Irritant	Irritant
Predicted Endpoint	Irritant	Non-Irritant	Irritant
Distance	0.808	0.824	0.856
Reference	85JCAE "Prehled Prumyslove Toxikologie; Organicke Latky," Marhold, J., Prague , Czechoslovakia, Avicenum, 1986 Volume(issue)/page/year: -,1062,1986	85JCAE "Prehled Prumyslove Toxikologie; Organicke Latky," Marhold, J., Prague , Czechoslovakia, Avicenum, 1986 Volume(issue)/page/year: -,1327,1986	YACHDS Yakuri to Chiryo. Pharmacology and Therapeutics. (Raifu Saiensu Shup pan K.K., 2-5-13, Yaesu, Chuo-ku, Tokyo 104, Japan) V.1- 1972- Volume(issue) /page/year: 19,3103,1991

Model Prediction

Prediction: Non-Irritant

Probability: 0.717

Enrichment: 0.779

Bayesian Score: -3.88

Mahalanobis Distance: 8.89

Mahalanobis Distance p-value: 0.482

Prediction: Positive if the Bayesian score is above the estimated best cutoff value from minimizing the false positive and false negative rate.
Probability: The esimated probability that the sample is in the positive category. This assumes that the Bayesian score follows a normal distribution and is different from the prediction using a cutoff.
Enrichment: An estimate of enrichment, that is, the increased likelihood (versus random) of this sample being in the category.
Bayesian Score: The standard Laplacian-modified Bayesian score.
Mahalanobis Distance: The Mahalanobis distance (MD) is the distance to the center of the training data. The larger the MD, the less trustworthy the prediction.
Mahalanobis Distance p-value: The p-value gives the fraction of training data with an MD greater than or equal to the one for the given sample, assuming normally distributed data. The smaller the p-value, the less trustworthy the prediciton. For highly non-normal X properties (e.g., fingerprints), the MD p-value is wildly inaccurate.

Model Applicability

Unknown features are fingerprint features in the query molecule, but not found or appearing too infrequently in the training set.

1. All properties and OPS components are within expected ranges.
2. Unknown FCFP_2 feature: -332197802: [*][c]1:[*]:[*]:[c](:[*]):n:1:n:[*]

Feature Contribution

Top features for positive contribution

Fingerprint	Bit/Smiles	Feature Structure	Score	Irritant in training set

FCFP_12	5	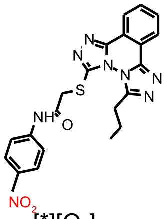 [*][O-]	0.0854	27 out of 27
FCFP_12	8	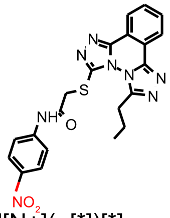 [*][N+](=[*])[*]	0.0843	20 out of 20
FCFP_12	-828984032	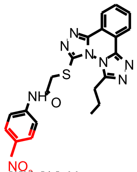 [*][N+](=[*])[c](:[cH]:[*]):[cH]:[*]	0.0795	9 out of 9
**Top Features for negative contribution**				
**Fingerprint**	**Bit/Smiles**	**Feature Structure**	**Score**	**Irritant in training set**
FCFP_12	1175665944	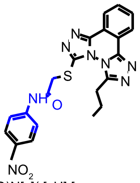 [*]CC(=O)N[c](:[cH]:[*]):[cH]:[*]	-1.02	2 out of 8
FCFP_12	-1838187238	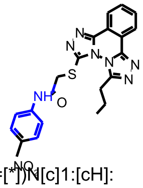 [*]C(=[*])N[c]1:[cH]: [cH]:[*]:[cH]:[cH]:1	-0.692	5 out of 12

FCFP_12	-451043714	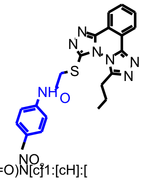 [*]CC(=O)N[c]1:[cH]:[cH]:[c]([*]):[cH]:[cH]:1	-0.65	0 out of 1

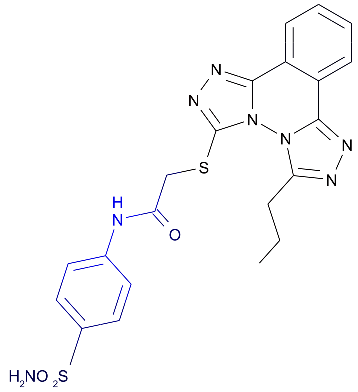

$C_{21}H_{20}N_8O_3S_2$
Molecular Weight: 496.56529
ALogP: 2.496
Rotatable Bonds: 7
Acceptors: 8
Donors: 2

Model Prediction

Prediction: Non-Irritant
Probability: 0.0519
Enrichment: 0.0563
Bayesian Score: -6.25
Mahalanobis Distance: 9.4
Mahalanobis Distance p-value: 0.229

Prediction: Positive if the Bayesian score is above the estimated best cutoff value from minimizing the false positive and false negative rate.
Probability: The esimated probability that the sample is in the positive category. This assumes that the Bayesian score follows a normal distribution and is different from the prediction using a cutoff.
Enrichment: An estimate of enrichment, that is, the increased likelihood (versus random) of this sample being in the category.
Bayesian Score: The standard Laplacian-modified Bayesian score.
Mahalanobis Distance: The Mahalanobis distance (MD) is the distance to the center of the training data. The larger the MD, the less trustworthy the prediction.
Mahalanobis Distance p-value: The p-value gives the fraction of training data with an MD greater than or equal to the one for the given sample, assuming normally distributed data. The smaller the p-value, the less trustworthy the prediciton. For highly non-normal X properties (e.g., fingerprints), the MD p-value is wildly inaccurate.

Structural Similar Compounds

Name	2,2'-Stilbenedisulfonic acid, 4,4'-dinitro-	Benzenesulfonic acid, 2,2'-(1,4-anthraquinonylenediimino)bis(5-meth yl-, disodium salt	2-Anthracenesulfonic acid, 1-amino-9,10-dihydro-9,10-dioxo-4-(2,4,6 -trimethylanilino)-, monosodium salt
Structure	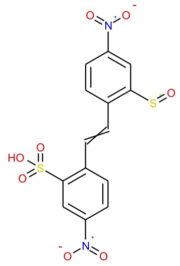	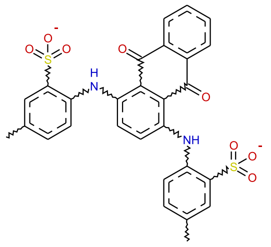	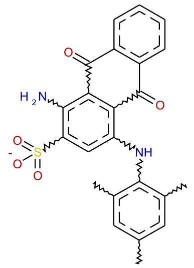
Actual Endpoint	Irritant	Irritant	Irritant
Predicted Endpoint	Irritant	Non-Irritant	Non-Irritant
Distance	0.748	0.764	0.825
Reference	85JCAE "Prehled Prumyslove Toxikologie; Organicke Latky," Marhold, J., Prague , Czechoslovakia, Avicenum, 1986 Volume(issue)/page/year: -,1062,1986	85JCAE "Prehled Prumyslove Toxikologie; Organicke Latky," Marhold, J., Prague , Czechoslovakia, Avicenum, 1986 Volume(issue)/page/year: -,1330,1986	85JCAE "Prehled Prumyslove Toxikologie; Organicke Latky," Marhold, J., Prague , Czechoslovakia, Avicenum, 1986 Volume(issue)/page/year: -,1327,1986

Model Applicability

Unknown features are fingerprint features in the query molecule, but not found or appearing too infrequently in the training set.

1. All properties and OPS components are within expected ranges.
2. Unknown FCFP_2 feature: -332197802: [*][c]1:[*]:[*]:[c](:[*]):n:1:n:[*]

Feature Contribution

Top features for positive contribution

Fingerprint	Bit/Smiles	Feature Structure	Score	Irritant in training set

FCFP_12	-1410079687	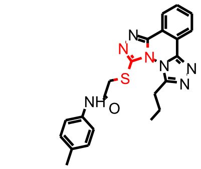 [*]S[c]1:n:[*]:[*]:n:1:[*]	0.0756	6 out of 6
FCFP_12	4427049	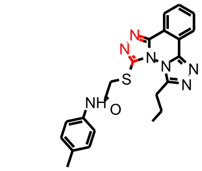 [*][c](:[*]):n:n(:[*]):[*]	0.0734	5 out of 5
FCFP_12	-1539162406	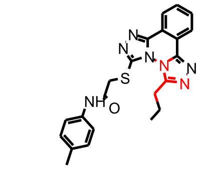 [*]C[c]1:n:[*]:[*]:n:1:[*]	0.0583	2 out of 2
**Top Features for negative contribution**				
**Fingerprint**	**Bit/Smiles**	**Feature Structure**	**Score**	**Irritant in training set**
FCFP_12	1175665944	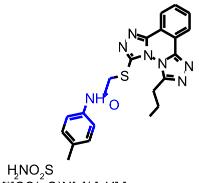 [*]CC(=O)N[c](:[cH]:[*]):[cH]:[*]	-1.02	2 out of 8
FCFP_12	-1838187238	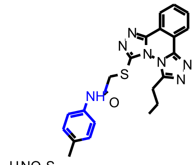 [*]C(=[*])N[c]1:[cH]:[cH]:[*]:[cH]:[cH]:1	-0.692	5 out of 12

FCFP_12	-451043714	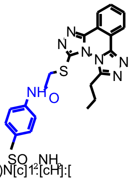 [*]CC(=O)N[c]1:[cH]:[cH]:[c]([*]):[cH]:[cH]:1	-0.65	0 out of 1

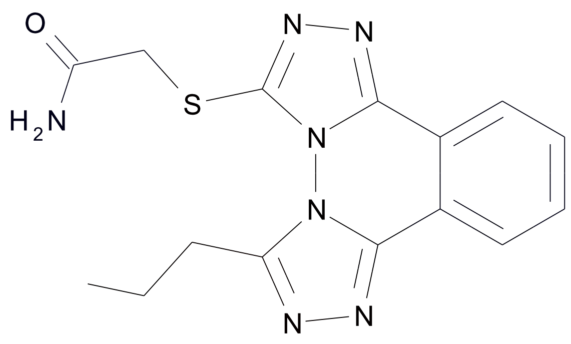

$C_{15}H_{15}N_7OS$
Molecular Weight: 341.39089
ALogP: 2.008
Rotatable Bonds: 5
Acceptors: 6
Donors: 1

Model Prediction

Prediction: Non-Irritant
Probability: 0.972
Enrichment: 1.06
Bayesian Score: -0.862
Mahalanobis Distance: 7.82
Mahalanobis Distance p-value: 0.926

Prediction: Positive if the Bayesian score is above the estimated best cutoff value from minimizing the false positive and false negative rate.
Probability: The esimated probability that the sample is in the positive category. This assumes that the Bayesian score follows a normal distribution and is different from the prediction using a cutoff.
Enrichment: An estimate of enrichment, that is, the increased likelihood (versus random) of this sample being in the category.
Bayesian Score: The standard Laplacian-modified Bayesian score.
Mahalanobis Distance: The Mahalanobis distance (MD) is the distance to the center of the training data. The larger the MD, the less trustworthy the prediction.
Mahalanobis Distance p-value: The p-value gives the fraction of training data with an MD greater than or equal to the one for the given sample, assuming normally distributed data. The smaller the p-value, the less trustworthy the prediciton. For highly non-normal X properties (e.g., fingerprints), the MD p-value is wildly inaccurate.

Structural Similar Compounds

Name	Benzenesulfonic acid, 2-anilino-5-nitro-	Benzenesulfonamide, 4-amino-N-(5,6-dimethoxy-4-pyrimidinyl)-	Acetic acid, 2-(sec-butyl)-4,6-dinitrophenyl ester
Structure	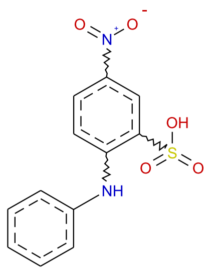	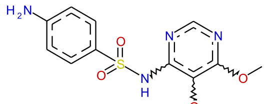	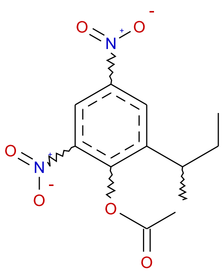
Actual Endpoint	Irritant	Irritant	Irritant
Predicted Endpoint	Non-Irritant	Non-Irritant	Non-Irritant
Distance	0.716	0.727	0.728
Reference	85JCAE "Prehled Prumyslove Toxikologie; Organicke Latky," Marhold, J., Prague , Czechoslovakia, Avicenum, 1986 Volume(issue)/page/year: -,1061,1986	FCTXAV 14,307,76	85JCAE "Prehled Prumyslove Toxikologie; Organicke Latky," Marhold, J., Prague , Czechoslovakia, Avicenum, 1986 Volume(issue)/page/year: -,750,1986

Model Applicability

Unknown features are fingerprint features in the query molecule, but not found or appearing too infrequently in the training set.

1. All properties and OPS components are within expected ranges.
2. Unknown FCFP_2 feature: -332197802: [*][c]1:[*]:[*]:[c](:[*]):n:1:n:[*]

Feature Contribution

Top features for positive contribution				
Fingerprint	Bit/Smiles	Feature Structure	Score	Irritant in training set

FCFP_12	-1410079687	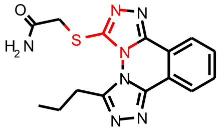 [*]S[c]1:n:[*]:[*]:n:1:[*]	0.0756	6 out of 6
FCFP_12	4427049	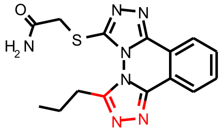 [*][c](:[*]):n:n(:[*]):[*]	0.0734	5 out of 5
FCFP_12	-1578026142	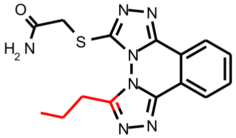 [*]:[c](:[*])CCC	0.0583	2 out of 2
**Top Features for negative contribution**				
**Fingerprint**	**Bit/Smiles**	**Feature Structure**	**Score**	**Irritant in training set**
FCFP_12	566058135	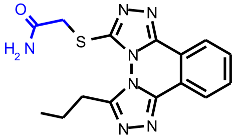 [*]CC(=O)N[*]	-0.367	13 out of 21
FCFP_12	-1320007763	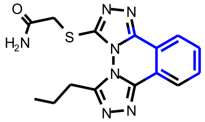 [*][c](:[*]):[c]1:[cH]:[cH]:[cH]:[*]:[c]:1:[*]	-0.0893	20 out of 24

FCFP_12	1618154665	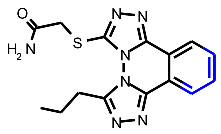 [*]:[cH]:[cH]:[cH]:[*<br>]	-0.0845	412 out of 490

Doxorubicin

TOPKAT_Skin_Irritancy_None_vs_Irritant

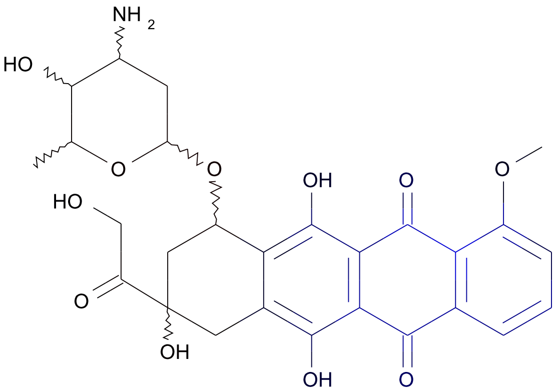

$C_{27}H_{29}NO_{11}$

Molecular Weight: 543.51925

ALogP: -4.4e-002

Rotatable Bonds: 5

Acceptors: 12

Donors: 6

Structural Similar Compounds

Name	1,3,6-Naphthalenetrisulfonic acid, 7-amino-	Amipurimycin, hydrate	2,2'-Benzidine disulfonic acid
Structure	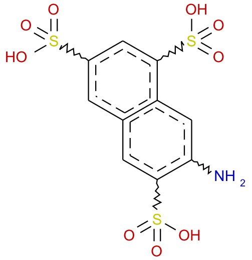	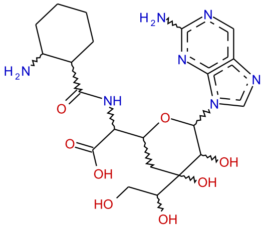	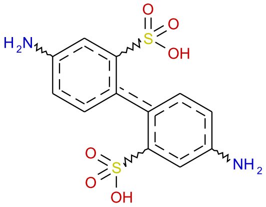
Actual Endpoint	Irritant	Irritant	Non-Irritant
Predicted Endpoint	Non-Irritant	Non-Irritant	Non-Irritant
Distance	1.158	1.186	1.233
Reference	85JCAE "Prehled Prumyslove Toxikologie; Organicke Latky," Marhold, J., Prague , Czechoslovakia, Avicenum, 1986 Volume(issue)/page/year: -,1058,1986	JANTAJ Journal of Antibiotics. (Japan Antibiotics Research Assoc., 2-20-8 Ka miosaki, Shinagawa-ku, Tokyo, 141, Japan) V.2-5, 1948-52; V.21- 1968- Volume(i ssue)/page/year: 30,1,1977	28ZPAK -,191,72

Model Prediction

Prediction: Non-Irritant

Probability: 0.464

Enrichment: 0.503

Bayesian Score: -4.65

Mahalanobis Distance: 14.4

Mahalanobis Distance p-value: 5.01e-012

Prediction: Positive if the Bayesian score is above the estimated best cutoff value from minimizing the false positive and false negative rate.
Probability: The esimated probability that the sample is in the positive category. This assumes that the Bayesian score follows a normal distribution and is different from the prediction using a cutoff.
Enrichment: An estimate of enrichment, that is, the increased likelihood (versus random) of this sample being in the category.
Bayesian Score: The standard Laplacian-modified Bayesian score.
Mahalanobis Distance: The Mahalanobis distance (MD) is the distance to the center of the training data. The larger the MD, the less trustworthy the prediction.
Mahalanobis Distance p-value: The p-value gives the fraction of training data with an MD greater than or equal to the one for the given sample, assuming normally distributed data. The smaller the p-value, the less trustworthy the prediciton. For highly non-normal X properties (e.g., fingerprints), the MD p-value is wildly inaccurate.

Model Applicability

Unknown features are fingerprint features in the query molecule, but not found or appearing too infrequently in the training set.

1. All properties and OPS components are within expected ranges.

Feature Contribution

Top features for positive contribution

Fingerprint	Bit/Smiles	Feature Structure	Score	Irritant in training set

FCFP_12	-415156552	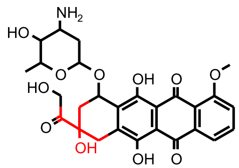 [*]CC(O)(C[*])C(=[*])[*]	0.0854	27 out of 27
FCFP_12	699500266	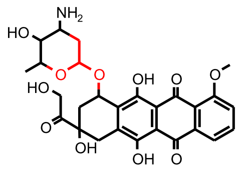 [*]CC(O[*])O[*]	0.0852	25 out of 25
FCFP_12	-1804899926	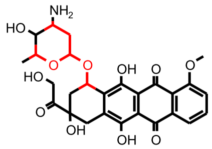 [*]C([*])OC1CC([*])[*]C([*])O1	0.0795	9 out of 9

Top Features for negative contribution

Fingerprint	Bit/Smiles	Feature Structure	Score	Irritant in training set
FCFP_12	979221427	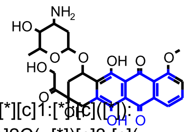 [*][c]1:[*]:[c]([*]):[c]2C(=[*])[c]3:[c]([*]):[*]:[cH]:[cH]:[c]:3C(=O)[c]:2:[c]:1O	-0.846	1 out of 4
FCFP_12	523781771	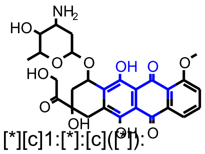 [*][c]1:[*]:[c]([*]):[c]2C(=[*])[*]:[c](:[*])C(=O)[c]:2:[c]:1O	-0.732	3 out of 8

FCFP_12	1392625818	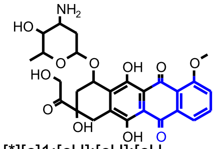 [*][c]1:[cH]:[cH]:[cH]:[c]2C(=O)[c](:[*]):[*]C(=[*])[c]:1:2	-0.558	9 out of 18

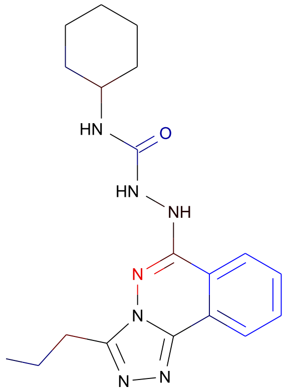

$C_{19}H_{25}N_7O$
Molecular Weight: 367.44809
ALogP: 3.681
Rotatable Bonds: 5
Acceptors: 5
Donors: 3

Model Prediction

Prediction: 8.1
Unit: mg/kg_body_weight/day
Mahalanobis Distance: 11.2
Mahalanobis Distance p-value: 0.000779

Mahalanobis Distance: The Mahalanobis distance (MD) is a generalization of the Euclidean distance that accounts for correlations among the X properties. It is calculated as the distance to the center of the training data. The larger the MD, the less trustworthy the prediction.
Mahalanobis Distance p-value: The p-value gives the fraction of training data with an MD greater than or equal to the one for the given sample, assuming normally distributed data. The smaller the p-value, the less trustworthy the prediciton. For highly non-normal X properties (e.g., fingerprints), the MD p-value is wildly inaccurate.

Structural Similar Compounds

Name	542	Ochratoxin A	Phenolphthalein
Structure	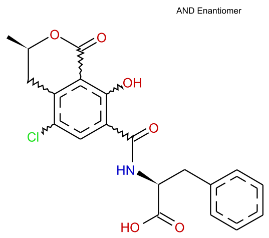	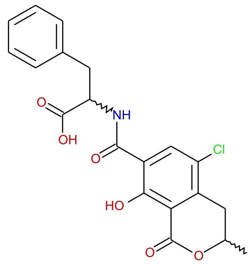	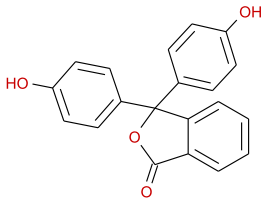
Actual Endpoint (-log C)	4.79932	4.79932	2.43468
Predicted Endpoint (-log C)	3.6353	3.6353	3.66084
Distance	0.699	0.699	0.703
Reference	CPDB	CPDB	CPDB

Model Applicability

Unknown features are fingerprint features in the query molecule, but not found or appearing too infrequently in the training set.

1. All properties and OPS components are within expected ranges.
2. Unknown ECFP_2 feature: -1507082173: [*][c]1:[*]:[*]:[c](:[*]):n:1:n:[*]
3. Unknown ECFP_2 feature: -435188506: [*]C[c]1:n:[*]:[*]:n:1:[*]

Feature Contribution

Top features for positive contribution

Fingerprint	Bit/Smiles	Feature Structure	Score
ECFP_6	655739385	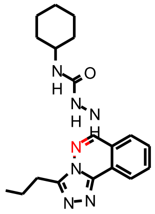 [*]:n:[*]	0.229

ECFP_6	1559650422	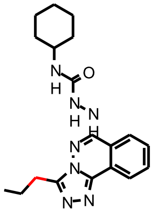 [*]C[*]	0.203
ECFP_6	1333660716	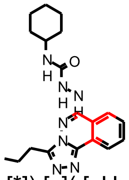 [*][c](:[*]):[c](:[cH]:[*]):[c](:[*]):[*]	0.0746
**Top Features for negative contribution**			
**Fingerprint**	**Bit/Smiles**	**Feature Structure**	**Score**
ECFP_6	1996767644	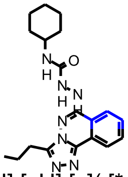 [*]:[cH]:[cH]:[c](:[*]):[*]	-0.251
ECFP_6	642810091	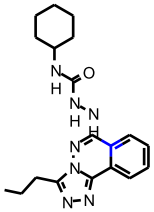 [*]:[c](:[*]):[*]	-0.247
ECFP_6	-182236392	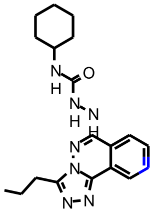 [*]:[cH]:[*]	-0.232

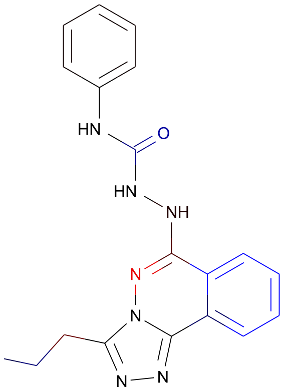

$C_{19}H_{19}N_7O$
Molecular Weight: 361.40046
ALogP: 3.399
Rotatable Bonds: 5
Acceptors: 5
Donors: 3

Model Prediction

Prediction: 7.62
Unit: mg/kg_body_weight/day
Mahalanobis Distance: 11.3
Mahalanobis Distance p-value: 0.000436

Mahalanobis Distance: The Mahalanobis distance (MD) is a generalization of the Euclidean distance that accounts for correlations among the X properties. It is calculated as the distance to the center of the training data. The larger the MD, the less trustworthy the prediction.
Mahalanobis Distance p-value: The p-value gives the fraction of training data with an MD greater than or equal to the one for the given sample, assuming normally distributed data. The smaller the p-value, the less trustworthy the prediciton. For highly non-normal X properties (e.g., fingerprints), the MD p-value is wildly inaccurate.

Structural Similar Compounds

Name	Phenolphthalein	646	542
Structure	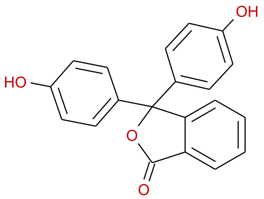	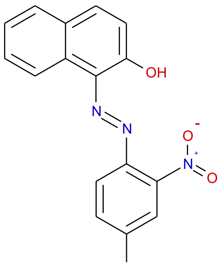	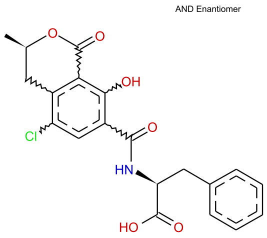
Actual Endpoint (-log C)	2.43468	0.937339	4.79932
Predicted Endpoint (-log C)	3.66084	3.26294	3.6353
Distance	0.751	0.813	0.818
Reference	CPDB	CPDB	CPDB

Model Applicability

Unknown features are fingerprint features in the query molecule, but not found or appearing too infrequently in the training set.

1. All properties and OPS components are within expected ranges.
2. Unknown ECFP_2 feature: -1507082173: [*][c]1:[*]:[*]:[c](:[*]):n:1:n:[*]
3. Unknown ECFP_2 feature: -435188506: [*]C[c]1:n:[*]:[*]:n:1:[*]

Feature Contribution

Top features for positive contribution

Fingerprint	Bit/Smiles	Feature Structure	Score
ECFP_6	655739385	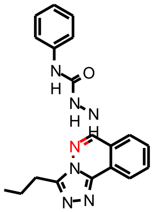 [*]:n:[*]	0.229

ECFP_6	1559650422	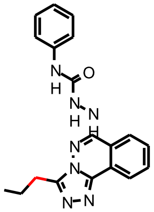 [*]C[*]	0.203
ECFP_6	1333660716	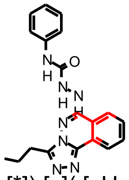 [*][c](:[*]):[c](:[cH]:[*]):[c](:[*]):[*]	0.0746
**Top Features for negative contribution**			
**Fingerprint**	**Bit/Smiles**	**Feature Structure**	**Score**
ECFP_6	1996767644	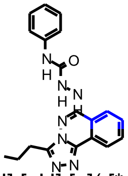 [*]:[cH]:[cH]:[c](:[*]):[*]	-0.251
ECFP_6	642810091	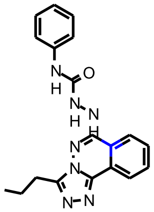 [*]:[c](:[*]):[*]	-0.247
ECFP_6	-182236392	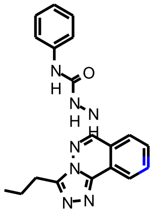 [*]:[cH]:[*]	-0.232

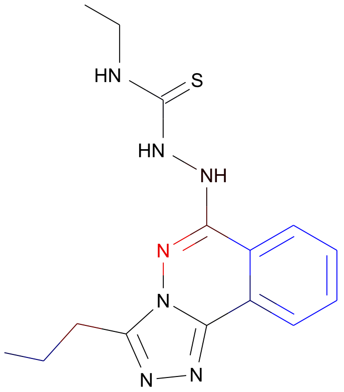

$C_{15}H_{19}N_7S$

Molecular Weight: 329.42325

ALogP: 3.388

Rotatable Bonds: 7

Acceptors: 5

Donors: 3

Model Prediction

Prediction: 18.5

Unit: mg/kg_body_weight/day

Mahalanobis Distance: 11.3

Mahalanobis Distance p-value: 0.000422

Mahalanobis Distance: The Mahalanobis distance (MD) is a generalization of the Euclidean distance that accounts for correlations among the X properties. It is calculated as the distance to the center of the training data. The larger the MD, the less trustworthy the prediction.
Mahalanobis Distance p-value: The p-value gives the fraction of training data with an MD greater than or equal to the one for the given sample, assuming normally distributed data. The smaller the p-value, the less trustworthy the prediciton. For highly non-normal X properties (e.g., fingerprints), the MD p-value is wildly inaccurate.

Structural Similar Compounds

Name	4-Chloro-6-(2,3-xylidino)-2-pyri-mi-dinylthio(N-b-hydroxy-ethyl) acetamide	542	Ochratoxin A
Structure	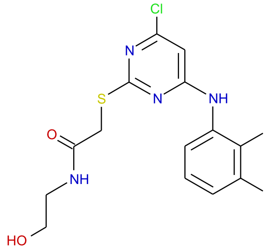	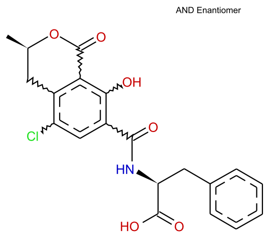	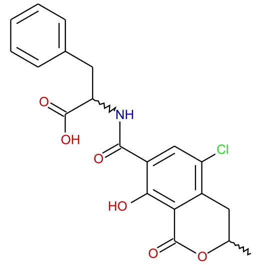
Actual Endpoint (-log C)	3.91517	4.79932	4.79932
Predicted Endpoint (-log C)	3.92186	3.6353	3.6353
Distance	0.669	0.683	0.683
Reference	CPDB	CPDB	CPDB

Model Applicability

Unknown features are fingerprint features in the query molecule, but not found or appearing too infrequently in the training set.

1. All properties and OPS components are within expected ranges.
2. Unknown ECFP_2 feature: -1507082173: [*][c]1:[*]:[*]:[c](:[*]):n:1:n:[*]
3. Unknown ECFP_2 feature: -435188506: [*]C[c]1:n:[*]:[*]:n:1:[*]
4. Unknown ECFP_2 feature: 150794520: [*]NC(=S)N[*]

Feature Contribution

Top features for positive contribution

Fingerprint	Bit/Smiles	Feature Structure	Score
ECFP_6	655739385	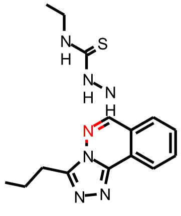 [*]:n:[*]	0.229

ECFP_6	1559650422	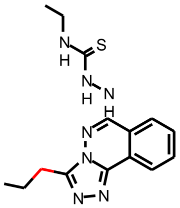 [*]C[*]	0.203
ECFP_6	1333660716	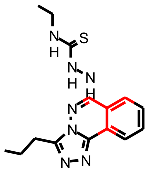 [*][c](:[*]):[c](:[cH]:[*]):[c](:[*]):[*]	0.0746
**Top Features for negative contribution**			
**Fingerprint**	**Bit/Smiles**	**Feature Structure**	**Score**
ECFP_6	1996767644	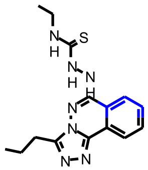 [*]:[cH]:[cH]:[c](:[*]):[*]	-0.251
ECFP_6	642810091	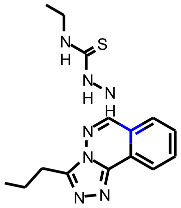 [*]:[c](:[*]):[*]	-0.247
ECFP_6	-182236392	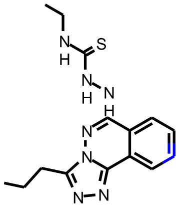 [*]:[cH]:[*]	-0.232

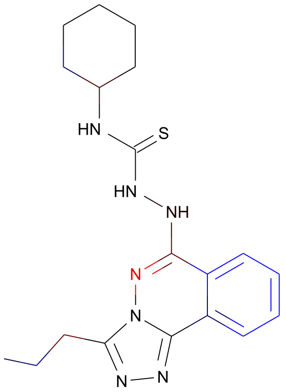

$C_{19}H_{25}N_7S$
Molecular Weight: 383.51369
ALogP: 4.898
Rotatable Bonds: 7
Acceptors: 5
Donors: 3

Model Prediction

Prediction: 6.43
Unit: mg/kg_body_weight/day
Mahalanobis Distance: 12.6
Mahalanobis Distance p-value: 9.23e-007

Mahalanobis Distance: The Mahalanobis distance (MD) is a generalization of the Euclidean distance that accounts for correlations among the X properties. It is calculated as the distance to the center of the training data. The larger the MD, the less trustworthy the prediction.
Mahalanobis Distance p-value: The p-value gives the fraction of training data with an MD greater than or equal to the one for the given sample, assuming normally distributed data. The smaller the p-value, the less trustworthy the prediciton. For highly non-normal X properties (e.g., fingerprints), the MD p-value is wildly inaccurate.

Structural Similar Compounds

Name	542	Ochratoxin A	4-Chloro-6-(2,3-xylidino)-2-pyri-mi-dinylthio(N-b-hydroxy-ethyl) acetamide
Structure	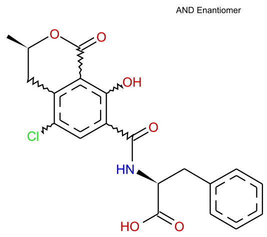	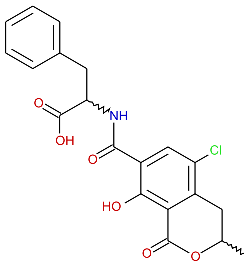	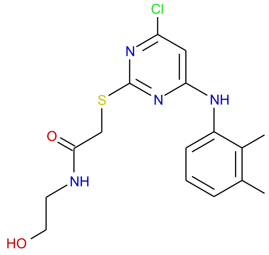
Actual Endpoint (-log C)	4.79932	4.79932	3.91517
Predicted Endpoint (-log C)	3.6353	3.6353	3.92186
Distance	0.716	0.716	0.764
Reference	CPDB	CPDB	CPDB

Model Applicability

Unknown features are fingerprint features in the query molecule, but not found or appearing too infrequently in the training set.

1. All properties and OPS components are within expected ranges.
2. Unknown ECFP_2 feature: -1507082173: [*][c]1:[*]:[*]:[c](:[*]):n:1:n:[*]
3. Unknown ECFP_2 feature: -435188506: [*]C[c]1:n:[*]:[*]:n:1:[*]
4. Unknown ECFP_2 feature: 150794520: [*]NC(=S)N[*]

Feature Contribution

Top features for positive contribution

Fingerprint	Bit/Smiles	Feature Structure	Score
ECFP_6	655739385	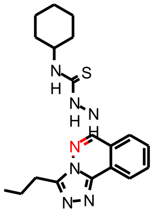 [*]:n:[*]	0.229

ECFP_6	1559650422	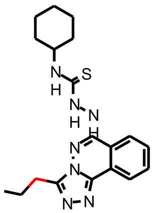 [*]C[*]	0.203
ECFP_6	1333660716	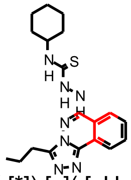 [*][c](:[*]):[c](:[cH]:[*]):[c](:[*]):[*]	0.0746

Top Features for negative contribution

Fingerprint	Bit/Smiles	Feature Structure	Score
ECFP_6	1996767644	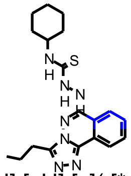 [*]:[cH]:[cH]:[c](:[*]):[*]	-0.251
ECFP_6	642810091	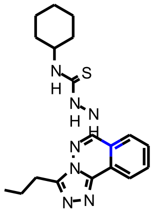 [*]:[c](:[*]):[*]	-0.247
ECFP_6	-182236392	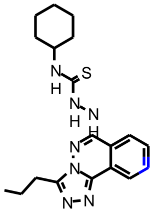 [*]:[cH]:[*]	-0.232

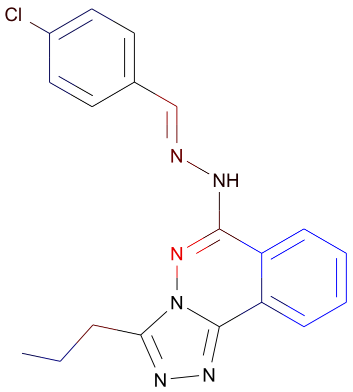

$C_{19}H_{17}ClN_6$

Molecular Weight: 364.83147

ALogP: 4.967

Rotatable Bonds: 5

Acceptors: 5

Donors: 1

Model Prediction

Prediction: 3.41

Unit: mg/kg_body_weight/day

Mahalanobis Distance: 13.3

Mahalanobis Distance p-value: 1.93e-008

Mahalanobis Distance: The Mahalanobis distance (MD) is a generalization of the Euclidean distance that accounts for correlations among the X properties. It is calculated as the distance to the center of the training data. The larger the MD, the less trustworthy the prediction.
Mahalanobis Distance p-value: The p-value gives the fraction of training data with an MD greater than or equal to the one for the given sample, assuming normally distributed data. The smaller the p-value, the less trustworthy the prediciton. For highly non-normal X properties (e.g., fingerprints), the MD p-value is wildly inaccurate.

Structural Similar Compounds

Name	646	Phenolphthalein	C.I. pigment red 3
Structure	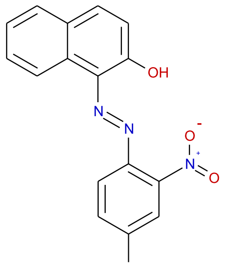	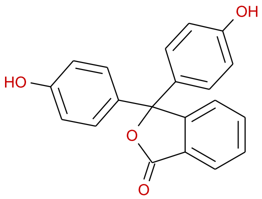	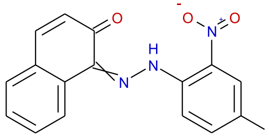
Actual Endpoint (-log C)	0.937339	2.43468	0.937339
Predicted Endpoint (-log C)	3.26294	3.66084	3.17837
Distance	0.726	0.754	0.847
Reference	CPDB	CPDB	CPDB

Model Applicability

Unknown features are fingerprint features in the query molecule, but not found or appearing too infrequently in the training set.

1. OPS PC24 out of range. Value: -4.6354. Training min, max, SD, explained variance: -4.4826, 3.8729, 1.034, 0.0133.
2. OPS PC26 out of range. Value: -3.6943. Training min, max, SD, explained variance: -2.9667, 3.5042, 1.009, 0.0127.
3. Unknown ECFP_2 feature: -1507082173: [*][c]1:[*]:[*]:[c](:[*]):n:1:n:[*]
4. Unknown ECFP_2 feature: -435188506: [*]C[c]1:n:[*]:[*]:n:1:[*]

Feature Contribution

Top features for positive contribution			
**Fingerprint**	**Bit/Smiles**	**Feature Structure**	**Score**
ECFP_6	655739385	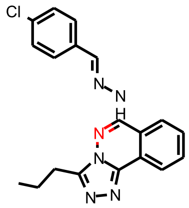 [*]:n:[*]	0.229

ECFP_6	1559650422	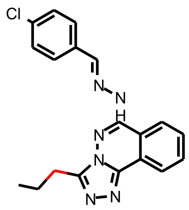 [*]C[*]	0.203
ECFP_6	-1925046727	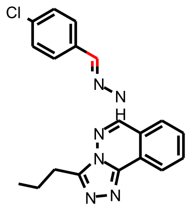 [*]C=[*]	0.145
**Top Features for negative contribution**			
**Fingerprint**	**Bit/Smiles**	**Feature Structure**	**Score**
ECFP_6	1996767644	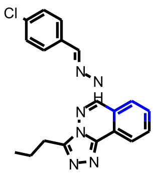 [*]:[cH]:[cH]:[c](:[*]):[*]	-0.251
ECFP_6	642810091	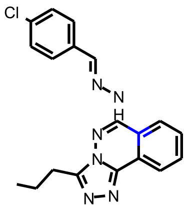 [*]:[c](:[*]):[*]	-0.247
ECFP_6	-182236392	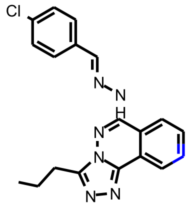 [*]:[cH]:[*]	-0.232

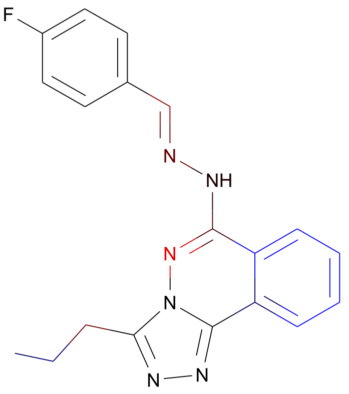

$C_{19}H_{17}FN_6$

Molecular Weight: 348.37688

ALogP: 4.508

Rotatable Bonds: 5

Acceptors: 5

Donors: 1

Model Prediction

Prediction: 4.41

Unit: mg/kg_body_weight/day

Mahalanobis Distance: 11.7

Mahalanobis Distance p-value: 8.38e-005

Mahalanobis Distance: The Mahalanobis distance (MD) is a generalization of the Euclidean distance that accounts for correlations among the X properties. It is calculated as the distance to the center of the training data. The larger the MD, the less trustworthy the prediction.
Mahalanobis Distance p-value: The p-value gives the fraction of training data with an MD greater than or equal to the one for the given sample, assuming normally distributed data. The smaller the p-value, the less trustworthy the prediciton. For highly non-normal X properties (e.g., fingerprints), the MD p-value is wildly inaccurate.

Structural Similar Compounds

Name	646	Phenolphthalein	C.I. pigment red 3
Structure	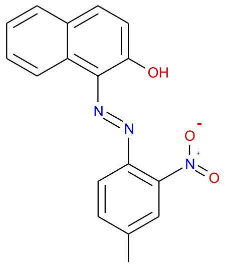	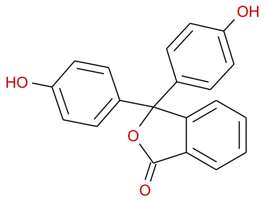	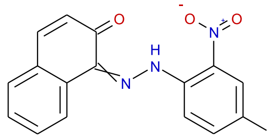
Actual Endpoint (-log C)	0.937339	2.43468	0.937339
Predicted Endpoint (-log C)	3.26294	3.66084	3.17837
Distance	0.726	0.744	0.840
Reference	CPDB	CPDB	CPDB

Model Applicability

Unknown features are fingerprint features in the query molecule, but not found or appearing too infrequently in the training set.

1. All properties and OPS components are within expected ranges.
2. Unknown ECFP_2 feature: -1507082173: [*][c]1:[*]:[*]:[c](:[*]):n:1:n:[*]
3. Unknown ECFP_2 feature: -435188506: [*]C[c]1:n:[*]:[*]:n:1:[*]

Feature Contribution

Top features for positive contribution			
Fingerprint	Bit/Smiles	Feature Structure	Score
ECFP_6	655739385	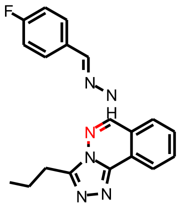 [*]:n:[*]	0.229

ECFP_6	1559650422	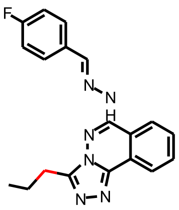 [*]C[*]	0.203
ECFP_6	-1925046727	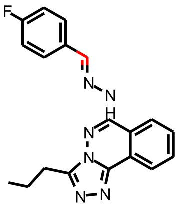 [*]C=[*]	0.145

Top Features for negative contribution

Fingerprint	Bit/Smiles	Feature Structure	Score
ECFP_6	1996767644	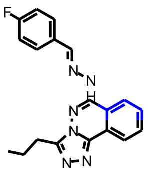 [*]:[cH]:[cH]:[c](:[*]):[*]	-0.251
ECFP_6	642810091	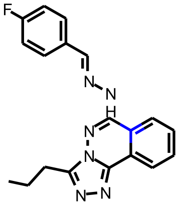 [*]:[c](:[*]):[*]	-0.247
ECFP_6	-182236392	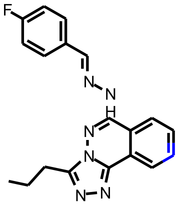 [*]:[cH]:[*]	-0.232

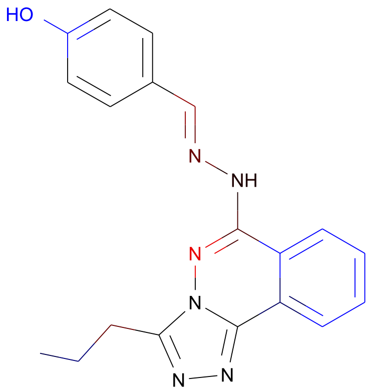

$C_{19}H_{18}N_6O$
Molecular Weight: 346.38582
ALogP: 4.061
Rotatable Bonds: 5
Acceptors: 6
Donors: 2

Model Prediction

Prediction: 10.5
Unit: mg/kg_body_weight/day
Mahalanobis Distance: 12.6
Mahalanobis Distance p-value: 1.12e-006

Mahalanobis Distance: The Mahalanobis distance (MD) is a generalization of the Euclidean distance that accounts for correlations among the X properties. It is calculated as the distance to the center of the training data. The larger the MD, the less trustworthy the prediction.
Mahalanobis Distance p-value: The p-value gives the fraction of training data with an MD greater than or equal to the one for the given sample, assuming normally distributed data. The smaller the p-value, the less trustworthy the prediciton. For highly non-normal X properties (e.g., fingerprints), the MD p-value is wildly inaccurate.

Structural Similar Compounds

Name	Phenolphthalein	646	C.I. pigment red 3
Structure	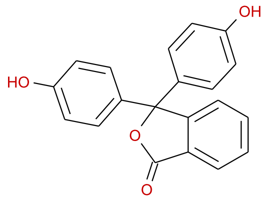	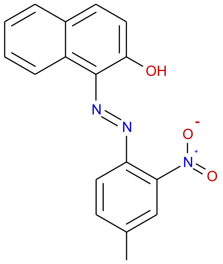	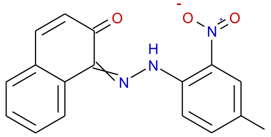
Actual Endpoint (-log C)	2.43468	0.937339	0.937339
Predicted Endpoint (-log C)	3.66084	3.26294	3.17837
Distance	0.706	0.725	0.842
Reference	CPDB	CPDB	CPDB

Model Applicability

Unknown features are fingerprint features in the query molecule, but not found or appearing too infrequently in the training set.

1. All properties and OPS components are within expected ranges.
2. Unknown ECFP_2 feature: -1507082173: [*][c]1:[*]:[*]:[c](:[*]):n:1:n:[*]
3. Unknown ECFP_2 feature: -435188506: [*]C[c]1:n:[*]:[*]:n:1:[*]

Feature Contribution

Top features for positive contribution

Fingerprint	Bit/Smiles	Feature Structure	Score
ECFP_6	655739385	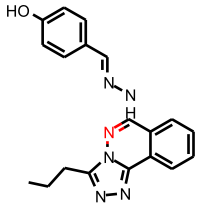 [*]:n:[*]	0.229

ECFP_6	1559650422	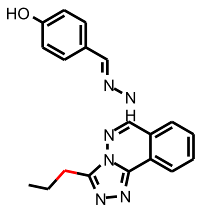 [*]C[*]	0.203
ECFP_6	-1925046727	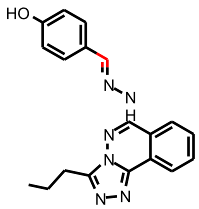 [*]C=[*]	0.145
**Top Features for negative contribution**			
**Fingerprint**	**Bit/Smiles**	**Feature Structure**	**Score**
ECFP_6	2019062761	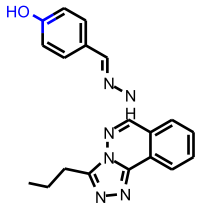 [*]:[c](:[*])O	-0.258
ECFP_6	1996767644	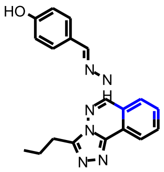 [*]:[cH]:[cH]:[c](:[*]):[*]	-0.251
ECFP_6	642810091	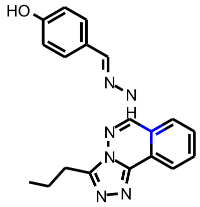 [*]:[c](:[*]):[*]	-0.247

TOPKAT_Carcinogenic_Potency_TD50_Mouse

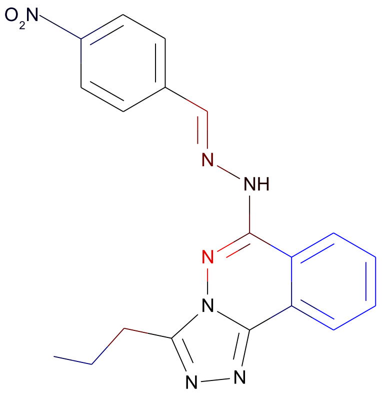

$C_{19}H_{17}N_7O_2$

Molecular Weight: 375.38397

ALogP: 4.197

Rotatable Bonds: 6

Acceptors: 7

Donors: 1

Model Prediction

Prediction: 5.83

Unit: mg/kg_body_weight/day

Mahalanobis Distance: 11.8

Mahalanobis Distance p-value: 4.31e-005

Mahalanobis Distance: The Mahalanobis distance (MD) is a generalization of the Euclidean distance that accounts for correlations among the X properties. It is calculated as the distance to the center of the training data. The larger the MD, the less trustworthy the prediction.
Mahalanobis Distance p-value: The p-value gives the fraction of training data with an MD greater than or equal to the one for the given sample, assuming normally distributed data. The smaller the p-value, the less trustworthy the prediciton. For highly non-normal X properties (e.g., fingerprints), the MD p-value is wildly inaccurate.

Structural Similar Compounds

Name	646	Azathioprine s	470
Structure	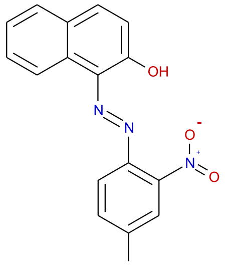	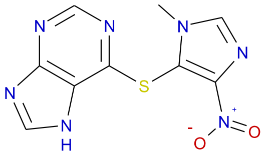	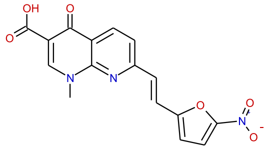
Actual Endpoint (-log C)	0.937339	4.49253	4.62839
Predicted Endpoint (-log C)	3.26294	4.28607	3.93264
Distance	0.738	0.827	0.842
Reference	CPDB	CPDB	CPDB

Model Applicability

Unknown features are fingerprint features in the query molecule, but not found or appearing too infrequently in the training set.

1. All properties and OPS components are within expected ranges.
2. Unknown ECFP_2 feature: -1507082173: [*][c]1:[*]:[*]:[c](:[*]):n:1:n:[*]
3. Unknown ECFP_2 feature: -435188506: [*]C[c]1:n:[*]:[*]:n:1:[*]

Feature Contribution

Top features for positive contribution

Fingerprint	Bit/Smiles	Feature Structure	Score
ECFP_6	655739385	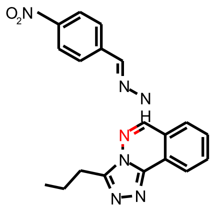 [*]:n:[*]	0.229

ECFP_6	1559650422	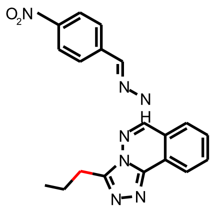 [*]C[*]	0.203
ECFP_6	-1925046727	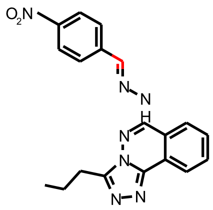 [*]C=[*]	0.145

Top Features for negative contribution

Fingerprint	Bit/Smiles	Feature Structure	Score
ECFP_6	1996767644	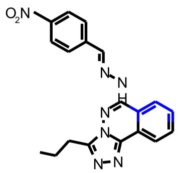 [*]:[cH]:[cH]:[c](:[*<br>]):[*]	-0.251
ECFP_6	642810091	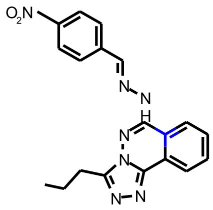 [*]:[c](:[*]):[*]	-0.247
ECFP_6	-182236392	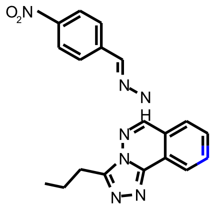 [*]:[cH]:[*]	-0.232

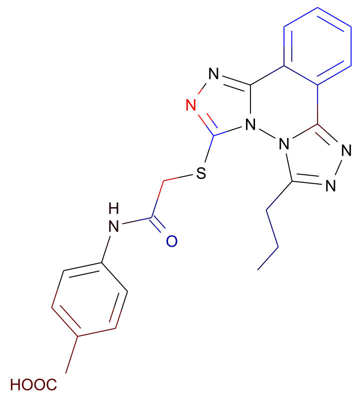

$C_{22}H_{19}N_7O_3S$

Molecular Weight: 461.49635

ALogP: 3.42

Rotatable Bonds: 7

Acceptors: 8

Donors: 2

Model Prediction

Prediction: 8.9

Unit: mg/kg_body_weight/day

Mahalanobis Distance: 12.3

Mahalanobis Distance p-value: 5.52e-006

Mahalanobis Distance: The Mahalanobis distance (MD) is a generalization of the Euclidean distance that accounts for correlations among the X properties. It is calculated as the distance to the center of the training data. The larger the MD, the less trustworthy the prediction.

Mahalanobis Distance p-value: The p-value gives the fraction of training data with an MD greater than or equal to the one for the given sample, assuming normally distributed data. The smaller the p-value, the less trustworthy the prediciton. For highly non-normal X properties (e.g., fingerprints), the MD p-value is wildly inaccurate.

Structural Similar Compounds

Name	223	420	Salicylazosulfapyridine
Structure	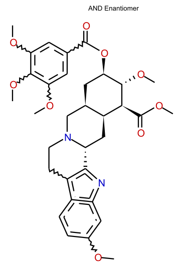	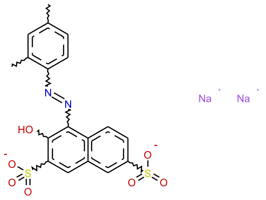	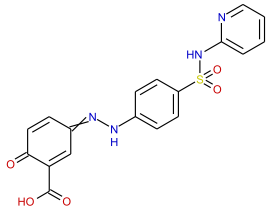
Actual Endpoint (-log C)	5.08368	2.78302	2.5034
Predicted Endpoint (-log C)	5.08273	3.31546	3.54214
Distance	0.966	0.993	0.997
Reference	CPDB	CPDB	CPDB

Model Applicability

Unknown features are fingerprint features in the query molecule, but not found or appearing too infrequently in the training set.

1. All properties and OPS components are within expected ranges.
2. Unknown ECFP_2 feature: 1986731747: [*]S[c]1:n:[*]:[*]:n:1:[*]
3. Unknown ECFP_2 feature: -435188506: [*]C[c]1:n:[*]:[*]:n:1:[*]
4. Unknown ECFP_2 feature: 78665610: [*][c]1:[*]:[*]:[c](:[*]):n:1:n(:[*]):[*]

Feature Contribution

Top features for positive contribution

Fingerprint	Bit/Smiles	Feature Structure	Score
ECFP_6	655739385	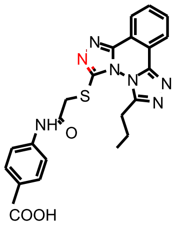 [*]:n:[*]	0.229

ECFP_6	1559650422	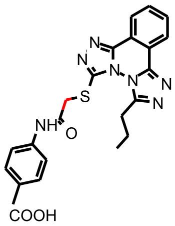 [*]C[*]	0.203
ECFP_6	-175146122	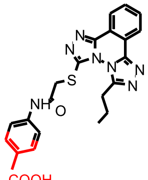 [*]C(=[*])[c](:[cH]:[*]):[cH]:[*]	0.107
**Top Features for negative contribution**			
**Fingerprint**	**Bit/Smiles**	**Feature Structure**	**Score**
ECFP_6	1996767644	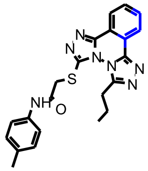 [*]:[cH]:[cH]:[c](:[*]):[*]	-0.251
ECFP_6	642810091	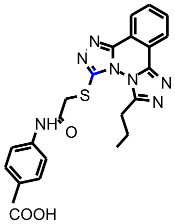 [*]:[c](:[*]):[*]	-0.247
ECFP_6	-182236392	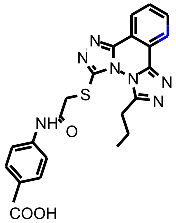 [*]:[cH]:[*]	-0.232

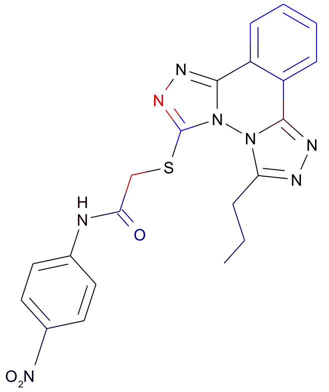

$C_{21}H_{18}N_8O_3S$
Molecular Weight: 462.48441
ALogP: 3.685
Rotatable Bonds: 7
Acceptors: 8
Donors: 1

Model Prediction

Prediction: 8.57
Unit: mg/kg_body_weight/day
Mahalanobis Distance: 11.7
Mahalanobis Distance p-value: 9.42e-005

Mahalanobis Distance: The Mahalanobis distance (MD) is a generalization of the Euclidean distance that accounts for correlations among the X properties. It is calculated as the distance to the center of the training data. The larger the MD, the less trustworthy the prediction.
Mahalanobis Distance p-value: The p-value gives the fraction of training data with an MD greater than or equal to the one for the given sample, assuming normally distributed data. The smaller the p-value, the less trustworthy the prediciton. For highly non-normal X properties (e.g., fingerprints), the MD p-value is wildly inaccurate.

Structural Similar Compounds

Name	223	Azathioprine s	420
Structure	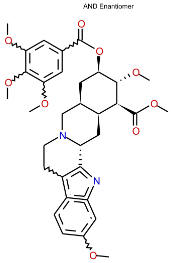	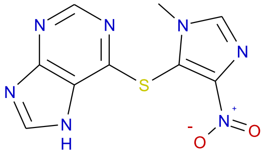	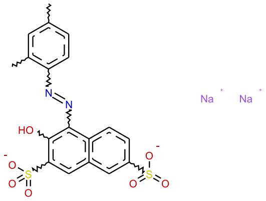
Actual Endpoint (-log C)	5.08368	4.49253	2.78302
Predicted Endpoint (-log C)	5.08273	4.28607	3.31546
Distance	0.968	0.975	0.979
Reference	CPDB	CPDB	CPDB

Model Applicability

Unknown features are fingerprint features in the query molecule, but not found or appearing too infrequently in the training set.

1. All properties and OPS components are within expected ranges.
2. Unknown ECFP_2 feature: 1986731747: [*]S[c]1:n:[*]:[*]:n:1:[*]
3. Unknown ECFP_2 feature: -435188506: [*]C[c]1:n:[*]:[*]:n:1:[*]
4. Unknown ECFP_2 feature: 78665610: [*][c]1:[*]:[*]:[c](:[*]):n:1:n(:[*]):[*]

Feature Contribution

Top features for positive contribution			
Fingerprint	Bit/Smiles	Feature Structure	Score
ECFP_6	655739385	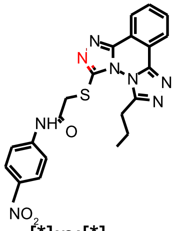 [*]:n:[*]	0.229

ECFP_6	1559650422	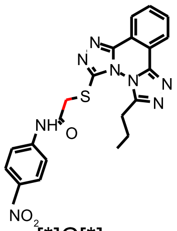 [*]C[*]	0.203
ECFP_6	781519895	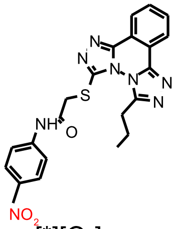 [*][O-]	0.0797
**Top Features for negative contribution**			
**Fingerprint**	**Bit/Smiles**	**Feature Structure**	**Score**
ECFP_6	1996767644	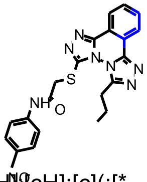 [*]:[cH]:[cH]:[c](:[*]):[*]	-0.251
ECFP_6	642810091	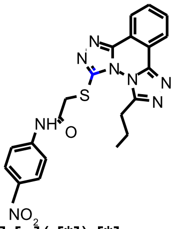 [*]:[c](:[*]):[*]	-0.247
ECFP_6	-182236392	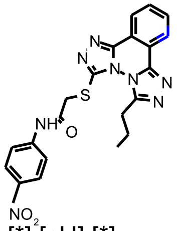 [*]:[cH]:[*]	-0.232

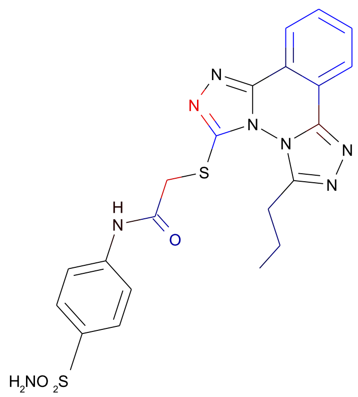

$C_{21}H_{20}N_8O_3S_2$
Molecular Weight: 496.56529
ALogP: 2.496
Rotatable Bonds: 7
Acceptors: 8
Donors: 2

Model Prediction

Prediction: 5.54
Unit: mg/kg_body_weight/day
Mahalanobis Distance: 12.4
Mahalanobis Distance p-value: 2.36e-006

Mahalanobis Distance: The Mahalanobis distance (MD) is a generalization of the Euclidean distance that accounts for correlations among the X properties. It is calculated as the distance to the center of the training data. The larger the MD, the less trustworthy the prediction.
Mahalanobis Distance p-value: The p-value gives the fraction of training data with an MD greater than or equal to the one for the given sample, assuming normally distributed data. The smaller the p-value, the less trustworthy the prediciton. For highly non-normal X properties (e.g., fingerprints), the MD p-value is wildly inaccurate.

Structural Similar Compounds

Name	420	Salicylazosulfapyridine	Azathioprine s
Structure	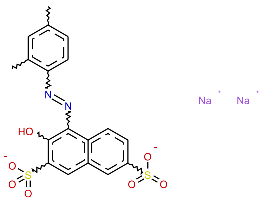	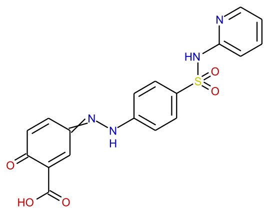	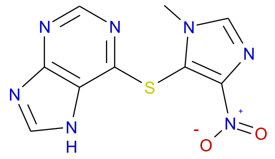
Actual Endpoint (-log C)	2.78302	2.5034	4.49253
Predicted Endpoint (-log C)	3.31546	3.54214	4.28607
Distance	0.936	1.012	1.033
Reference	CPDB	CPDB	CPDB

Model Applicability

Unknown features are fingerprint features in the query molecule, but not found or appearing too infrequently in the training set.

1. All properties and OPS components are within expected ranges.
2. Unknown ECFP_2 feature: 1986731747: [*]S[c]1:n:[*]:[*]:n:1:[*]
3. Unknown ECFP_2 feature: -435188506: [*]C[c]1:n:[*]:[*]:n:1:[*]
4. Unknown ECFP_2 feature: 78665610: [*][c]1:[*]:[*]:[c](:[*]):n:1:n(:[*]):[*]

Feature Contribution

Top features for positive contribution			
**Fingerprint**	**Bit/Smiles**	**Feature Structure**	**Score**
ECFP_6	655739385	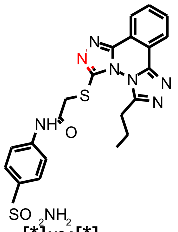 [*]:n:[*]	0.229

ECFP_6	1572579716	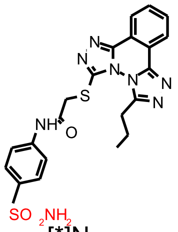 [*]N	0.225
ECFP_6	1559650422	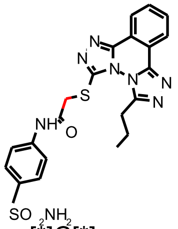 [*]C[*]	0.203

Top Features for negative contribution

Fingerprint	Bit/Smiles	Feature Structure	Score
ECFP_6	1996767644	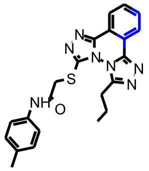 [*]:[cH]:[cH]:[c](:[*]):[*]	-0.251
ECFP_6	642810091	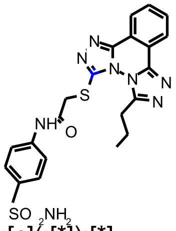 [*]:[c](:[*]):[*]	-0.247
ECFP_6	-182236392	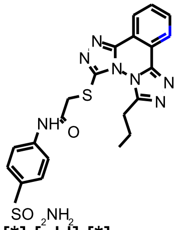 [*]:[cH]:[*]	-0.232

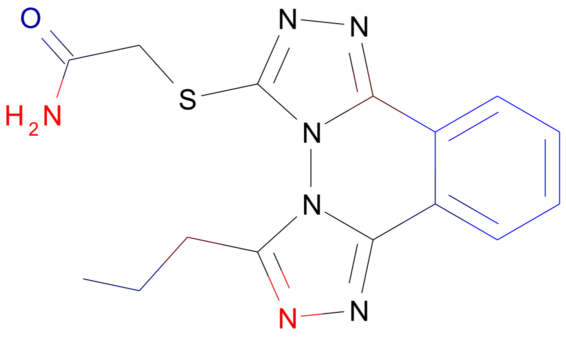

$C_{15}H_{15}N_7OS$
Molecular Weight: 341.39089
ALogP: 2.008
Rotatable Bonds: 5
Acceptors: 6
Donors: 1

Model Prediction

Prediction: 14.9
Unit: mg/kg_body_weight/day
Mahalanobis Distance: 10.7
Mahalanobis Distance p-value: 0.00395

Mahalanobis Distance: The Mahalanobis distance (MD) is a generalization of the Euclidean distance that accounts for correlations among the X properties. It is calculated as the distance to the center of the training data. The larger the MD, the less trustworthy the prediction.
Mahalanobis Distance p-value: The p-value gives the fraction of training data with an MD greater than or equal to the one for the given sample, assuming normally distributed data. The smaller the p-value, the less trustworthy the prediciton. For highly non-normal X properties (e.g., fingerprints), the MD p-value is wildly inaccurate.

Structural Similar Compounds

Name	Azathioprine s	646	470
Structure	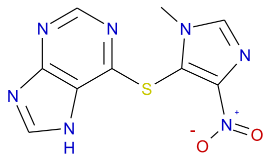	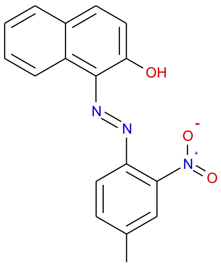	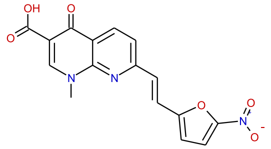
Actual Endpoint (-log C)	4.49253	0.937339	4.62839
Predicted Endpoint (-log C)	4.28607	3.26294	3.93264
Distance	0.724	0.810	0.814
Reference	CPDB	CPDB	CPDB

Model Applicability

Unknown features are fingerprint features in the query molecule, but not found or appearing too infrequently in the training set.

1. All properties and OPS components are within expected ranges.
2. Unknown ECFP_2 feature: 78665610: [*][c]1:[*]:[*]:[c](:[*]):n:1:n(:[*]):[*]
3. Unknown ECFP_2 feature: -435188506: [*]C[c]1:n:[*]:[*]:n:1:[*]
4. Unknown ECFP_2 feature: 1986731747: [*]S[c]1:n:[*]:[*]:n:1:[*]

Feature Contribution

Top features for positive contribution			
**Fingerprint**	**Bit/Smiles**	**Feature Structure**	**Score**
ECFP_6	655739385	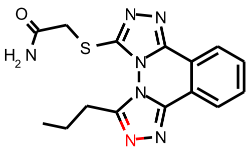 [*]:n:[*]	0.229

ECFP_6	1572579716	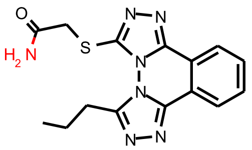 [*]N	0.225
ECFP_6	1559650422	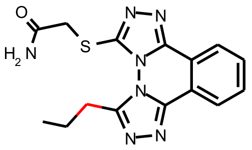 [*]C[*]	0.203
**Top Features for negative contribution**			
**Fingerprint**	**Bit/Smiles**	**Feature Structure**	**Score**
ECFP_6	1996767644	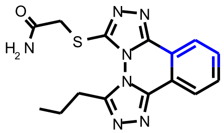 [*]:[cH]:[cH]:[c](:[*]):[*]	-0.251
ECFP_6	642810091	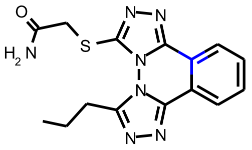 [*]:[c](:[*]):[*]	-0.247
ECFP_6	-182236392	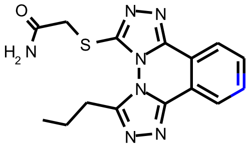 [*]:[cH]:[*]	-0.232

Doxorubicin

TOPKAT_Carcinogenic_Potency_TD50_Mouse

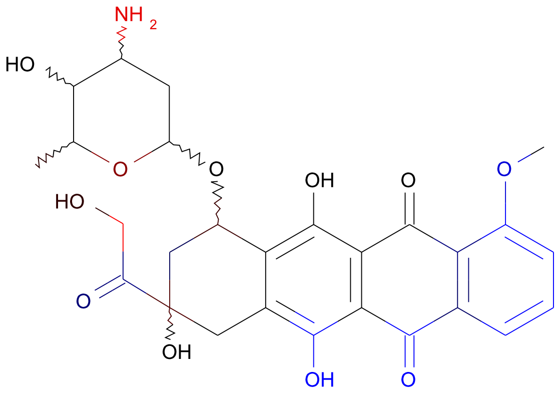

$C_{27}H_{29}NO_{11}$

Molecular Weight: 543.51925

ALogP: -4.4e-002

Rotatable Bonds: 5

Acceptors: 12

Donors: 6

Structural Similar Compounds

Name	532	338	380
Structure	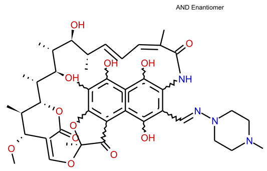	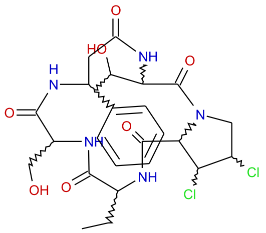	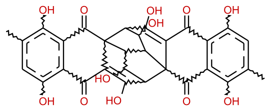
Actual Endpoint (-log C)	4.38903	4.39533	4.48977
Predicted Endpoint (-log C)	5.60554	4.31268	6.22716
Distance	0.909	0.937	0.952
Reference	CPDB	CPDB	CPDB

Model Prediction

Prediction: 11.2

Unit: mg/kg_body_weight/day

Mahalanobis Distance: 16.6

Mahalanobis Distance p-value: 3.86e-018

Mahalanobis Distance: The Mahalanobis distance (MD) is a generalization of the Euclidean distance that accounts for correlations among the X properties. It is calculated as the distance to the center of the training data. The larger the MD, the less trustworthy the prediction.
Mahalanobis Distance p-value: The p-value gives the fraction of training data with an MD greater than or equal to the one for the given sample, assuming normally distributed data. The smaller the p-value, the less trustworthy the prediciton. For highly non-normal X properties (e.g., fingerprints), the MD p-value is wildly inaccurate.

Model Applicability

Unknown features are fingerprint features in the query molecule, but not found or appearing too infrequently in the training set.

1. All properties and OPS components are within expected ranges.
2. Unknown ECFP_2 feature: 140080459: [*]CC(O)(C[*])C(=[*])[*]
3. Unknown ECFP_2 feature: 456242574: [*]C([*])OC([*])[*]
4. Unknown ECFP_2 feature: -1783281539: [*]CC(=O)C([*])([*])[*]
5. Unknown ECFP_2 feature: -1907393688: [*]C(=[*])CO
6. Unknown ECFP_2 feature: 1535429263: [*]OC(C)C([*])[*]
7. Unknown ECFP_2 feature: 1201786014: [*]CC(N)C([*])[*]
8. Unknown ECFP_2 feature: -932844120: [*]C([*])N
9. Unknown ECFP_2 feature: -2061744983: [*]CC(O[*])O[*]

Feature Contribution

Top features for positive contribution

Fingerprint	Bit/Smiles	Feature Structure	Score

ECFP_6	1572579716	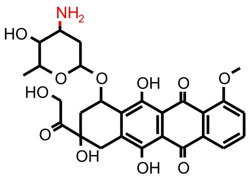 [*]N	0.225
ECFP_6	1559650422	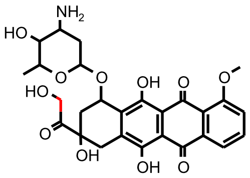 [*]C[*]	0.203
ECFP_6	683445015	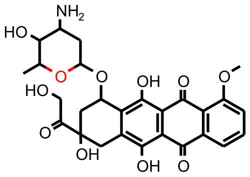 [*]O[*]	0.136

Top Features for negative contribution

**Fingerprint**	**Bit/Smiles**	**Feature Structure**	**Score**
ECFP_6	2106656448	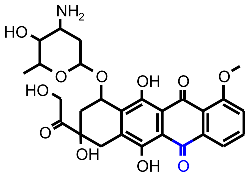 [*]C(=O)[*]	-0.275
ECFP_6	2019062761	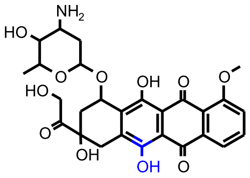 [*]:[c](:[*])O	-0.258

ECFP_6	1996767644	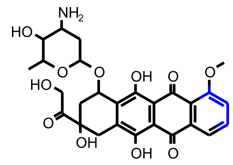 [*]:[cH]:[cH]:[c](:[*]):[*]	-0.251

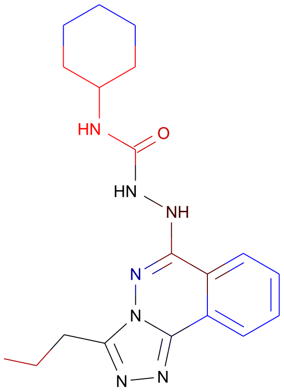

$C_{19}H_{25}N_7O$
Molecular Weight: 367.44809
ALogP: 3.681
Rotatable Bonds: 5
Acceptors: 5
Donors: 3

Model Prediction

Prediction: 0.651
Unit: mg/kg_body_weight/day
Mahalanobis Distance: 14.7
Mahalanobis Distance p-value: 2.78e-010

Mahalanobis Distance: The Mahalanobis distance (MD) is a generalization of the Euclidean distance that accounts for correlations among the X properties. It is calculated as the distance to the center of the training data. The larger the MD, the less trustworthy the prediction.
Mahalanobis Distance p-value: The p-value gives the fraction of training data with an MD greater than or equal to the one for the given sample, assuming normally distributed data. The smaller the p-value, the less trustworthy the prediciton. For highly non-normal X properties (e.g., fingerprints), the MD p-value is wildly inaccurate.

Structural Similar Compounds

Name	542	Ochratoxin A	Fluvastatin
Structure	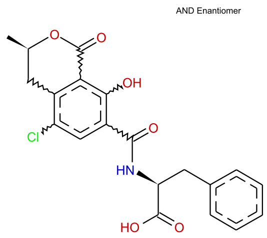	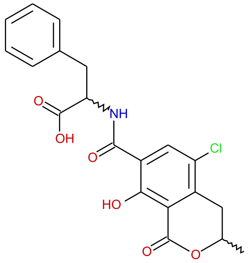	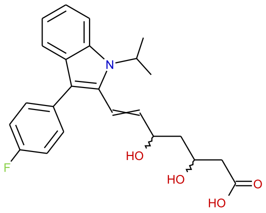
Actual Endpoint (-log C)	6.59334	6.47264	3.51742
Predicted Endpoint (-log C)	5.06501	5.06501	5.41573
Distance	0.659	0.659	0.675
Reference	CPDB	CPDB	CPDB

Model Applicability

Unknown features are fingerprint features in the query molecule, but not found or appearing too infrequently in the training set.

1. All properties and OPS components are within expected ranges.

Feature Contribution

Top features for positive contribution			
Fingerprint	Bit/Smiles	Feature Structure	Score
FCFP_6	-1043250487	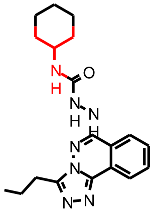 [*]CC(C[*])N[*]	1.15

FCFP_6	1	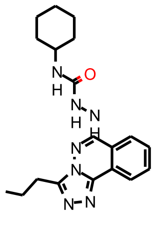 [*]=O	0.234
FCFP_6	-885550502	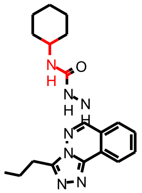 [*]C([*])NC(=[*])[*]	0.229
**Top Features for negative contribution**			
**Fingerprint**	**Bit/Smiles**	**Feature Structure**	**Score**
FCFP_6	1175638033	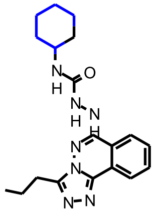 [*]C1[*]CCCC1	-0.512
FCFP_6	991735244	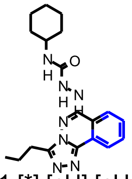 [*]:[c]1:[*]:[cH]:[cH]:[cH]:[cH]:1	-0.422
FCFP_6	16	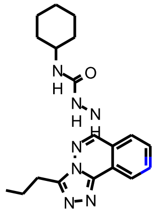 [*]:[cH]:[*]	-0.354

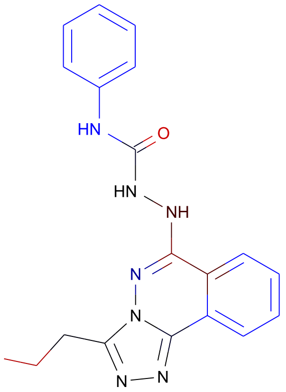

$C_{19}H_{19}N_7O$

Molecular Weight: 361.40046

ALogP: 3.399

Rotatable Bonds: 5

Acceptors: 5

Donors: 3

Model Prediction

Prediction: 19.9

Unit: mg/kg_body_weight/day

Mahalanobis Distance: 14.7

Mahalanobis Distance p-value: 2.53e-010

Mahalanobis Distance: The Mahalanobis distance (MD) is a generalization of the Euclidean distance that accounts for correlations among the X properties. It is calculated as the distance to the center of the training data. The larger the MD, the less trustworthy the prediction.
Mahalanobis Distance p-value: The p-value gives the fraction of training data with an MD greater than or equal to the one for the given sample, assuming normally distributed data. The smaller the p-value, the less trustworthy the prediciton. For highly non-normal X properties (e.g., fingerprints), the MD p-value is wildly inaccurate.

Structural Similar Compounds

Name	Fluvastatin	913	4-(2-Hydroxyethylamino)-2-(5-nitro-2-thienyl)quinazoline
Structure	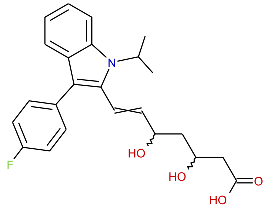	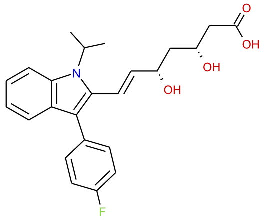	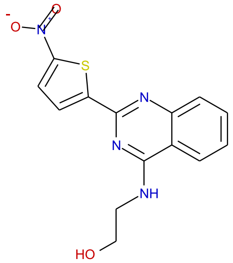
Actual Endpoint (-log C)	3.51742	3.51742	5.22831
Predicted Endpoint (-log C)	5.41573	5.41573	4.31976
Distance	0.716	0.716	0.726
Reference	CPDB	CPDB	CPDB

Model Applicability

Unknown features are fingerprint features in the query molecule, but not found or appearing too infrequently in the training set.

1. All properties and OPS components are within expected ranges.

Feature Contribution

Top features for positive contribution

Fingerprint	Bit/Smiles	Feature Structure	Score
FCFP_6	1	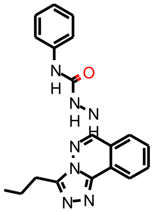 [*]=O	0.234

FCFP_6	307419094	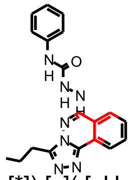 [*][c](:[*]):[c](:[cH]:[*]):[c](:[*]):[*]	0.121
FCFP_6	-1272798659	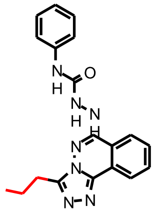 [*]CCC	0.11

Top Features for negative contribution

Fingerprint	Bit/Smiles	Feature Structure	Score
FCFP_6	991735244	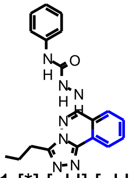 [*]:[c]1:[*]:[cH]:[cH]:[cH]:[cH]:1	-0.422
FCFP_6	-2093839777	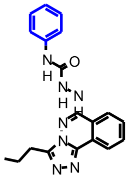 [*][c]1:[cH]:[cH]:[cH]:[cH]:[cH]:1	-0.378
FCFP_6	16	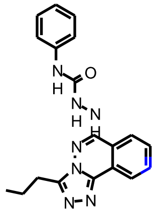 [*]:[cH]:[*]	-0.354

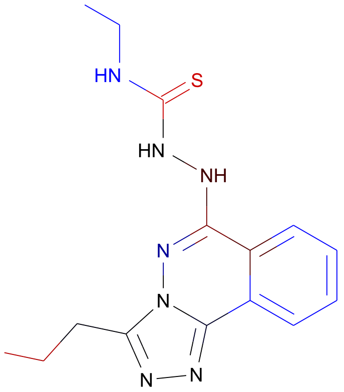

$C_{15}H_{19}N_7S$

Molecular Weight: 329.42325

ALogP: 3.388

Rotatable Bonds: 7

Acceptors: 5

Donors: 3

Model Prediction

Prediction: 34.6

Unit: mg/kg_body_weight/day

Mahalanobis Distance: 12.8

Mahalanobis Distance p-value: 1.55e-005

Mahalanobis Distance: The Mahalanobis distance (MD) is a generalization of the Euclidean distance that accounts for correlations among the X properties. It is calculated as the distance to the center of the training data. The larger the MD, the less trustworthy the prediction.
Mahalanobis Distance p-value: The p-value gives the fraction of training data with an MD greater than or equal to the one for the given sample, assuming normally distributed data. The smaller the p-value, the less trustworthy the prediciton. For highly non-normal X properties (e.g., fingerprints), the MD p-value is wildly inaccurate.

Structural Similar Compounds

Name	4-(2-Hydroxyethylamino)-2-(5-nitro-2-thienyl)quinazoline	4-Chloro-6-(2,3-xylidino)-2-pyri-mi-dinylthio(N-b-hydroxy-ethyl) acetamide	Fluvastatin
Structure	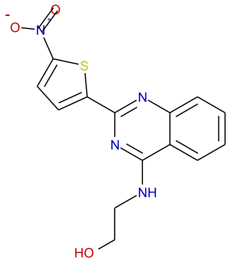	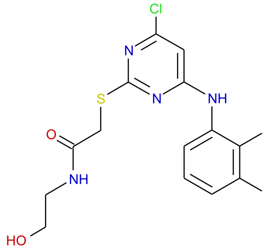	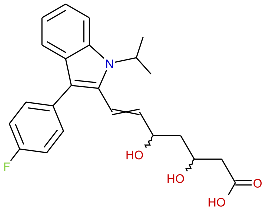
Actual Endpoint (-log C)	5.22831	4.75226	3.51742
Predicted Endpoint (-log C)	4.31976	3.29421	5.41573
Distance	0.623	0.629	0.638
Reference	CPDB	CPDB	CPDB

Model Applicability

Unknown features are fingerprint features in the query molecule, but not found or appearing too infrequently in the training set.

1. All properties and OPS components are within expected ranges.

Feature Contribution

Top features for positive contribution

Fingerprint	Bit/Smiles	Feature Structure	Score
FCFP_6	1	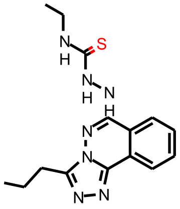 [*]=O	0.234

FCFP_6	-885550502	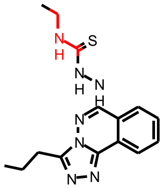 [*]C([*])NC(=[*])[*]	0.229
FCFP_6	307419094	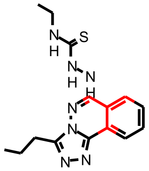 [*][c](:[*]):[c](:[cH]:[*]):[c](:[*]):[*]	0.121

Top Features for negative contribution

Fingerprint	Bit/Smiles	Feature Structure	Score
FCFP_6	-1272709286	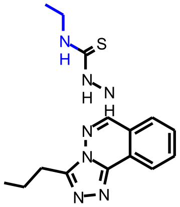 [*]NCC	-0.526
FCFP_6	991735244	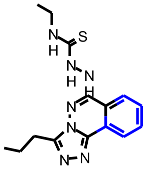 [*]:[c]1:[*]:[cH]:[cH]:[cH]:[cH]:1	-0.422
FCFP_6	16	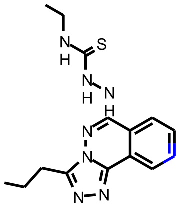 [*]:[cH]:[*]	-0.354

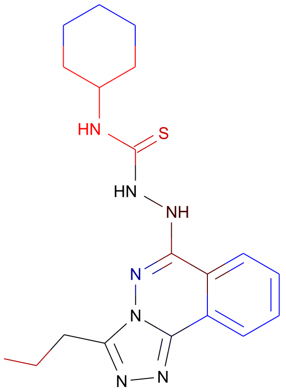

$C_{19}H_{25}N_7S$
Molecular Weight: 383.51369
ALogP: 4.898
Rotatable Bonds: 7
Acceptors: 5
Donors: 3

Model Prediction

Prediction: 0.873
Unit: mg/kg_body_weight/day
Mahalanobis Distance: 14.4
Mahalanobis Distance p-value: 1.34e-009

Mahalanobis Distance: The Mahalanobis distance (MD) is a generalization of the Euclidean distance that accounts for correlations among the X properties. It is calculated as the distance to the center of the training data. The larger the MD, the less trustworthy the prediction.
Mahalanobis Distance p-value: The p-value gives the fraction of training data with an MD greater than or equal to the one for the given sample, assuming normally distributed data. The smaller the p-value, the less trustworthy the prediciton. For highly non-normal X properties (e.g., fingerprints), the MD p-value is wildly inaccurate.

Structural Similar Compounds

Name	913	Fluvastatin	542
Structure	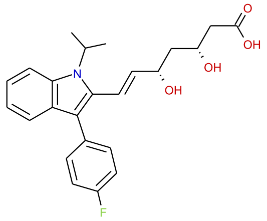	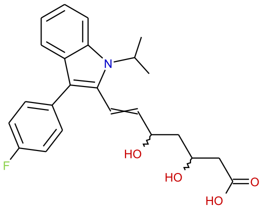	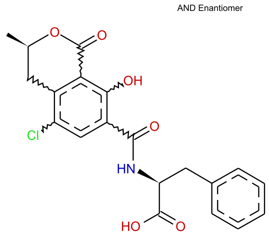
Actual Endpoint (-log C)	3.51742	3.51742	6.59334
Predicted Endpoint (-log C)	5.41573	5.41573	5.06501
Distance	0.618	0.618	0.671
Reference	CPDB	CPDB	CPDB

Model Applicability

Unknown features are fingerprint features in the query molecule, but not found or appearing too infrequently in the training set.

1. All properties and OPS components are within expected ranges.

Feature Contribution

Top features for positive contribution			
**Fingerprint**	**Bit/Smiles**	**Feature Structure**	**Score**
FCFP_6	-1043250487	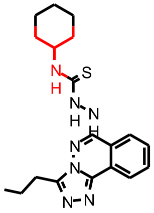 [*]CC(C[*])N[*]	1.15

FCFP_6	1	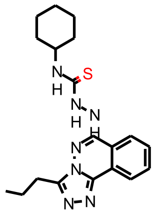 [*]=O	0.234
FCFP_6	-885550502	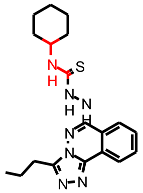 [*]C([*])NC(=[*])[*]	0.229
**Top Features for negative contribution**			
**Fingerprint**	**Bit/Smiles**	**Feature Structure**	**Score**
FCFP_6	1175638033	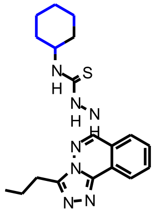 [*]C1[*]CCCC1	-0.512
FCFP_6	991735244	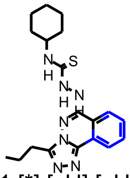 [*]:[c]1:[*]:[cH]:[cH]:[cH]:[cH]:1	-0.422
FCFP_6	16	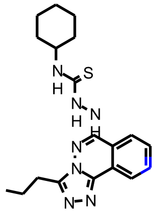 [*]:[cH]:[*]	-0.354

9a **TOPKAT_Carcinogenic_Potency_TD50_Rat**

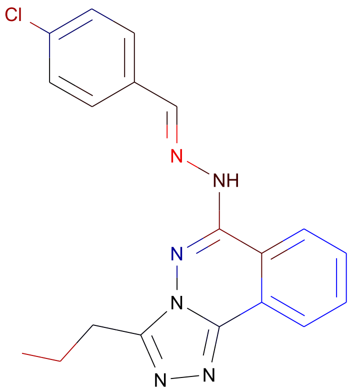

$C_{19}H_{17}ClN_6$
Molecular Weight: 364.83147
ALogP: 4.967
Rotatable Bonds: 5
Acceptors: 5
Donors: 1

Model Prediction

Prediction: 2.51
Unit: mg/kg_body_weight/day
Mahalanobis Distance: 13.1
Mahalanobis Distance p-value: 3.12e-006

Mahalanobis Distance: The Mahalanobis distance (MD) is a generalization of the Euclidean distance that accounts for correlations among the X properties. It is calculated as the distance to the center of the training data. The larger the MD, the less trustworthy the prediction.
Mahalanobis Distance p-value: The p-value gives the fraction of training data with an MD greater than or equal to the one for the given sample, assuming normally distributed data. The smaller the p-value, the less trustworthy the prediciton. For highly non-normal X properties (e.g., fingerprints), the MD p-value is wildly inaccurate.

Structural Similar Compounds

Name	Indomethacin	646	Omeprazole
Structure	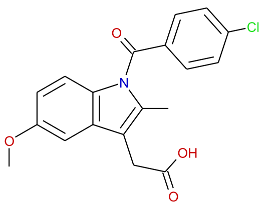	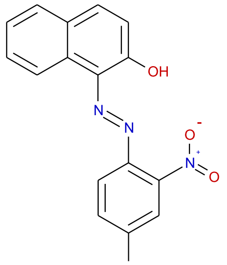	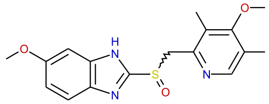
Actual Endpoint (-log C)	5.49293	2.41938	3.4628
Predicted Endpoint (-log C)	4.9569	3.77987	4.7324
Distance	0.621	0.680	0.711
Reference	CPDB	CPDB	CPDB

Model Applicability

Unknown features are fingerprint features in the query molecule, but not found or appearing too infrequently in the training set.

1. All properties and OPS components are within expected ranges.

Feature Contribution

Top features for positive contribution

Fingerprint	Bit/Smiles	Feature Structure	Score
FCFP_6	1	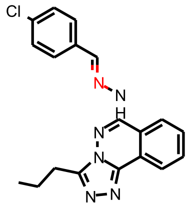 [*]=O	0.234

FCFP_6	32	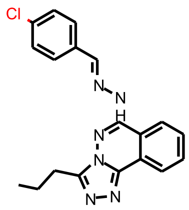 [*]Cl	0.154
FCFP_6	203677720	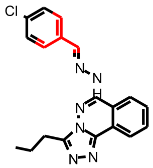 [*]=C[c](:[cH]:[*]):[cH]:[*]	0.137
**Top Features for negative contribution**			
**Fingerprint**	**Bit/Smiles**	**Feature Structure**	**Score**
FCFP_6	991735244	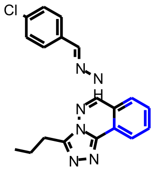 [*]:[c]1:[*]:[cH]:[cH]:[cH]:[cH]:1	-0.422
FCFP_6	16	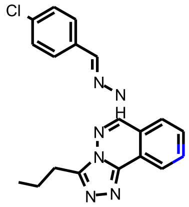 [*]:[cH]:[*]	-0.354
FCFP_6	17	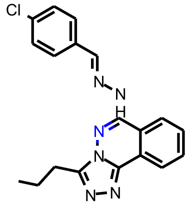 [*]:n:[*]	-0.149

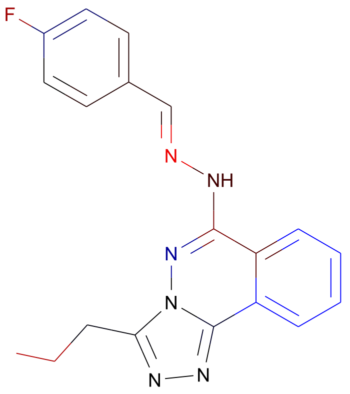

$C_{19}H_{17}FN_6$
Molecular Weight: 348.37688
ALogP: 4.508
Rotatable Bonds: 5
Acceptors: 5
Donors: 1

Model Prediction

Prediction: 2.67
Unit: mg/kg_body_weight/day
Mahalanobis Distance: 13.2
Mahalanobis Distance p-value: 2.07e-006

Mahalanobis Distance: The Mahalanobis distance (MD) is a generalization of the Euclidean distance that accounts for correlations among the X properties. It is calculated as the distance to the center of the training data. The larger the MD, the less trustworthy the prediction.
Mahalanobis Distance p-value: The p-value gives the fraction of training data with an MD greater than or equal to the one for the given sample, assuming normally distributed data. The smaller the p-value, the less trustworthy the prediciton. For highly non-normal X properties (e.g., fingerprints), the MD p-value is wildly inaccurate.

Structural Similar Compounds

Name	Indomethacin	646	Bemitradine
Structure	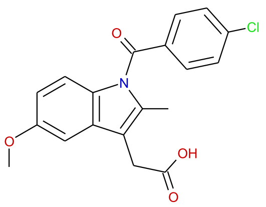	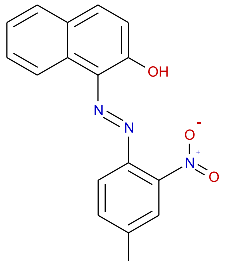	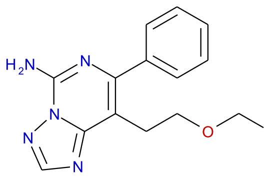
Actual Endpoint (-log C)	5.49293	2.41938	2.71351
Predicted Endpoint (-log C)	4.9569	3.77987	4.65043
Distance	0.616	0.681	0.688
Reference	CPDB	CPDB	CPDB

Model Applicability

Unknown features are fingerprint features in the query molecule, but not found or appearing too infrequently in the training set.

1. All properties and OPS components are within expected ranges.

Feature Contribution

Top features for positive contribution			
Fingerprint	Bit/Smiles	Feature Structure	Score
FCFP_6	1	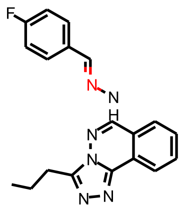 [*]=O	0.234

FCFP_6	32	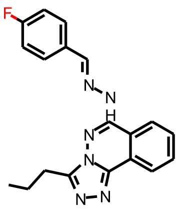 [*]Cl	0.154
FCFP_6	203677720	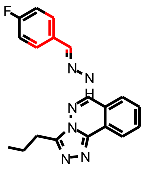 [*]=C[c](:[cH]:[*]):[cH]:[*]	0.137
**Top Features for negative contribution**			
**Fingerprint**	**Bit/Smiles**	**Feature Structure**	**Score**
FCFP_6	991735244	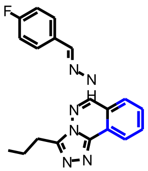 [*]:[c]1:[*]:[cH]:[cH]:[cH]:[cH]:1	-0.422
FCFP_6	16	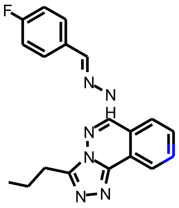 [*]:[cH]:[*]	-0.354
FCFP_6	17	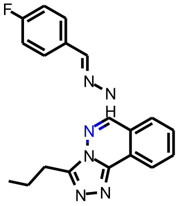 [*]:n:[*]	-0.149

9c

TOPKAT_Carcinogenic_Potency_TD50_Rat

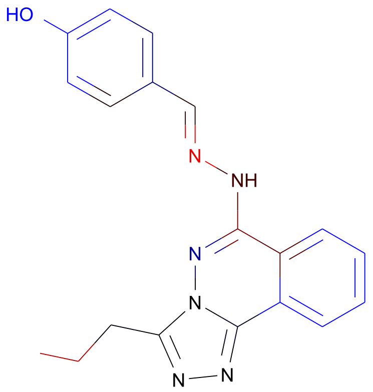

$C_{19}H_{18}N_6O$

Molecular Weight: 346.38582

ALogP: 4.061

Rotatable Bonds: 5

Acceptors: 6

Donors: 2

Model Prediction

Prediction: 20.5

Unit: mg/kg_body_weight/day

Mahalanobis Distance: 13.1

Mahalanobis Distance p-value: 2.81e-006

Mahalanobis Distance: The Mahalanobis distance (MD) is a generalization of the Euclidean distance that accounts for correlations among the X properties. It is calculated as the distance to the center of the training data. The larger the MD, the less trustworthy the prediction.
Mahalanobis Distance p-value: The p-value gives the fraction of training data with an MD greater than or equal to the one for the given sample, assuming normally distributed data. The smaller the p-value, the less trustworthy the prediciton. For highly non-normal X properties (e.g., fingerprints), the MD p-value is wildly inaccurate.

Structural Similar Compounds

Name	Phenolphthalein	646	Omeprazole
Structure	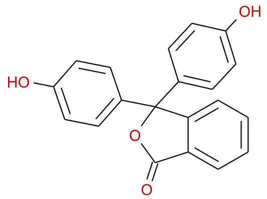	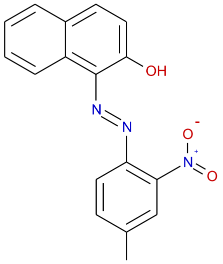	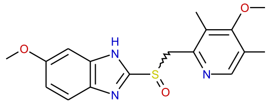
Actual Endpoint (-log C)	2.54766	2.41938	3.4628
Predicted Endpoint (-log C)	3.7508	3.77987	4.7324
Distance	0.671	0.680	0.698
Reference	CPDB	CPDB	CPDB

Model Applicability

Unknown features are fingerprint features in the query molecule, but not found or appearing too infrequently in the training set.

1. OPS PC7 out of range. Value: 6.5394. Training min, max, SD, explained variance: -5.0422, 6.1749, 1.868, 0.0335.

Feature Contribution

Top features for positive contribution			
**Fingerprint**	**Bit/Smiles**	**Feature Structure**	**Score**
FCFP_6	1	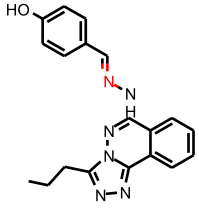 [*]=O	0.234

FCFP_6	203677720	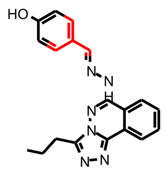 [*]=C[c](:[cH]:[*]):[cH]:[*]	0.137
FCFP_6	307419094	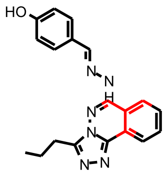 [*][c](:[*]):[c](:[cH]:[*]):[c](:[*]):[*]	0.121

Top Features for negative contribution

Fingerprint	Bit/Smiles	Feature Structure	Score
FCFP_6	991735244	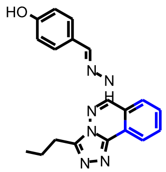 [*]:[c]1:[*]:[cH]:[cH]:[cH]:[cH]:1	-0.422
FCFP_6	7	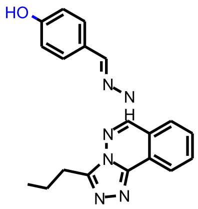 [*]O	-0.372
FCFP_6	16	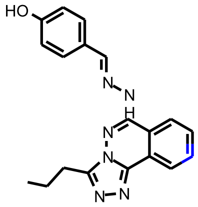 [*]:[cH]:[*]	-0.354

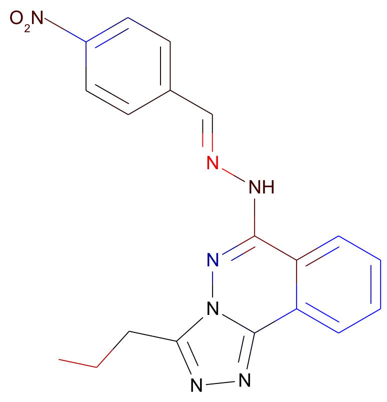

$C_{19}H_{17}N_7O_2$

Molecular Weight: 375.38397

ALogP: 4.197

Rotatable Bonds: 6

Acceptors: 7

Donors: 1

Model Prediction

Prediction: 1.99

Unit: mg/kg_body_weight/day

Mahalanobis Distance: 13.6

Mahalanobis Distance p-value: 1.67e-007

Mahalanobis Distance: The Mahalanobis distance (MD) is a generalization of the Euclidean distance that accounts for correlations among the X properties. It is calculated as the distance to the center of the training data. The larger the MD, the less trustworthy the prediction.

Mahalanobis Distance p-value: The p-value gives the fraction of training data with an MD greater than or equal to the one for the given sample, assuming normally distributed data. The smaller the p-value, the less trustworthy the prediciton. For highly non-normal X properties (e.g., fingerprints), the MD p-value is wildly inaccurate.

Structural Similar Compounds

Name	C.I. direct brown 95	4-Morpholino-2-(5-nitro-2-thi-enyl)quinazoline	4-Bis(2-hydroxyethyl)amino-2-(5-nitro-2-thienyl)quinazoline
Structure	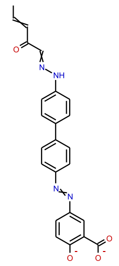	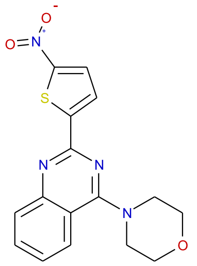	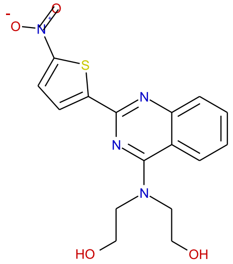
Actual Endpoint (-log C)	5.31387	4.83293	5.05984
Predicted Endpoint (-log C)	4.30266	4.97658	4.23808
Distance	0.677	0.691	0.707
Reference	CPDB	CPDB	CPDB

Model Applicability

Unknown features are fingerprint features in the query molecule, but not found or appearing too infrequently in the training set.

1. All properties and OPS components are within expected ranges.

Feature Contribution

Top features for positive contribution

Fingerprint	Bit/Smiles	Feature Structure	Score
FCFP_6	5	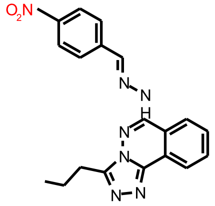 [*][O-]	0.431

FCFP_6	8	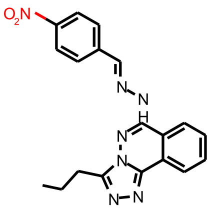<br>[*][N+](=[*])[*]	0.336
FCFP_6	1	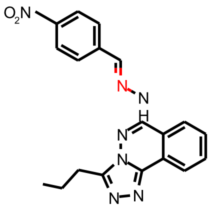<br>[*]=O	0.234
**Top Features for negative contribution**			
**Fingerprint**	**Bit/Smiles**	**Feature Structure**	**Score**
FCFP_6	991735244	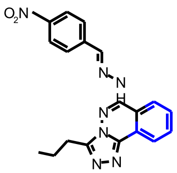<br>[*]:[c]1:[*]:[cH]:[cH<br>]:[cH]:[cH]:1	-0.422
FCFP_6	16	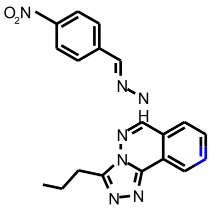<br>[*]:[cH]:[*]	-0.354
FCFP_6	1872392852	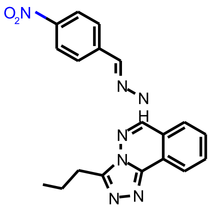<br>[*][N+](=O)[*]	-0.155

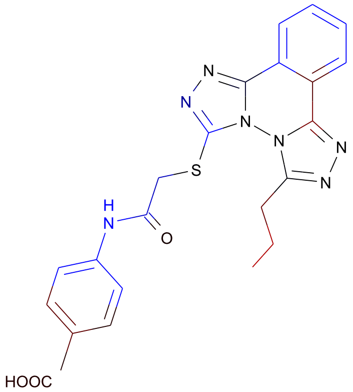

$C_{22}H_{19}N_7O_3S$

Molecular Weight: 461.49635

ALogP: 3.42

Rotatable Bonds: 7

Acceptors: 8

Donors: 2

Model Prediction

Prediction: 25.2

Unit: mg/kg_body_weight/day

Mahalanobis Distance: 17.2

Mahalanobis Distance p-value: 2.58e-018

Mahalanobis Distance: The Mahalanobis distance (MD) is a generalization of the Euclidean distance that accounts for correlations among the X properties. It is calculated as the distance to the center of the training data. The larger the MD, the less trustworthy the prediction.

Mahalanobis Distance p-value: The p-value gives the fraction of training data with an MD greater than or equal to the one for the given sample, assuming normally distributed data. The smaller the p-value, the less trustworthy the prediciton. For highly non-normal X properties (e.g., fingerprints), the MD p-value is wildly inaccurate.

Structural Similar Compounds

Name	C.I. direct brown 95	4-Bis(2-hydroxyethyl)amino-2-(5-nitro-2-thienyl)quinazoline	623
Structure	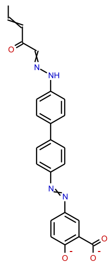	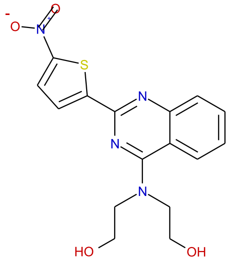	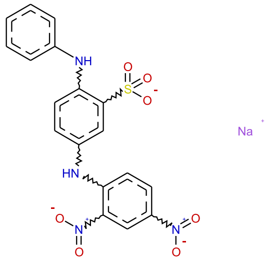
Actual Endpoint (-log C)	5.31387	5.05984	2.39985
Predicted Endpoint (-log C)	4.30266	4.23808	3.4177
Distance	0.827	0.833	0.840
Reference	CPDB	CPDB	CPDB

Model Applicability

Unknown features are fingerprint features in the query molecule, but not found or appearing too infrequently in the training set.

1. All properties and OPS components are within expected ranges.

Feature Contribution

Top features for positive contribution

Fingerprint	Bit/Smiles	Feature Structure	Score
FCFP_6	1	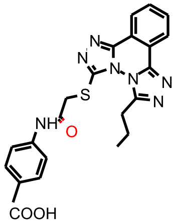 [*]=O	0.234

FCFP_6	203677720	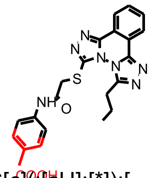 [*]=C[c](:[cH]:[*]):[cH]:[*]	0.137
FCFP_6	-1272768868	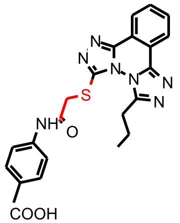 [*]SCC(=[*])[*]	0.127
**Top Features for negative contribution**			
**Fingerprint**	**Bit/Smiles**	**Feature Structure**	**Score**
FCFP_6	991735244	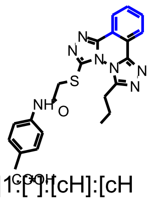 [*]:[c]1:[*]:[cH]:[cH]:[cH]:[cH]:1	-0.422
FCFP_6	7	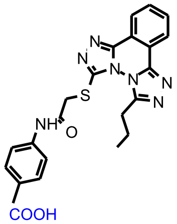 [*]O	-0.372
FCFP_6	16	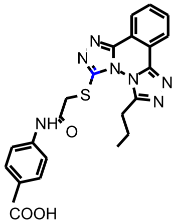 [*]:[cH]:[*]	-0.354

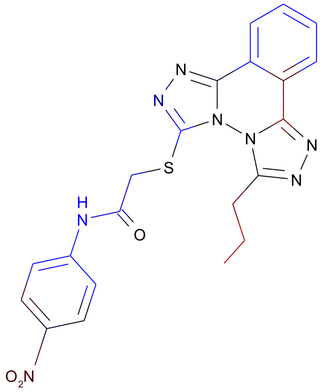

$C_{21}H_{18}N_8O_3S$

Molecular Weight: 462.48441

ALogP: 3.685

Rotatable Bonds: 7

Acceptors: 8

Donors: 1

Model Prediction

Prediction: 5.43

Unit: mg/kg_body_weight/day

Mahalanobis Distance: 17

Mahalanobis Distance p-value: 1.16e-017

Mahalanobis Distance: The Mahalanobis distance (MD) is a generalization of the Euclidean distance that accounts for correlations among the X properties. It is calculated as the distance to the center of the training data. The larger the MD, the less trustworthy the prediction.
Mahalanobis Distance p-value: The p-value gives the fraction of training data with an MD greater than or equal to the one for the given sample, assuming normally distributed data. The smaller the p-value, the less trustworthy the prediciton. For highly non-normal X properties (e.g., fingerprints), the MD p-value is wildly inaccurate.

Structural Similar Compounds

Name	623	C.I. direct brown 95	4-Bis(2-hydroxyethyl)amino-2-(5-nitro-2-thienyl)quinazoline
Structure	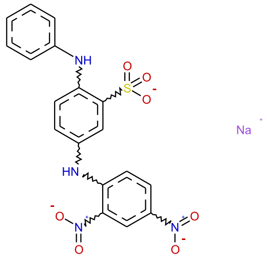	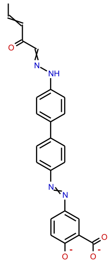	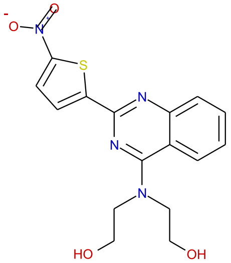
Actual Endpoint (-log C)	2.39985	5.31387	5.05984
Predicted Endpoint (-log C)	3.4177	4.30266	4.23808
Distance	0.803	0.829	0.843
Reference	CPDB	CPDB	CPDB

Model Applicability

Unknown features are fingerprint features in the query molecule, but not found or appearing too infrequently in the training set.

1. All properties and OPS components are within expected ranges.

Feature Contribution

Top features for positive contribution

Fingerprint	Bit/Smiles	Feature Structure	Score
FCFP_6	5	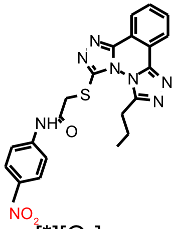 [*][O-]	0.431

FCFP_6	8	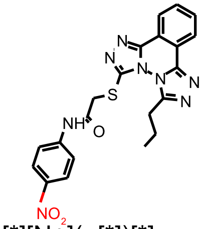 [*][N+](=[*])[*]	0.336
FCFP_6	1	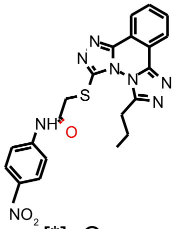 [*]=O	0.234
**Top Features for negative contribution**			
**Fingerprint**	**Bit/Smiles**	**Feature Structure**	**Score**
FCFP_6	991735244	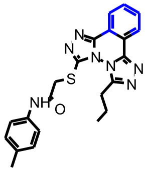 [*]:[c]1:[*]:[cH]:[cH]:[cH]:[cH]:1	-0.422
FCFP_6	16	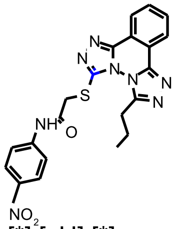 [*]:[cH]:[*]	-0.354
FCFP_6	590925877	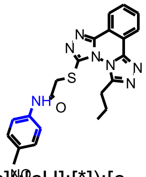 [*]N[c](:[cH]:[*]):[cH]:[*]	-0.323

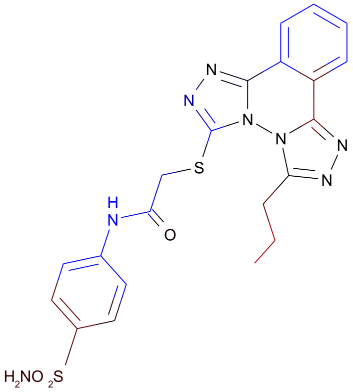

$C_{21}H_{20}N_8O_3S_2$
Molecular Weight: 496.56529
ALogP: 2.496
Rotatable Bonds: 7
Acceptors: 8
Donors: 2

Model Prediction

Prediction: 22
Unit: mg/kg_body_weight/day
Mahalanobis Distance: 15.7
Mahalanobis Distance p-value: 1.78e-013

Mahalanobis Distance: The Mahalanobis distance (MD) is a generalization of the Euclidean distance that accounts for correlations among the X properties. It is calculated as the distance to the center of the training data. The larger the MD, the less trustworthy the prediction.
Mahalanobis Distance p-value: The p-value gives the fraction of training data with an MD greater than or equal to the one for the given sample, assuming normally distributed data. The smaller the p-value, the less trustworthy the prediciton. For highly non-normal X properties (e.g., fingerprints), the MD p-value is wildly inaccurate.

Structural Similar Compounds

Name	623	4-Bis(2-hydroxyethyl)amino-2-(5-nitro-2-thienyl)quinazoline	418
Structure	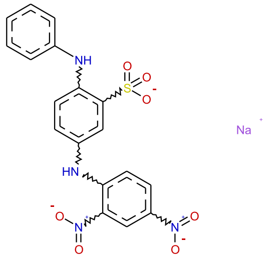	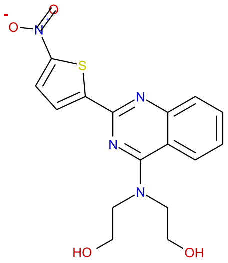	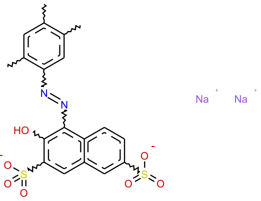
Actual Endpoint (-log C)	2.39985	5.05984	2.9349
Predicted Endpoint (-log C)	3.4177	4.23808	3.45907
Distance	0.800	0.870	0.870
Reference	CPDB	CPDB	CPDB

Model Applicability

Unknown features are fingerprint features in the query molecule, but not found or appearing too infrequently in the training set.

1. All properties and OPS components are within expected ranges.

Feature Contribution

Top features for positive contribution			
**Fingerprint**	**Bit/Smiles**	**Feature Structure**	**Score**
FCFP_6	1	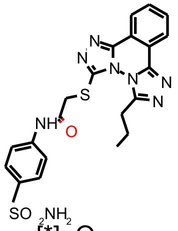 [*]=O	0.234

FCFP_6	203677720	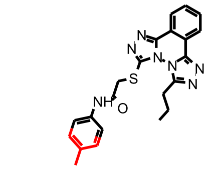 [*]=C[c](:[cH]:[*]):[cH]:[*]	0.137
FCFP_6	-1272768868	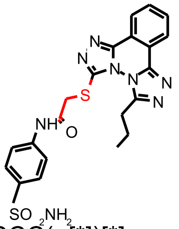 [*]SCC(=[*])[*]	0.127

Top Features for negative contribution

Fingerprint	Bit/Smiles	Feature Structure	Score
FCFP_6	991735244	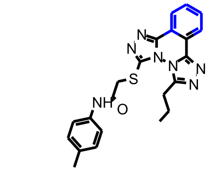 [*]:[c]1:[*]:[cH]:[cH]:[cH]:[cH]:1	-0.422
FCFP_6	16	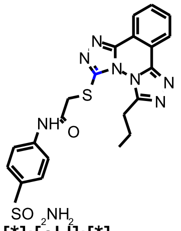 [*]:[cH]:[*]	-0.354
FCFP_6	590925877	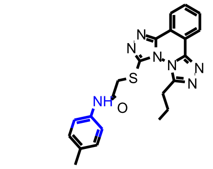 [*]N[c](:[cH]:[*]):[cH]:[*]	-0.323

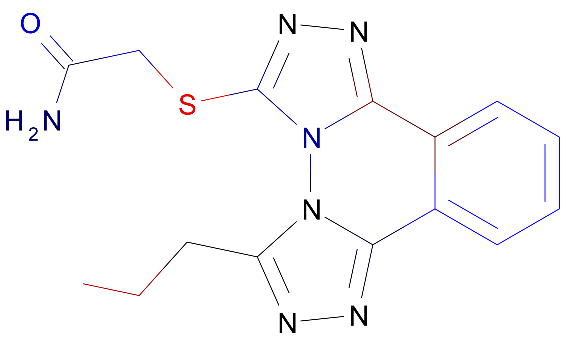

$C_{15}H_{15}N_7OS$
Molecular Weight: 341.39089
ALogP: 2.008
Rotatable Bonds: 5
Acceptors: 6
Donors: 1

Model Prediction

Prediction: 23.3
Unit: mg/kg_body_weight/day
Mahalanobis Distance: 14.5
Mahalanobis Distance p-value: 1.12e-009

Mahalanobis Distance: The Mahalanobis distance (MD) is a generalization of the Euclidean distance that accounts for correlations among the X properties. It is calculated as the distance to the center of the training data. The larger the MD, the less trustworthy the prediction.
Mahalanobis Distance p-value: The p-value gives the fraction of training data with an MD greater than or equal to the one for the given sample, assuming normally distributed data. The smaller the p-value, the less trustworthy the prediciton. For highly non-normal X properties (e.g., fingerprints), the MD p-value is wildly inaccurate.

Structural Similar Compounds

Name	4-(2-Hydroxyethylamino)-2-(5-nitro-2-thienyl)quinazoline	4-Morpholino-2-(5-nitro-2-thi-enyl)quinazoline	4-Bis(2-hydroxyethyl)amino-2-(5-nitro-2-thienyl)quinazoline
Structure	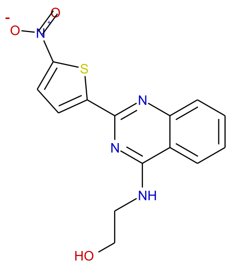	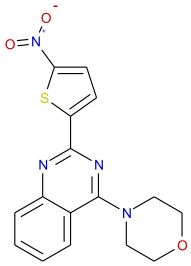	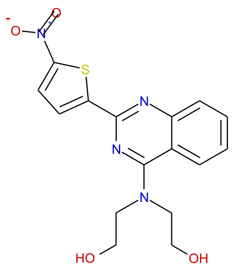
Actual Endpoint (-log C)	5.22831	4.83293	5.05984
Predicted Endpoint (-log C)	4.31976	4.97658	4.23808
Distance	0.662	0.675	0.678
Reference	CPDB	CPDB	CPDB

Model Applicability

Unknown features are fingerprint features in the query molecule, but not found or appearing too infrequently in the training set.

1. All properties and OPS components are within expected ranges.

Feature Contribution

Top features for positive contribution

Fingerprint	Bit/Smiles	Feature Structure	Score
FCFP_6	1	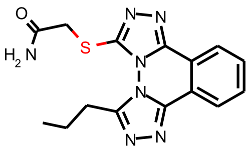 [*]=O	0.234

FCFP_6	-1272768868	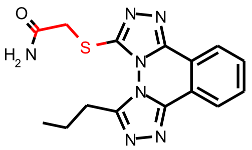 [*]SCC(=[*])[*]	0.127
FCFP_6	307419094	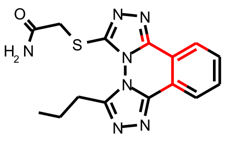 [*][c](:[*]):[c](:[cH]:[*]):[c](:[*]):[*]	0.121

Top Features for negative contribution

Fingerprint	Bit/Smiles	Feature Structure	Score
FCFP_6	991735244	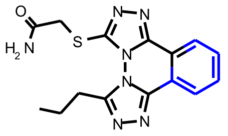 [*]:[c]1:[*]:[cH]:[cH]:[cH]:[cH]:1	-0.422
FCFP_6	16	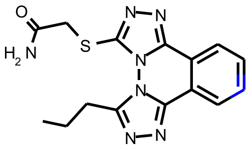 [*]:[cH]:[*]	-0.354
FCFP_6	566058135	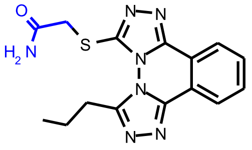 [*]CC(=O)N[*]	-0.182

Doxorubicin

TOPKAT_Carcinogenic_Potency_TD50_Rat

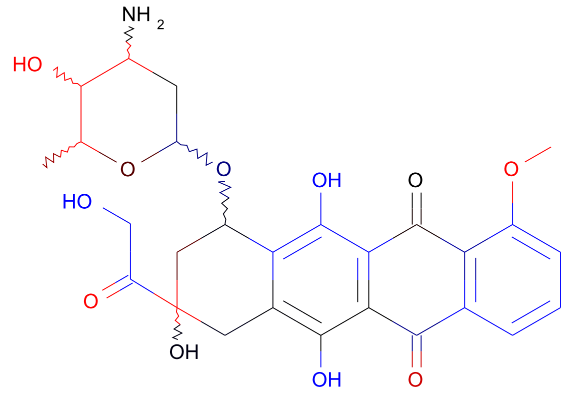

$C_{27}H_{29}NO_{11}$
Molecular Weight: 543.51925
ALogP: -4.4e-002
Rotatable Bonds: 5
Acceptors: 12
Donors: 6

Structural Similar Compounds

Name	424	D & C red no. 5	Salicylazosulfapyridine
Structure	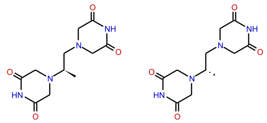	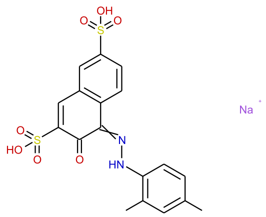	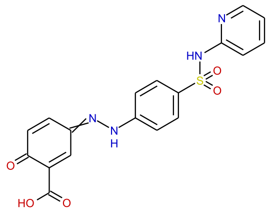
Actual Endpoint (-log C)	4.70022	3.04419	2.39891
Predicted Endpoint (-log C)	5.67571	4.52866	3.17598
Distance	1.039	1.056	1.057
Reference	CPDB	CPDB	CPDB

Model Prediction

Prediction: 0.861
Unit: mg/kg_body_weight/day
Mahalanobis Distance: 17.4
Mahalanobis Distance p-value: 6.65e-019

Mahalanobis Distance: The Mahalanobis distance (MD) is a generalization of the Euclidean distance that accounts for correlations among the X properties. It is calculated as the distance to the center of the training data. The larger the MD, the less trustworthy the prediction.
Mahalanobis Distance p-value: The p-value gives the fraction of training data with an MD greater than or equal to the one for the given sample, assuming normally distributed data. The smaller the p-value, the less trustworthy the prediciton. For highly non-normal X properties (e.g., fingerprints), the MD p-value is wildly inaccurate.

Model Applicability

Unknown features are fingerprint features in the query molecule, but not found or appearing too infrequently in the training set.

1. All properties and OPS components are within expected ranges.

Feature Contribution

Top features for positive contribution

Fingerprint	Bit/Smiles	Feature Structure	Score
FCFP_6	-1043250487	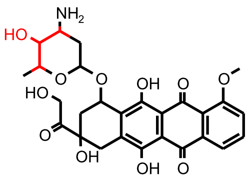 [*]CC(C[*])N[*]	1.15

FCFP_6	136627117	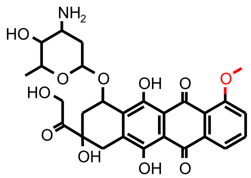 [*]OC	0.69
FCFP_6	565968762	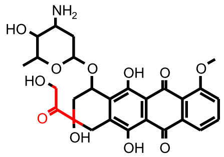 [*]CC(=O)C([*])([*])[*]	0.266
**Top Features for negative contribution**			
**Fingerprint**	**Bit/Smiles**	**Feature Structure**	**Score**
FCFP_6	-1272709286	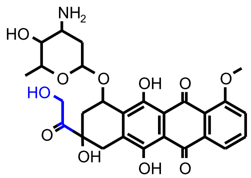 [*]NCC	-0.526
FCFP_6	991735244	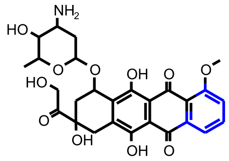 [*]:[c]1:[*]:[cH]:[cH]:[cH]:[cH]:1	-0.422
FCFP_6	7	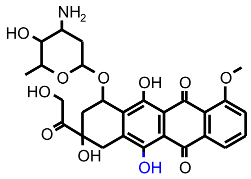 [*]O	-0.372

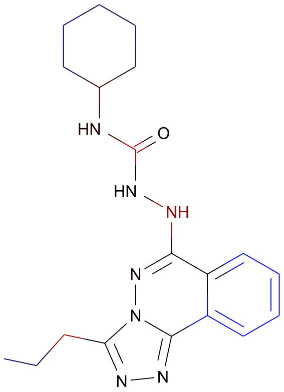

$C_{19}H_{25}N_7O$
Molecular Weight: 367.44809
ALogP: 3.681
Rotatable Bonds: 5
Acceptors: 5
Donors: 3

Model Prediction

Prediction: 0.118
Unit: g/kg_body_weight
Mahalanobis Distance: Not Available
Mahalanobis Distance p-value: Not Available

Mahalanobis Distance: The Mahalanobis distance (MD) is a generalization of the Euclidean distance that accounts for correlations among the X properties. It is calculated as the distance to the center of the training data. The larger the MD, the less trustworthy the prediction.
Mahalanobis Distance p-value: The p-value gives the fraction of training data with an MD greater than or equal to the one for the given sample, assuming normally distributed data. The smaller the p-value, the less trustworthy the prediciton. For highly non-normal X properties (e.g., fingerprints), the MD p-value is wildly inaccurate.

Model Applicability

Unknown features are fingerprint features in the query molecule, but not found or appearing too infrequently in the training set.

1. OPS PC18 out of range. Value: -5.5208. Training min, max, SD, explained variance: -4.7991, 6.1674, 1.831, 0.0147.
2. OPS PC31 out of range. Value: 6.0969. Training min, max, SD, explained variance: -4.0208, 4.2529, 1.246, 0.0068.
3. Unknown FCFP_2 feature: -332197802: [*][c]1:[*]:[*]:[c](:[*]):n:1:n:[*]
4. Unknown FCFP_2 feature: 1294344583: [*]NN[c](:[*]):[*]
5.
6.
7.
8. Unknown ECFP_6 feature: 672362763: [*]:n(:[*]):[*]
9. Unknown ECFP_6 feature: 1997021792: [*]:[cH]:[cH]:[cH]:[*]
10. Unknown ECFP_6 feature: 1333660716: [*][c](:[*]):[c](:[cH]:[*]):[c](:[*]):[*]
11. Unknown ECFP_6 feature: 1049768340: [*]N[c](:n:[*]):[c](:[*]):[*]
12. Unknown ECFP_6 feature: 835630791: [*][c](:[*]):n:n(:[*]):[*]
13. Unknown ECFP_6 feature: -1507082173: [*][c]1:[*]:[*]:[c](:[*]):n:1:n:[*]
14. Unknown ECFP_6 feature: 1049075205: [*]:n1:[*]:[*]:n:[c]:1:[c](:[*]):[*]
15. Unknown ECFP_6 feature: -1238415266: [*]NN[c](:[*]):[*]
16. Unknown ECFP_6 feature: -435188506: [*]C[c]1:n:[*]:[*]:n:1:[*]
17. Unknown ECFP_6 feature: 911256832: [*][c]1:[*]:[*]:n:n:1
18. Unknown ECFP_6 feature: -1795525632: [*]CC[c](:[*]):[*]
19. Unknown ECFP_6 feature: -1793471910: [*]CCC
20. Unknown ECFP_6 feature: 1635339976: [*]NNC(=[*])[*]
21. Unknown ECFP_6 feature: -649580166: [*]NC(=O)N[*]
22. Unknown ECFP_6 feature: -2091181441: [*]C([*])NC(=[*])[*]
23. Unknown ECFP_6 feature: -859078569: [*]CC(C[*])N[*]
24. Unknown ECFP_6 feature: -1332781180: [*]CCC[*]

Feature Contribution

Top features for positive contribution			
Fingerprint	Bit/Smiles	Feature Structure	Score

ECFP_6	-167460056	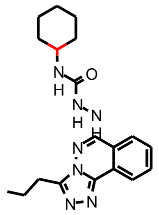<br>[*]C([*])[*]	0.136
ECFP_6	1559650422	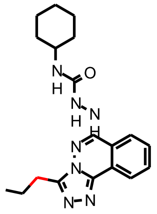<br>[*]C[*]	0.129
FCFP_6	3	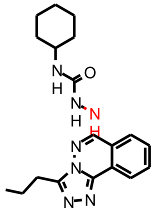<br>[*]N[*]	0.0924
**Top Features for negative contribution**			
**Fingerprint**	**Bit/Smiles**	**Feature Structure**	**Score**
FCFP_6	991735244	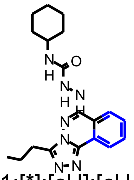<br>[*]:[c]1:[*]:[cH]:[cH<br>]:[cH]:[cH]:1	-0.134
ECFP_6	1564392544	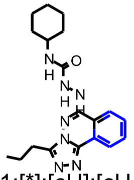<br>[*]:[c]1:[*]:[cH]:[cH<br>]:[cH]:[cH]:1	-0.133

FCFP_6	1	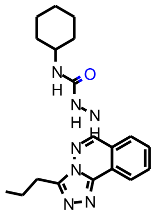 [*]=O	-0.102

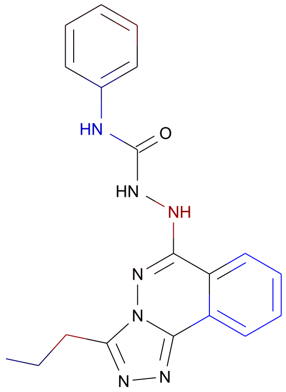

$C_{19}H_{19}N_7O$
Molecular Weight: 361.40046
ALogP: 3.399
Rotatable Bonds: 5
Acceptors: 5
Donors: 3

Model Prediction

Prediction: 0.142
Unit: g/kg_body_weight
Mahalanobis Distance: Not Available
Mahalanobis Distance p-value: Not Available

Mahalanobis Distance: The Mahalanobis distance (MD) is a generalization of the Euclidean distance that accounts for correlations among the X properties. It is calculated as the distance to the center of the training data. The larger the MD, the less trustworthy the prediction.
Mahalanobis Distance p-value: The p-value gives the fraction of training data with an MD greater than or equal to the one for the given sample, assuming normally distributed data. The smaller the p-value, the less trustworthy the prediciton. For highly non-normal X properties (e.g., fingerprints), the MD p-value is wildly inaccurate.

Model Applicability

Unknown features are fingerprint features in the query molecule, but not found or appearing too infrequently in the training set.

1. OPS PC31 out of range. Value: 5.2395. Training min, max, SD, explained variance: -4.0208, 4.2529, 1.246, 0.0068.
2. Unknown FCFP_2 feature: -332197802: [*][c]1:[*]:[*]:[c](:[*]):n:1:n:[*]
3. Unknown FCFP_2 feature: 1294344583: [*]NN[c](:[*]):[*]
4.
5.
6.
7.
8. Unknown ECFP_6 feature: 672362763: [*]:n(:[*]):[*]
9. Unknown ECFP_6 feature: 1997021792: [*]:[cH]:[cH]:[cH]:[*]
10. Unknown ECFP_6 feature: 1333660716: [*][c](:[*]):[c](:[cH]:[*]):[c](:[*]):[*]
11. Unknown ECFP_6 feature: 1049768340: [*]N[c](:n:[*]):[c](:[*]):[*]
12. Unknown ECFP_6 feature: 835630791: [*][c](:[*]):n:n(:[*]):[*]
13. Unknown ECFP_6 feature: -1507082173: [*][c]1:[*]:[*]:[c](:[*]):n:1:n:[*]
14. Unknown ECFP_6 feature: 1049075205: [*]:n1:[*]:[*]:n:[c]:1:[c](:[*]):[*]
15. Unknown ECFP_6 feature: -1238415266: [*]NN[c](:[*]):[*]
16. Unknown ECFP_6 feature: -435188506: [*]C[c]1:n:[*]:[*]:n:1:[*]
17. Unknown ECFP_6 feature: 911256832: [*][c]1:[*]:[*]:n:n:1
18. Unknown ECFP_6 feature: -1795525632: [*]CC[c](:[*]):[*]
19. Unknown ECFP_6 feature: -1793471910: [*]CCC
20. Unknown ECFP_6 feature: 1635339976: [*]NNC(=[*])[*]
21. Unknown ECFP_6 feature: -649580166: [*]NC(=O)N[*]
22. Unknown ECFP_6 feature: -177077903: [*]N[c](:[cH]:[*]):[cH]:[*]

Feature Contribution

Top features for positive contribution			
**Fingerprint**	**Bit/Smiles**	**Feature Structure**	**Score**
ECFP_6	1559650422	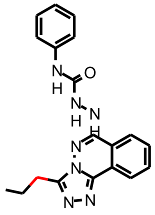 [*]C[*]	0.129

FCFP_6	3	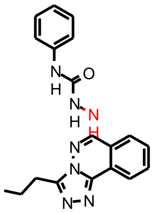<br>[*]N[*]	0.0924
FCFP_6	-2093839777	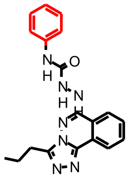<br>[*][c]1:[cH]:[cH]:[cH<br>]:[cH]:[cH]:1	0.078

Top Features for negative contribution

Fingerprint	Bit/Smiles	Feature Structure	Score
FCFP_6	991735244	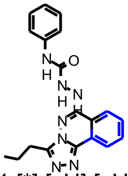<br>[*]:[c]1:[*]:[cH]:[cH<br>]:[cH]:[cH]:1	-0.134
ECFP_6	1564392544	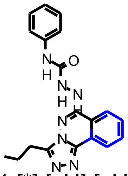<br>[*]:[c]1:[*]:[cH]:[cH<br>]:[cH]:[cH]:1	-0.133
FCFP_6	1	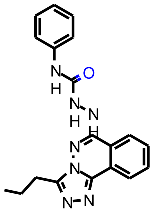<br>[*]=O	-0.102

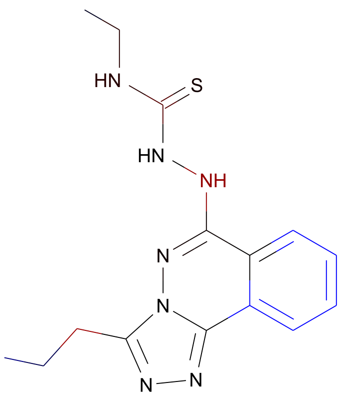

$C_{15}H_{19}N_7S$
Molecular Weight: 329.42325
ALogP: 3.388
Rotatable Bonds: 7
Acceptors: 5
Donors: 3

Model Prediction

Prediction: 0.0717
Unit: g/kg_body_weight
Mahalanobis Distance: Not Available
Mahalanobis Distance p-value: Not Available

Mahalanobis Distance: The Mahalanobis distance (MD) is a generalization of the Euclidean distance that accounts for correlations among the X properties. It is calculated as the distance to the center of the training data. The larger the MD, the less trustworthy the prediction.
Mahalanobis Distance p-value: The p-value gives the fraction of training data with an MD greater than or equal to the one for the given sample, assuming normally distributed data. The smaller the p-value, the less trustworthy the prediciton. For highly non-normal X properties (e.g., fingerprints), the MD p-value is wildly inaccurate.

Model Applicability

Unknown features are fingerprint features in the query molecule, but not found or appearing too infrequently in the training set.

1. OPS PC31 out of range. Value: 4.987. Training min, max, SD, explained variance: -4.0208, 4.2529, 1.246, 0.0068.
2. Unknown FCFP_2 feature: -332197802: [*][c]1:[*]:[*]:[c](:[*]):n:1:n:[*]
3. Unknown FCFP_2 feature: 1294344583: [*]NN[c](:[*]):[*]
4.
5.
6.
7.
8. Unknown ECFP_6 feature: 672362763: [*]:n(:[*]):[*]
9. Unknown ECFP_6 feature: 1997021792: [*]:[cH]:[cH]:[cH]:[*]
10. Unknown ECFP_6 feature: 1333660716: [*][c](:[*]):[c](:[cH]:[*]):[c](:[*]):[*]
11. Unknown ECFP_6 feature: 1049768340: [*]N[c](:n:[*]):[c](:[*]):[*]
12. Unknown ECFP_6 feature: 835630791: [*][c](:[*]):n:n(:[*]):[*]
13. Unknown ECFP_6 feature: -1507082173: [*][c]1:[*]:[*]:[c](:[*]):n:1:n:[*]
14. Unknown ECFP_6 feature: 1049075205: [*]:n1:[*]:[*]:n:[c]:1:[c](:[*]):[*]
15. Unknown ECFP_6 feature: -1238415266: [*]NN[c](:[*]):[*]
16. Unknown ECFP_6 feature: -435188506: [*]C[c]1:n:[*]:[*]:n:1:[*]
17. Unknown ECFP_6 feature: 911256832: [*][c]1:[*]:[*]:n:n:1
18. Unknown ECFP_6 feature: -1795525632: [*]CC[c](:[*]):[*]
19. Unknown ECFP_6 feature: -1793471910: [*]CCC
20. Unknown ECFP_6 feature: 1635339976: [*]NNC(=[*])[*]
21. Unknown ECFP_6 feature: 150794520: [*]NC(=S)N[*]
22. Unknown ECFP_6 feature: 1979182050: [*]C(=S)[*]
23. Unknown ECFP_6 feature: 497523368: [*]CNC(=[*])[*]
24. Unknown ECFP_6 feature: -950223878: [*]NCC

Feature Contribution

Top features for positive contribution			
**Fingerprint**	**Bit/Smiles**	**Feature Structure**	**Score**
ECFP_6	1559650422	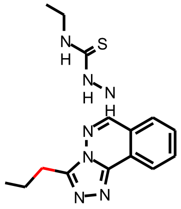 [*]C[*]	0.129

ECFP_6	-845108448	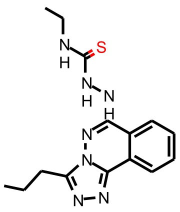<br>[*]=S	0.105
FCFP_6	3	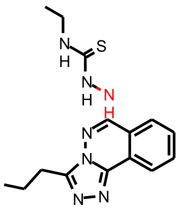<br>[*]N[*]	0.0924
**Top Features for negative contribution**			
**Fingerprint**	**Bit/Smiles**	**Feature Structure**	**Score**
FCFP_6	991735244	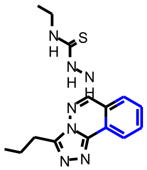<br>[*]:[c]1:[*]:[cH]:[cH<br>]:[cH]:[cH]:1	-0.134
ECFP_6	1564392544	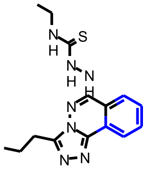<br>[*]:[c]1:[*]:[cH]:[cH<br>]:[cH]:[cH]:1	-0.133
FCFP_6	1	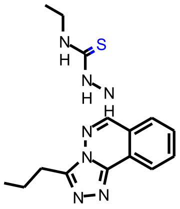<br>[*]=O	-0.102

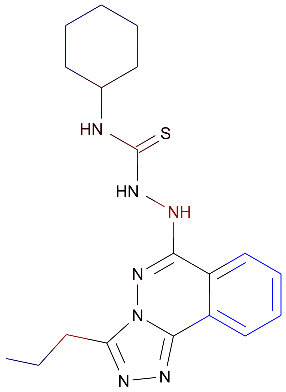

$C_{19}H_{25}N_7S$

Molecular Weight: 383.51369

ALogP: 4.898

Rotatable Bonds: 7

Acceptors: 5

Donors: 3

Model Prediction

Prediction: 0.0838

Unit: g/kg_body_weight

Mahalanobis Distance: Not Available

Mahalanobis Distance p-value: Not Available

Mahalanobis Distance: The Mahalanobis distance (MD) is a generalization of the Euclidean distance that accounts for correlations among the X properties. It is calculated as the distance to the center of the training data. The larger the MD, the less trustworthy the prediction.
Mahalanobis Distance p-value: The p-value gives the fraction of training data with an MD greater than or equal to the one for the given sample, assuming normally distributed data. The smaller the p-value, the less trustworthy the prediciton. For highly non-normal X properties (e.g., fingerprints), the MD p-value is wildly inaccurate.

Model Applicability

Unknown features are fingerprint features in the query molecule, but not found or appearing too infrequently in the training set.

1. OPS PC20 out of range. Value: 6.397. Training min, max, SD, explained variance: -5.771, 6.0322, 1.721, 0.0130.
2. OPS PC31 out of range. Value: 5.4043. Training min, max, SD, explained variance: -4.0208, 4.2529, 1.246, 0.0068.
3. Unknown FCFP_2 feature: -332197802: [*][c]1:[*]:[*]:[c](:[*]):n:1:n:[*]
4. Unknown FCFP_2 feature: 1294344583: [*]NN[c](:[*]):[*]
5.
6.
7.
8. Unknown ECFP_6 feature: 672362763: [*]:n(:[*]):[*]
9. Unknown ECFP_6 feature: 1997021792: [*]:[cH]:[cH]:[cH]:[*]
10. Unknown ECFP_6 feature: 1333660716: [*][c](:[*]):[c](:[cH]:[*]):[c](:[*]):[*]
11. Unknown ECFP_6 feature: 1049768340: [*]N[c](:n:[*]):[c](:[*]):[*]
12. Unknown ECFP_6 feature: 835630791: [*][c](:[*]):n:n(:[*]):[*]
13. Unknown ECFP_6 feature: -1507082173: [*][c]1:[*]:[*]:[c](:[*]):n:1:n:[*]
14. Unknown ECFP_6 feature: 1049075205: [*]:n1:[*]:[*]:n:[c]:1:[c](:[*]):[*]
15. Unknown ECFP_6 feature: -1238415266: [*]NN[c](:[*]):[*]
16. Unknown ECFP_6 feature: -435188506: [*]C[c]1:n:[*]:[*]:n:1:[*]
17. Unknown ECFP_6 feature: 911256832: [*][c]1:[*]:[*]:n:n:1
18. Unknown ECFP_6 feature: -1795525632: [*]CC[c](:[*]):[*]
19. Unknown ECFP_6 feature: -1793471910: [*]CCC
20. Unknown ECFP_6 feature: 1635339976: [*]NNC(=[*])[*]
21. Unknown ECFP_6 feature: 150794520: [*]NC(=S)N[*]
22. Unknown ECFP_6 feature: 1979182050: [*]C(=S)[*]
23. Unknown ECFP_6 feature: -2091181441: [*]C([*])NC(=[*])[*]
24. Unknown ECFP_6 feature: -859078569: [*]CC(C[*])N[*]
25. Unknown ECFP_6 feature: -1332781180: [*]CCC[*]

Feature Contribution

Top features for positive contribution			
**Fingerprint**	**Bit/Smiles**	**Feature Structure**	**Score**

ECFP_6	-167460056	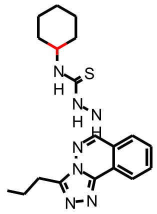 [*]C([*])[*]	0.136
ECFP_6	1559650422	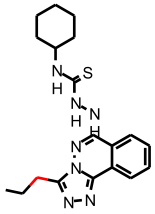 [*]C[*]	0.129
ECFP_6	-845108448	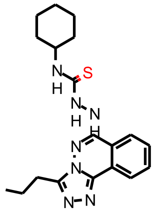 [*]=S	0.105

Top Features for negative contribution

Fingerprint	Bit/Smiles	Feature Structure	Score
FCFP_6	991735244	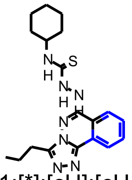 [*]:[c]1:[*]:[cH]:[cH]:[cH]:[cH]:1	-0.134
ECFP_6	1564392544	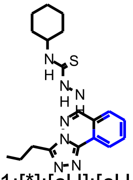 [*]:[c]1:[*]:[cH]:[cH]:[cH]:[cH]:1	-0.133

FCFP_6	1	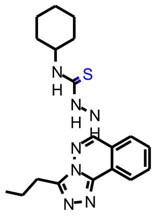 [*]=O	-0.102

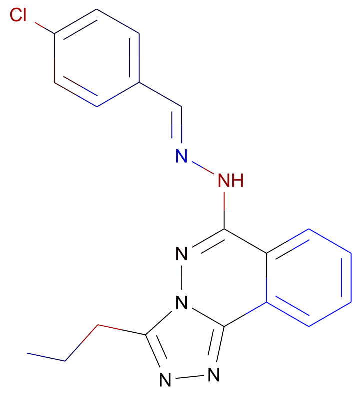

$C_{19}H_{17}ClN_6$
Molecular Weight: 364.83147
ALogP: 4.967
Rotatable Bonds: 5
Acceptors: 5
Donors: 1

Model Prediction

Prediction: 0.059
Unit: g/kg_body_weight
Mahalanobis Distance: Not Available
Mahalanobis Distance p-value: Not Available

Mahalanobis Distance: The Mahalanobis distance (MD) is a generalization of the Euclidean distance that accounts for correlations among the X properties. It is calculated as the distance to the center of the training data. The larger the MD, the less trustworthy the prediction.
Mahalanobis Distance p-value: The p-value gives the fraction of training data with an MD greater than or equal to the one for the given sample, assuming normally distributed data. The smaller the p-value, the less trustworthy the prediciton. For highly non-normal X properties (e.g., fingerprints), the MD p-value is wildly inaccurate.

Model Applicability

Unknown features are fingerprint features in the query molecule, but not found or appearing too infrequently in the training set.

1. All properties and OPS components are within expected ranges.
2. Unknown FCFP_2 feature: -332197802: [*][c]1:[*]:[*]:[c](:[*]):n:1:n:[*]
3. Unknown FCFP_2 feature: 1294285001: [*]=NN[c](:[*]):[*]
4.
5.
6.
7.
8. Unknown ECFP_6 feature: 672362763: [*]:n(:[*]):[*]
9. Unknown ECFP_6 feature: 1997021792: [*]:[cH]:[cH]:[cH]:[*]
10. Unknown ECFP_6 feature: 1333660716: [*][c](:[*]):[c](:[cH]:[*]):[c](:[*]):[*]
11. Unknown ECFP_6 feature: 1049768340: [*]N[c](:n:[*]):[c](:[*]):[*]
12. Unknown ECFP_6 feature: 835630791: [*][c](:[*]):n:n(:[*]):[*]
13. Unknown ECFP_6 feature: -1507082173: [*][c]1:[*]:[*]:[c](:[*]):n:1:n:[*]
14. Unknown ECFP_6 feature: 1049075205: [*]:n1:[*]:[*]:n:[c]:1:[c](:[*]):[*]
15. Unknown ECFP_6 feature: -435188506: [*]C[c]1:n:[*]:[*]:n:1:[*]
16. Unknown ECFP_6 feature: 911256832: [*][c]1:[*]:[*]:n:n:1
17. Unknown ECFP_6 feature: -1795525632: [*]CC[c](:[*]):[*]
18. Unknown ECFP_6 feature: -1793471910: [*]CCC
19. Unknown ECFP_6 feature: -1236714312: [*]=NN[c](:[*]):[*]
20. Unknown ECFP_6 feature: 1814278164: [*]N\N=C\[*]
21. Unknown ECFP_6 feature: -1832102709: [*]\N=C\[c](:[*]):[*]
22. Unknown ECFP_6 feature: -176483725: [*]=C[c](:[cH]:[*]):[cH]:[*]
23. Unknown ECFP_6 feature: -176494269: [*]:[cH]:[c](Cl):[cH]:[*]
24. Unknown ECFP_6 feature: 99947387: [*]:[c](:[*])Cl

Feature Contribution

Top features for positive contribution			
**Fingerprint**	**Bit/Smiles**	**Feature Structure**	**Score**
ECFP_6	1559650422	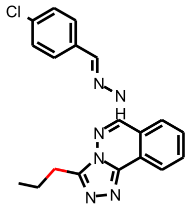 [*]C[*]	0.129

FCFP_6	32	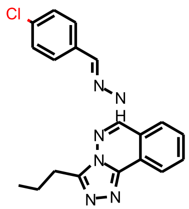 [*]Cl	0.101
FCFP_6	3	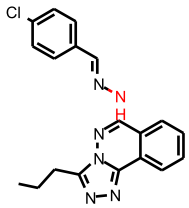 [*]N[*]	0.0924
**Top Features for negative contribution**			
**Fingerprint**	**Bit/Smiles**	**Feature Structure**	**Score**
FCFP_6	991735244	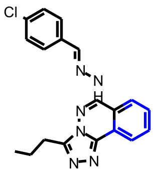 [*]:[c]1:[*]:[cH]:[cH]:[cH]:[cH]:1	-0.134
ECFP_6	1564392544	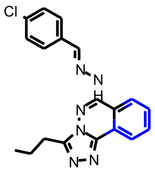 [*]:[c]1:[*]:[cH]:[cH]:[cH]:[cH]:1	-0.133
FCFP_6	1	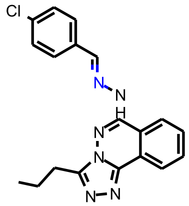 [*]=O	-0.102

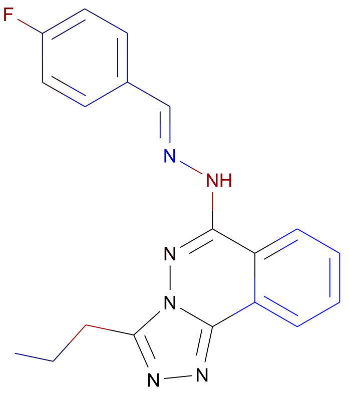

$C_{19}H_{17}FN_6$
Molecular Weight: 348.37688
ALogP: 4.508
Rotatable Bonds: 5
Acceptors: 5
Donors: 1

Model Prediction

Prediction: 0.0634
Unit: g/kg_body_weight
Mahalanobis Distance: Not Available
Mahalanobis Distance p-value: Not Available

Mahalanobis Distance: The Mahalanobis distance (MD) is a generalization of the Euclidean distance that accounts for correlations among the X properties. It is calculated as the distance to the center of the training data. The larger the MD, the less trustworthy the prediction.
Mahalanobis Distance p-value: The p-value gives the fraction of training data with an MD greater than or equal to the one for the given sample, assuming normally distributed data. The smaller the p-value, the less trustworthy the prediciton. For highly non-normal X properties (e.g., fingerprints), the MD p-value is wildly inaccurate.

Model Applicability

Unknown features are fingerprint features in the query molecule, but not found or appearing too infrequently in the training set.

1. All properties and OPS components are within expected ranges.
2. Unknown FCFP_2 feature: -332197802: [*][c]1:[*]:[*]:[c](:[*]):n:1:n:[*]
3. Unknown FCFP_2 feature: 1294285001: [*]=NN[c](:[*]):[*]
4.
5.
6.
7.
8. Unknown ECFP_6 feature: 672362763: [*]:n(:[*]):[*]
9. Unknown ECFP_6 feature: -1046436026: [*]F
10. Unknown ECFP_6 feature: 1997021792: [*]:[cH]:[cH]:[cH]:[*]
11. Unknown ECFP_6 feature: 1333660716: [*][c](:[*]):[c](:[cH]:[*]):[c](:[*]):[*]
12. Unknown ECFP_6 feature: 1049768340: [*]N[c](:n:[*]):[c](:[*]):[*]
13. Unknown ECFP_6 feature: 835630791: [*][c](:[*]):n:n(:[*]):[*]
14. Unknown ECFP_6 feature: -1507082173: [*][c]1:[*]:[*]:[c](:[*]):n:1:n:[*]
15. Unknown ECFP_6 feature: 1049075205: [*]:n1:[*]:[*]:n:[c]:1:[c](:[*]):[*]
16. Unknown ECFP_6 feature: -435188506: [*]C[c]1:n:[*]:[*]:n:1:[*]
17. Unknown ECFP_6 feature: 911256832: [*][c]1:[*]:[*]:n:n:1
18. Unknown ECFP_6 feature: -1795525632: [*]CC[c](:[*]):[*]
19. Unknown ECFP_6 feature: -1793471910: [*]CCC
20. Unknown ECFP_6 feature: -1236714312: [*]=NN[c](:[*]):[*]
21. Unknown ECFP_6 feature: 1814278164: [*]N\N=C\[*]
22. Unknown ECFP_6 feature: -1832102709: [*]\N=C\[c](:[*]):[*]
23. Unknown ECFP_6 feature: -176483725: [*]=C[c](:[cH]:[*]):[cH]:[*]
24. Unknown ECFP_6 feature: -176686665: [*]:[cH]:[c](F):[cH]:[*]
25. Unknown ECFP_6 feature: 220735655: [*]:[c](:[*])F

Feature Contribution

Top features for positive contribution			
**Fingerprint**	**Bit/Smiles**	**Feature Structure**	**Score**
ECFP_6	1559650422	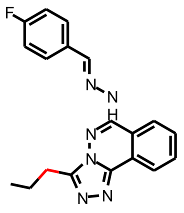 [*]C[*]	0.129

FCFP_6	32	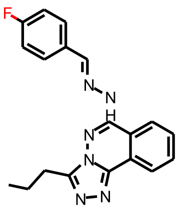<br>[*]Cl	0.101
FCFP_6	3	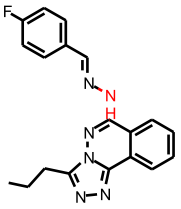<br>[*]N[*]	0.0924
**Top Features for negative contribution**			
**Fingerprint**	**Bit/Smiles**	**Feature Structure**	**Score**
FCFP_6	991735244	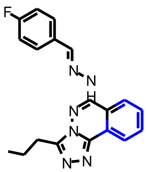<br>[*]:[c]1:[*]:[cH]:[cH<br>]:[cH]:[cH]:1	-0.134
ECFP_6	1564392544	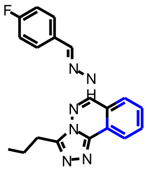<br>[*]:[c]1:[*]:[cH]:[cH<br>]:[cH]:[cH]:1	-0.133
FCFP_6	1	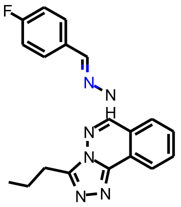<br>[*]=O	-0.102

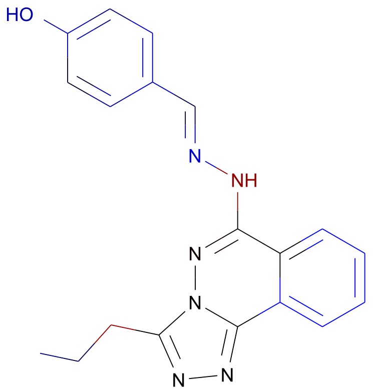

$C_{19}H_{18}N_6O$
Molecular Weight: 346.38582
ALogP: 4.061
Rotatable Bonds: 5
Acceptors: 6
Donors: 2

Model Prediction

Prediction: 0.148
Unit: g/kg_body_weight
Mahalanobis Distance: Not Available
Mahalanobis Distance p-value: Not Available

Mahalanobis Distance: The Mahalanobis distance (MD) is a generalization of the Euclidean distance that accounts for correlations among the X properties. It is calculated as the distance to the center of the training data. The larger the MD, the less trustworthy the prediction.
Mahalanobis Distance p-value: The p-value gives the fraction of training data with an MD greater than or equal to the one for the given sample, assuming normally distributed data. The smaller the p-value, the less trustworthy the prediciton. For highly non-normal X properties (e.g., fingerprints), the MD p-value is wildly inaccurate.

Model Applicability

Unknown features are fingerprint features in the query molecule, but not found or appearing too infrequently in the training set.

1. OPS PC31 out of range. Value: 4.9251. Training min, max, SD, explained variance: -4.0208, 4.2529, 1.246, 0.0068.
2. Unknown FCFP_2 feature: -332197802: [*][c]1:[*]:[*]:[c](:[*]):n:1:n:[*]
3. Unknown FCFP_2 feature: 1294285001: [*]=NN[c](:[*]):[*]
4.
5.
6.
7.
8. Unknown ECFP_6 feature: 672362763: [*]:n(:[*]):[*]
9. Unknown ECFP_6 feature: 1997021792: [*]:[cH]:[cH]:[cH]:[*]
10. Unknown ECFP_6 feature: 1333660716: [*][c](:[*]):[c](:[cH]:[*]):[c](:[*]):[*]
11. Unknown ECFP_6 feature: 1049768340: [*]N[c](:n:[*]):[c](:[*]):[*]
12. Unknown ECFP_6 feature: 835630791: [*][c](:[*]):n:n(:[*]):[*]
13. Unknown ECFP_6 feature: -1507082173: [*][c]1:[*]:[*]:[c](:[*]):n:1:n:[*]
14. Unknown ECFP_6 feature: 1049075205: [*]:n1:[*]:[*]:n:[c]:1:[c](:[*]):[*]
15. Unknown ECFP_6 feature: -435188506: [*]C[c]1:n:[*]:[*]:n:1:[*]
16. Unknown ECFP_6 feature: 911256832: [*][c]1:[*]:[*]:n:n:1
17. Unknown ECFP_6 feature: -1795525632: [*]CC[c](:[*]):[*]
18. Unknown ECFP_6 feature: -1793471910: [*]CCC
19. Unknown ECFP_6 feature: -1236714312: [*]=NN[c](:[*]):[*]
20. Unknown ECFP_6 feature: 1814278164: [*]N\N=C\[*]
21. Unknown ECFP_6 feature: -1832102709: [*]\N=C\[c](:[*]):[*]
22. Unknown ECFP_6 feature: -176483725: [*]=C[c](:[cH]:[*]):[cH]:[*]
23. Unknown ECFP_6 feature: -177786161: [*]:[cH]:[c](O):[cH]:[*]
24. Unknown ECFP_6 feature: 2019062761: [*]:[c](:[*])O

Feature Contribution

Top features for positive contribution			
**Fingerprint**	**Bit/Smiles**	**Feature Structure**	**Score**
ECFP_6	1559650422	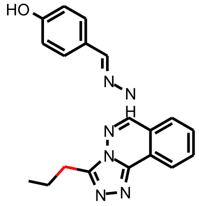 [*]C[*]	0.129

FCFP_6	3	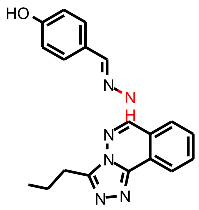 [*]N[*]	0.0924
ECFP_6	-1925046727	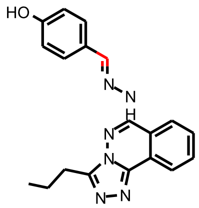 [*]C=[*]	0.0915
**Top Features for negative contribution**			
**Fingerprint**	**Bit/Smiles**	**Feature Structure**	**Score**
FCFP_6	991735244	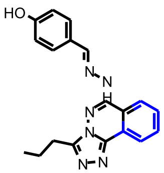 [*]:[c]1:[*]:[cH]:[cH]:[cH]:[cH]:1	-0.134
ECFP_6	1564392544	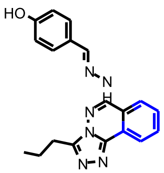 [*]:[c]1:[*]:[cH]:[cH]:[cH]:[cH]:1	-0.133
FCFP_6	1	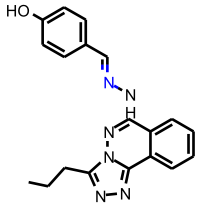 [*]=O	-0.102

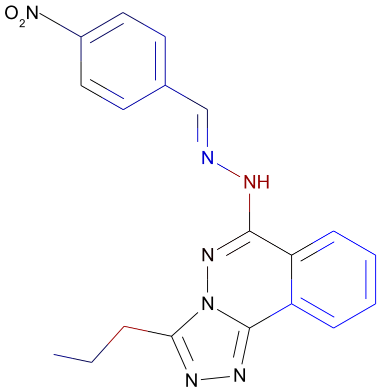

$C_{19}H_{17}N_7O_2$

Molecular Weight: 375.38397

ALogP: 4.197

Rotatable Bonds: 6

Acceptors: 7

Donors: 1

Model Prediction

Prediction: 0.13

Unit: g/kg_body_weight

Mahalanobis Distance: Not Available

Mahalanobis Distance p-value: Not Available

Mahalanobis Distance: The Mahalanobis distance (MD) is a generalization of the Euclidean distance that accounts for correlations among the X properties. It is calculated as the distance to the center of the training data. The larger the MD, the less trustworthy the prediction.

Mahalanobis Distance p-value: The p-value gives the fraction of training data with an MD greater than or equal to the one for the given sample, assuming normally distributed data. The smaller the p-value, the less trustworthy the prediciton. For highly non-normal X properties (e.g., fingerprints), the MD p-value is wildly inaccurate.

Model Applicability

Unknown features are fingerprint features in the query molecule, but not found or appearing too infrequently in the training set.

1. All properties and OPS components are within expected ranges.
2. Unknown FCFP_2 feature: 5: [*][O-]
3. Unknown FCFP_2 feature: -332197802: [*][c]1:[*]:[*]:[c](:[*]):n:1:n:[*]
4. Unknown FCFP_2 feature: 1294285001: [*]=NN[c](:[*]):[*]
5. Unknown FCFP_2 feature: -828984032: [*][N+](=[*])[c](:[cH]:[*]):[cH]:[*]
6. Unknown FCFP_2 feature: -1338588315: [*]:[c](:[*])[N+](=O)[O-]
7. Unknown FCFP_2 feature: 1872392852: [*][N+](=O)[*]
8. Unknown FCFP_2 feature: 260476081: [*][N+](=[*])[O-]
9. Unknown ECFP_6 feature: 672362763: [*]:n(:[*]):[*]
10. Unknown ECFP_6 feature: 1043790491: [*][N+](=[*])[*]
11. Unknown ECFP_6 feature: 781519895: [*][O-]
12. Unknown ECFP_6 feature: 1997021792: [*]:[cH]:[cH]:[cH]:[*]
13. Unknown ECFP_6 feature: 1333660716: [*][c](:[*]):[c](:[cH]:[*]):[c](:[*]):[*]
14. Unknown ECFP_6 feature: 1049768340: [*]N[c](:n:[*]):[c](:[*]):[*]
15. Unknown ECFP_6 feature: 835630791: [*][c](:[*]):n:n(:[*]):[*]
16. Unknown ECFP_6 feature: -1507082173: [*][c]1:[*]:[*]:[c](:[*]):n:1:n:[*]
17. Unknown ECFP_6 feature: 1049075205: [*]:n1:[*]:[*]:n:[c]:1:[c](:[*]):[*]
18. Unknown ECFP_6 feature: -435188506: [*]C[c]1:n:[*]:[*]:n:1:[*]
19. Unknown ECFP_6 feature: 911256832: [*][c]1:[*]:[*]:n:n:1
20. Unknown ECFP_6 feature: -1795525632: [*]CC[c](:[*]):[*]
21. Unknown ECFP_6 feature: -1793471910: [*]CCC
22. Unknown ECFP_6 feature: -1236714312: [*]=NN[c](:[*]):[*]
23. Unknown ECFP_6 feature: 1814278164: [*]N\N=C\[*]
24. Unknown ECFP_6 feature: -1832102709: [*]\N=C\[c](:[*]):[*]
25. Unknown ECFP_6 feature: -176483725: [*]=C[c](:[cH]:[*]):[cH]:[*]
26. Unknown ECFP_6 feature: -179073144: [*][N+](=[*])[c](:[cH]:[*]):[cH]:[*]
27. Unknown ECFP_6 feature: -215026467: [*]:[c](:[*])[N+](=O)[O-]
28. Unknown ECFP_6 feature: 2104376220: [*][N+](=O)[*]
29. Unknown ECFP_6 feature: -659271057: [*][N+](=[*])[O-]

Feature Contribution

Top features for positive contribution

Fingerprint	Bit/Smiles	Feature Structure	Score

ECFP_6	1559650422	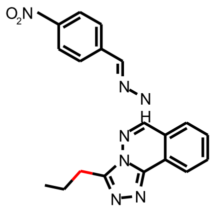 [*]C[*]	0.129
FCFP_6	3	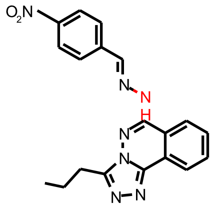 [*]N[*]	0.0924
ECFP_6	-1925046727	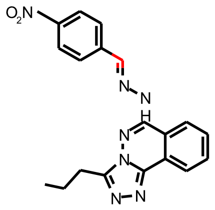 [*]C=[*]	0.0915
**Top Features for negative contribution**			
**Fingerprint**	**Bit/Smiles**	**Feature Structure**	**Score**
FCFP_6	991735244	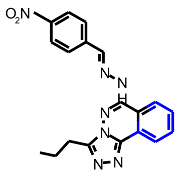 [*]:[c]1:[*]:[cH]:[cH]:[cH]:[cH]:1	-0.134
ECFP_6	1564392544	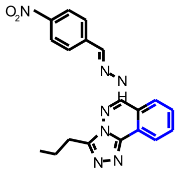 [*]:[c]1:[*]:[cH]:[cH]:[cH]:[cH]:1	-0.133

FCFP_6	1	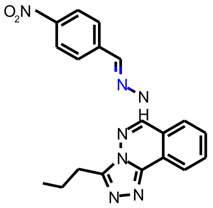 [*]=O	-0.102

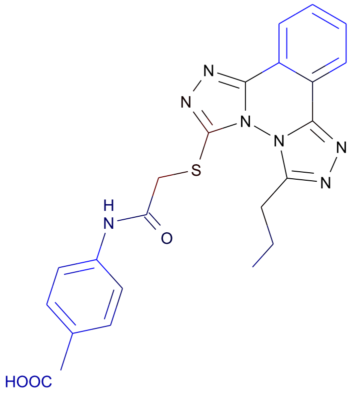

$C_{22}H_{19}N_7O_3S$
Molecular Weight: 461.49635
ALogP: 3.42
Rotatable Bonds: 7
Acceptors: 8
Donors: 2

Model Prediction

Prediction: 0.212
Unit: g/kg_body_weight
Mahalanobis Distance: Not Available
Mahalanobis Distance p-value: Not Available

Mahalanobis Distance: The Mahalanobis distance (MD) is a generalization of the Euclidean distance that accounts for correlations among the X properties. It is calculated as the distance to the center of the training data. The larger the MD, the less trustworthy the prediction.
Mahalanobis Distance p-value: The p-value gives the fraction of training data with an MD greater than or equal to the one for the given sample, assuming normally distributed data. The smaller the p-value, the less trustworthy the prediciton. For highly non-normal X properties (e.g., fingerprints), the MD p-value is wildly inaccurate.

Model Applicability

Unknown features are fingerprint features in the query molecule, but not found or appearing too infrequently in the training set.

1. All properties and OPS components are within expected ranges.
2. Unknown FCFP_2 feature: -332197802: [*][c]1:[*]:[*]:[c](:[*]):n:1:n:[*]
3.
4.
5.
6.
7.
8. Unknown ECFP_6 feature: -830332112: [*]S[*]
9. Unknown ECFP_6 feature: 672362763: [*]:n(:[*]):[*]
10. Unknown ECFP_6 feature: 1731843802: [*]CC(=O)N[*]
11. Unknown ECFP_6 feature: -955816473: [*]SCC(=[*])[*]
12. Unknown ECFP_6 feature: 1427820655: [*]CS[c](:[*]):[*]
13. Unknown ECFP_6 feature: 1986731747: [*]S[c]1:n:[*]:[*]:n:1:[*]
14. Unknown ECFP_6 feature: 911256832: [*][c]1:[*]:[*]:n:n:1
15. Unknown ECFP_6 feature: -1793471910: [*]CCC
16. Unknown ECFP_6 feature: -1795525632: [*]CC[c](:[*]):[*]
17. Unknown ECFP_6 feature: -435188506: [*]C[c]1:n:[*]:[*]:n:1:[*]
18. Unknown ECFP_6 feature: 1049075205: [*]:n1:[*]:[*]:n:[c]:1:[c](:[*]):[*]
19. Unknown ECFP_6 feature: 78665610: [*][c]1:[*]:[*]:[c](:[*]):n:1:n(:[*]):[*]
20. Unknown ECFP_6 feature: 1333660716: [*][c](:[*]):[c](:[cH]:[*]):[c](:[*]):[*]
21. Unknown ECFP_6 feature: 1997021792: [*]:[cH]:[cH]:[cH]:[*]
22. Unknown ECFP_6 feature: -177077903: [*]N[c](:[cH]:[*]):[cH]:[*]
23. Unknown ECFP_6 feature: -175146122: [*]C(=[*])[c](:[cH]:[*]):[cH]:[*]
24. Unknown ECFP_6 feature: 1429461619: [*]:[c](:[*])C(=O)O

Feature Contribution

Top features for positive contribution

Fingerprint	Bit/Smiles	Feature Structure	Score
ECFP_6	1559650422	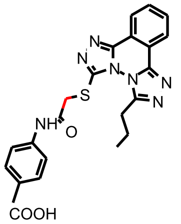 [*]C[*]	0.129

FCFP_6	3	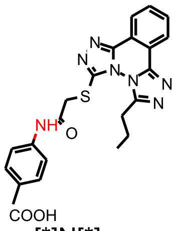<br>[*]N[*]	0.0924
ECFP_6	2099970318	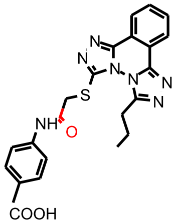<br>[*]C(=O)[*]	0.0766
**Top Features for negative contribution**			
**Fingerprint**	**Bit/Smiles**	**Feature Structure**	**Score**
FCFP_6	991735244	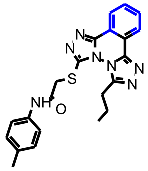<br>[*]:[c]1:[*]:[cH]:[cH]:[cH]:[cH]:1	-0.134
ECFP_6	1564392544	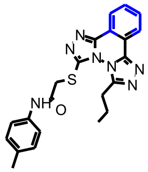<br>[*]:[c]1:[*]:[cH]:[cH]:[cH]:[cH]:1	-0.133
FCFP_6	1	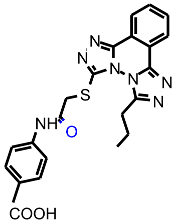<br>[*]=O	-0.102

14b

TOPKAT_Chronic_LOAEL

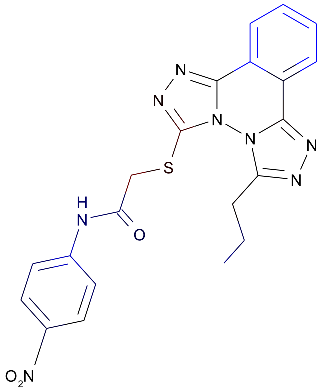

$C_{21}H_{18}N_8O_3S$

Molecular Weight: 462.48441

ALogP: 3.685

Rotatable Bonds: 7

Acceptors: 8

Donors: 1

Model Prediction

Prediction: 0.0948

Unit: g/kg_body_weight

Mahalanobis Distance: Not Available

Mahalanobis Distance p-value: Not Available

Mahalanobis Distance: The Mahalanobis distance (MD) is a generalization of the Euclidean distance that accounts for correlations among the X properties. It is calculated as the distance to the center of the training data. The larger the MD, the less trustworthy the prediction.

Mahalanobis Distance p-value: The p-value gives the fraction of training data with an MD greater than or equal to the one for the given sample, assuming normally distributed data. The smaller the p-value, the less trustworthy the prediciton. For highly non-normal X properties (e.g., fingerprints), the MD p-value is wildly inaccurate.

Model Applicability

Unknown features are fingerprint features in the query molecule, but not found or appearing too infrequently in the training set.

1. All properties and OPS components are within expected ranges.
2. Unknown FCFP_2 feature: 5: [*][O-]
3. Unknown FCFP_2 feature: -332197802: [*][c]1:[*]:[*]:[c](:[*]):n:1:n:[*]
4. Unknown FCFP_2 feature: -828984032: [*][N+](=[*])[c](:[cH]:[*]):[cH]:[*]
5. Unknown FCFP_2 feature: -1338588315: [*]:[c](:[*])[N+](=O)[O-]
6. Unknown FCFP_2 feature: 1872392852: [*][N+](=O)[*]
7. Unknown FCFP_2 feature: 260476081: [*][N+](=[*])[O-]
8. Unknown ECFP_6 feature: -830332112: [*]S[*]
9. Unknown ECFP_6 feature: 672362763: [*]:n(:[*]):[*]
10. Unknown ECFP_6 feature: 1043790491: [*][N+](=[*])[*]
11. Unknown ECFP_6 feature: 781519895: [*][O-]
12. Unknown ECFP_6 feature: 1731843802: [*]CC(=O)N[*]
13. Unknown ECFP_6 feature: -955816473: [*]SCC(=[*])[*]
14. Unknown ECFP_6 feature: 1427820655: [*]CS[c](:[*]):[*]
15. Unknown ECFP_6 feature: 1986731747: [*]S[c]1:n:[*]:[*]:n:1:[*]
16. Unknown ECFP_6 feature: 911256832: [*][c]1:[*]:[*]:n:n:1
17. Unknown ECFP_6 feature: -1793471910: [*]CCC
18. Unknown ECFP_6 feature: -1795525632: [*]CC[c](:[*]):[*]
19. Unknown ECFP_6 feature: -435188506: [*]C[c]1:n:[*]:[*]:n:1:[*]
20. Unknown ECFP_6 feature: 1049075205: [*]:n1:[*]:[*]:n:[c]:1:[c](:[*]):[*]
21. Unknown ECFP_6 feature: 78665610: [*][c]1:[*]:[*]:[c](:[*]):n:1:n(:[*]):[*]
22. Unknown ECFP_6 feature: 1333660716: [*][c](:[*]):[c](:[cH]:[*]):[c](:[*]):[*]
23. Unknown ECFP_6 feature: 1997021792: [*]:[cH]:[cH]:[cH]:[*]
24. Unknown ECFP_6 feature: -177077903: [*]N[c](:[cH]:[*]):[cH]:[*]
25. Unknown ECFP_6 feature: -179073144: [*][N+](=[*])[c](:[cH]:[*]):[cH]:[*]
26. Unknown ECFP_6 feature: -215026467: [*]:[c](:[*])[N+](=O)[O-]
27. Unknown ECFP_6 feature: 2104376220: [*][N+](=O)[*]
28. Unknown ECFP_6 feature: -659271057: [*][N+](=[*])[O-]

Feature Contribution

Top features for positive contribution			
Fingerprint	Bit/Smiles	Feature Structure	Score

ECFP_6	1559650422	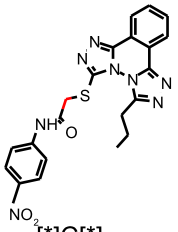 [*]C[*]	0.129
FCFP_6	3	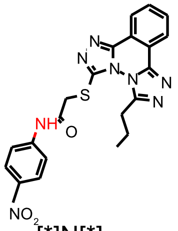 [*]N[*]	0.0924
ECFP_6	2099970318	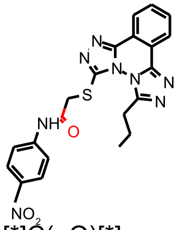 [*]C(=O)[*]	0.0766

Top Features for negative contribution

**Fingerprint**	**Bit/Smiles**	**Feature Structure**	**Score**
FCFP_6	991735244	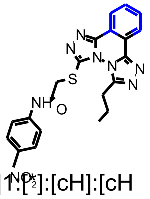 [*]:[c]1:[*]:[cH]:[cH]:[cH]:[cH]:1	-0.134
ECFP_6	1564392544	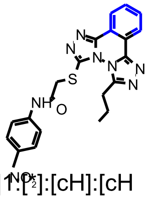 [*]:[c]1:[*]:[cH]:[cH]:[cH]:[cH]:1	-0.133

FCFP_6	1	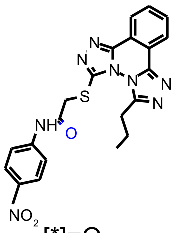 [*]=O	-0.102

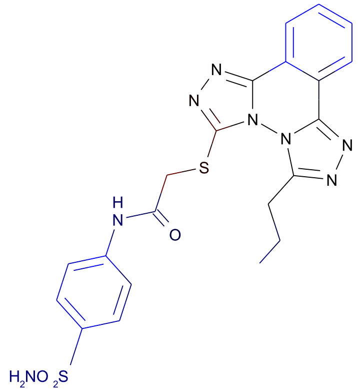

$C_{21}H_{20}N_8O_3S_2$
Molecular Weight: 496.56529
ALogP: 2.496
Rotatable Bonds: 7
Acceptors: 8
Donors: 2

Model Prediction

Prediction: 0.17
Unit: g/kg_body_weight
Mahalanobis Distance: Not Available
Mahalanobis Distance p-value: Not Available

Mahalanobis Distance: The Mahalanobis distance (MD) is a generalization of the Euclidean distance that accounts for correlations among the X properties. It is calculated as the distance to the center of the training data. The larger the MD, the less trustworthy the prediction.
Mahalanobis Distance p-value: The p-value gives the fraction of training data with an MD greater than or equal to the one for the given sample, assuming normally distributed data. The smaller the p-value, the less trustworthy the prediciton. For highly non-normal X properties (e.g., fingerprints), the MD p-value is wildly inaccurate.

Model Applicability

Unknown features are fingerprint features in the query molecule, but not found or appearing too infrequently in the training set.

1. All properties and OPS components are within expected ranges.
2. Unknown FCFP_2 feature: -332197802: [*][c]1:[*]:[*]:[c](:[*]):n:1:n:[*]
3.
4.
5.
6.
7.
8. Unknown ECFP_6 feature: -830332112: [*]S[*]
9. Unknown ECFP_6 feature: 672362763: [*]:n(:[*]):[*]
10. Unknown ECFP_6 feature: -797085356: [*]S(=[*])(=[*])[*]
11. Unknown ECFP_6 feature: 1731843802: [*]CC(=O)N[*]
12. Unknown ECFP_6 feature: -955816473: [*]SCC(=[*])[*]
13. Unknown ECFP_6 feature: 1427820655: [*]CS[c](:[*]):[*]
14. Unknown ECFP_6 feature: 1986731747: [*]S[c]1:n:[*]:[*]:n:1:[*]
15. Unknown ECFP_6 feature: 911256832: [*][c]1:[*]:[*]:n:n:1
16. Unknown ECFP_6 feature: -1793471910: [*]CCC
17. Unknown ECFP_6 feature: -1795525632: [*]CC[c](:[*]):[*]
18. Unknown ECFP_6 feature: -435188506: [*]C[c]1:n:[*]:[*]:n:1:[*]
19. Unknown ECFP_6 feature: 1049075205: [*]:n1:[*]:[*]:n:[c]:1:[c](:[*]):[*]
20. Unknown ECFP_6 feature: 78665610: [*][c]1:[*]:[*]:[c](:[*]):n:1:n(:[*]):[*]
21. Unknown ECFP_6 feature: 1333660716: [*][c](:[*]):[c](:[cH]:[*]):[c](:[*]):[*]
22. Unknown ECFP_6 feature: 1997021792: [*]:[cH]:[cH]:[cH]:[*]
23. Unknown ECFP_6 feature: -177077903: [*]N[c](:[cH]:[*]):[cH]:[*]
24. Unknown ECFP_6 feature: -177264675: [*]S(=[*])(=[*])[c](:[cH]:[*]):[cH]:[*]
25. Unknown ECFP_6 feature: -2121766239: [*]:[c](:[*])S(=O)(=O)N
26. Unknown ECFP_6 feature: 2102150379: [*]S(=[*])(=O)[*]
27. Unknown ECFP_6 feature: -934226723: [*]S(=[*])(=[*])N

Feature Contribution

Top features for positive contribution			
Fingerprint	Bit/Smiles	Feature Structure	Score

ECFP_6	1559650422	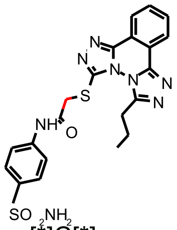<br>[*]C[*]	0.129
FCFP_6	3	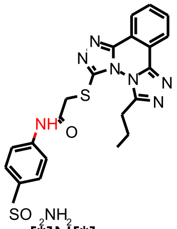<br>[*]N[*]	0.0924
ECFP_6	2099970318	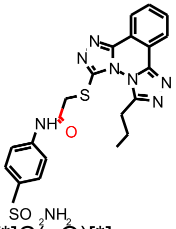<br>[*]C(=O)[*]	0.0766
**Top Features for negative contribution**			
**Fingerprint**	**Bit/Smiles**	**Feature Structure**	**Score**
FCFP_6	991735244	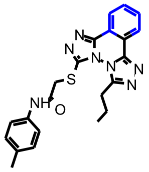<br>[*]:[c]1:[*]:[cH]:[cH<br>]:[cH]:[cH]:1	-0.134
ECFP_6	1564392544	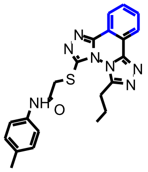<br>[*]:[c]1:[*]:[cH]:[cH<br>]:[cH]:[cH]:1	-0.133

FCFP_6	1	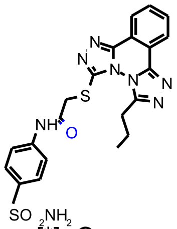<br>[*]=O	-0.102

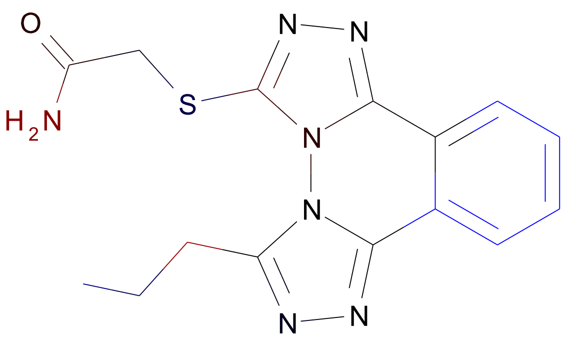

$C_{15}H_{15}N_7OS$
Molecular Weight: 341.39089
ALogP: 2.008
Rotatable Bonds: 5
Acceptors: 6
Donors: 1

Model Prediction

Prediction: 0.0849
Unit: g/kg_body_weight
Mahalanobis Distance: Not Available
Mahalanobis Distance p-value: Not Available

Mahalanobis Distance: The Mahalanobis distance (MD) is a generalization of the Euclidean distance that accounts for correlations among the X properties. It is calculated as the distance to the center of the training data. The larger the MD, the less trustworthy the prediction.
Mahalanobis Distance p-value: The p-value gives the fraction of training data with an MD greater than or equal to the one for the given sample, assuming normally distributed data. The smaller the p-value, the less trustworthy the prediciton. For highly non-normal X properties (e.g., fingerprints), the MD p-value is wildly inaccurate.

Model Applicability

Unknown features are fingerprint features in the query molecule, but not found or appearing too infrequently in the training set.

1. All properties and OPS components are within expected ranges.
2. Unknown FCFP_2 feature: -332197802: [*][c]1:[*]:[*]:[c](:[*]):n:1:n:[*]
3.
4.
5.
6.
7.
8. Unknown ECFP_6 feature: 672362763: [*]:n(:[*]):[*]
9. Unknown ECFP_6 feature: -830332112: [*]S[*]
10. Unknown ECFP_6 feature: 1997021792: [*]:[cH]:[cH]:[cH]:[*]
11. Unknown ECFP_6 feature: 1333660716: [*][c](:[*]):[c](:[cH]:[*]):[c](:[*]):[*]
12. Unknown ECFP_6 feature: 1049075205: [*]:n1:[*]:[*]:n:[c]:1:[c](:[*]):[*]
13. Unknown ECFP_6 feature: 78665610: [*][c]1:[*]:[*]:[c](:[*]):n:1:n(:[*]):[*]
14. Unknown ECFP_6 feature: -435188506: [*]C[c]1:n:[*]:[*]:n:1:[*]
15. Unknown ECFP_6 feature: 911256832: [*][c]1:[*]:[*]:n:n:1
16. Unknown ECFP_6 feature: -1795525632: [*]CC[c](:[*]):[*]
17. Unknown ECFP_6 feature: -1793471910: [*]CCC
18. Unknown ECFP_6 feature: 1986731747: [*]S[c]1:n:[*]:[*]:n:1:[*]
19. Unknown ECFP_6 feature: 1427820655: [*]CS[c](:[*]):[*]
20. Unknown ECFP_6 feature: -955816473: [*]SCC(=[*])[*]
21. Unknown ECFP_6 feature: -1708545601: [*]CC(=O)N
22. Unknown ECFP_6 feature: -932108170: [*]C(=[*])N

Feature Contribution

Top features for positive contribution			
Fingerprint	Bit/Smiles	Feature Structure	Score
ECFP_6	1559650422	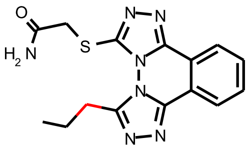 [*]C[*]	0.129

FCFP_6	3	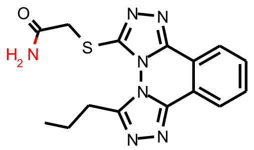 [*]N[*]	0.0924
ECFP_6	2099970318	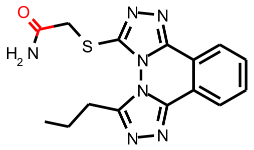 [*]C(=O)[*]	0.0766

Top Features for negative contribution

Fingerprint	Bit/Smiles	Feature Structure	Score
FCFP_6	991735244	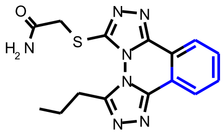 [*]:[c]1:[*]:[cH]:[cH]:[cH]:[cH]:1	-0.134
ECFP_6	1564392544	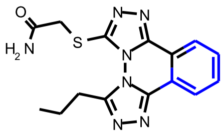 [*]:[c]1:[*]:[cH]:[cH]:[cH]:[cH]:1	-0.133
FCFP_6	1	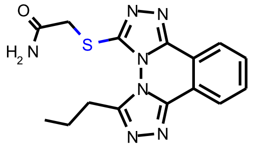 [*]=O	-0.102

Doxorubicin

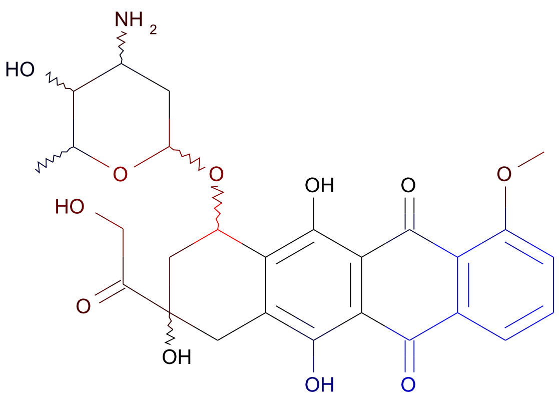

$C_{27}H_{29}NO_{11}$

Molecular Weight: 543.51925

ALogP: -4.4e-002

Rotatable Bonds: 5

Acceptors: 12

Donors: 6

Model Prediction

Prediction: 0.013

Unit: g/kg_body_weight

Mahalanobis Distance: Not Available

Mahalanobis Distance p-value: Not Available

Mahalanobis Distance: The Mahalanobis distance (MD) is a generalization of the Euclidean distance that accounts for correlations among the X properties. It is calculated as the distance to the center of the training data. The larger the MD, the less trustworthy the prediction.

Mahalanobis Distance p-value: The p-value gives the fraction of training data with an MD greater than or equal to the one for the given sample, assuming normally distributed data. The smaller the p-value, the less trustworthy the prediciton. For highly non-normal X properties (e.g., fingerprints), the MD p-value is wildly inaccurate.

Model Applicability

Unknown features are fingerprint features in the query molecule, but not found or appearing too infrequently in the training set.

1. All properties and OPS components are within expected ranges.
2.
3.
4.
5.
6.
7.
8. Unknown ECFP_6 feature: 1997021792: [*]:[cH]:[cH]:[cH]:[*]
9. Unknown ECFP_6 feature: -813997308: [*]C(=[*])[c](:[c]([*]):[*]):[c]([*]):[*]
10. Unknown ECFP_6 feature: 1717462980: [*]:[c](:[*])C(=O)[c](:[*]):[*]
11. Unknown ECFP_6 feature: -1660913849: [*][c](:[*]):[c](O):[c]([*]):[*]
12. Unknown ECFP_6 feature: -1659009760: [*]C([*])[c](:[c]([*]):[*]):[c]([*]):[*]
13. Unknown ECFP_6 feature: -1660340418: [*]C[c](:[c]([*]):[*]):[c]([*]):[*]
14. Unknown ECFP_6 feature: 1123660302: [*]CC(O[*])[c](:[*]):[*]
15. Unknown ECFP_6 feature: 413587124: [*]C([*])CC([*])([*])[*]
16. Unknown ECFP_6 feature: 140080459: [*]CC(O)(C[*])C(=[*])[*]
17. Unknown ECFP_6 feature: 407900312: [*]C([*])([*])C[c](:[*]):[*]
18. Unknown ECFP_6 feature: 1307307440: [*]:[c](:[*])OC
19. Unknown ECFP_6 feature: 2019062761: [*]:[c](:[*])O
20. Unknown ECFP_6 feature: 456242574: [*]C([*])OC([*])[*]
21. Unknown ECFP_6 feature: -1783281539: [*]CC(=O)C([*])([*])[*]
22. Unknown ECFP_6 feature: -1907393688: [*]C(=[*])CO
23. Unknown ECFP_6 feature: 2022454958: [*]CO
24. Unknown ECFP_6 feature: 2018700401: [*]C([*])([*])O
25. Unknown ECFP_6 feature: 1535429263: [*]OC(C)C([*])[*]
26. Unknown ECFP_6 feature: 305695353: [*]C([*])C(O)C([*])[*]
27. Unknown ECFP_6 feature: 1201786014: [*]CC(N)C([*])[*]
28. Unknown ECFP_6 feature: -801490360: [*]C([*])CC([*])[*]
29. Unknown ECFP_6 feature: -1409796893: [*]C([*])OC([*])[*]
30. Unknown ECFP_6 feature: -932844120: [*]C([*])N
31. Unknown ECFP_6 feature: 865482986: [*]C([*])C
32. Unknown ECFP_6 feature: 2024749573: [*]C([*])O
33. Unknown ECFP_6 feature: -2061744983: [*]CC(O[*])O[*]

Feature Contribution

Top features for positive contribution			
**Fingerprint**	**Bit/Smiles**	**Feature Structure**	**Score**

ECFP_6	-167460056	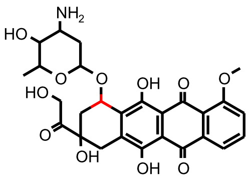 [*]C([*])[*]	0.136
FCFP_6	-1143715940	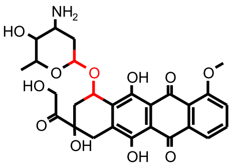 [*]C([*])OC([*])[*]	0.13
ECFP_6	1559650422	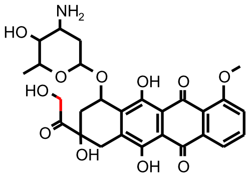 [*]C[*]	0.129
**Top Features for negative contribution**			
**Fingerprint**	**Bit/Smiles**	**Feature Structure**	**Score**
FCFP_6	991735244	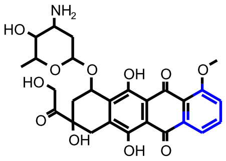 [*]:[c]1:[*]:[cH]:[cH]:[cH]:[cH]:1	-0.134
ECFP_6	2106656448	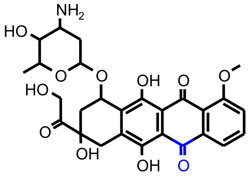 [*]C(=O)[*]	-0.11

FCFP_6	1	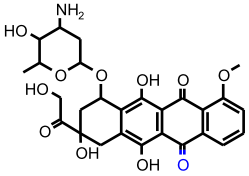 [*]=O	-0.102

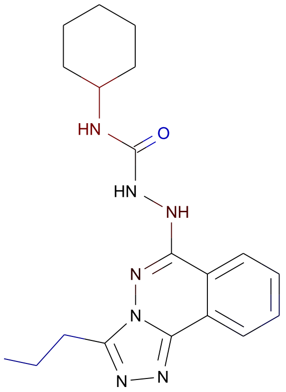

$C_{19}H_{25}N_7O$
Molecular Weight: 367.44809
ALogP: 3.681
Rotatable Bonds: 5
Acceptors: 5
Donors: 3

Model Prediction

Prediction: 0.222
Unit: g/kg_body_weight
Mahalanobis Distance: 8.93
Mahalanobis Distance p-value: 0.00177

Mahalanobis Distance: The Mahalanobis distance (MD) is a generalization of the Euclidean distance that accounts for correlations among the X properties. It is calculated as the distance to the center of the training data. The larger the MD, the less trustworthy the prediction.
Mahalanobis Distance p-value: The p-value gives the fraction of training data with an MD greater than or equal to the one for the given sample, assuming normally distributed data. The smaller the p-value, the less trustworthy the prediciton. For highly non-normal X properties (e.g., fingerprints), the MD p-value is wildly inaccurate.

Structural Similar Compounds

Name	PHENOLPHTHALEIN	DISPERSE YELLOW 3	C.I.PIGMENT RED 3
Structure	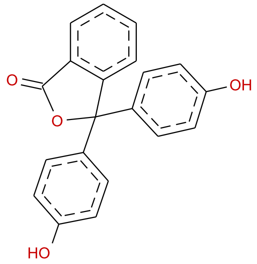	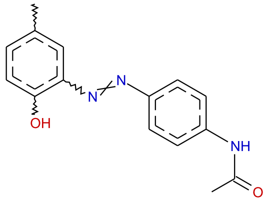	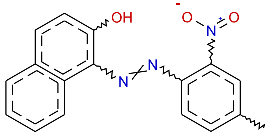
Actual Endpoint (-log C)	2.20184	2.77703	2.65635
Predicted Endpoint (-log C)	2.8857	2.80195	2.97957
Distance	0.695	0.739	0.754
Reference	NCI/NTP TR-465	NCI/NTP TR-222	NCI/NTP TR-407

Model Applicability

Unknown features are fingerprint features in the query molecule, but not found or appearing too infrequently in the training set.

1. All properties and OPS components are within expected ranges.
2. Unknown FCFP_2 feature: -332197802: [*][c]1:[*]:[*]:[c](:[*]):n:1:n:[*]
3. Unknown FCFP_2 feature: -885461129: [*]NNC(=[*])[*]

Feature Contribution

Top features for positive contribution

Fingerprint	Bit/Smiles	Feature Structure	Score
FCFP_2	-885550502	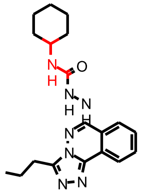 [*]C([*])NC(=[*])[*]	0.115

FCFP_2	3	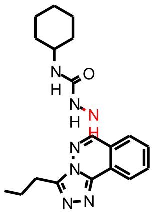 [*]N[*]	0.0737
FCFP_2	17	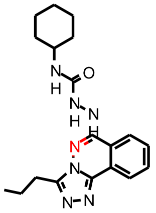 [*]:n:[*]	0.0441
**Top Features for negative contribution**			
**Fingerprint**	**Bit/Smiles**	**Feature Structure**	**Score**
FCFP_2	-1272798659	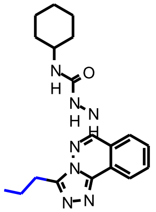 [*]CCC	-0.111
FCFP_2	1872154524	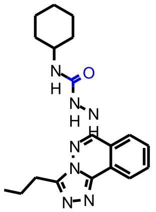 [*]C(=O)[*]	-0.105
FCFP_2	1	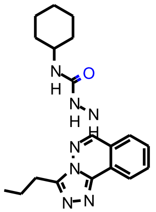 [*]=O	-0.0796

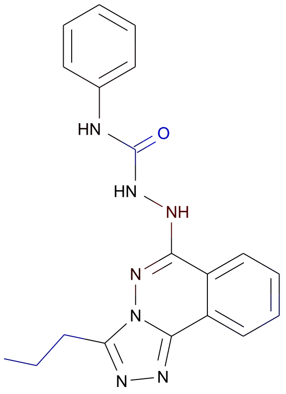

$C_{19}H_{19}N_7O$
Molecular Weight: 361.40046
ALogP: 3.399
Rotatable Bonds: 5
Acceptors: 5
Donors: 3

Model Prediction

Prediction: 0.369
Unit: g/kg_body_weight
Mahalanobis Distance: 10.5
Mahalanobis Distance p-value: 5.88e-006

Mahalanobis Distance: The Mahalanobis distance (MD) is a generalization of the Euclidean distance that accounts for correlations among the X properties. It is calculated as the distance to the center of the training data. The larger the MD, the less trustworthy the prediction.
Mahalanobis Distance p-value: The p-value gives the fraction of training data with an MD greater than or equal to the one for the given sample, assuming normally distributed data. The smaller the p-value, the less trustworthy the prediciton. For highly non-normal X properties (e.g., fingerprints), the MD p-value is wildly inaccurate.

Structural Similar Compounds

Name	PHENOLPHTHALEIN	C.I.PIGMENT RED 3	SALICYLAZOSULFAPYRIDINE
Structure	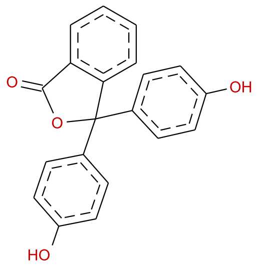	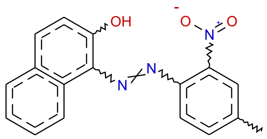	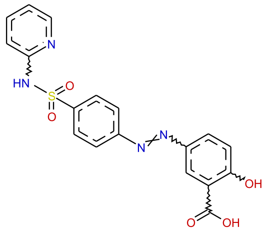
Actual Endpoint (-log C)	2.20184	2.65635	3.375
Predicted Endpoint (-log C)	2.8857	2.97957	2.80292
Distance	0.765	0.818	0.837
Reference	NCI/NTP TR-465	NCI/NTP TR-407	NCI/NTP TR-457

Model Applicability

Unknown features are fingerprint features in the query molecule, but not found or appearing too infrequently in the training set.

1. All properties and OPS components are within expected ranges.
2. Unknown FCFP_2 feature: -332197802: [*][c]1:[*]:[*]:[c](:[*]):n:1:n:[*]
3. Unknown FCFP_2 feature: -885461129: [*]NNC(=[*])[*]

Feature Contribution

Top features for positive contribution			
**Fingerprint**	**Bit/Smiles**	**Feature Structure**	**Score**
FCFP_2	3	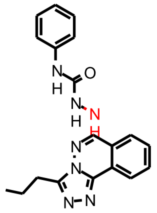 [*]N[*]	0.0737

FCFP_2	17	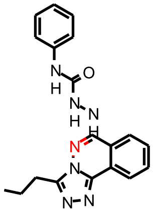<br>[*]:n:[*]	0.0441
FCFP_2	590925877	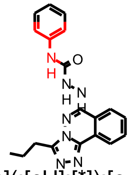<br>[*]N[c](:[cH]:[*]):[cH]:[*]	0.00762

Top Features for negative contribution

Fingerprint	Bit/Smiles	Feature Structure	Score
FCFP_2	-1272798659	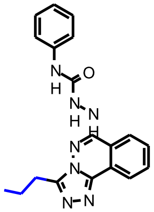<br>[*]CCC	-0.111
FCFP_2	1872154524	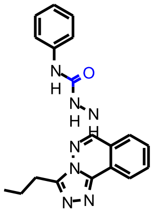<br>[*]C(=O)[*]	-0.105
FCFP_2	1	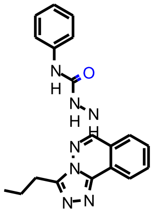<br>[*]=O	-0.0796

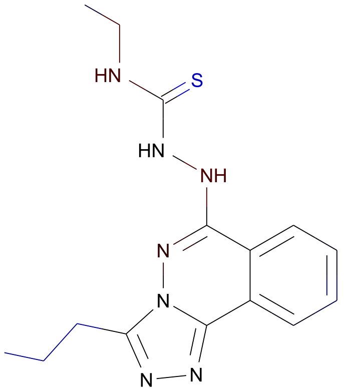

$C_{15}H_{19}N_7S$
Molecular Weight: 329.42325
ALogP: 3.388
Rotatable Bonds: 7
Acceptors: 5
Donors: 3

Model Prediction

Prediction: 0.366
Unit: g/kg_body_weight
Mahalanobis Distance: 8.77
Mahalanobis Distance p-value: 0.00286

Mahalanobis Distance: The Mahalanobis distance (MD) is a generalization of the Euclidean distance that accounts for correlations among the X properties. It is calculated as the distance to the center of the training data. The larger the MD, the less trustworthy the prediction.
Mahalanobis Distance p-value: The p-value gives the fraction of training data with an MD greater than or equal to the one for the given sample, assuming normally distributed data. The smaller the p-value, the less trustworthy the prediciton. For highly non-normal X properties (e.g., fingerprints), the MD p-value is wildly inaccurate.

Structural Similar Compounds

Name	SALICYLAZOSULFAPYRIDINE	FUROSEMIDE	TOLBUTAMIDE
Structure	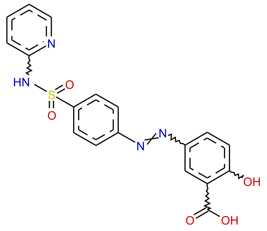	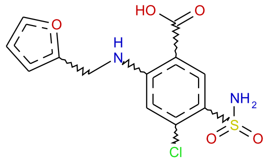	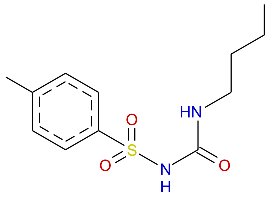
Actual Endpoint (-log C)	3.375	4.04236	2.3985
Predicted Endpoint (-log C)	2.80292	2.8614	3.32272
Distance	0.737	0.751	0.795
Reference	NCI/NTP TR-457	NCI/NTP TR-356	NCI/NTP TR-031

Model Applicability

Unknown features are fingerprint features in the query molecule, but not found or appearing too infrequently in the training set.

1. OPS PC12 out of range. Value: -2.4923. Training min, max, SD, explained variance: -2.364, 2.9228, 1.079, 0.0263.
2. Unknown FCFP_2 feature: -332197802: [*][c]1:[*]:[*]:[c](:[*]):n:1:n:[*]
3. Unknown FCFP_2 feature: -885461129: [*]NNC(=[*])[*]

Feature Contribution

Top features for positive contribution

Fingerprint	Bit/Smiles	Feature Structure	Score
FCFP_2	-885550502	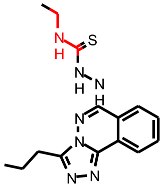 [*]C([*])NC(=[*])[*]	0.115

FCFP_2	3	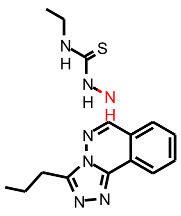 [*]N[*]	0.0737
FCFP_2	17	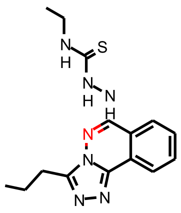 [*]:n:[*]	0.0441
**Top Features for negative contribution**			
**Fingerprint**	**Bit/Smiles**	**Feature Structure**	**Score**
FCFP_2	-1272798659	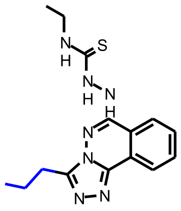 [*]CCC	-0.111
FCFP_2	1872154524	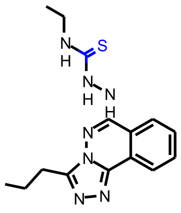 [*]C(=O)[*]	-0.105
FCFP_2	1	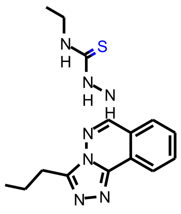 [*]=O	-0.0796

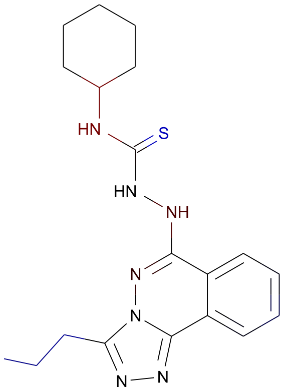

$C_{19}H_{25}N_7S$
Molecular Weight: 383.51369
ALogP: 4.898
Rotatable Bonds: 7
Acceptors: 5
Donors: 3

Model Prediction

Prediction: 0.26
Unit: g/kg_body_weight
Mahalanobis Distance: 8.89
Mahalanobis Distance p-value: 0.00199

Mahalanobis Distance: The Mahalanobis distance (MD) is a generalization of the Euclidean distance that accounts for correlations among the X properties. It is calculated as the distance to the center of the training data. The larger the MD, the less trustworthy the prediction.
Mahalanobis Distance p-value: The p-value gives the fraction of training data with an MD greater than or equal to the one for the given sample, assuming normally distributed data. The smaller the p-value, the less trustworthy the prediciton. For highly non-normal X properties (e.g., fingerprints), the MD p-value is wildly inaccurate.

Structural Similar Compounds

Name	SALICYLAZOSULFAPYRIDINE	C.I.PIGMENT RED 3	ACETOHEXAMIDE
Structure	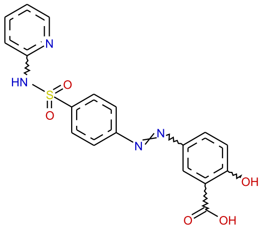	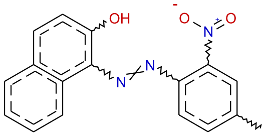	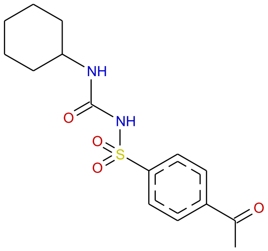
Actual Endpoint (-log C)	3.375	2.65635	2.55683
Predicted Endpoint (-log C)	2.80292	2.97957	3.62413
Distance	0.742	0.802	0.822
Reference	NCI/NTP TR-457	NCI/NTP TR-407	NCI/NTP TR-050

Model Applicability

Unknown features are fingerprint features in the query molecule, but not found or appearing too infrequently in the training set.

1. All properties and OPS components are within expected ranges.
2. Unknown FCFP_2 feature: -332197802: [*][c]1:[*]:[*]:[c](:[*]):n:1:n:[*]
3. Unknown FCFP_2 feature: -885461129: [*]NNC(=[*])[*]

Feature Contribution

Top features for positive contribution

Fingerprint	Bit/Smiles	Feature Structure	Score
FCFP_2	-885550502	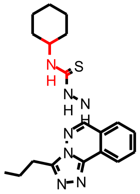 [*]C([*])NC(=[*])[*]	0.115

FCFP_2	3	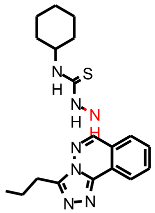 [*]N[*]	0.0737
FCFP_2	17	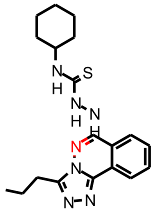 [*]:n:[*]	0.0441

Top Features for negative contribution

Fingerprint	Bit/Smiles	Feature Structure	Score
FCFP_2	-1272798659	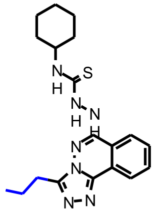 [*]CCC	-0.111
FCFP_2	1872154524	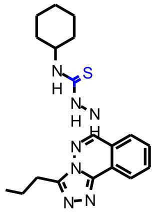 [*]C(=O)[*]	-0.105
FCFP_2	1	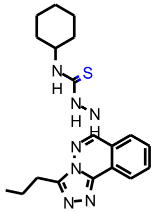 [*]=O	-0.0796

TOPKAT_Rat_Maximum_Tolerated_Dose_Feed

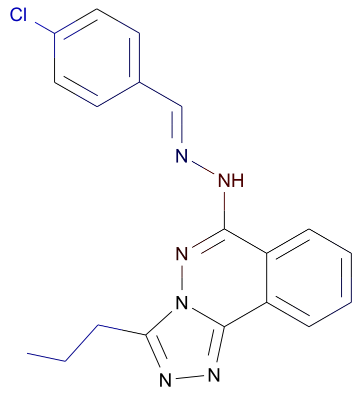

$C_{19}H_{17}ClN_6$
Molecular Weight: 364.83147
ALogP: 4.967
Rotatable Bonds: 5
Acceptors: 5
Donors: 1

Model Prediction

Prediction: 0.29
Unit: g/kg_body_weight
Mahalanobis Distance: 11.7
Mahalanobis Distance p-value: 3.8e-008

Mahalanobis Distance: The Mahalanobis distance (MD) is a generalization of the Euclidean distance that accounts for correlations among the X properties. It is calculated as the distance to the center of the training data. The larger the MD, the less trustworthy the prediction.
Mahalanobis Distance p-value: The p-value gives the fraction of training data with an MD greater than or equal to the one for the given sample, assuming normally distributed data. The smaller the p-value, the less trustworthy the prediciton. For highly non-normal X properties (e.g., fingerprints), the MD p-value is wildly inaccurate.

Structural Similar Compounds

Name	C.I.PIGMENT RED 3	PHENOLPHTHALEIN	CHLORBENZILATE
Structure	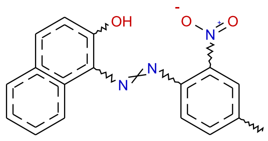	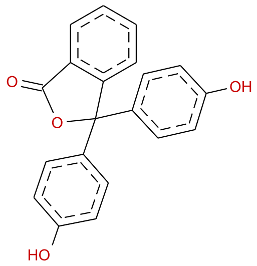	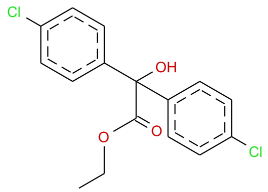
Actual Endpoint (-log C)	2.65635	2.20184	3.38252
Predicted Endpoint (-log C)	2.97957	2.8857	3.27894
Distance	0.716	0.761	0.788
Reference	NCI/NTP TR-407	NCI/NTP TR-465	NCI/NTP TR-75

Model Applicability

Unknown features are fingerprint features in the query molecule, but not found or appearing too infrequently in the training set.

1. All properties and OPS components are within expected ranges.
2. Unknown FCFP_2 feature: -332197802: [*][c]1:[*]:[*]:[c](:[*]):n:1:n:[*]
3. Unknown FCFP_2 feature: 1294285001: [*]=NN[c](:[*]):[*]

Feature Contribution

Top features for positive contribution

Fingerprint	Bit/Smiles	Feature Structure	Score
FCFP_2	3	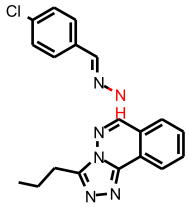 [*]N[*]	0.0737

FCFP_2	17	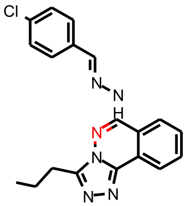 [*]:n:[*]	0.0441
**Top Features for negative contribution**			
**Fingerprint**	**Bit/Smiles**	**Feature Structure**	**Score**
FCFP_2	71476542	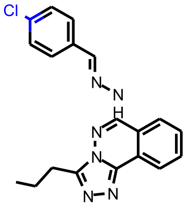 [*]:[c](:[*])Cl	-0.134
FCFP_2	-1272798659	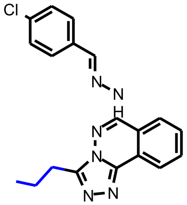 [*]CCC	-0.111
FCFP_2	203677720	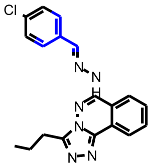 [*]=C[c](:[cH]:[*]):[cH]:[*]	-0.0829

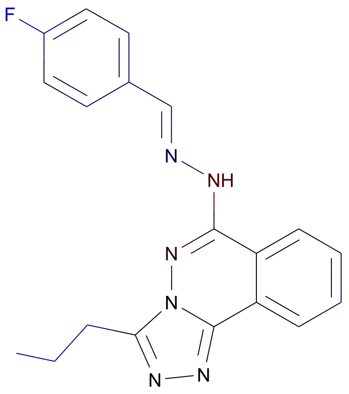

$C_{19}H_{17}FN_6$
Molecular Weight: 348.37688
ALogP: 4.508
Rotatable Bonds: 5
Acceptors: 5
Donors: 1

Model Prediction

Prediction: 0.311
Unit: g/kg_body_weight
Mahalanobis Distance: 11.9
Mahalanobis Distance p-value: 2.12e-008

Mahalanobis Distance: The Mahalanobis distance (MD) is a generalization of the Euclidean distance that accounts for correlations among the X properties. It is calculated as the distance to the center of the training data. The larger the MD, the less trustworthy the prediction.
Mahalanobis Distance p-value: The p-value gives the fraction of training data with an MD greater than or equal to the one for the given sample, assuming normally distributed data. The smaller the p-value, the less trustworthy the prediciton. For highly non-normal X properties (e.g., fingerprints), the MD p-value is wildly inaccurate.

Structural Similar Compounds

Name	C.I.PIGMENT RED 3	PHENOLPHTHALEIN	CHLORBENZILATE
Structure	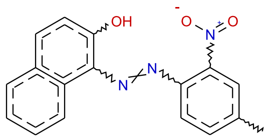	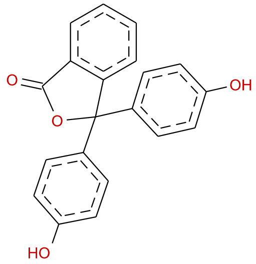	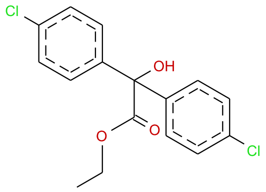
Actual Endpoint (-log C)	2.65635	2.20184	3.38252
Predicted Endpoint (-log C)	2.97957	2.8857	3.27894
Distance	0.715	0.748	0.781
Reference	NCI/NTP TR-407	NCI/NTP TR-465	NCI/NTP TR-75

Model Applicability

Unknown features are fingerprint features in the query molecule, but not found or appearing too infrequently in the training set.

1. All properties and OPS components are within expected ranges.
2. Unknown FCFP_2 feature: -332197802: [*][c]1:[*]:[*]:[c](:[*]):n:1:n:[*]
3. Unknown FCFP_2 feature: 1294285001: [*]=NN[c](:[*]):[*]

Feature Contribution

Top features for positive contribution

Fingerprint	Bit/Smiles	Feature Structure	Score
FCFP_2	3	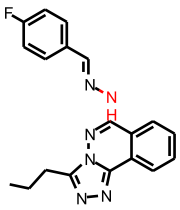 [*]N[*]	0.0737

FCFP_2	17	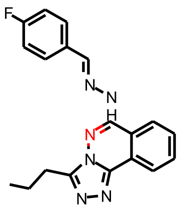 [*]:n:[*]	0.0441
**Top Features for negative contribution**			
**Fingerprint**	**Bit/Smiles**	**Feature Structure**	**Score**
FCFP_2	71476542	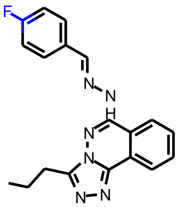 [*]:[c](:[*])Cl	-0.134
FCFP_2	-1272798659	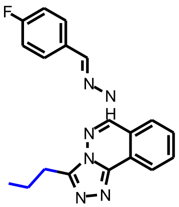 [*]CCC	-0.111
FCFP_2	203677720	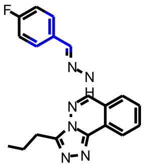 [*]=C[c](:[cH]:[*]):[cH]:[*]	-0.0829

TOPKAT_Rat_Maximum_Tolerated_Dose_Feed

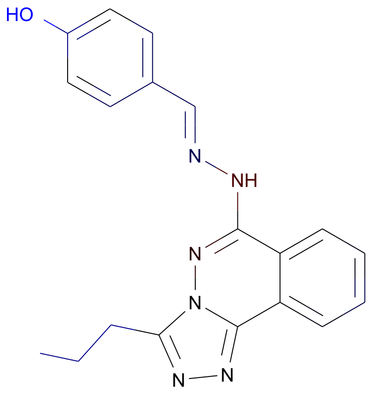

$C_{19}H_{18}N_6O$

Molecular Weight: 346.38582

ALogP: 4.061

Rotatable Bonds: 5

Acceptors: 6

Donors: 2

Model Prediction

Prediction: 0.779

Unit: g/kg_body_weight

Mahalanobis Distance: 10.6

Mahalanobis Distance p-value: 4.05e-006

Mahalanobis Distance: The Mahalanobis distance (MD) is a generalization of the Euclidean distance that accounts for correlations among the X properties. It is calculated as the distance to the center of the training data. The larger the MD, the less trustworthy the prediction.
Mahalanobis Distance p-value: The p-value gives the fraction of training data with an MD greater than or equal to the one for the given sample, assuming normally distributed data. The smaller the p-value, the less trustworthy the prediciton. For highly non-normal X properties (e.g., fingerprints), the MD p-value is wildly inaccurate.

Structural Similar Compounds

Name	C.I.PIGMENT RED 3	PHENOLPHTHALEIN	DISPERSE YELLOW 3
Structure	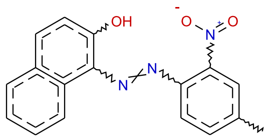	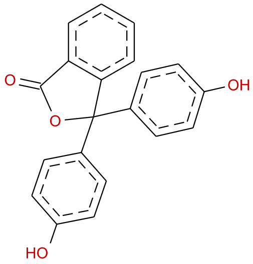	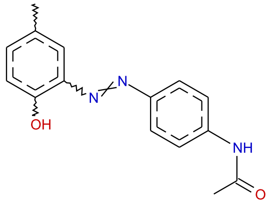
Actual Endpoint (-log C)	2.65635	2.20184	2.77703
Predicted Endpoint (-log C)	2.97957	2.8857	2.80195
Distance	0.654	0.675	0.814
Reference	NCI/NTP TR-407	NCI/NTP TR-465	NCI/NTP TR-222

Model Applicability

Unknown features are fingerprint features in the query molecule, but not found or appearing too infrequently in the training set.

1. All properties and OPS components are within expected ranges.
2. Unknown FCFP_2 feature: -332197802: [*][c]1:[*]:[*]:[c](:[*]):n:1:n:[*]
3. Unknown FCFP_2 feature: 1294285001: [*]=NN[c](:[*]):[*]

Feature Contribution

Top features for positive contribution			
**Fingerprint**	**Bit/Smiles**	**Feature Structure**	**Score**
FCFP_2	3	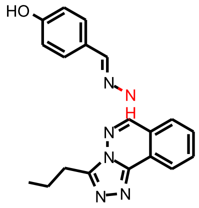 [*]N[*]	0.0737

FCFP_2	17	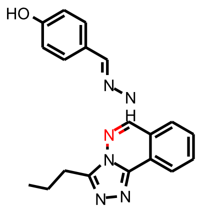 [*]:n:[*]	0.0441

Top Features for negative contribution			
**Fingerprint**	**Bit/Smiles**	**Feature Structure**	**Score**
FCFP_2	7	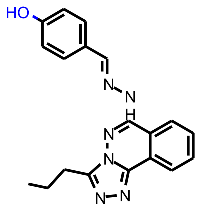 [*]O	-0.214
FCFP_2	-549108873	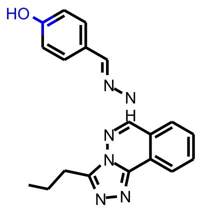 [*]:[c](:[*])O	-0.127
FCFP_2	-1272798659	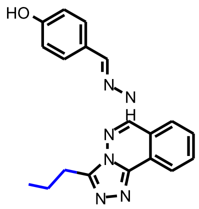 [*]CCC	-0.111

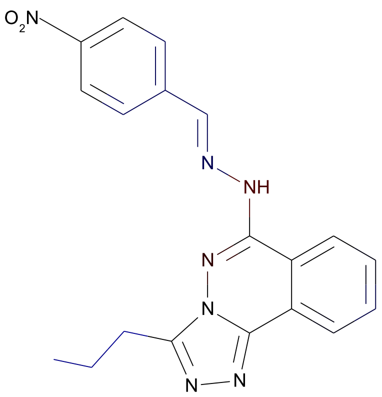

$C_{19}H_{17}N_7O_2$
Molecular Weight: 375.38397
ALogP: 4.197
Rotatable Bonds: 6
Acceptors: 7
Donors: 1

Model Prediction

Prediction: 0.177
Unit: g/kg_body_weight
Mahalanobis Distance: 10.5
Mahalanobis Distance p-value: 6.76e-006

Mahalanobis Distance: The Mahalanobis distance (MD) is a generalization of the Euclidean distance that accounts for correlations among the X properties. It is calculated as the distance to the center of the training data. The larger the MD, the less trustworthy the prediction.
Mahalanobis Distance p-value: The p-value gives the fraction of training data with an MD greater than or equal to the one for the given sample, assuming normally distributed data. The smaller the p-value, the less trustworthy the prediciton. For highly non-normal X properties (e.g., fingerprints), the MD p-value is wildly inaccurate.

Structural Similar Compounds

Name	C.I.PIGMENT RED 3	SALICYLAZOSULFAPYRIDINE	C.I.PIGMENT RED 23
Structure	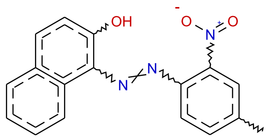	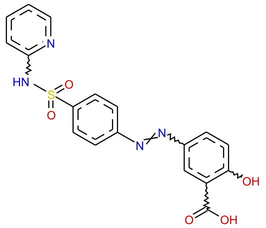	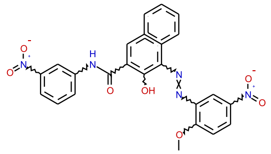
Actual Endpoint (-log C)	2.65635	3.375	2.30052
Predicted Endpoint (-log C)	2.97957	2.80292	3.55333
Distance	0.771	0.840	0.905
Reference	NCI/NTP TR-407	NCI/NTP TR-457	NCI/NTP TR-411

Model Applicability

Unknown features are fingerprint features in the query molecule, but not found or appearing too infrequently in the training set.

1. All properties and OPS components are within expected ranges.
2. Unknown FCFP_2 feature: 8: [*][N+](=[*])[*]
3. Unknown FCFP_2 feature: 5: [*][O-]
4. Unknown FCFP_2 feature: -332197802: [*][c]1:[*]:[*]:[c](:[*]):n:1:n:[*]
5. Unknown FCFP_2 feature: 1294285001: [*]=NN[c](:[*]):[*]
6. Unknown FCFP_2 feature: -828984032: [*][N+](=[*])[c](:[cH]:[*]):[cH]:[*]
7. Unknown FCFP_2 feature: -1338588315: [*]:[c](:[*])[N+](=O)[O-]
8. Unknown FCFP_2 feature: 1872392852: [*][N+](=O)[*]
9. Unknown FCFP_2 feature: 260476081: [*][N+](=[*])[O-]

Feature Contribution

Top features for positive contribution

Fingerprint	Bit/Smiles	Feature Structure	Score

FCFP_2	3	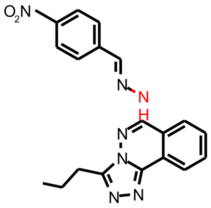<br>[*]N[*]	0.0737
FCFP_2	17	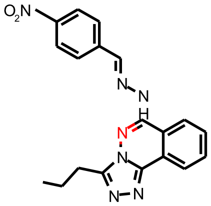<br>[*]:n:[*]	0.0441
**Top Features for negative contribution**			
**Fingerprint**	**Bit/Smiles**	**Feature Structure**	**Score**
FCFP_2	-1272798659	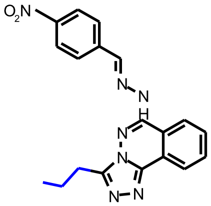<br>[*]CCC	-0.111
FCFP_2	203677720	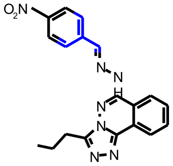<br>[*]=C[c](:[cH]:[*]):[cH]:[*]	-0.0829
FCFP_2	1	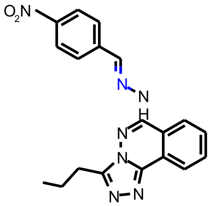<br>[*]=O	-0.0796

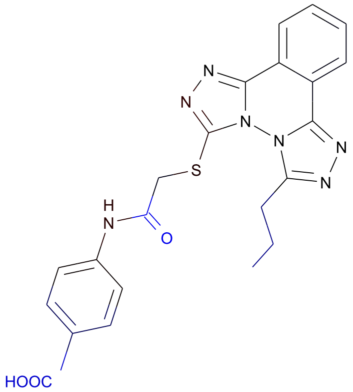

$C_{22}H_{19}N_7O_3S$
Molecular Weight: 461.49635
ALogP: 3.42
Rotatable Bonds: 7
Acceptors: 8
Donors: 2

Model Prediction

Prediction: 0.429
Unit: g/kg_body_weight
Mahalanobis Distance: 13.2
Mahalanobis Distance p-value: 5.35e-011

Mahalanobis Distance: The Mahalanobis distance (MD) is a generalization of the Euclidean distance that accounts for correlations among the X properties. It is calculated as the distance to the center of the training data. The larger the MD, the less trustworthy the prediction.
Mahalanobis Distance p-value: The p-value gives the fraction of training data with an MD greater than or equal to the one for the given sample, assuming normally distributed data. The smaller the p-value, the less trustworthy the prediciton. For highly non-normal X properties (e.g., fingerprints), the MD p-value is wildly inaccurate.

Structural Similar Compounds

Name	C.I.PIGMENT RED 23	SALICYLAZOSULFAPYRIDINE	RESERPINE
Structure	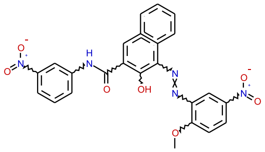	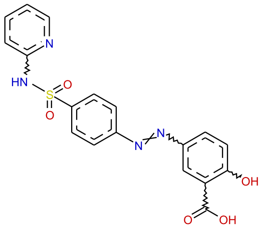	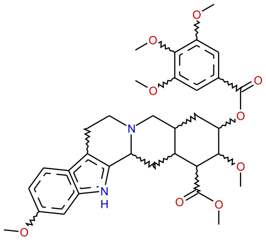
Actual Endpoint (-log C)	2.30052	3.375	6.13118
Predicted Endpoint (-log C)	3.55333	2.80292	4.38304
Distance	0.650	0.779	0.999
Reference	NCI/NTP TR-411	NCI/NTP TR-457	NCI/NTP TR-193

Model Applicability

Unknown features are fingerprint features in the query molecule, but not found or appearing too infrequently in the training set.

1. Num_AromaticRings out of range. Value: 5. Training min, max, mean, SD: 0, 4, 1.1685, 0.8469.
2. Unknown FCFP_2 feature: -1410079687: [*]S[c]1:n:[*]:[*]:n:1:[*]
3. Unknown FCFP_2 feature: -332197802: [*][c]1:[*]:[*]:[c](:[*]):n:1:n:[*]

Feature Contribution

Top features for positive contribution			
**Fingerprint**	**Bit/Smiles**	**Feature Structure**	**Score**
FCFP_2	1036089772	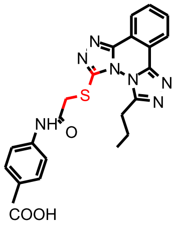 [*]CS[c](:[*]):[*]	0.0749

FCFP_2	3	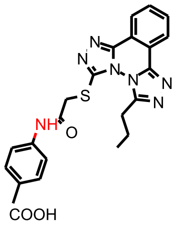 [*]N[*]	0.0737
FCFP_2	17	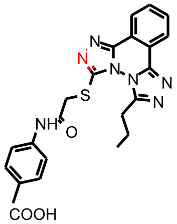 [*]:n:[*]	0.0441
**Top Features for negative contribution**			
**Fingerprint**	**Bit/Smiles**	**Feature Structure**	**Score**
FCFP_2	7	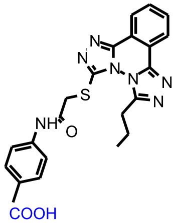 [*]O	-0.214
FCFP_2	-548632217	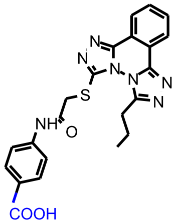 [*]C(=[*])O	-0.119
FCFP_2	-1272798659	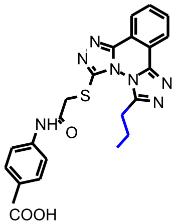 [*]CCC	-0.111

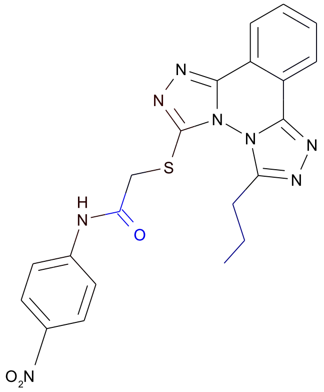

$C_{21}H_{18}N_8O_3S$
Molecular Weight: 462.48441
ALogP: 3.685
Rotatable Bonds: 7
Acceptors: 8
Donors: 1

Model Prediction

Prediction: 0.0899
Unit: g/kg_body_weight
Mahalanobis Distance: 13
Mahalanobis Distance p-value: 1.37e-010

Mahalanobis Distance: The Mahalanobis distance (MD) is a generalization of the Euclidean distance that accounts for correlations among the X properties. It is calculated as the distance to the center of the training data. The larger the MD, the less trustworthy the prediction.
Mahalanobis Distance p-value: The p-value gives the fraction of training data with an MD greater than or equal to the one for the given sample, assuming normally distributed data. The smaller the p-value, the less trustworthy the prediciton. For highly non-normal X properties (e.g., fingerprints), the MD p-value is wildly inaccurate.

Structural Similar Compounds

Name	C.I.PIGMENT RED 23	SALICYLAZOSULFAPYRIDINE	RESERPINE
Structure	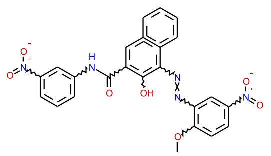	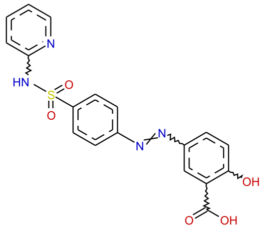	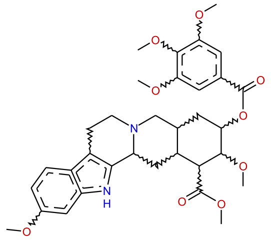
Actual Endpoint (-log C)	2.30052	3.375	6.13118
Predicted Endpoint (-log C)	3.55333	2.80292	4.38304
Distance	0.698	0.924	1.020
Reference	NCI/NTP TR-411	NCI/NTP TR-457	NCI/NTP TR-193

Model Applicability

Unknown features are fingerprint features in the query molecule, but not found or appearing too infrequently in the training set.

1. Num_AromaticRings out of range. Value: 5. Training min, max, mean, SD: 0, 4, 1.1685, 0.8469.
2. OPS PC9 out of range. Value: 4.4579. Training min, max, SD, explained variance: -2.8548, 3.3954, 1.263, 0.0360.
3. Unknown FCFP_2 feature: 8: [*][N+](=[*])[*]
4. Unknown FCFP_2 feature: 5: [*][O-]
5. Unknown FCFP_2 feature: -1410079687: [*]S[c]1:n:[*]:[*]:n:1:[*]
6. Unknown FCFP_2 feature: -332197802: [*][c]1:[*]:[*]:[c](:[*]):n:1:n:[*]
7. Unknown FCFP_2 feature: -828984032: [*][N+](=[*])[c](:[cH]:[*]):[cH]:[*]
8. Unknown FCFP_2 feature: -1338588315: [*]:[c](:[*])[N+](=O)[O-]
9. Unknown FCFP_2 feature: 1872392852: [*][N+](=O)[*]
10. Unknown FCFP_2 feature: 260476081: [*][N+](=[*])[O-]

Feature Contribution

Top features for positive contribution			
Fingerprint	Bit/Smiles	Feature Structure	Score

FCFP_2	1036089772	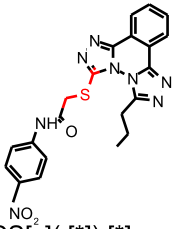 [*]CS[c](:[*]):[*]	0.0749
FCFP_2	3	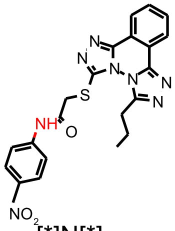 [*]N[*]	0.0737
FCFP_2	17	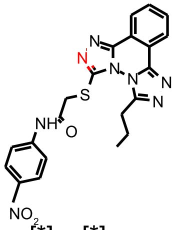 [*]:n:[*]	0.0441

Top Features for negative contribution

Fingerprint	Bit/Smiles	Feature Structure	Score
FCFP_2	-1272798659	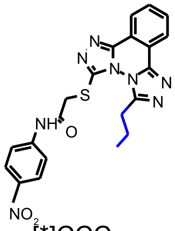 [*]CCC	-0.111
FCFP_2	1872154524	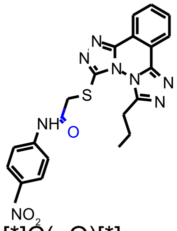 [*]C(=O)[*]	-0.105

FCFP_2	1	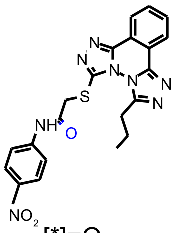<br>[*]=O	-0.0796

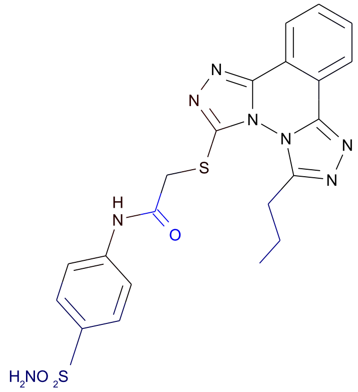

$C_{21}H_{20}N_8O_3S_2$
Molecular Weight: 496.56529
ALogP: 2.496
Rotatable Bonds: 7
Acceptors: 8
Donors: 2

Model Prediction

Prediction: 0.0954
Unit: g/kg_body_weight
Mahalanobis Distance: 14.1
Mahalanobis Distance p-value: 7.51e-013

Mahalanobis Distance: The Mahalanobis distance (MD) is a generalization of the Euclidean distance that accounts for correlations among the X properties. It is calculated as the distance to the center of the training data. The larger the MD, the less trustworthy the prediction.
Mahalanobis Distance p-value: The p-value gives the fraction of training data with an MD greater than or equal to the one for the given sample, assuming normally distributed data. The smaller the p-value, the less trustworthy the prediciton. For highly non-normal X properties (e.g., fingerprints), the MD p-value is wildly inaccurate.

Structural Similar Compounds

Name	C.I.PIGMENT RED 23	SALICYLAZOSULFAPYRIDINE	4,4'-DIAMINO-2,2'-STILBENEDISULFONIC ACID.2NaSALT
Structure	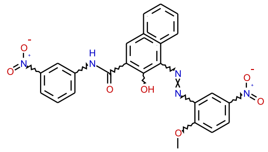	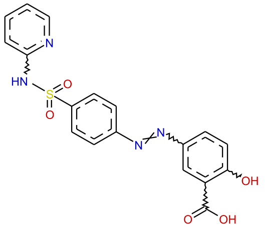	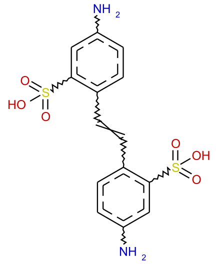
Actual Endpoint (-log C)	2.30052	3.375	2.50759
Predicted Endpoint (-log C)	3.55333	2.80292	3.26068
Distance	0.675	0.870	1.078
Reference	NCI/NTP TR-411	NCI/NTP TR-457	NCI/NTP TR-412

Model Applicability

Unknown features are fingerprint features in the query molecule, but not found or appearing too infrequently in the training set.

1. Num_AromaticRings out of range. Value: 5. Training min, max, mean, SD: 0, 4, 1.1685, 0.8469.
2. OPS PC9 out of range. Value: 4.4353. Training min, max, SD, explained variance: -2.8548, 3.3954, 1.263, 0.0360.
3. Unknown FCFP_2 feature: -1410079687: [*]S[c]1:n:[*]:[*]:n:1:[*]
4. Unknown FCFP_2 feature: -332197802: [*][c]1:[*]:[*]:[c](:[*]):n:1:n:[*]

Feature Contribution

Top features for positive contribution

Fingerprint	Bit/Smiles	Feature Structure	Score
FCFP_2	1036089772	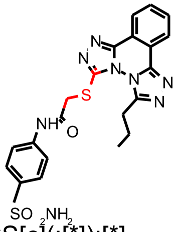 [*]CS[c](:[*]):[*]	0.0749

FCFP_2	3	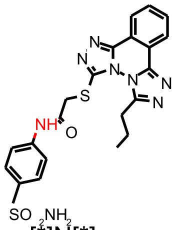 [*]N[*]	0.0737
FCFP_2	17	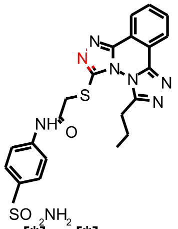 [*]:n:[*]	0.0441

Top Features for negative contribution

Fingerprint	Bit/Smiles	Feature Structure	Score
FCFP_2	-1272798659	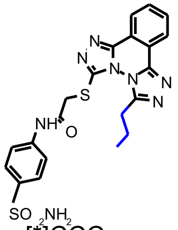 [*]CCC	-0.111
FCFP_2	1872154524	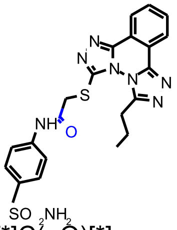 [*]C(=O)[*]	-0.105
FCFP_2	203677720	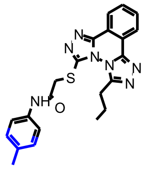 [*]=C[c](:[cH]:[*]):[cH]:[*]	-0.0829

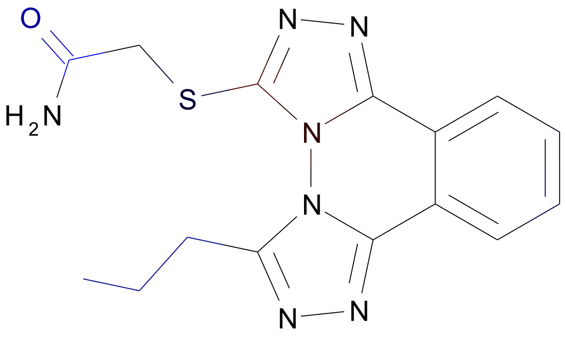

$C_{15}H_{15}N_7OS$
Molecular Weight: 341.39089
ALogP: 2.008
Rotatable Bonds: 5
Acceptors: 6
Donors: 1

Model Prediction

Prediction: 0.118
Unit: g/kg_body_weight
Mahalanobis Distance: 12.2
Mahalanobis Distance p-value: 4.36e-009

Mahalanobis Distance: The Mahalanobis distance (MD) is a generalization of the Euclidean distance that accounts for correlations among the X properties. It is calculated as the distance to the center of the training data. The larger the MD, the less trustworthy the prediction.
Mahalanobis Distance p-value: The p-value gives the fraction of training data with an MD greater than or equal to the one for the given sample, assuming normally distributed data. The smaller the p-value, the less trustworthy the prediciton. For highly non-normal X properties (e.g., fingerprints), the MD p-value is wildly inaccurate.

Structural Similar Compounds

Name	C.I.PIGMENT RED 3	SALICYLAZOSULFAPYRIDINE	TRIAMTERENE
Structure	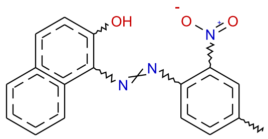	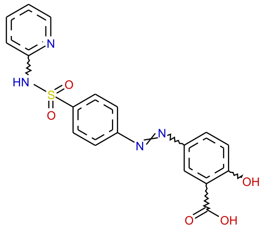	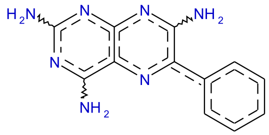
Actual Endpoint (-log C)	2.65635	3.375	4.00564
Predicted Endpoint (-log C)	2.97957	2.80292	3.1992
Distance	0.831	0.846	0.884
Reference	NCI/NTP TR-407	NCI/NTP TR-457	NCI/NTP TR-420

Model Applicability

Unknown features are fingerprint features in the query molecule, but not found or appearing too infrequently in the training set.

1. All properties and OPS components are within expected ranges.
2. Unknown FCFP_2 feature: -332197802: [*][c]1:[*]:[*]:[c](:[*]):n:1:n:[*]
3. Unknown FCFP_2 feature: -1410079687: [*]S[c]1:n:[*]:[*]:n:1:[*]

Feature Contribution

Top features for positive contribution

Fingerprint	Bit/Smiles	Feature Structure	Score
FCFP_2	1036089772	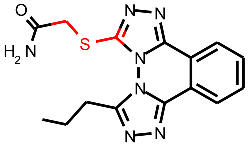 [*]CS[c](:[*]):[*]	0.0749

FCFP_2	3	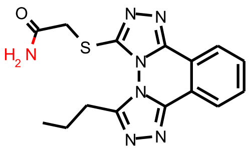 [*]N[*]	0.0737
FCFP_2	17	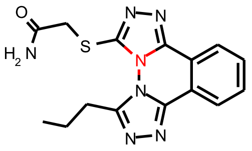 [*]:n:[*]	0.0441

Top Features for negative contribution

Fingerprint	Bit/Smiles	Feature Structure	Score
FCFP_2	-1272798659	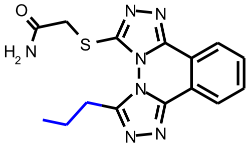 [*]CCC	-0.111
FCFP_2	1872154524	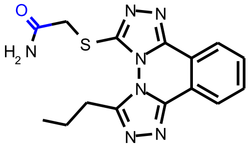 [*]C(=O)[*]	-0.105
FCFP_2	1	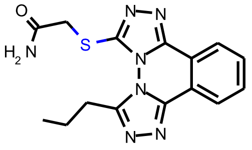 [*]=O	-0.0796

Doxorubicin

TOPKAT_Rat_Maximum_Tolerated_Dose_Feed

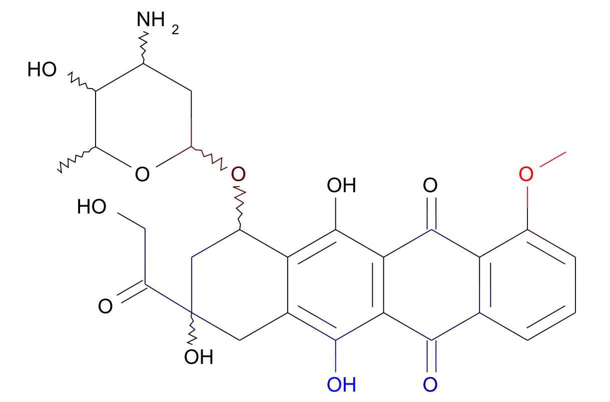

$C_{27}H_{29}NO_{11}$

Molecular Weight: 543.51925

ALogP: -4.4e-002

Rotatable Bonds: 5

Acceptors: 12

Donors: 6

Structural Similar Compounds

Name	OXYTETRACYCLINE	4,4'-DIAMINO-2,2'-STILBENEDISULFONIC ACID.2NaSALT	ERYTHROMYCIN
Structure	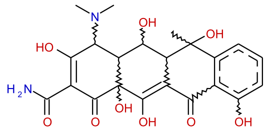	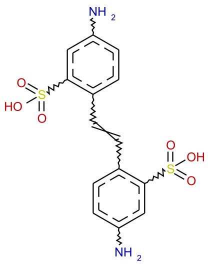	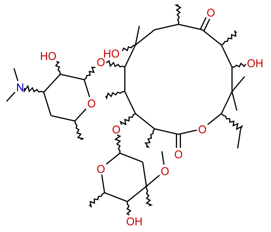
Actual Endpoint (-log C)	2.36214	2.50759	3.29629
Predicted Endpoint (-log C)	2.77834	3.26068	4.83895
Distance	0.706	0.916	1.034
Reference	NCI/NTP TR-315	NCI/NTP TR-412	NCI/NTP TR-338

Model Prediction

Prediction: 0.277

Unit: g/kg_body_weight

Mahalanobis Distance: 11.1

Mahalanobis Distance p-value: 5.33e-007

Mahalanobis Distance: The Mahalanobis distance (MD) is a generalization of the Euclidean distance that accounts for correlations among the X properties. It is calculated as the distance to the center of the training data. The larger the MD, the less trustworthy the prediction.
Mahalanobis Distance p-value: The p-value gives the fraction of training data with an MD greater than or equal to the one for the given sample, assuming normally distributed data. The smaller the p-value, the less trustworthy the prediciton. For highly non-normal X properties (e.g., fingerprints), the MD p-value is wildly inaccurate.

Model Applicability

Unknown features are fingerprint features in the query molecule, but not found or appearing too infrequently in the training set.

1. Molecular_PolarSurfaceArea out of range. Value: 206.07. Training min, max, mean, SD: 0, 201.84, 63.052, 40.7.
2. Unknown FCFP_2 feature: 1186333723: [*]CC(O[*])[c](:[*]):[*]

Feature Contribution

Top features for positive contribution			
Fingerprint	Bit/Smiles	Feature Structure	Score
FCFP_2	136627117	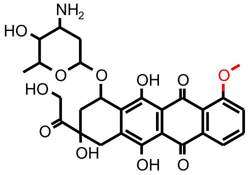 [*]OC	0.173

FCFP_2	-1143715940	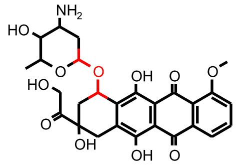 [*]C([*])OC([*])[*]	0.095
FCFP_2	1036089772	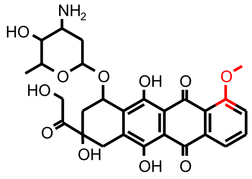 [*]CS[c](:[*]):[*]	0.0749
**Top Features for negative contribution**			
**Fingerprint**	**Bit/Smiles**	**Feature Structure**	**Score**
FCFP_2	7	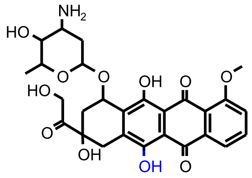 [*]O	-0.214
FCFP_2	-549108873	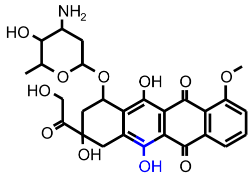 [*]:[c](:[*])O	-0.127
FCFP_2	-1272798659	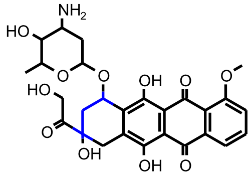 [*]CCC	-0.111

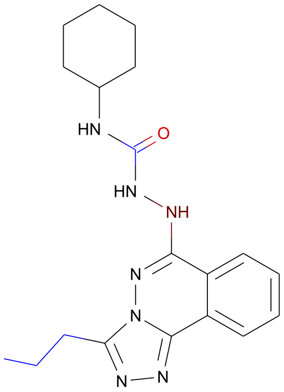

$C_{19}H_{25}N_7O$

Molecular Weight: 367.44809

ALogP: 3.681

Rotatable Bonds: 5

Acceptors: 5

Donors: 3

Model Prediction

Prediction: 0.00961

Unit: g/kg_body_weight

Mahalanobis Distance: 14

Mahalanobis Distance p-value: 1.21e-011

Mahalanobis Distance: The Mahalanobis distance (MD) is a generalization of the Euclidean distance that accounts for correlations among the X properties. It is calculated as the distance to the center of the training data. The larger the MD, the less trustworthy the prediction.
Mahalanobis Distance p-value: The p-value gives the fraction of training data with an MD greater than or equal to the one for the given sample, assuming normally distributed data. The smaller the p-value, the less trustworthy the prediciton. For highly non-normal X properties (e.g., fingerprints), the MD p-value is wildly inaccurate.

Structural Similar Compounds

Name	OCHRATOXIN	SULFISOOXAZOLE	PENICILLIN VK
Structure	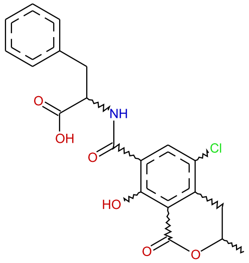	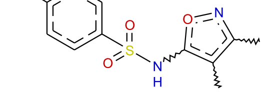	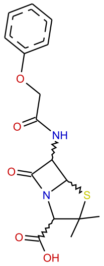
Actual Endpoint (-log C)	6.28396	2.82494	2.54455
Predicted Endpoint (-log C)	5.12358	3.0705	3.9702
Distance	0.706	0.886	1.038
Reference	NCI/NTP TR-358	NCI/NTP TR-138	NCI/NTP TR-336

Model Applicability

Unknown features are fingerprint features in the query molecule, but not found or appearing too infrequently in the training set.

1. Num_AromaticRings out of range. Value: 3. Training min, max, mean, SD: 0, 2, 0.5625, 0.693.
2. OPS PC9 out of range. Value: -2.7104. Training min, max, SD, explained variance: -2.7086, 2.9267, 1.019, 0.0321.
3. Unknown FCFP_2 feature: -332197802: [*][c]1:[*]:[*]:[c](:[*]):n:1:n:[*]
4. Unknown FCFP_2 feature: -1564473960: [*]:n1:[*]:[*]:n:[c]:1:[c](:[*]):[*]
5. Unknown FCFP_2 feature: 1294344583: [*]NN[c](:[*]):[*]
6. Unknown FCFP_2 feature: -1539162406: [*]C[c]1:n:[*]:[*]:n:1:[*]
7. Unknown FCFP_2 feature: -885461129: [*]NNC(=[*])[*]
8. Unknown FCFP_2 feature: 1499521844: [*]NC(=O)N[*]

Feature Contribution

Top features for positive contribution

Fingerprint	Bit/Smiles	Feature Structure	Score

FCFP_2	1	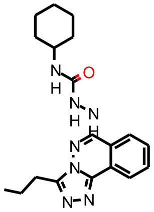 [*]=O	0.511
FCFP_2	3	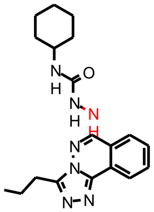 [*]N[*]	0.104
FCFP_2	-1272798659	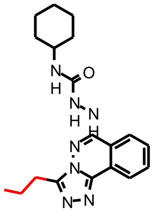 [*]CCC	0.0703
**Top Features for negative contribution**			
**Fingerprint**	**Bit/Smiles**	**Feature Structure**	**Score**
FCFP_2	136597326	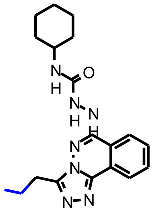 [*]CC	-0.489
FCFP_2	1872154524	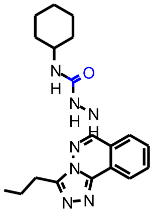 [*]C(=O)[*]	-0.307

FCFP_2	0	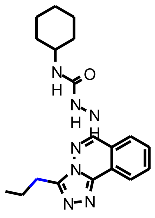 [*]C[*]	-0.29

7b TOPKAT_Rat_Maximum_Tolerated_Dose_Gavage

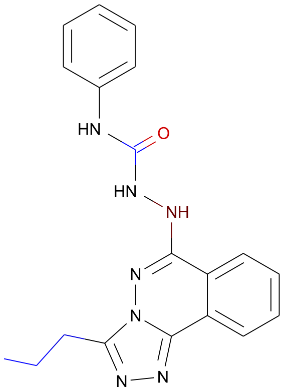

$C_{19}H_{19}N_7O$
Molecular Weight: 361.40046
ALogP: 3.399
Rotatable Bonds: 5
Acceptors: 5
Donors: 3

Model Prediction

Prediction: 0.0102
Unit: g/kg_body_weight
Mahalanobis Distance: 16.9
Mahalanobis Distance p-value: 1.3e-015

Mahalanobis Distance: The Mahalanobis distance (MD) is a generalization of the Euclidean distance that accounts for correlations among the X properties. It is calculated as the distance to the center of the training data. The larger the MD, the less trustworthy the prediction.
Mahalanobis Distance p-value: The p-value gives the fraction of training data with an MD greater than or equal to the one for the given sample, assuming normally distributed data. The smaller the p-value, the less trustworthy the prediciton. For highly non-normal X properties (e.g., fingerprints), the MD p-value is wildly inaccurate.

Structural Similar Compounds

Name	OCHRATOXIN	SULFISOOXAZOLE	PENICILLIN VK
Structure	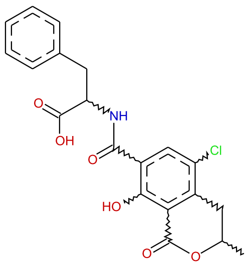	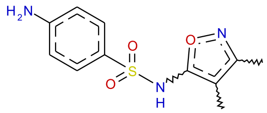	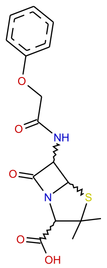
Actual Endpoint (-log C)	6.28396	2.82494	2.54455
Predicted Endpoint (-log C)	5.12358	3.0705	3.9702
Distance	0.957	0.977	1.275
Reference	NCI/NTP TR-358	NCI/NTP TR-138	NCI/NTP TR-336

Model Applicability

Unknown features are fingerprint features in the query molecule, but not found or appearing too infrequently in the training set.

1. Num_AromaticRings out of range. Value: 4. Training min, max, mean, SD: 0, 2, 0.5625, 0.693.
2. OPS PC9 out of range. Value: -2.7992. Training min, max, SD, explained variance: -2.7086, 2.9267, 1.019, 0.0321.
3. Unknown FCFP_2 feature: -332197802: [*][c]1:[*]:[*]:[c](:[*]):n:1:n:[*]
4. Unknown FCFP_2 feature: -1564473960: [*]:n1:[*]:[*]:n:[c]:1:[c](:[*]):[*]
5. Unknown FCFP_2 feature: 1294344583: [*]NN[c](:[*]):[*]
6. Unknown FCFP_2 feature: -1539162406: [*]C[c]1:n:[*]:[*]:n:1:[*]
7. Unknown FCFP_2 feature: -885461129: [*]NNC(=[*])[*]
8. Unknown FCFP_2 feature: 1499521844: [*]NC(=O)N[*]

Feature Contribution

Top features for positive contribution

Fingerprint	Bit/Smiles	Feature Structure	Score

FCFP_2	1	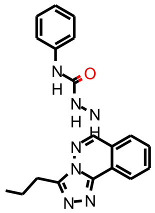 [*]=O	0.511
FCFP_2	3	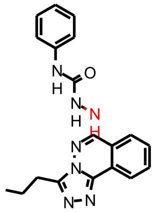 [*]N[*]	0.104
FCFP_2	-1272798659	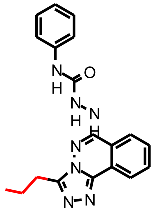 [*]CCC	0.0703
**Top Features for negative contribution**			
**Fingerprint**	**Bit/Smiles**	**Feature Structure**	**Score**
FCFP_2	136597326	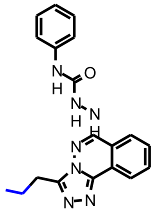 [*]CC	-0.489
FCFP_2	1872154524	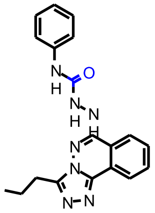 [*]C(=O)[*]	-0.307

FCFP_2	0	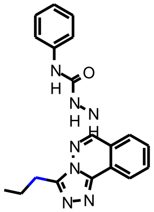<br>[*]C[*]	-0.29

TOPKAT_Rat_Maximum_Tolerated_Dose_Gavage

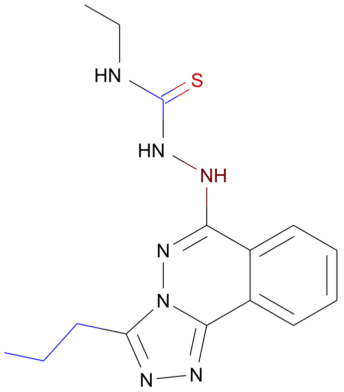

$C_{15}H_{19}N_7S$

Molecular Weight: 329.42325

ALogP: 3.388

Rotatable Bonds: 7

Acceptors: 5

Donors: 3

Model Prediction

Prediction: 0.0421

Unit: g/kg_body_weight

Mahalanobis Distance: 12

Mahalanobis Distance p-value: 8.55e-009

Mahalanobis Distance: The Mahalanobis distance (MD) is a generalization of the Euclidean distance that accounts for correlations among the X properties. It is calculated as the distance to the center of the training data. The larger the MD, the less trustworthy the prediction.
Mahalanobis Distance p-value: The p-value gives the fraction of training data with an MD greater than or equal to the one for the given sample, assuming normally distributed data. The smaller the p-value, the less trustworthy the prediciton. For highly non-normal X properties (e.g., fingerprints), the MD p-value is wildly inaccurate.

Structural Similar Compounds

Name	OCHRATOXIN	SULFISOOXAZOLE	PENICILLIN VK
Structure	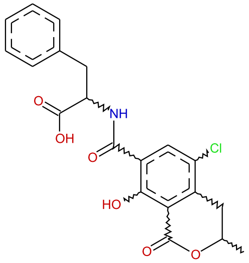	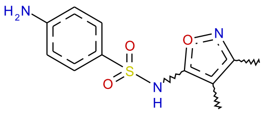	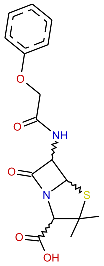
Actual Endpoint (-log C)	6.28396	2.82494	2.54455
Predicted Endpoint (-log C)	5.12358	3.0705	3.9702
Distance	0.721	0.867	1.029
Reference	NCI/NTP TR-358	NCI/NTP TR-138	NCI/NTP TR-336

Model Applicability

Unknown features are fingerprint features in the query molecule, but not found or appearing too infrequently in the training set.

1. Num_AromaticRings out of range. Value: 3. Training min, max, mean, SD: 0, 2, 0.5625, 0.693.
2. OPS PC9 out of range. Value: -2.8136. Training min, max, SD, explained variance: -2.7086, 2.9267, 1.019, 0.0321.
3. Unknown FCFP_2 feature: -332197802: [*][c]1:[*]:[*]:[c](:[*]):n:1:n:[*]
4. Unknown FCFP_2 feature: -1564473960: [*]:n1:[*]:[*]:n:[c]:1:[c](:[*]):[*]
5. Unknown FCFP_2 feature: 1294344583: [*]NN[c](:[*]):[*]
6. Unknown FCFP_2 feature: -1539162406: [*]C[c]1:n:[*]:[*]:n:1:[*]
7. Unknown FCFP_2 feature: -885461129: [*]NNC(=[*])[*]
8. Unknown FCFP_2 feature: 1499521844: [*]NC(=O)N[*]

Feature Contribution

Top features for positive contribution

Fingerprint	Bit/Smiles	Feature Structure	Score

FCFP_2	1	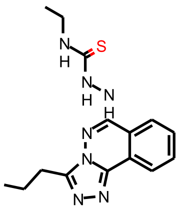 [*]=O	0.511
FCFP_2	3	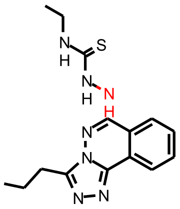 [*]N[*]	0.104
FCFP_2	-1272798659	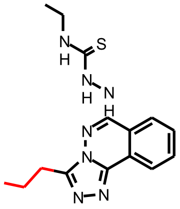 [*]CCC	0.0703

Top Features for negative contribution

**Fingerprint**	**Bit/Smiles**	**Feature Structure**	**Score**
FCFP_2	136597326	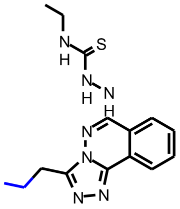 [*]CC	-0.489
FCFP_2	1872154524	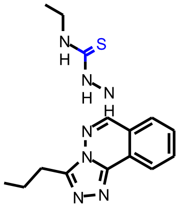 [*]C(=O)[*]	-0.307

FCFP_2	0	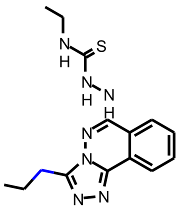 [*]C[*]	-0.29

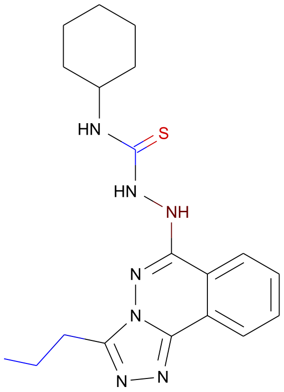

$C_{19}H_{25}N_7S$
Molecular Weight: 383.51369
ALogP: 4.898
Rotatable Bonds: 7
Acceptors: 5
Donors: 3

Model Prediction

Prediction: 0.0189
Unit: g/kg_body_weight
Mahalanobis Distance: 12.3
Mahalanobis Distance p-value: 3.65e-009

Mahalanobis Distance: The Mahalanobis distance (MD) is a generalization of the Euclidean distance that accounts for correlations among the X properties. It is calculated as the distance to the center of the training data. The larger the MD, the less trustworthy the prediction.
Mahalanobis Distance p-value: The p-value gives the fraction of training data with an MD greater than or equal to the one for the given sample, assuming normally distributed data. The smaller the p-value, the less trustworthy the prediciton. For highly non-normal X properties (e.g., fingerprints), the MD p-value is wildly inaccurate.

Structural Similar Compounds

Name	OCHRATOXIN	SULFISOOXAZOLE	PENICILLIN VK
Structure	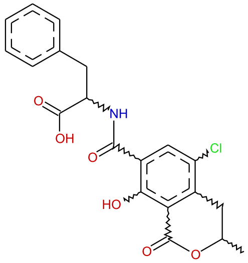	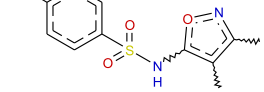	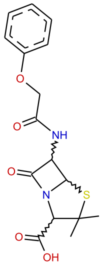
Actual Endpoint (-log C)	6.28396	2.82494	2.54455
Predicted Endpoint (-log C)	5.12358	3.0705	3.9702
Distance	0.713	0.999	1.097
Reference	NCI/NTP TR-358	NCI/NTP TR-138	NCI/NTP TR-336

Model Applicability

Unknown features are fingerprint features in the query molecule, but not found or appearing too infrequently in the training set.

1. Num_AromaticRings out of range. Value: 3. Training min, max, mean, SD: 0, 2, 0.5625, 0.693.
2. OPS PC9 out of range. Value: -2.7796. Training min, max, SD, explained variance: -2.7086, 2.9267, 1.019, 0.0321.
3. Unknown FCFP_2 feature: -332197802: [*][c]1:[*]:[*]:[c](:[*]):n:1:n:[*]
4. Unknown FCFP_2 feature: -1564473960: [*]:n1:[*]:[*]:n:[c]:1:[c](:[*]):[*]
5. Unknown FCFP_2 feature: 1294344583: [*]NN[c](:[*]):[*]
6. Unknown FCFP_2 feature: -1539162406: [*]C[c]1:n:[*]:[*]:n:1:[*]
7. Unknown FCFP_2 feature: -885461129: [*]NNC(=[*])[*]
8. Unknown FCFP_2 feature: 1499521844: [*]NC(=O)N[*]

Feature Contribution

Top features for positive contribution

Fingerprint	Bit/Smiles	Feature Structure	Score

FCFP_2	1	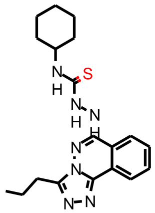<br>[*]=O	0.511
FCFP_2	3	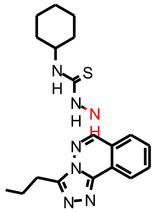<br>[*]N[*]	0.104
FCFP_2	-1272798659	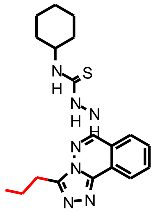<br>[*]CCC	0.0703

Top Features for negative contribution

Fingerprint	Bit/Smiles	Feature Structure	Score
FCFP_2	136597326	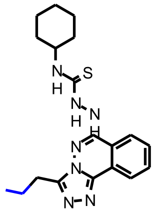<br>[*]CC	-0.489
FCFP_2	1872154524	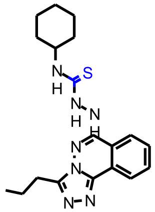<br>[*]C(=O)[*]	-0.307

FCFP_2	0	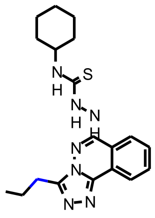 [*]C[*]	-0.29

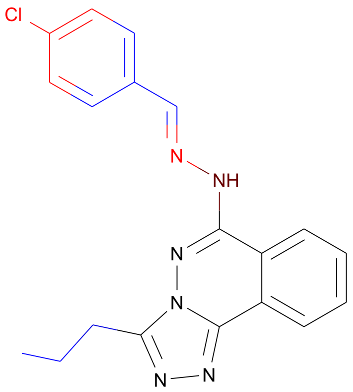

$C_{19}H_{17}ClN_6$

Molecular Weight: 364.83147

ALogP: 4.967

Rotatable Bonds: 5

Acceptors: 5

Donors: 1

Model Prediction

Prediction: 0.000411

Unit: g/kg_body_weight

Mahalanobis Distance: 16

Mahalanobis Distance p-value: 2.06e-014

Mahalanobis Distance: The Mahalanobis distance (MD) is a generalization of the Euclidean distance that accounts for correlations among the X properties. It is calculated as the distance to the center of the training data. The larger the MD, the less trustworthy the prediction.
Mahalanobis Distance p-value: The p-value gives the fraction of training data with an MD greater than or equal to the one for the given sample, assuming normally distributed data. The smaller the p-value, the less trustworthy the prediciton. For highly non-normal X properties (e.g., fingerprints), the MD p-value is wildly inaccurate.

Structural Similar Compounds

Name	PHENYLBUTAZONE	PROMETHAZINE.HCL	CHLORPHENIRAMINE MALEATE
Structure	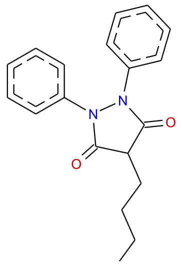	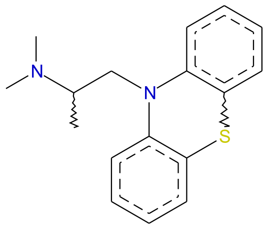	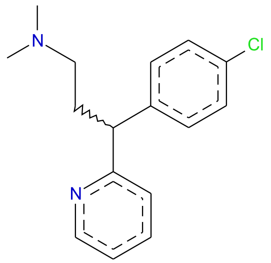
Actual Endpoint (-log C)	3.48909	3.93152	3.96188
Predicted Endpoint (-log C)	3.17333	4.72433	3.83117
Distance	1.074	1.102	1.116
Reference	NCI/NTP TR-367	NCI/NTP TR-425	NCI/NTP TR-317

Model Applicability

Unknown features are fingerprint features in the query molecule, but not found or appearing too infrequently in the training set.

1. Num_AromaticRings out of range. Value: 4. Training min, max, mean, SD: 0, 2, 0.5625, 0.693.
2. OPS PC7 out of range. Value: -3.0704. Training min, max, SD, explained variance: -2.8003, 2.9332, 1.16, 0.0416.
3. Unknown FCFP_2 feature: -332197802: [*][c]1:[*]:[*]:[c](:[*]):n:1:n:[*]
4. Unknown FCFP_2 feature: -1564473960: [*]:n1:[*]:[*]:n:[c]:1:[c](:[*]):[*]
5. Unknown FCFP_2 feature: -1539162406: [*]C[c]1:n:[*]:[*]:n:1:[*]
6. Unknown FCFP_2 feature: 1294285001: [*]=NN[c](:[*]):[*]
7. Unknown FCFP_2 feature: 581019816: [*]N\N=C\[*]

Feature Contribution

Top features for positive contribution			
Fingerprint	Bit/Smiles	Feature Structure	Score

FCFP_2	32	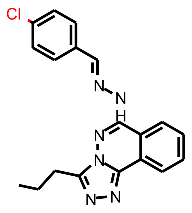 [*]Cl	0.526
FCFP_2	1	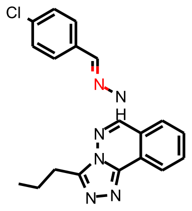 [*]=O	0.511
FCFP_2	367998008	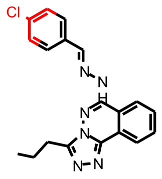 [*]:[cH]:[c](Cl):[cH]:[*]	0.413
**Top Features for negative contribution**			
**Fingerprint**	**Bit/Smiles**	**Feature Structure**	**Score**
FCFP_2	136597326	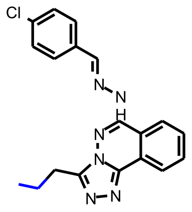 [*]CC	-0.489
FCFP_2	203677720	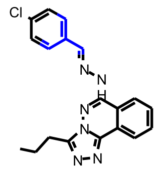 [*]=C[c](:[cH]:[*]):[cH]:[*]	-0.406

FCFP_2	0	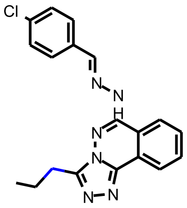 [*]C[*]	-0.29

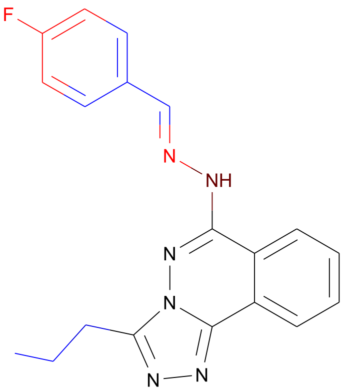

$C_{19}H_{17}FN_6$
Molecular Weight: 348.37688
ALogP: 4.508
Rotatable Bonds: 5
Acceptors: 5
Donors: 1

Model Prediction

Prediction: 0.000525
Unit: g/kg_body_weight
Mahalanobis Distance: 16.1
Mahalanobis Distance p-value: 1.64e-014

Mahalanobis Distance: The Mahalanobis distance (MD) is a generalization of the Euclidean distance that accounts for correlations among the X properties. It is calculated as the distance to the center of the training data. The larger the MD, the less trustworthy the prediction.
Mahalanobis Distance p-value: The p-value gives the fraction of training data with an MD greater than or equal to the one for the given sample, assuming normally distributed data. The smaller the p-value, the less trustworthy the prediciton. For highly non-normal X properties (e.g., fingerprints), the MD p-value is wildly inaccurate.

Structural Similar Compounds

Name	PHENYLBUTAZONE	PROMETHAZINE.HCL	CHLORPHENIRAMINE MALEATE
Structure	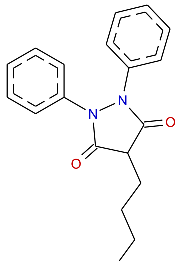	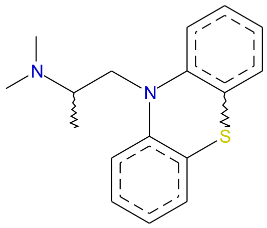	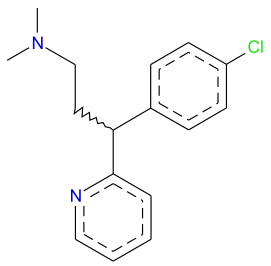
Actual Endpoint (-log C)	3.48909	3.93152	3.96188
Predicted Endpoint (-log C)	3.17333	4.72433	3.83117
Distance	1.062	1.092	1.101
Reference	NCI/NTP TR-367	NCI/NTP TR-425	NCI/NTP TR-317

Model Applicability

Unknown features are fingerprint features in the query molecule, but not found or appearing too infrequently in the training set.

1. Num_AromaticRings out of range. Value: 4. Training min, max, mean, SD: 0, 2, 0.5625, 0.693.
2. OPS PC7 out of range. Value: -3.0294. Training min, max, SD, explained variance: -2.8003, 2.9332, 1.16, 0.0416.
3. Unknown FCFP_2 feature: -332197802: [*][c]1:[*]:[*]:[c](:[*]):n:1:n:[*]
4. Unknown FCFP_2 feature: -1564473960: [*]:n1:[*]:[*]:n:[c]:1:[c](:[*]):[*]
5. Unknown FCFP_2 feature: -1539162406: [*]C[c]1:n:[*]:[*]:n:1:[*]
6. Unknown FCFP_2 feature: 1294285001: [*]=NN[c](:[*]):[*]
7. Unknown FCFP_2 feature: 581019816: [*]N\N=C\[*]

Feature Contribution

Top features for positive contribution

Fingerprint	Bit/Smiles	Feature Structure	Score

FCFP_2	32	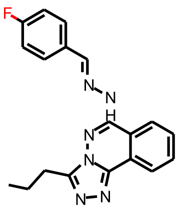 [*]Cl	0.526
FCFP_2	1	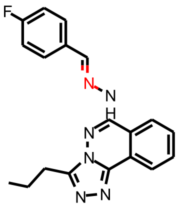 [*]=O	0.511
FCFP_2	367998008	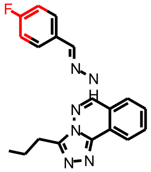 [*]:[cH]:[c](Cl):[cH]:[*]	0.413
**Top Features for negative contribution**			
**Fingerprint**	**Bit/Smiles**	**Feature Structure**	**Score**
FCFP_2	136597326	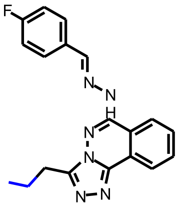 [*]CC	-0.489
FCFP_2	203677720	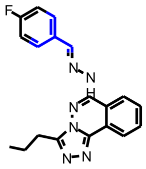 [*]=C[c](:[cH]:[*]):[cH]:[*]	-0.406

FCFP_2	0	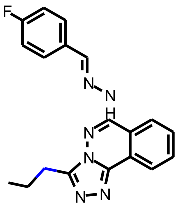 [*]C[*]	-0.29

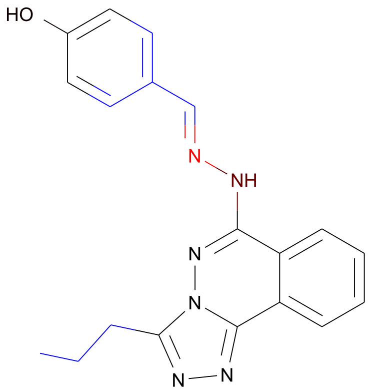

$C_{19}H_{18}N_6O$
Molecular Weight: 346.38582
ALogP: 4.061
Rotatable Bonds: 5
Acceptors: 6
Donors: 2

Model Prediction

Prediction: 0.00197
Unit: g/kg_body_weight
Mahalanobis Distance: 19.1
Mahalanobis Distance p-value: 2.18e-018

Mahalanobis Distance: The Mahalanobis distance (MD) is a generalization of the Euclidean distance that accounts for correlations among the X properties. It is calculated as the distance to the center of the training data. The larger the MD, the less trustworthy the prediction.
Mahalanobis Distance p-value: The p-value gives the fraction of training data with an MD greater than or equal to the one for the given sample, assuming normally distributed data. The smaller the p-value, the less trustworthy the prediciton. For highly non-normal X properties (e.g., fingerprints), the MD p-value is wildly inaccurate.

Structural Similar Compounds

Name	OCHRATOXIN	SULFISOOXAZOLE	PROBENECID
Structure	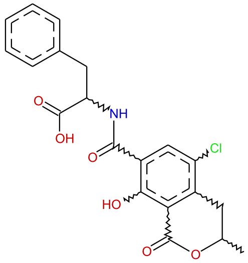	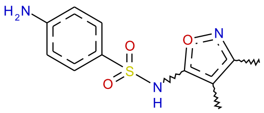	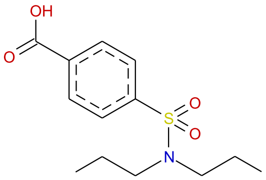
Actual Endpoint (-log C)	6.28396	2.82494	2.85333
Predicted Endpoint (-log C)	5.12358	3.0705	2.4258
Distance	0.968	1.064	1.232
Reference	NCI/NTP TR-358	NCI/NTP TR-138	NCI/NTP TR-395

Model Applicability

Unknown features are fingerprint features in the query molecule, but not found or appearing too infrequently in the training set.

1. Num_AromaticRings out of range. Value: 4. Training min, max, mean, SD: 0, 2, 0.5625, 0.693.
2. Unknown FCFP_2 feature: -332197802: [*][c]1:[*]:[*]:[c](:[*]):n:1:n:[*]
3. Unknown FCFP_2 feature: -1564473960: [*]:n1:[*]:[*]:n:[c]:1:[c](:[*]):[*]
4. Unknown FCFP_2 feature: -1539162406: [*]C[c]1:n:[*]:[*]:n:1:[*]
5. Unknown FCFP_2 feature: 1294285001: [*]=NN[c](:[*]):[*]
6. Unknown FCFP_2 feature: 581019816: [*]N\N=C\[*]

Feature Contribution

Top features for positive contribution

Fingerprint	Bit/Smiles	Feature Structure	Score
FCFP_2	1	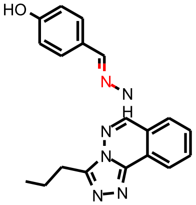 [*]=O	0.511

FCFP_2	3	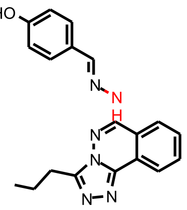 [*]N[*]	0.104
FCFP_2	-1272798659	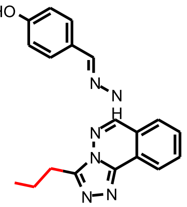 [*]CCC	0.0703
**Top Features for negative contribution**			
**Fingerprint**	**Bit/Smiles**	**Feature Structure**	**Score**
FCFP_2	136597326	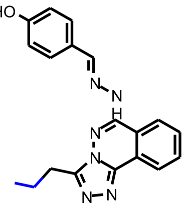 [*]CC	-0.489
FCFP_2	203677720	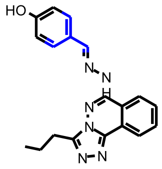 [*]=C[c](:[cH]:[*]):[cH]:[*]	-0.406
FCFP_2	0	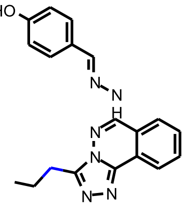 [*]C[*]	-0.29

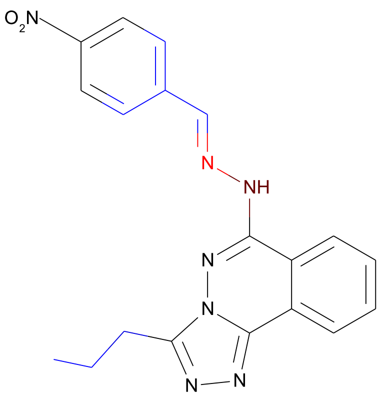

$C_{19}H_{17}N_7O_2$

Molecular Weight: 375.38397

ALogP: 4.197

Rotatable Bonds: 6

Acceptors: 7

Donors: 1

Model Prediction

Prediction: 0.00156

Unit: g/kg_body_weight

Mahalanobis Distance: 17.6

Mahalanobis Distance p-value: 1.56e-016

Mahalanobis Distance: The Mahalanobis distance (MD) is a generalization of the Euclidean distance that accounts for correlations among the X properties. It is calculated as the distance to the center of the training data. The larger the MD, the less trustworthy the prediction.
Mahalanobis Distance p-value: The p-value gives the fraction of training data with an MD greater than or equal to the one for the given sample, assuming normally distributed data. The smaller the p-value, the less trustworthy the prediciton. For highly non-normal X properties (e.g., fingerprints), the MD p-value is wildly inaccurate.

Structural Similar Compounds

Name	OCHRATOXIN	SULFISOOXAZOLE	PROBENECID
Structure	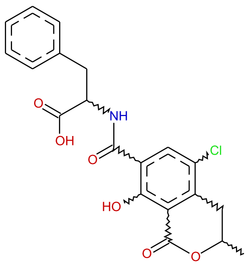	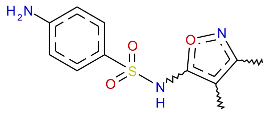	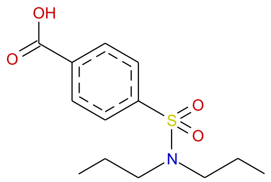
Actual Endpoint (-log C)	6.28396	2.82494	2.85333
Predicted Endpoint (-log C)	5.12358	3.0705	2.4258
Distance	1.096	1.156	1.294
Reference	NCI/NTP TR-358	NCI/NTP TR-138	NCI/NTP TR-395

Model Applicability

Unknown features are fingerprint features in the query molecule, but not found or appearing too infrequently in the training set.

1. Num_H_Acceptors out of range. Value: 7. Training min, max, mean, SD: 0, 6, 1.6146, 1.644.
2. Num_AromaticRings out of range. Value: 4. Training min, max, mean, SD: 0, 2, 0.5625, 0.693.
3. OPS PC6 out of range. Value: -2.6439. Training min, max, SD, explained variance: -2.4321, 2.9885, 1.256, 0.0488.
4. Unknown FCFP_2 feature: 8: [*][N+](=[*])[*]
5. Unknown FCFP_2 feature: 5: [*][O-]
6. Unknown FCFP_2 feature: -332197802: [*][c]1:[*]:[*]:[c](:[*]):n:1:n:[*]
7. Unknown FCFP_2 feature: -1564473960: [*]:n1:[*]:[*]:n:[c]:1:[c](:[*]):[*]
8. Unknown FCFP_2 feature: -1539162406: [*]C[c]1:n:[*]:[*]:n:1:[*]
9. Unknown FCFP_2 feature: 1294285001: [*]=NN[c](:[*]):[*]
10. Unknown FCFP_2 feature: 581019816: [*]N\N=C\[*]
11. Unknown FCFP_2 feature: -828984032: [*][N+](=[*])[c](:[cH]:[*]):[cH]:[*]
12. Unknown FCFP_2 feature: -1338588315: [*]:[c](:[*])[N+](=O)[O-]
13. Unknown FCFP_2 feature: 1872392852: [*][N+](=O)[*]
14. Unknown FCFP_2 feature: 260476081: [*][N+](=[*])[O-]

Feature Contribution

Top features for positive contribution			
Fingerprint	Bit/Smiles	Feature Structure	Score

FCFP_2	1	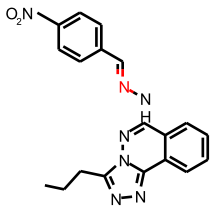 [*]=O	0.511
FCFP_2	3	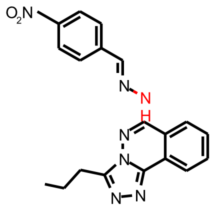 [*]N[*]	0.104
FCFP_2	-1272798659	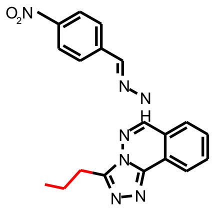 [*]CCC	0.0703
**Top Features for negative contribution**			
**Fingerprint**	**Bit/Smiles**	**Feature Structure**	**Score**
FCFP_2	136597326	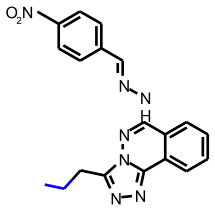 [*]CC	-0.489
FCFP_2	203677720	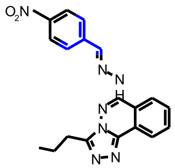 [*]=C[c](:[cH]:[*]):[cH]:[*]	-0.406

FCFP_2	0	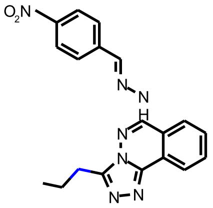<br>[*]C[*]	-0.29

TOPKAT_Rat_Maximum_Tolerated_Dose_Gavage

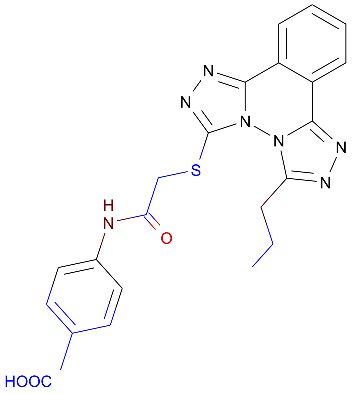

$C_{22}H_{19}N_7O_3S$

Molecular Weight: 461.49635

ALogP: 3.42

Rotatable Bonds: 7

Acceptors: 8

Donors: 2

Model Prediction

Prediction: 0.00596

Unit: g/kg_body_weight

Mahalanobis Distance: 18.3

Mahalanobis Distance p-value: 2.45e-017

Mahalanobis Distance: The Mahalanobis distance (MD) is a generalization of the Euclidean distance that accounts for correlations among the X properties. It is calculated as the distance to the center of the training data. The larger the MD, the less trustworthy the prediction.
Mahalanobis Distance p-value: The p-value gives the fraction of training data with an MD greater than or equal to the one for the given sample, assuming normally distributed data. The smaller the p-value, the less trustworthy the prediciton. For highly non-normal X properties (e.g., fingerprints), the MD p-value is wildly inaccurate.

Structural Similar Compounds

Name	OCHRATOXIN	SULFISOOXAZOLE	PENICILLIN VK
Structure	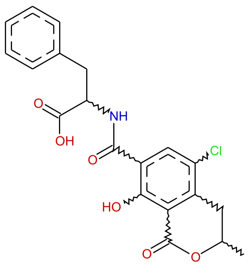	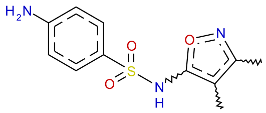	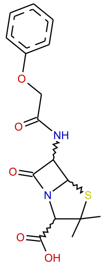
Actual Endpoint (-log C)	6.28396	2.82494	2.54455
Predicted Endpoint (-log C)	5.12358	3.0705	3.9702
Distance	1.228	1.392	1.462
Reference	NCI/NTP TR-358	NCI/NTP TR-138	NCI/NTP TR-336

Model Applicability

Unknown features are fingerprint features in the query molecule, but not found or appearing too infrequently in the training set.

1. Molecular_Weight out of range. Value: 461.5. Training min, max, mean, SD: 68.074, 434.63, 171.13, 85.06.
2. Num_H_Acceptors out of range. Value: 8. Training min, max, mean, SD: 0, 6, 1.6146, 1.644.
3. Num_AromaticRings out of range. Value: 5. Training min, max, mean, SD: 0, 2, 0.5625, 0.693.
4. Molecular_PolarSurfaceArea out of range. Value: 152.07. Training min, max, mean, SD: 0, 138.03, 28.978, 32.1.
5. OPS PC1 out of range. Value: 8.6119. Training min, max, SD, explained variance: -4.0008, 7.9165, 2.861, 0.2531.
6. Unknown FCFP_2 feature: -1539162406: [*]C[c]1:n:[*]:[*]:n:1:[*]
7. Unknown FCFP_2 feature: -1564473960: [*]:n1:[*]:[*]:n:[c]:1:[c](:[*]):[*]
8. Unknown FCFP_2 feature: -332197802: [*][c]1:[*]:[*]:[c](:[*]):n:1:n:[*]

Feature Contribution

Top features for positive contribution			
Fingerprint	Bit/Smiles	Feature Structure	Score

FCFP_2	1	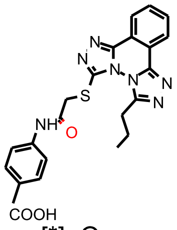 [*]=O	0.511
FCFP_2	3	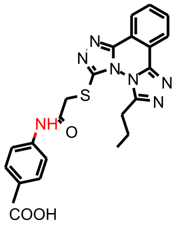 [*]N[*]	0.104
FCFP_2	-1272798659	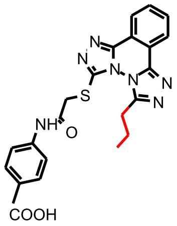 [*]CCC	0.0703

Top Features for negative contribution

**Fingerprint**	**Bit/Smiles**	**Feature Structure**	**Score**
FCFP_2	136597326	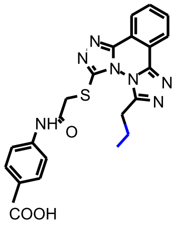 [*]CC	-0.489
FCFP_2	203677720	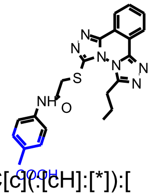 [*]=C[c](:[cH]:[*]):[cH]:[*]	-0.406

FCFP_2	1872154524	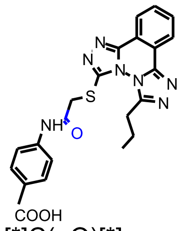<br>[*]C(=O)[*]	-0.307

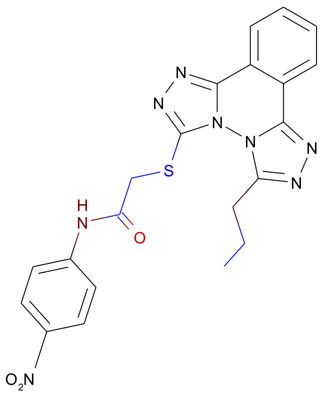

$C_{21}H_{18}N_8O_3S$
Molecular Weight: 462.48441
ALogP: 3.685
Rotatable Bonds: 7
Acceptors: 8
Donors: 1

Model Prediction

Prediction: 0.00306
Unit: g/kg_body_weight
Mahalanobis Distance: 18.8
Mahalanobis Distance p-value: 4.78e-018

Mahalanobis Distance: The Mahalanobis distance (MD) is a generalization of the Euclidean distance that accounts for correlations among the X properties. It is calculated as the distance to the center of the training data. The larger the MD, the less trustworthy the prediction.
Mahalanobis Distance p-value: The p-value gives the fraction of training data with an MD greater than or equal to the one for the given sample, assuming normally distributed data. The smaller the p-value, the less trustworthy the prediciton. For highly non-normal X properties (e.g., fingerprints), the MD p-value is wildly inaccurate.

Structural Similar Compounds

Name	OCHRATOXIN	SULFISOOXAZOLE	PENICILLIN VK
Structure	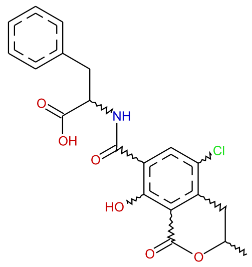	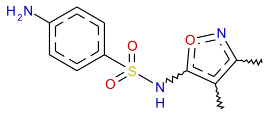	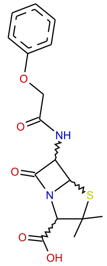
Actual Endpoint (-log C)	6.28396	2.82494	2.54455
Predicted Endpoint (-log C)	5.12358	3.0705	3.9702
Distance	1.372	1.468	1.544
Reference	NCI/NTP TR-358	NCI/NTP TR-138	NCI/NTP TR-336

Model Applicability

Unknown features are fingerprint features in the query molecule, but not found or appearing too infrequently in the training set.

1. Molecular_Weight out of range. Value: 462.48. Training min, max, mean, SD: 68.074, 434.63, 171.13, 85.06.
2. Num_H_Acceptors out of range. Value: 8. Training min, max, mean, SD: 0, 6, 1.6146, 1.644.
3. Num_AromaticRings out of range. Value: 5. Training min, max, mean, SD: 0, 2, 0.5625, 0.693.
4. Molecular_PolarSurfaceArea out of range. Value: 160.6. Training min, max, mean, SD: 0, 138.03, 28.978, 32.1.
5. OPS PC9 out of range. Value: -2.7709. Training min, max, SD, explained variance: -2.7086, 2.9267, 1.019, 0.0321.
6. Unknown FCFP_2 feature: 8: [*][N+](=[*])[*]
7. Unknown FCFP_2 feature: 5: [*][O-]
8. Unknown FCFP_2 feature: -1539162406: [*]C[c]1:n:[*]:[*]:n:1:[*]
9. Unknown FCFP_2 feature: -1564473960: [*]:n1:[*]:[*]:n:[c]:1:[c](:[*]):[*]
10. Unknown FCFP_2 feature: -332197802: [*][c]1:[*]:[*]:[c](:[*]):n:1:n:[*]
11. Unknown FCFP_2 feature: -828984032: [*][N+](=[*])[c](:[cH]:[*]):[cH]:[*]
12. Unknown FCFP_2 feature: -1338588315: [*]:[c](:[*])[N+](=O)[O-]
13. Unknown FCFP_2 feature: 1872392852: [*][N+](=O)[*]
14. Unknown FCFP_2 feature: 260476081: [*][N+](=[*])[O-]

Feature Contribution

Top features for positive contribution

Fingerprint	Bit/Smiles	Feature Structure	Score
FCFP_2	1	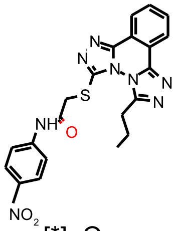 [*]=O	0.511
FCFP_2	3	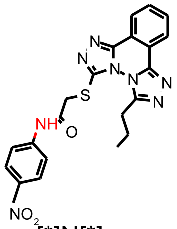 [*]N[*]	0.104
FCFP_2	-1272798659	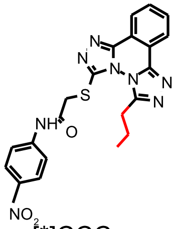 [*]CCC	0.0703
**Top Features for negative contribution**			
**Fingerprint**	**Bit/Smiles**	**Feature Structure**	**Score**
FCFP_2	136597326	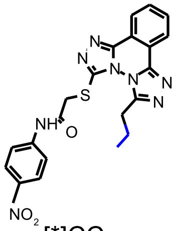 [*]CC	-0.489

FCFP_2	1872154524	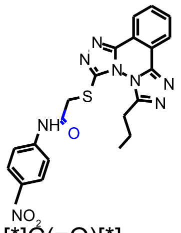 [*]C(=O)[*]	-0.307
FCFP_2	0	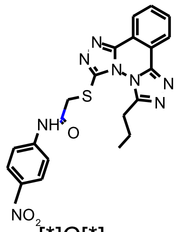 [*]C[*]	-0.29

14c

TOPKAT_Rat_Maximum_Tolerated_Dose_Gavage

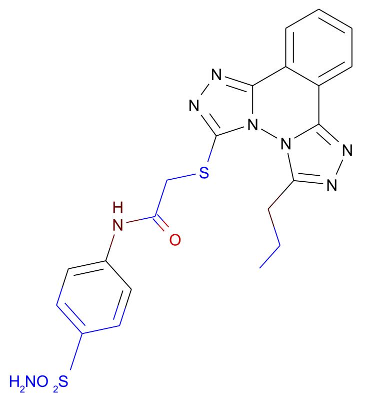

$C_{21}H_{20}N_8O_3S_2$

Molecular Weight: 496.56529

ALogP: 2.496

Rotatable Bonds: 7

Acceptors: 8

Donors: 2

Model Prediction

Prediction: 0.0275

Unit: g/kg_body_weight

Mahalanobis Distance: 16

Mahalanobis Distance p-value: 2.39e-014

Mahalanobis Distance: The Mahalanobis distance (MD) is a generalization of the Euclidean distance that accounts for correlations among the X properties. It is calculated as the distance to the center of the training data. The larger the MD, the less trustworthy the prediction.
Mahalanobis Distance p-value: The p-value gives the fraction of training data with an MD greater than or equal to the one for the given sample, assuming normally distributed data. The smaller the p-value, the less trustworthy the prediciton. For highly non-normal X properties (e.g., fingerprints), the MD p-value is wildly inaccurate.

Structural Similar Compounds

Name	OCHRATOXIN	SULFISOOXAZOLE	PENICILLIN VK
Structure	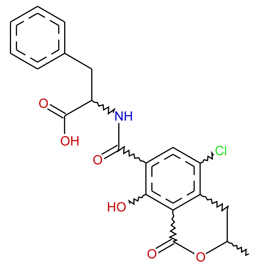	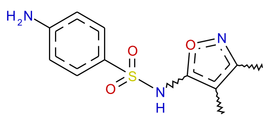	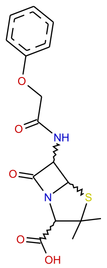
Actual Endpoint (-log C)	6.28396	2.82494	2.54455
Predicted Endpoint (-log C)	5.12358	3.0705	3.9702
Distance	1.363	1.453	1.562
Reference	NCI/NTP TR-358	NCI/NTP TR-138	NCI/NTP TR-336

Model Applicability

Unknown features are fingerprint features in the query molecule, but not found or appearing too infrequently in the training set.

1. Molecular_Weight out of range. Value: 496.57. Training min, max, mean, SD: 68.074, 434.63, 171.13, 85.06.
2. Num_H_Acceptors out of range. Value: 8. Training min, max, mean, SD: 0, 6, 1.6146, 1.644.
3. Num_AromaticRings out of range. Value: 5. Training min, max, mean, SD: 0, 2, 0.5625, 0.693.
4. Molecular_PolarSASA out of range. Value: 269.26. Training min, max, mean, SD: 0, 223.97, 50.816, 55.15.
5. Molecular_PolarSurfaceArea out of range. Value: 183.32. Training min, max, mean, SD: 0, 138.03, 28.978, 32.1.
6. OPS PC1 out of range. Value: 8.7116. Training min, max, SD, explained variance: -4.0008, 7.9165, 2.861, 0.2531.
7. OPS PC6 out of range. Value: -2.4899. Training min, max, SD, explained variance: -2.4321, 2.9885, 1.256, 0.0488.
8. OPS PC9 out of range. Value: -3.1064. Training min, max, SD, explained variance: -2.7086, 2.9267, 1.019, 0.0321.
9. Unknown FCFP_2 feature: -1539162406: [*]C[c]1:n:[*]:[*]:n:1:[*]
10. Unknown FCFP_2 feature: -1564473960: [*]:n1:[*]:[*]:n:[c]:1:[c](:[*]):[*]
11. Unknown FCFP_2 feature: -332197802: [*][c]1:[*]:[*]:[c](:[*]):n:1:n:[*]

Feature Contribution

Top features for positive contribution

Fingerprint	Bit/Smiles	Feature Structure	Score
FCFP_2	1	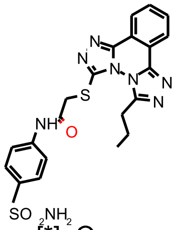 [*]=O	0.511
FCFP_2	3	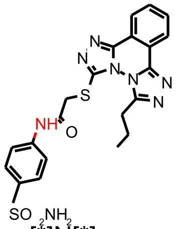 [*]N[*]	0.104
FCFP_2	-1272798659	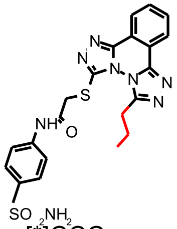 [*]CCC	0.0703
**Top Features for negative contribution**			
**Fingerprint**	**Bit/Smiles**	**Feature Structure**	**Score**
FCFP_2	136597326	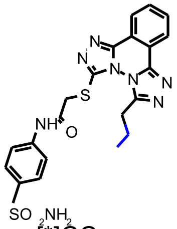 [*]CC	-0.489

FCFP_2	203677720	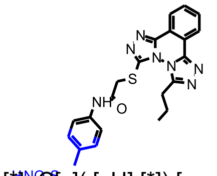 [*]=C[c](:[cH]:[*]):[cH]:[*]	-0.406
FCFP_2	1872154524	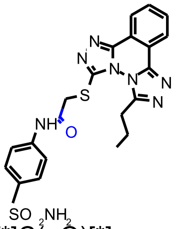 [*]C(=O)[*]	-0.307

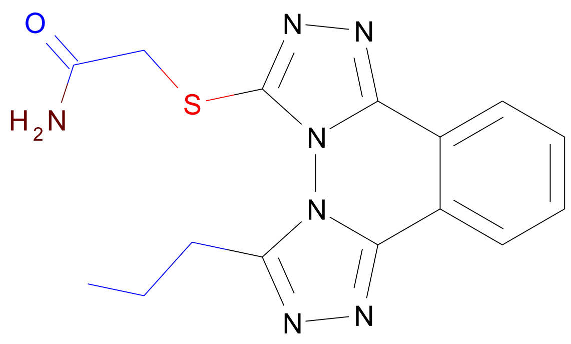

$C_{15}H_{15}N_7OS$
Molecular Weight: 341.39089
ALogP: 2.008
Rotatable Bonds: 5
Acceptors: 6
Donors: 1

Model Prediction

Prediction: 0.0362
Unit: g/kg_body_weight
Mahalanobis Distance: 15
Mahalanobis Distance p-value: 5.46e-013

Mahalanobis Distance: The Mahalanobis distance (MD) is a generalization of the Euclidean distance that accounts for correlations among the X properties. It is calculated as the distance to the center of the training data. The larger the MD, the less trustworthy the prediction.
Mahalanobis Distance p-value: The p-value gives the fraction of training data with an MD greater than or equal to the one for the given sample, assuming normally distributed data. The smaller the p-value, the less trustworthy the prediciton. For highly non-normal X properties (e.g., fingerprints), the MD p-value is wildly inaccurate.

Structural Similar Compounds

Name	SULFISOOXAZOLE	OCHRATOXIN	PENICILLIN VK
Structure	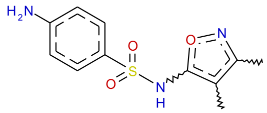	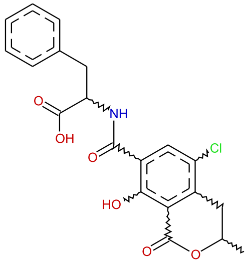	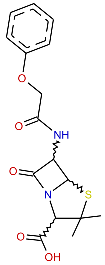
Actual Endpoint (-log C)	2.82494	6.28396	2.54455
Predicted Endpoint (-log C)	3.0705	5.12358	3.9702
Distance	1.009	1.086	1.153
Reference	NCI/NTP TR-138	NCI/NTP TR-358	NCI/NTP TR-336

Model Applicability

Unknown features are fingerprint features in the query molecule, but not found or appearing too infrequently in the training set.

1. Num_AromaticRings out of range. Value: 4. Training min, max, mean, SD: 0, 2, 0.5625, 0.693.
2. Unknown FCFP_2 feature: -1564473960: [*]:n1:[*]:[*]:n:[c]:1:[c](:[*]):[*]
3. Unknown FCFP_2 feature: -332197802: [*][c]1:[*]:[*]:[c](:[*]):n:1:n:[*]
4. Unknown FCFP_2 feature: -1539162406: [*]C[c]1:n:[*]:[*]:n:1:[*]

Feature Contribution

Top features for positive contribution

Fingerprint	Bit/Smiles	Feature Structure	Score
FCFP_2	1	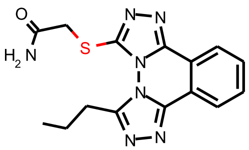 [*]=O	0.511

FCFP_2	3	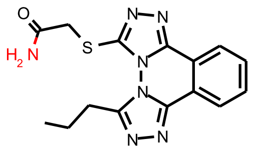<br>[*]N[*]	0.104
FCFP_2	-1272798659	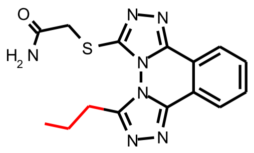<br>[*]CCC	0.0703
**Top Features for negative contribution**			
**Fingerprint**	**Bit/Smiles**	**Feature Structure**	**Score**
FCFP_2	136597326	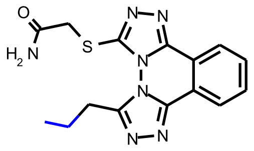<br>[*]CC	-0.489
FCFP_2	1872154524	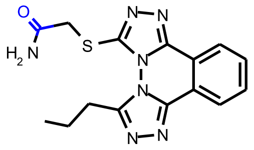<br>[*]C(=O)[*]	-0.307
FCFP_2	0	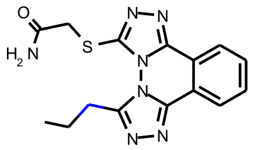<br>[*]C[*]	-0.29

Doxorubicin

TOPKAT_Rat_Maximum_Tolerated_Dose_Gavage

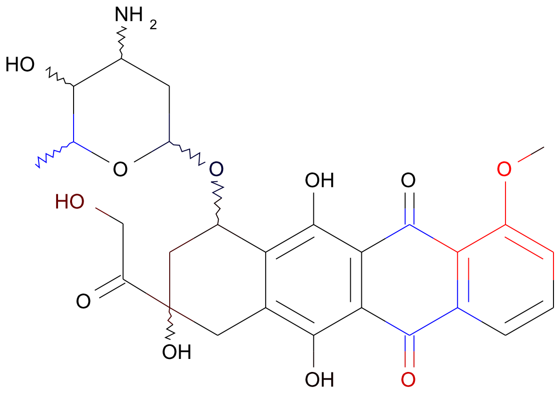

$C_{27}H_{29}NO_{11}$

Molecular Weight: 543.51925

ALogP: -4.4e-002

Rotatable Bonds: 5

Acceptors: 12

Donors: 6

Model Prediction

Prediction: 4.1e-005

Unit: g/kg_body_weight

Mahalanobis Distance: 18.3

Mahalanobis Distance p-value: 1.97e-017

Mahalanobis Distance: The Mahalanobis distance (MD) is a generalization of the Euclidean distance that accounts for correlations among the X properties. It is calculated as the distance to the center of the training data. The larger the MD, the less trustworthy the prediction.
Mahalanobis Distance p-value: The p-value gives the fraction of training data with an MD greater than or equal to the one for the given sample, assuming normally distributed data. The smaller the p-value, the less trustworthy the prediciton. For highly non-normal X properties (e.g., fingerprints), the MD p-value is wildly inaccurate.

Structural Similar Compounds

Name	AMPICILLIN TRIHYDRATE	OCHRATOXIN	PENICILLIN VK
Structure	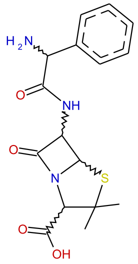	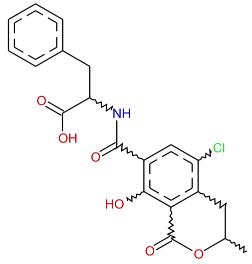	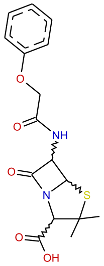
Actual Endpoint (-log C)	2.36724	6.28396	2.54455
Predicted Endpoint (-log C)	2.27651	5.12358	3.9702
Distance	1.486	1.531	1.674
Reference	NCI/NTP TR-318	NCI/NTP TR-358	NCI/NTP TR-336

Model Applicability

Unknown features are fingerprint features in the query molecule, but not found or appearing too infrequently in the training set.

1. Molecular_Weight out of range. Value: 543.52. Training min, max, mean, SD: 68.074, 434.63, 171.13, 85.06.
2. Num_H_Donors out of range. Value: 6. Training min, max, mean, SD: 0, 3, 0.4375, 0.8311.
3. Num_H_Acceptors out of range. Value: 12. Training min, max, mean, SD: 0, 6, 1.6146, 1.644.
4. Molecular_PolarSASA out of range. Value: 345.3. Training min, max, mean, SD: 0, 223.97, 50.816, 55.15.
5. Molecular_PolarSurfaceArea out of range. Value: 206.07. Training min, max, mean, SD: 0, 138.03, 28.978, 32.1.
6. OPS PC1 out of range. Value: 12.738. Training min, max, SD, explained variance: -4.0008, 7.9165, 2.861, 0.2531.
7. Unknown FCFP_2 feature: -1549669478: [*]:[c](:[*])C(=O)[c](:[*]):[*]
8. Unknown FCFP_2 feature: -415156552: [*]CC(O)(C[*])C(=[*])[*]

Feature Contribution

Top features for positive contribution

Fingerprint	Bit/Smiles	Feature Structure	Score

FCFP_2	332760439	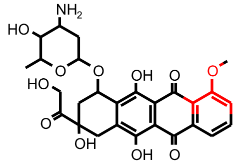 [*]O[c](:[cH]:[*]):[c]([*]):[*]	0.672
FCFP_2	1	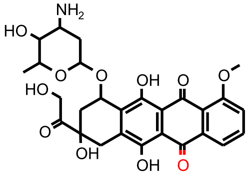 [*]=O	0.511
FCFP_2	3	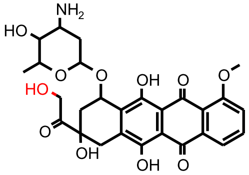 [*]N[*]	0.104
**Top Features for negative contribution**			
**Fingerprint**	**Bit/Smiles**	**Feature Structure**	**Score**
FCFP_2	136597326	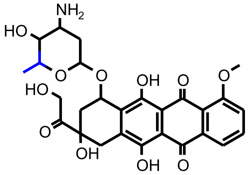 [*]CC	-0.489
FCFP_2	203677720	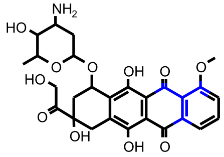 [*]=C[c](:[cH]:[*]):[cH]:[*]	-0.406

FCFP_2	1872154524	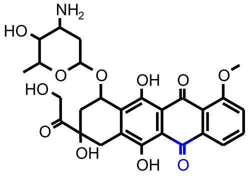<br>[*]C(=O)[*]	-0.307

7a TOPKAT_Rat_Oral_LD50

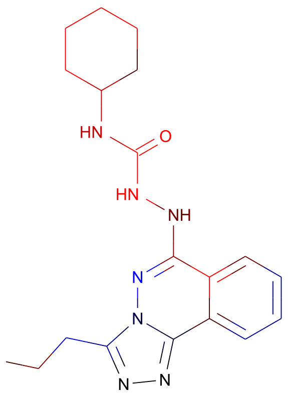

$C_{19}H_{25}N_7O$

Molecular Weight: 367.44809

ALogP: 3.681

Rotatable Bonds: 5

Acceptors: 5

Donors: 3

Structural Similar Compounds

Name	FLUBENDAZOLE	CARBAMIC ACID; N-(5-BENZOYLBENZIMIDAZOL-2-YL)-; METHYL ESTER	METHYL-1-(BUTYLCARBAMOYL)-2-BENZIMIDAZOLE CARBAMATE
Structure	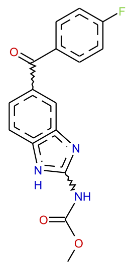	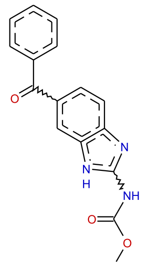	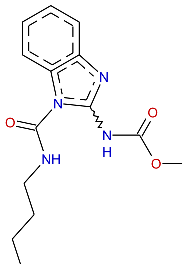
Actual Endpoint (-log C)	2.088	2.617	1.463
Predicted Endpoint (-log C)	2.69288	2.2368	1.78491
Distance	0.641	0.651	0.701
Reference	YRTMA6 9;11;78	IYKEDH 19;735;88	JHEMA2 24;295;80

Model Prediction

Prediction: 0.861

Unit: g/kg_body_weight

Mahalanobis Distance: 27.2

Mahalanobis Distance p-value: 1.37e-044

Mahalanobis Distance: The Mahalanobis distance (MD) is a generalization of the Euclidean distance that accounts for correlations among the X properties. It is calculated as the distance to the center of the training data. The larger the MD, the less trustworthy the prediction.

Mahalanobis Distance p-value: The p-value gives the fraction of training data with an MD greater than or equal to the one for the given sample, assuming normally distributed data. The smaller the p-value, the less trustworthy the prediciton. For highly non-normal X properties (e.g., fingerprints), the MD p-value is wildly inaccurate.

Model Applicability

Unknown features are fingerprint features in the query molecule, but not found or appearing too infrequently in the training set.

1. All properties and OPS components are within expected ranges.
2. Unknown FCFP_6 feature: 16: [*]:[cH]:[*]
3. Unknown FCFP_6 feature: 1618154665: [*]:[cH]:[cH]:[cH]:[*]
4. Unknown FCFP_6 feature: -1151884458: [*]N[c](:n:[*]):[c](:[*]):[*]
5. Unknown FCFP_6 feature: 4427049: [*][c](:[*]):n:n(:[*]):[*]
6. Unknown FCFP_6 feature: -332197802: [*][c]1:[*]:[*]:[c](:[*]):n:1:n:[*]
7. Unknown FCFP_6 feature: -1564473960: [*]:n1:[*]:[*]:n:[c]:1:[c](:[*]):[*]
8. Unknown FCFP_6 feature: 1294344583: [*]NN[c](:[*]):[*]
9. Unknown FCFP_6 feature: -1539162406: [*]C[c]1:n:[*]:[*]:n:1:[*]
10. Unknown FCFP_6 feature: -885461129: [*]NNC(=[*])[*]

Feature Contribution

Top features for positive contribution			
**Fingerprint**	**Bit/Smiles**	**Feature Structure**	**Score**

ECFP_6	642810091	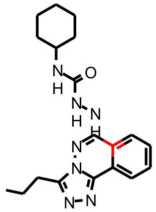 [*]:[c](:[*]):[*]	0.281
FCFP_6	1499521844	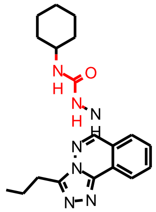 [*]NC(=O)N[*]	0.258
ECFP_6	-1897341097	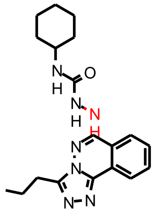 [*]N[*]	0.216
**Top Features for negative contribution**			
**Fingerprint**	**Bit/Smiles**	**Feature Structure**	**Score**
ECFP_6	655739385	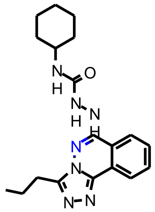 [*]:n:[*]	-0.239
ECFP_6	734603939	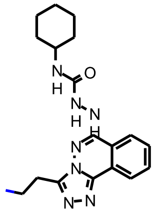 [*]C	-0.201

ECFP_6	-1795525632	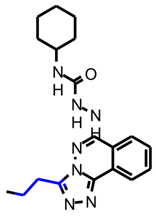<br>[*]CC[c](:[*]):[*]	-0.176

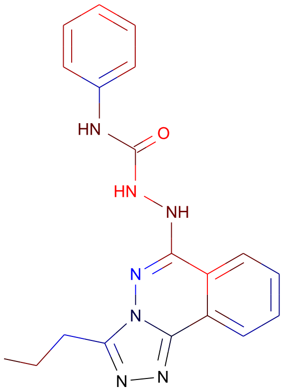

$C_{19}H_{19}N_7O$
Molecular Weight: 361.40046
ALogP: 3.399
Rotatable Bonds: 5
Acceptors: 5
Donors: 3

Structural Similar Compounds

Name	CARBAMIC ACID; N-(5-BENZOYLBENZIMIDAZOL-2-YL)-; METHYL ESTER	FLUBENDAZOLE	SULFAQUINOXALINE
Structure	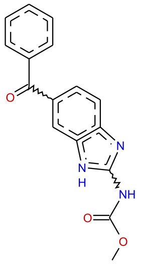	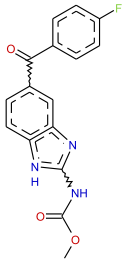	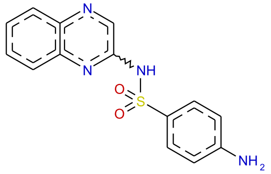
Actual Endpoint (-log C)	2.617	2.088	2.341
Predicted Endpoint (-log C)	2.2368	2.69288	2.42674
Distance	0.705	0.709	0.785
Reference	IYKEDH 19;735;88	YRTMA6 9;11;78	MahWM# 16NOV82

Model Prediction

Prediction: 2.51
Unit: g/kg_body_weight
Mahalanobis Distance: 27.5
Mahalanobis Distance p-value: 2.14e-046

Mahalanobis Distance: The Mahalanobis distance (MD) is a generalization of the Euclidean distance that accounts for correlations among the X properties. It is calculated as the distance to the center of the training data. The larger the MD, the less trustworthy the prediction.
Mahalanobis Distance p-value: The p-value gives the fraction of training data with an MD greater than or equal to the one for the given sample, assuming normally distributed data. The smaller the p-value, the less trustworthy the prediciton. For highly non-normal X properties (e.g., fingerprints), the MD p-value is wildly inaccurate.

Model Applicability

Unknown features are fingerprint features in the query molecule, but not found or appearing too infrequently in the training set.

1. All properties and OPS components are within expected ranges.
2. Unknown FCFP_6 feature: 16: [*]:[cH]:[*]
3. Unknown FCFP_6 feature: 1618154665: [*]:[cH]:[cH]:[cH]:[*]
4. Unknown FCFP_6 feature: -1151884458: [*]N[c](:n:[*]):[c](:[*]):[*]
5. Unknown FCFP_6 feature: 4427049: [*][c](:[*]):n:n(:[*]):[*]
6. Unknown FCFP_6 feature: -332197802: [*][c]1:[*]:[*]:[c](:[*]):n:1:n:[*]
7. Unknown FCFP_6 feature: -1564473960: [*]:n1:[*]:[*]:n:[c]:1:[c](:[*]):[*]
8. Unknown FCFP_6 feature: 1294344583: [*]NN[c](:[*]):[*]
9. Unknown FCFP_6 feature: -1539162406: [*]C[c]1:n:[*]:[*]:n:1:[*]
10. Unknown FCFP_6 feature: -885461129: [*]NNC(=[*])[*]

Feature Contribution

Top features for positive contribution

Fingerprint	Bit/Smiles	Feature Structure	Score

ECFP_6	642810091	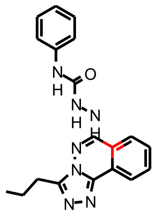 [*]:[c](:[*]):[*]	0.281
FCFP_6	1499521844	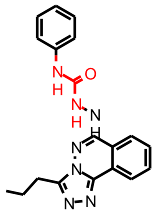 [*]NC(=O)N[*]	0.258
ECFP_6	-1897341097	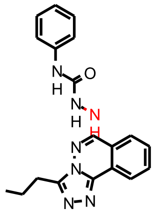 [*]N[*]	0.216
**Top Features for negative contribution**			
**Fingerprint**	**Bit/Smiles**	**Feature Structure**	**Score**
ECFP_6	655739385	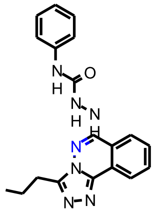 [*]:n:[*]	-0.239
ECFP_6	734603939	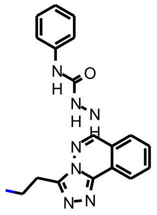 [*]C	-0.201

ECFP_6	-1795525632	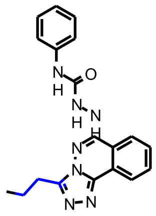 [*]CC[c](:[*]):[*]	-0.176

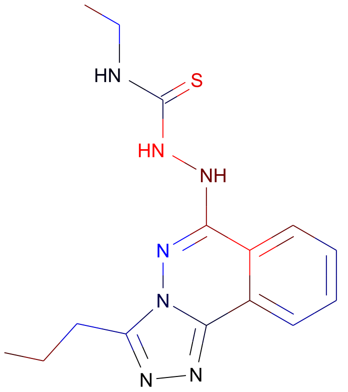

$C_{15}H_{19}N_7S$

Molecular Weight: 329.42325

ALogP: 3.388

Rotatable Bonds: 7

Acceptors: 5

Donors: 3

Structural Similar Compounds

Name	CARBAMIC ACID; [1-[(5-CYANOPENTYL)CARBAMOYL]BENZIMIDAZOL-2-YL]-; METHYL ESTER	OCHRATOXIN A	CARBAMIC ACID; N-(5-BENZOYLBENZIMIDAZOL-2-YL)-; METHYL ESTER
Structure	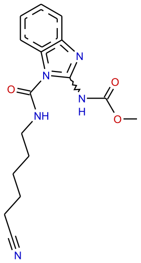	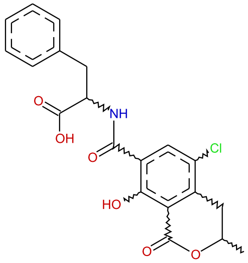	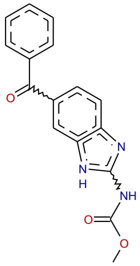
Actual Endpoint (-log C)	2.12	4.305	2.617
Predicted Endpoint (-log C)	1.78415	3.03558	2.2368
Distance	0.666	0.720	0.725
Reference	85ARAE 4;118;76/77	FCTXAV 6;479;68	IYKEDH 19;735;88

Model Prediction

Prediction: 0.742

Unit: g/kg_body_weight

Mahalanobis Distance: 26.7

Mahalanobis Distance p-value: 2.43e-041

Mahalanobis Distance: The Mahalanobis distance (MD) is a generalization of the Euclidean distance that accounts for correlations among the X properties. It is calculated as the distance to the center of the training data. The larger the MD, the less trustworthy the prediction.
Mahalanobis Distance p-value: The p-value gives the fraction of training data with an MD greater than or equal to the one for the given sample, assuming normally distributed data. The smaller the p-value, the less trustworthy the prediciton. For highly non-normal X properties (e.g., fingerprints), the MD p-value is wildly inaccurate.

Model Applicability

Unknown features are fingerprint features in the query molecule, but not found or appearing too infrequently in the training set.

1. All properties and OPS components are within expected ranges.
2. Unknown FCFP_6 feature: 16: [*]:[cH]:[*]
3. Unknown FCFP_6 feature: 1618154665: [*]:[cH]:[cH]:[cH]:[*]
4. Unknown FCFP_6 feature: -1151884458: [*]N[c](:n:[*]):[c](:[*]):[*]
5. Unknown FCFP_6 feature: 4427049: [*][c](:[*]):n:n(:[*]):[*]
6. Unknown FCFP_6 feature: -332197802: [*][c]1:[*]:[*]:[c](:[*]):n:1:n:[*]
7. Unknown FCFP_6 feature: -1564473960: [*]:n1:[*]:[*]:n:[c]:1:[c](:[*]):[*]
8. Unknown FCFP_6 feature: 1294344583: [*]NN[c](:[*]):[*]
9. Unknown FCFP_6 feature: -1539162406: [*]C[c]1:n:[*]:[*]:n:1:[*]
10. Unknown FCFP_6 feature: -885461129: [*]NNC(=[*])[*]

Feature Contribution

Top features for positive contribution			
Fingerprint	Bit/Smiles	Feature Structure	Score

ECFP_6	642810091	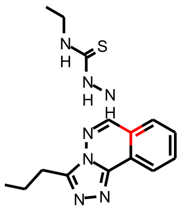 [*]:[c](:[*]):[*]	0.281
FCFP_6	1499521844	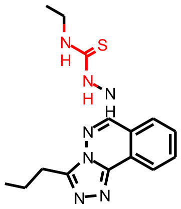 [*]NC(=O)N[*]	0.258
ECFP_6	-1897341097	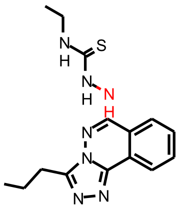 [*]N[*]	0.216
**Top Features for negative contribution**			
**Fingerprint**	**Bit/Smiles**	**Feature Structure**	**Score**
ECFP_6	497523368	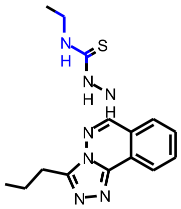 [*]CNC(=[*])[*]	-0.301
ECFP_6	655739385	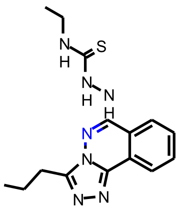 [*]:n:[*]	-0.239

ECFP_6	734603939	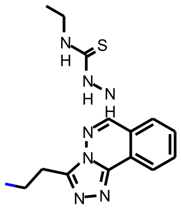 [*]C	-0.201

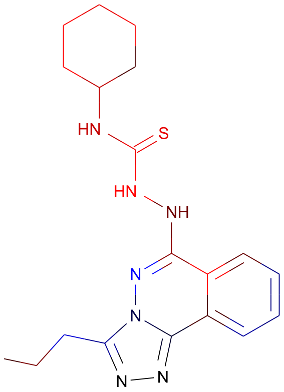

$C_{19}H_{25}N_7S$

Molecular Weight: 383.51369

ALogP: 4.898

Rotatable Bonds: 7

Acceptors: 5

Donors: 3

Structural Similar Compounds

Name	PHOSPHORAMIDOTHIOIC ACID; ACETIMIDOYL-; O;O-bis-(p-CHLOROPHENYL)ESTER	OCHRATOXIN A	[4-CHLORO-6-(2;3-XYLIDINO)2-PYRIMIDINYLTHIO]ACETIC ACID
Structure	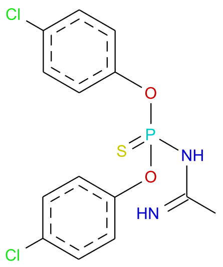	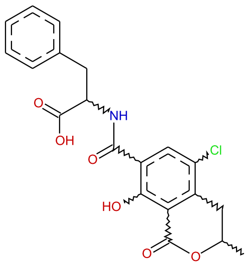	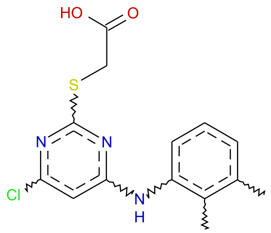
Actual Endpoint (-log C)	5.006	4.305	1.892
Predicted Endpoint (-log C)	3.23989	3.03558	3.41405
Distance	0.716	0.730	0.737
Reference	FMCHA2 -;C149;89	FCTXAV 6;479;68	DRFUD4 4;273;79

Model Prediction

Prediction: 0.297

Unit: g/kg_body_weight

Mahalanobis Distance: 27.1

Mahalanobis Distance p-value: 1.27e-043

Mahalanobis Distance: The Mahalanobis distance (MD) is a generalization of the Euclidean distance that accounts for correlations among the X properties. It is calculated as the distance to the center of the training data. The larger the MD, the less trustworthy the prediction.

Mahalanobis Distance p-value: The p-value gives the fraction of training data with an MD greater than or equal to the one for the given sample, assuming normally distributed data. The smaller the p-value, the less trustworthy the prediciton. For highly non-normal X properties (e.g., fingerprints), the MD p-value is wildly inaccurate.

Model Applicability

Unknown features are fingerprint features in the query molecule, but not found or appearing too infrequently in the training set.

1. All properties and OPS components are within expected ranges.
2. Unknown FCFP_6 feature: 16: [*]:[cH]:[*]
3. Unknown FCFP_6 feature: 1618154665: [*]:[cH]:[cH]:[cH]:[*]
4. Unknown FCFP_6 feature: -1151884458: [*]N[c](:n:[*]):[c](:[*]):[*]
5. Unknown FCFP_6 feature: 4427049: [*][c](:[*]):n:n(:[*]):[*]
6. Unknown FCFP_6 feature: -332197802: [*][c]1:[*]:[*]:[c](:[*]):n:1:n:[*]
7. Unknown FCFP_6 feature: -1564473960: [*]:n1:[*]:[*]:n:[c]:1:[c](:[*]):[*]
8. Unknown FCFP_6 feature: 1294344583: [*]NN[c](:[*]):[*]
9. Unknown FCFP_6 feature: -1539162406: [*]C[c]1:n:[*]:[*]:n:1:[*]
10. Unknown FCFP_6 feature: -885461129: [*]NNC(=[*])[*]

Feature Contribution

Top features for positive contribution			
**Fingerprint**	**Bit/Smiles**	**Feature Structure**	**Score**

ECFP_6	642810091	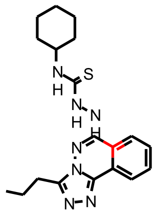 [*]:[c](:[*]):[*]	0.281
FCFP_6	1499521844	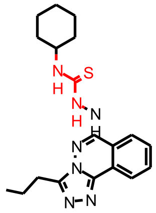 [*]NC(=O)N[*]	0.258
ECFP_6	-1897341097	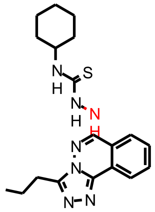 [*]N[*]	0.216
**Top Features for negative contribution**			
**Fingerprint**	**Bit/Smiles**	**Feature Structure**	**Score**
ECFP_6	655739385	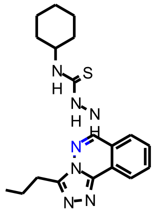 [*]:n:[*]	-0.239
ECFP_6	734603939	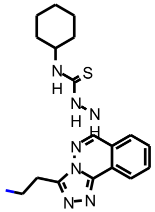 [*]C	-0.201

ECFP_6	-1795525632	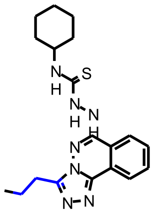 [*]CC[c](:[*]):[*]	-0.176

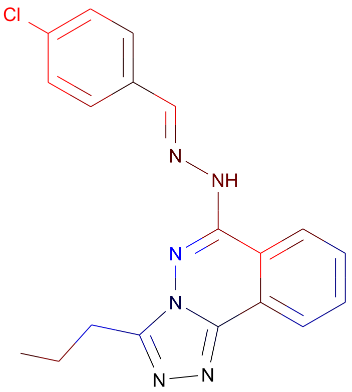

$C_{19}H_{17}ClN_6$
Molecular Weight: 364.83147
ALogP: 4.967
Rotatable Bonds: 5
Acceptors: 5
Donors: 1

Model Prediction

Prediction: 0.447
Unit: g/kg_body_weight
Mahalanobis Distance: 23.9
Mahalanobis Distance p-value: 2.66e-025

Mahalanobis Distance: The Mahalanobis distance (MD) is a generalization of the Euclidean distance that accounts for correlations among the X properties. It is calculated as the distance to the center of the training data. The larger the MD, the less trustworthy the prediction.
Mahalanobis Distance p-value: The p-value gives the fraction of training data with an MD greater than or equal to the one for the given sample, assuming normally distributed data. The smaller the p-value, the less trustworthy the prediciton. For highly non-normal X properties (e.g., fingerprints), the MD p-value is wildly inaccurate.

Structural Similar Compounds

Name	TALNIFLUMATE	INDOMETHAZINE	ACRIDINE; 9-[3-(DIMETHYLAMINO)PROPYLAMINO]-1-NITRO-
Structure	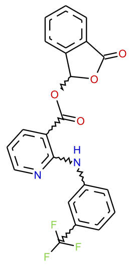	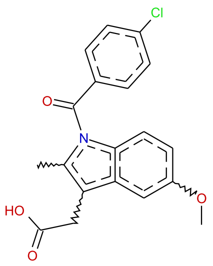	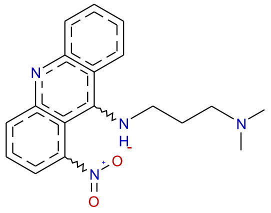
Actual Endpoint (-log C)	1.538	5.17	4.101
Predicted Endpoint (-log C)	2.82541	3.33605	3.3633
Distance	0.671	0.676	0.687
Reference	FRPSAX 36;372;81	ARZNAD 25;1526;75	MMDPA6 8;252;76

Model Applicability

Unknown features are fingerprint features in the query molecule, but not found or appearing too infrequently in the training set.

1. All properties and OPS components are within expected ranges.
2. Unknown FCFP_6 feature: 16: [*]:[cH]:[*]
3. Unknown FCFP_6 feature: 1618154665: [*]:[cH]:[cH]:[cH]:[*]
4. Unknown FCFP_6 feature: -1151884458: [*]N[c](:n:[*]):[c](:[*]):[*]
5. Unknown FCFP_6 feature: 4427049: [*][c](:[*]):n:n(:[*]):[*]
6. Unknown FCFP_6 feature: -332197802: [*][c]1:[*]:[*]:[c](:[*]):n:1:n:[*]
7. Unknown FCFP_6 feature: -1564473960: [*]:n1:[*]:[*]:n:[c]:1:[c](:[*]):[*]
8. Unknown FCFP_6 feature: -1539162406: [*]C[c]1:n:[*]:[*]:n:1:[*]
9. Unknown FCFP_6 feature: 1294285001: [*]=NN[c](:[*]):[*]
10. Unknown FCFP_6 feature: 581019816: [*]N\N=C\[*]
11. Unknown FCFP_6 feature: -2100785893: [*]\N=C\[c](:[*]):[*]
12. Unknown FCFP_6 feature: 71476542: [*]:[c](:[*])Cl

Feature Contribution

Top features for positive contribution			
Fingerprint	Bit/Smiles	Feature Structure	Score

ECFP_6	642810091	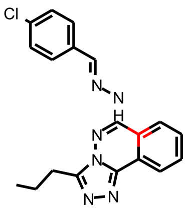 [*]:[c](:[*]):[*]	0.281
ECFP_6	-1897341097	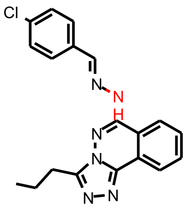 [*]N[*]	0.216
FCFP_6	-149636017	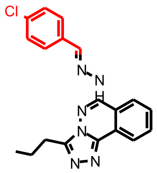 [*]=C[c]1:[cH]:[cH]:[c](Cl):[cH]:[cH]:1	0.193
**Top Features for negative contribution**			
**Fingerprint**	**Bit/Smiles**	**Feature Structure**	**Score**
ECFP_6	-817402818	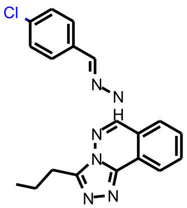 [*]Cl	-0.263
ECFP_6	655739385	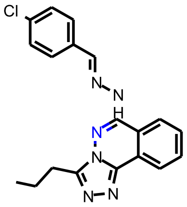 [*]:n:[*]	-0.239

ECFP_6	734603939	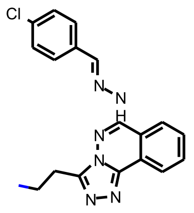<br>[*]C	-0.201

9b

TOPKAT_Rat_Oral_LD50

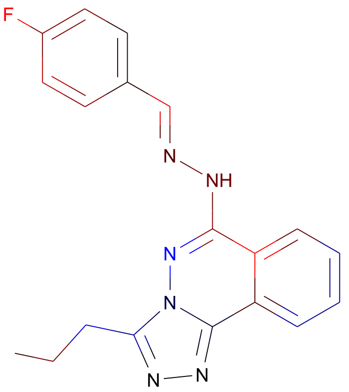

$C_{19}H_{17}FN_6$

Molecular Weight: 348.37688

ALogP: 4.508

Rotatable Bonds: 5

Acceptors: 5

Donors: 1

Model Prediction

Prediction: 0.229

Unit: g/kg_body_weight

Mahalanobis Distance: 25.2

Mahalanobis Distance p-value: 3e-032

Mahalanobis Distance: The Mahalanobis distance (MD) is a generalization of the Euclidean distance that accounts for correlations among the X properties. It is calculated as the distance to the center of the training data. The larger the MD, the less trustworthy the prediction.
Mahalanobis Distance p-value: The p-value gives the fraction of training data with an MD greater than or equal to the one for the given sample, assuming normally distributed data. The smaller the p-value, the less trustworthy the prediciton. For highly non-normal X properties (e.g., fingerprints), the MD p-value is wildly inaccurate.

Structural Similar Compounds

**Name**	**ACRIDINE; 9-[3-(DIMETHYLAMINO)PROPYLAMINO]-1-NITRO-**	**INDOMETHAZINE**	**TALNIFLUMATE**
Structure	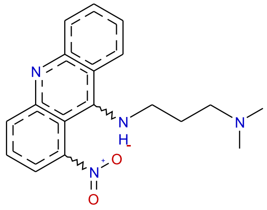	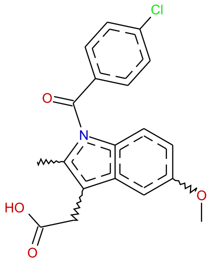	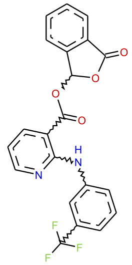
Actual Endpoint (-log C)	4.101	5.17	1.538
Predicted Endpoint (-log C)	3.3633	3.33605	2.82541
Distance	0.657	0.680	0.682
Reference	MMDPA6 8;252;76	ARZNAD 25;1526;75	FRPSAX 36;372;81

Model Applicability

Unknown features are fingerprint features in the query molecule, but not found or appearing too infrequently in the training set.

1. All properties and OPS components are within expected ranges.
2. Unknown FCFP_6 feature: 16: [*]:[cH]:[*]
3. Unknown FCFP_6 feature: 1618154665: [*]:[cH]:[cH]:[cH]:[*]
4. Unknown FCFP_6 feature: -1151884458: [*]N[c](:n:[*]):[c](:[*]):[*]
5. Unknown FCFP_6 feature: 4427049: [*][c](:[*]):n:n(:[*]):[*]
6. Unknown FCFP_6 feature: -332197802: [*][c]1:[*]:[*]:[c](:[*]):n:1:n:[*]
7. Unknown FCFP_6 feature: -1564473960: [*]:n1:[*]:[*]:n:[c]:1:[c](:[*]):[*]
8. Unknown FCFP_6 feature: -1539162406: [*]C[c]1:n:[*]:[*]:n:1:[*]
9. Unknown FCFP_6 feature: 1294285001: [*]=NN[c](:[*]):[*]
10. Unknown FCFP_6 feature: 581019816: [*]N\N=C\[*]
11. Unknown FCFP_6 feature: -2100785893: [*]\N=C\[c](:[*]):[*]
12. Unknown FCFP_6 feature: 71476542: [*]:[c](:[*])Cl

Feature Contribution

**Top features for positive contribution**			
**Fingerprint**	**Bit/Smiles**	**Feature Structure**	**Score**

ECFP_6	-1046436026	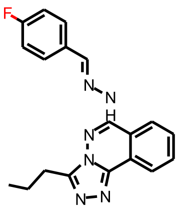 [*]F	0.349
ECFP_6	642810091	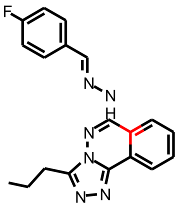 [*]:[c](:[*]):[*]	0.281
ECFP_6	-1897341097	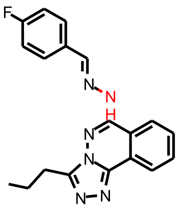 [*]N[*]	0.216
**Top Features for negative contribution**			
**Fingerprint**	**Bit/Smiles**	**Feature Structure**	**Score**
ECFP_6	655739385	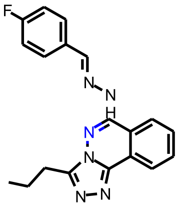 [*]:n:[*]	-0.239
ECFP_6	734603939	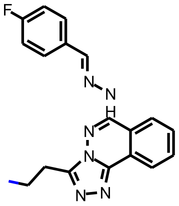 [*]C	-0.201

ECFP_6	-1795525632	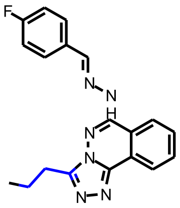 [*]CC[c](:[*]):[*]	-0.176

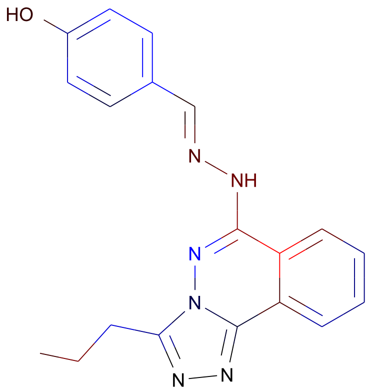

$C_{19}H_{18}N_6O$

Molecular Weight: 346.38582

ALogP: 4.061

Rotatable Bonds: 5

Acceptors: 6

Donors: 2

Model Prediction

Prediction: 0.501

Unit: g/kg_body_weight

Mahalanobis Distance: 24.6

Mahalanobis Distance p-value: 9.13e-029

Mahalanobis Distance: The Mahalanobis distance (MD) is a generalization of the Euclidean distance that accounts for correlations among the X properties. It is calculated as the distance to the center of the training data. The larger the MD, the less trustworthy the prediction.

Mahalanobis Distance p-value: The p-value gives the fraction of training data with an MD greater than or equal to the one for the given sample, assuming normally distributed data. The smaller the p-value, the less trustworthy the prediciton. For highly non-normal X properties (e.g., fingerprints), the MD p-value is wildly inaccurate.

Structural Similar Compounds

Name	ACRIDINE; 9-[3-(DIMETHYLAMINO)PROPYLAMINO]-1-NITRO-	FLUBENDAZOLE	CARBAMIC ACID; N-(5-BENZOYLBENZIMIDAZOL-2-YL)-; METHYL ESTER
Structure	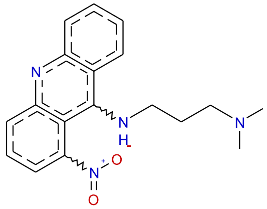	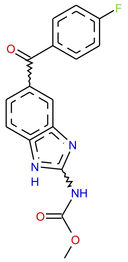	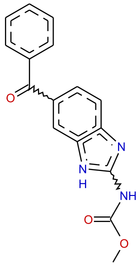
Actual Endpoint (-log C)	4.101	2.088	2.617
Predicted Endpoint (-log C)	3.3633	2.69288	2.2368
Distance	0.701	0.709	0.713
Reference	MMDPA6 8;252;76	YRTMA6 9;11;78	IYKEDH 19;735;88

Model Applicability

Unknown features are fingerprint features in the query molecule, but not found or appearing too infrequently in the training set.

1. All properties and OPS components are within expected ranges.
2. Unknown FCFP_6 feature: 16: [*]:[cH]:[*]
3. Unknown FCFP_6 feature: 1618154665: [*]:[cH]:[cH]:[cH]:[*]
4. Unknown FCFP_6 feature: -1151884458: [*]N[c](:n:[*]):[c](:[*]):[*]
5. Unknown FCFP_6 feature: 4427049: [*][c](:[*]):n:n(:[*]):[*]
6. Unknown FCFP_6 feature: -332197802: [*][c]1:[*]:[*]:[c](:[*]):n:1:n:[*]
7. Unknown FCFP_6 feature: -1564473960: [*]:n1:[*]:[*]:n:[c]:1:[c](:[*]):[*]
8. Unknown FCFP_6 feature: -1539162406: [*]C[c]1:n:[*]:[*]:n:1:[*]
9. Unknown FCFP_6 feature: 1294285001: [*]=NN[c](:[*]):[*]
10. Unknown FCFP_6 feature: 581019816: [*]N\N=C\[*]
11. Unknown FCFP_6 feature: -2100785893: [*]\N=C\[c](:[*]):[*]
12. Unknown FCFP_6 feature: 74595001: [*]:[cH]:[c](O):[cH]:[*]
13. Unknown FCFP_6 feature: -549108873: [*]:[c](:[*])O

Feature Contribution

Top features for positive contribution			
**Fingerprint**	**Bit/Smiles**	**Feature Structure**	**Score**

ECFP_6	642810091	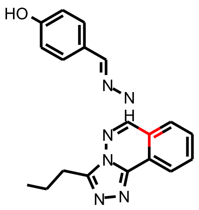 [*]:[c](:[*]):[*]	0.281
ECFP_6	-1897341097	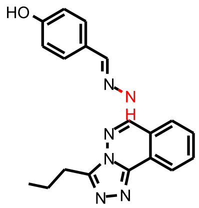 [*]N[*]	0.216
ECFP_6	2019062761	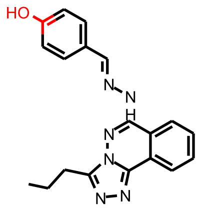 [*]:[c](:[*])O	0.138
**Top Features for negative contribution**			
**Fingerprint**	**Bit/Smiles**	**Feature Structure**	**Score**
ECFP_6	655739385	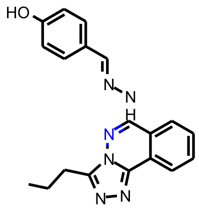 [*]:n:[*]	-0.239
FCFP_6	946589555	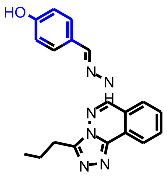 [*][c]1:[*]:[cH]:[c](O):[cH]:[cH]:1	-0.204

ECFP_6	734603939	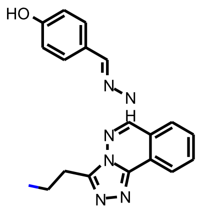 [*]C	-0.201

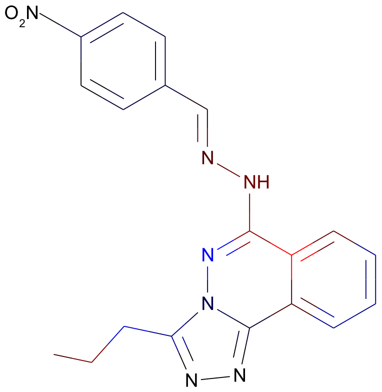

$C_{19}H_{17}N_7O_2$
Molecular Weight: 375.38397
ALogP: 4.197
Rotatable Bonds: 6
Acceptors: 7
Donors: 1

Model Prediction

Prediction: 0.573
Unit: g/kg_body_weight
Mahalanobis Distance: 27.1
Mahalanobis Distance p-value: 5.18e-044

Mahalanobis Distance: The Mahalanobis distance (MD) is a generalization of the Euclidean distance that accounts for correlations among the X properties. It is calculated as the distance to the center of the training data. The larger the MD, the less trustworthy the prediction.
Mahalanobis Distance p-value: The p-value gives the fraction of training data with an MD greater than or equal to the one for the given sample, assuming normally distributed data. The smaller the p-value, the less trustworthy the prediciton. For highly non-normal X properties (e.g., fingerprints), the MD p-value is wildly inaccurate.

Structural Similar Compounds

Name	ACEMETACIN	bis-OXATIN ACETATE	PRASOZIN .HCl (HCl STRIPPED)
Structure	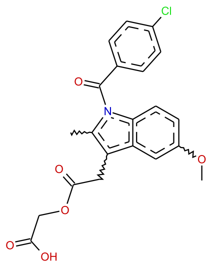	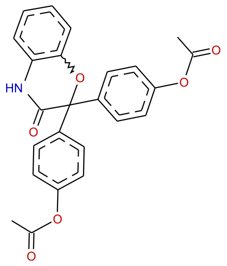	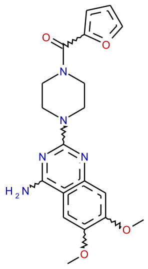
Actual Endpoint (-log C)	4.235	1.717	2.294
Predicted Endpoint (-log C)	3.39415	2.40947	3.00765
Distance	0.695	0.721	0.728
Reference	ARZNAD 30;1398;80	NIIRDN 6;609;82	NIIRDN 6;688;82

Model Applicability

Unknown features are fingerprint features in the query molecule, but not found or appearing too infrequently in the training set.

1. All properties and OPS components are within expected ranges.
2. Unknown ECFP_2 feature: 1043790491: [*][N+](=[*])[*]
3. Unknown ECFP_2 feature: 781519895: [*][O-]
4. Unknown ECFP_2 feature: -179073144: [*][N+](=[*])[c](:[cH]:[*]):[cH]:[*]
5. Unknown ECFP_2 feature: -215026467: [*]:[c](:[*])[N+](=O)[O-]
6. Unknown ECFP_2 feature: 2104376220: [*][N+](=O)[*]
7. Unknown ECFP_2 feature: -659271057: [*][N+](=[*])[O-]
8. Unknown FCFP_6 feature: 16: [*]:[cH]:[*]
9. Unknown FCFP_6 feature: 8: [*][N+](=[*])[*]
10. Unknown FCFP_6 feature: 5: [*][O-]
11. Unknown FCFP_6 feature: 1618154665: [*]:[cH]:[cH]:[cH]:[*]
12. Unknown FCFP_6 feature: -1151884458: [*]N[c](:n:[*]):[c](:[*]):[*]
13. Unknown FCFP_6 feature: 4427049: [*][c](:[*]):n:n(:[*]):[*]
14. Unknown FCFP_6 feature: -332197802: [*][c]1:[*]:[*]:[c](:[*]):n:1:n:[*]
15. Unknown FCFP_6 feature: -1564473960: [*]:n1:[*]:[*]:n:[c]:1:[c](:[*]):[*]
16. Unknown FCFP_6 feature: -1539162406: [*]C[c]1:n:[*]:[*]:n:1:[*]
17. Unknown FCFP_6 feature: 1294285001: [*]=NN[c](:[*]):[*]
18. Unknown FCFP_6 feature: 581019816: [*]N\N=C\[*]
19. Unknown FCFP_6 feature: -2100785893: [*]\N=C\[c](:[*]):[*]

20. Unknown FCFP_6 feature: -828984032: [*][N+](=[*])[c](:[cH]:[*]):[cH]:[*]
21. Unknown FCFP_6 feature: -1338588315: [*]:[c](:[*])[N+](=O)[O-]
22. Unknown FCFP_6 feature: 1872392852: [*][N+](=O)[*]
23. Unknown FCFP_6 feature: 260476081: [*][N+](=[*])[O-]

Feature Contribution

Top features for positive contribution

Fingerprint	Bit/Smiles	Feature Structure	Score
ECFP_6	642810091	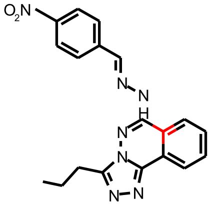 [*]:[c](:[*]):[*]	0.281
ECFP_6	-1897341097	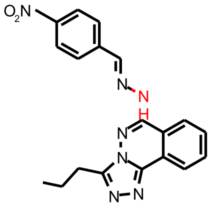 [*]N[*]	0.216
ECFP_6	-1074141656	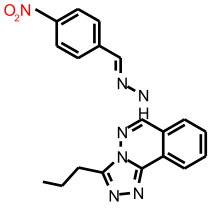 [*]=O	0.142

Top Features for negative contribution

Fingerprint	Bit/Smiles	Feature Structure	Score

ECFP_6	655739385	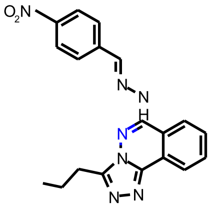 [*]:n:[*]	-0.239
ECFP_6	734603939	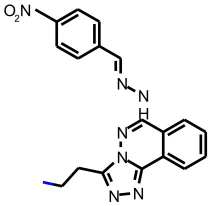 [*]C	-0.201
ECFP_6	-1795525632	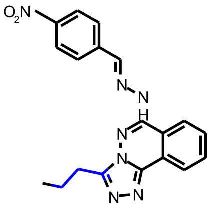 [*]CC[c](:[*]):[*]	-0.176

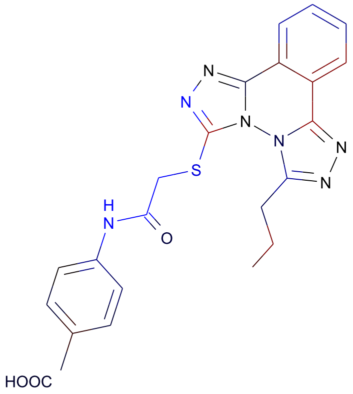

$C_{22}H_{19}N_7O_3S$
Molecular Weight: 461.49635
ALogP: 3.42
Rotatable Bonds: 7
Acceptors: 8
Donors: 2

Model Prediction

Prediction: 2.37
Unit: g/kg_body_weight
Mahalanobis Distance: 28.8
Mahalanobis Distance p-value: 1.8e-055

Mahalanobis Distance: The Mahalanobis distance (MD) is a generalization of the Euclidean distance that accounts for correlations among the X properties. It is calculated as the distance to the center of the training data. The larger the MD, the less trustworthy the prediction.
Mahalanobis Distance p-value: The p-value gives the fraction of training data with an MD greater than or equal to the one for the given sample, assuming normally distributed data. The smaller the p-value, the less trustworthy the prediciton. For highly non-normal X properties (e.g., fingerprints), the MD p-value is wildly inaccurate.

Structural Similar Compounds

Name	BENZENESULFONIC ACID; 2;2'-(4;4'-BIPHENYLYLENEDIVINYLENE)DI-; DISODIUM SALT (Na STRIPPED)	BENZOTHIAZOLE; 6-NITRO-2-(p-NITROBENZOYLAMINO)-	AZOSEMIDE
Structure	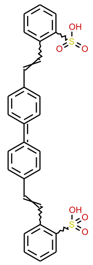	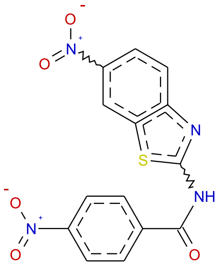	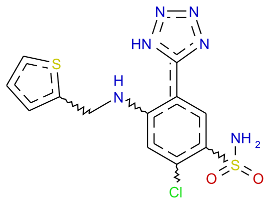
Actual Endpoint (-log C)	1.968	2.361	2.163
Predicted Endpoint (-log C)	1.72109	2.96257	2.21052
Distance	0.839	0.960	0.986
Reference	MVCRB3 2;193;73	JPETAB 90;260;47	IYKEDH 18;666;87

Model Applicability

Unknown features are fingerprint features in the query molecule, but not found or appearing too infrequently in the training set.

1. All properties and OPS components are within expected ranges.
2. Unknown ECFP_2 feature: 1986731747: [*]S[c]1:n:[*]:[*]:n:1:[*]
3. Unknown ECFP_2 feature: 78665610: [*][c]1:[*]:[*]:[c](:[*]):n:1:n(:[*]):[*]
4. Unknown FCFP_6 feature: 16: [*]:[cH]:[*]
5. Unknown FCFP_6 feature: -1410079687: [*]S[c]1:n:[*]:[*]:n:1:[*]
6. Unknown FCFP_6 feature: 4427049: [*][c](:[*]):n:n(:[*]):[*]
7. Unknown FCFP_6 feature: -1539162406: [*]C[c]1:n:[*]:[*]:n:1:[*]
8. Unknown FCFP_6 feature: -1564473960: [*]:n1:[*]:[*]:n:[c]:1:[c](:[*]):[*]
9. Unknown FCFP_6 feature: -332197802: [*][c]1:[*]:[*]:[c](:[*]):n:1:n:[*]
10. Unknown FCFP_6 feature: 1618154665: [*]:[cH]:[cH]:[cH]:[*]
11. Unknown FCFP_6 feature: -1549222613: [*]:[c](:[*])C(=O)O

Feature Contribution

Top features for positive contribution

Fingerprint	Bit/Smiles	Feature Structure	Score

ECFP_6	642810091	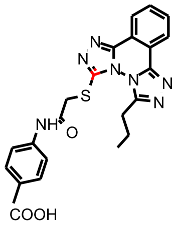<br>[*]:[c](:[*]):[*]	0.281
ECFP_6	-1897341097	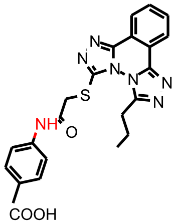<br>[*]N[*]	0.216
ECFP_6	1444581947	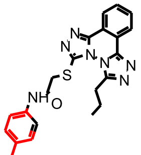<br>[*]C(=[*])[c]1:[cH]:[*]:[c]([*]):[cH]:[cH]:1	0.163
**Top Features for negative contribution**			
**Fingerprint**	**Bit/Smiles**	**Feature Structure**	**Score**
ECFP_6	655739385	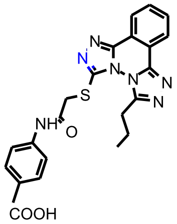<br>[*]:n:[*]	-0.239
FCFP_6	566058135	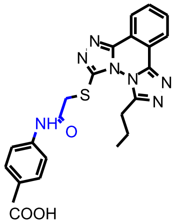<br>[*]CC(=O)N[*]	-0.216

ECFP_6	734603939	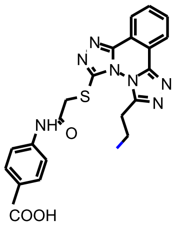 [*]C	-0.201

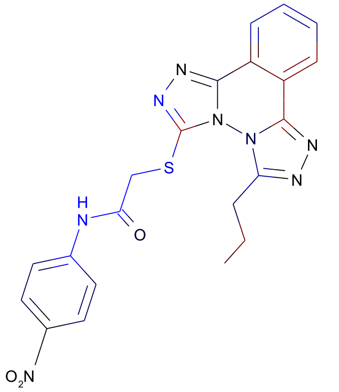

$C_{21}H_{18}N_8O_3S$

Molecular Weight: 462.48441

ALogP: 3.685

Rotatable Bonds: 7

Acceptors: 8

Donors: 1

Structural Similar Compounds

Name	BENZENESULFONIC ACID; 2;2'-(4;4'-BIPHENYLYLENEDIVINYLENE)DI-; DISODIUM SALT (Na STRIPPED)	BENZOTHIAZOLE; 6-NITRO-2-(p-NITROBENZOYLAMINO)-	ACEMETACIN
Structure	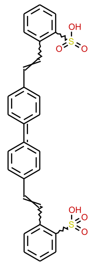	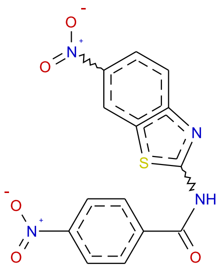	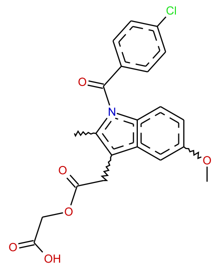
Actual Endpoint (-log C)	1.968	2.361	4.235
Predicted Endpoint (-log C)	1.72109	2.96257	3.39415
Distance	0.884	0.916	1.015
Reference	MVCRB3 2;193;73	JPETAB 90;260;47	ARZNAD 30;1398;80

Model Prediction

Prediction: 1.86

Unit: g/kg_body_weight

Mahalanobis Distance: 27.7

Mahalanobis Distance p-value: 6.3e-048

Mahalanobis Distance: The Mahalanobis distance (MD) is a generalization of the Euclidean distance that accounts for correlations among the X properties. It is calculated as the distance to the center of the training data. The larger the MD, the less trustworthy the prediction.
Mahalanobis Distance p-value: The p-value gives the fraction of training data with an MD greater than or equal to the one for the given sample, assuming normally distributed data. The smaller the p-value, the less trustworthy the prediciton. For highly non-normal X properties (e.g., fingerprints), the MD p-value is wildly inaccurate.

Model Applicability

Unknown features are fingerprint features in the query molecule, but not found or appearing too infrequently in the training set.

1. All properties and OPS components are within expected ranges.
2. Unknown ECFP_2 feature: 1043790491: [*][N+](=[*])[*]
3. Unknown ECFP_2 feature: 781519895: [*][O-]
4. Unknown ECFP_2 feature: 1986731747: [*]S[c]1:n:[*]:[*]:n:1:[*]
5. Unknown ECFP_2 feature: 78665610: [*][c]1:[*]:[*]:[c](:[*]):n:1:n(:[*]):[*]
6. Unknown ECFP_2 feature: -179073144: [*][N+](=[*])[c](:[cH]:[*]):[cH]:[*]
7. Unknown ECFP_2 feature: -215026467: [*]:[c](:[*])[N+](=O)[O-]
8. Unknown ECFP_2 feature: 2104376220: [*][N+](=O)[*]
9. Unknown ECFP_2 feature: -659271057: [*][N+](=[*])[O-]
10. Unknown FCFP_6 feature: 16: [*]:[cH]:[*]
11. Unknown FCFP_6 feature: 8: [*][N+](=[*])[*]
12. Unknown FCFP_6 feature: 5: [*][O-]
13. Unknown FCFP_6 feature: -1410079687: [*]S[c]1:n:[*]:[*]:n:1:[*]
14. Unknown FCFP_6 feature: 4427049: [*][c](:[*]):n:n(:[*]):[*]
15. Unknown FCFP_6 feature: -1539162406: [*]C[c]1:n:[*]:[*]:n:1:[*]
16. Unknown FCFP_6 feature: -1564473960: [*]:n1:[*]:[*]:n:[c]:1:[c](:[*]):[*]
17. Unknown FCFP_6 feature: -332197802: [*][c]1:[*]:[*]:[c](:[*]):n:1:n:[*]

18. Unknown FCFP_6 feature: 1618154665: [*]:[cH]:[cH]:[cH]:[*]
19. Unknown FCFP_6 feature: -828984032: [*][N+](=[*])[c](:[cH]:[*]):[cH]:[*]
20. Unknown FCFP_6 feature: -1338588315: [*]:[c](:[*])[N+](=O)[O-]
21. Unknown FCFP_6 feature: 1872392852: [*][N+](=O)[*]
22. Unknown FCFP_6 feature: 260476081: [*][N+](=[*])[O-]

Feature Contribution

Top features for positive contribution

Fingerprint	Bit/Smiles	Feature Structure	Score
ECFP_6	642810091	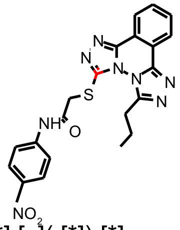 [*]:[c](:[*]):[*]	0.281
ECFP_6	-1897341097	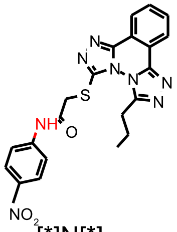 [*]N[*]	0.216
ECFP_6	-1074141656	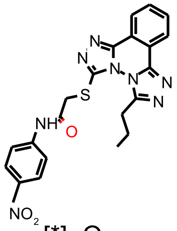 [*]=O	0.142

Top Features for negative contribution

Fingerprint	Bit/Smiles	Feature Structure	Score

ECFP_6	655739385	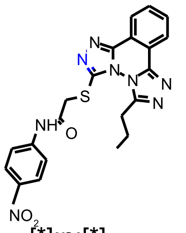 [*]:n:[*]	-0.239
FCFP_6	566058135	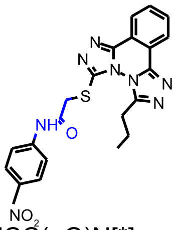 [*]CC(=O)N[*]	-0.216
ECFP_6	734603939	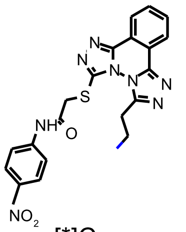 [*]C	-0.201

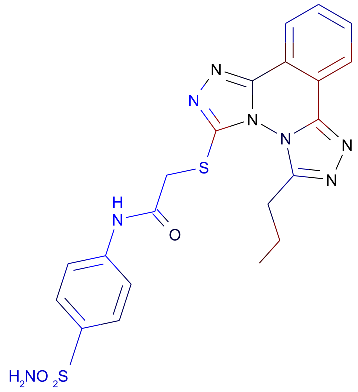

$C_{21}H_{20}N_8O_3S_2$
Molecular Weight: 496.56529
ALogP: 2.496
Rotatable Bonds: 7
Acceptors: 8
Donors: 2

Structural Similar Compounds

Name	AZOSEMIDE	BENZENESULFONIC ACID; 2;2'-(4;4'-BIPHENYLYLENEDIVINYLENE)DI-; DISODIUM SALT (Na STRIPPED)	2;7-NAPHTHALENE DISULFONIC ACID; 4-AMINO-5-HYDROXY-; p-TOLUENE SULFONATE ESTER
Structure	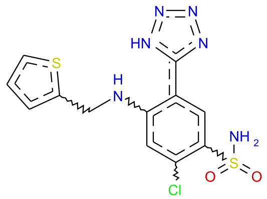	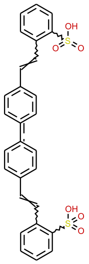	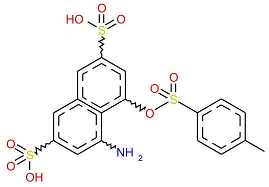
Actual Endpoint (-log C)	2.163	1.968	1.615
Predicted Endpoint (-log C)	2.21052	1.72109	1.79606
Distance	0.931	0.947	0.955
Reference	IYKEDH 18;666;87	MVCRB3 2;193;73	85JCAE -;1063;86

Model Prediction

Prediction: 12.3
Unit: g/kg_body_weight
Mahalanobis Distance: 29.9
Mahalanobis Distance p-value: 8.72e-064

Mahalanobis Distance: The Mahalanobis distance (MD) is a generalization of the Euclidean distance that accounts for correlations among the X properties. It is calculated as the distance to the center of the training data. The larger the MD, the less trustworthy the prediction.
Mahalanobis Distance p-value: The p-value gives the fraction of training data with an MD greater than or equal to the one for the given sample, assuming normally distributed data. The smaller the p-value, the less trustworthy the prediciton. For highly non-normal X properties (e.g., fingerprints), the MD p-value is wildly inaccurate.

Model Applicability

Unknown features are fingerprint features in the query molecule, but not found or appearing too infrequently in the training set.

1. All properties and OPS components are within expected ranges.
2. Unknown ECFP_2 feature: 1986731747: [*]S[c]1:n:[*]:[*]:n:1:[*]
3. Unknown ECFP_2 feature: 78665610: [*][c]1:[*]:[*]:[c](:[*]):n:1:n(:[*]):[*]
4. Unknown FCFP_6 feature: 16: [*]:[cH]:[*]
5. Unknown FCFP_6 feature: -1410079687: [*]S[c]1:n:[*]:[*]:n:1:[*]
6. Unknown FCFP_6 feature: 4427049: [*][c](:[*]):n:n(:[*]):[*]
7. Unknown FCFP_6 feature: -1539162406: [*]C[c]1:n:[*]:[*]:n:1:[*]
8. Unknown FCFP_6 feature: -1564473960: [*]:n1:[*]:[*]:n:[c]:1:[c](:[*]):[*]
9. Unknown FCFP_6 feature: -332197802: [*][c]1:[*]:[*]:[c](:[*]):n:1:n:[*]
10. Unknown FCFP_6 feature: 1618154665: [*]:[cH]:[cH]:[cH]:[*]

Feature Contribution

Top features for positive contribution

Fingerprint	Bit/Smiles	Feature Structure	Score

ECFP_6	642810091	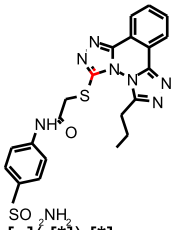 [*]:[c](:[*]):[*]	0.281
ECFP_6	-1897341097	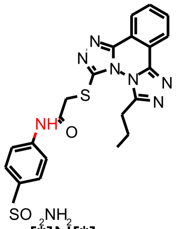 [*]N[*]	0.216
ECFP_6	-1074141656	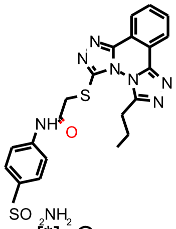 [*]=O	0.142

Top Features for negative contribution

Fingerprint	Bit/Smiles	Feature Structure	Score
ECFP_6	655739385	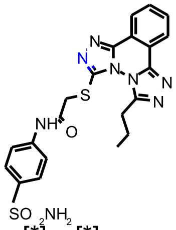 [*]:n:[*]	-0.239
FCFP_6	-1096219292	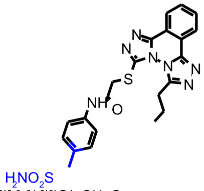 [*]:[c](:[*])S(=O)(=O)N	-0.225

FCFP_6	566058135	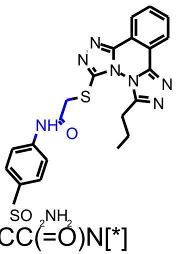 [*]CC(=O)N[*]	-0.216

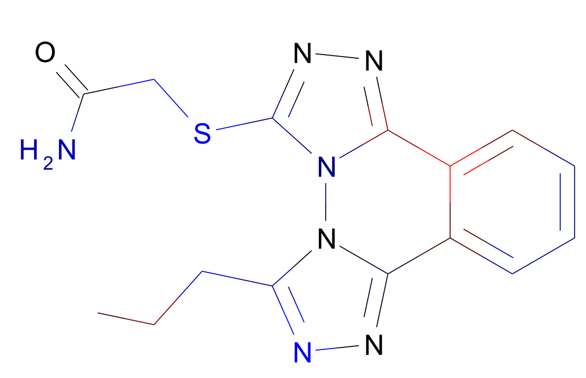

$C_{15}H_{15}N_{7}OS$

Molecular Weight: 341.39089

ALogP: 2.008

Rotatable Bonds: 5

Acceptors: 6

Donors: 1

Model Prediction

Prediction: 0.542

Unit: g/kg_body_weight

Mahalanobis Distance: 25.8

Mahalanobis Distance p-value: 5.79e-036

Mahalanobis Distance: The Mahalanobis distance (MD) is a generalization of the Euclidean distance that accounts for correlations among the X properties. It is calculated as the distance to the center of the training data. The larger the MD, the less trustworthy the prediction.
Mahalanobis Distance p-value: The p-value gives the fraction of training data with an MD greater than or equal to the one for the given sample, assuming normally distributed data. The smaller the p-value, the less trustworthy the prediciton. For highly non-normal X properties (e.g., fingerprints), the MD p-value is wildly inaccurate.

Structural Similar Compounds

Name	PRASOZIN .HCl (HCl STRIPPED)	AZATHIOPRINE	SULFAQUINOXALINE
Structure	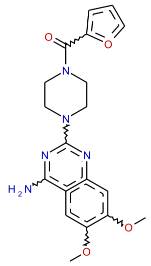	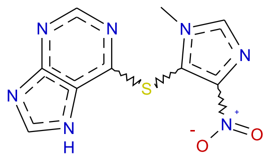	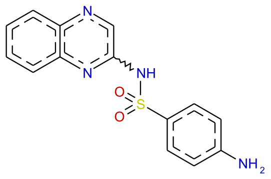
Actual Endpoint (-log C)	2.294	2.715	2.341
Predicted Endpoint (-log C)	3.00765	2.77505	2.42674
Distance	0.684	0.700	0.729
Reference	NIIRDN 6;688;82	NIIRDN 6;3;82	MahWM# 16NOV82

Model Applicability

Unknown features are fingerprint features in the query molecule, but not found or appearing too infrequently in the training set.

1. All properties and OPS components are within expected ranges.
2. Unknown ECFP_2 feature: 78665610: [*][c]1:[*]:[*]:[c](:[*]):n:1:n(:[*]):[*]
3. Unknown ECFP_2 feature: 1986731747: [*]S[c]1:n:[*]:[*]:n:1:[*]
4. Unknown FCFP_6 feature: 16: [*]:[cH]:[*]
5. Unknown FCFP_6 feature: 1618154665: [*]:[cH]:[cH]:[cH]:[*]
6. Unknown FCFP_6 feature: -1564473960: [*]:n1:[*]:[*]:n:[c]:1:[c](:[*]):[*]
7. Unknown FCFP_6 feature: -332197802: [*][c]1:[*]:[*]:[c](:[*]):n:1:n:[*]
8. Unknown FCFP_6 feature: -1539162406: [*]C[c]1:n:[*]:[*]:n:1:[*]
9. Unknown FCFP_6 feature: 4427049: [*][c](:[*]):n:n(:[*]):[*]
10. Unknown FCFP_6 feature: -1410079687: [*]S[c]1:n:[*]:[*]:n:1:[*]

Feature Contribution

Top features for positive contribution			
**Fingerprint**	**Bit/Smiles**	**Feature Structure**	**Score**

ECFP_6	642810091	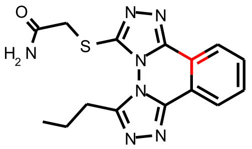 [*]:[c](:[*]):[*]	0.281
ECFP_6	-1074141656	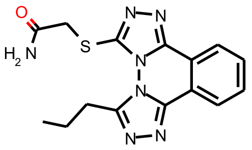 [*]=O	0.142
ECFP_6	-932108170	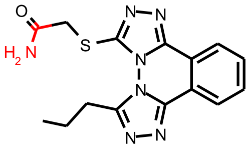 [*]C(=[*])N	0.126

Top Features for negative contribution

Fingerprint	Bit/Smiles	Feature Structure	Score
ECFP_6	655739385	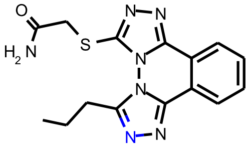 [*]:n:[*]	-0.239
FCFP_6	566058135	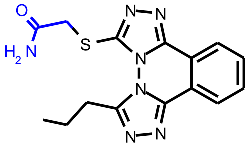 [*]CC(=O)N[*]	-0.216

ECFP_6	734603939	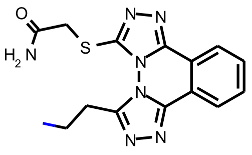<br>[*]C	-0.201

Doxorubicin

TOPKAT_Rat_Oral_LD50

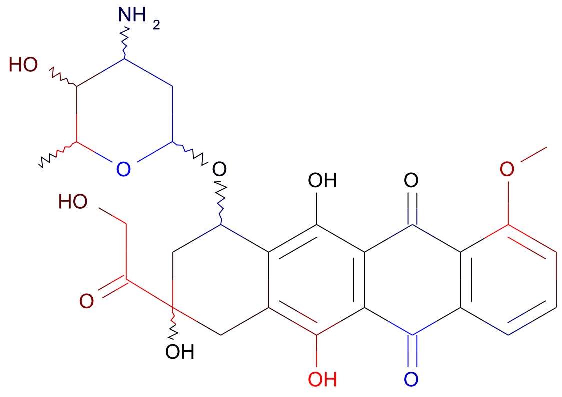

$C_{27}H_{29}NO_{11}$
Molecular Weight: 543.51925
ALogP: -4.4e-002
Rotatable Bonds: 5
Acceptors: 12
Donors: 6

Model Prediction

Prediction: 0.227
Unit: g/kg_body_weight
Mahalanobis Distance: 23.8
Mahalanobis Distance p-value: 7.02e-025

Mahalanobis Distance: The Mahalanobis distance (MD) is a generalization of the Euclidean distance that accounts for correlations among the X properties. It is calculated as the distance to the center of the training data. The larger the MD, the less trustworthy the prediction.
Mahalanobis Distance p-value: The p-value gives the fraction of training data with an MD greater than or equal to the one for the given sample, assuming normally distributed data. The smaller the p-value, the less trustworthy the prediciton. For highly non-normal X properties (e.g., fingerprints), the MD p-value is wildly inaccurate.

Structural Similar Compounds

Name	DAUNOMYCIN	VIRIDICATUM TOXIN	SECALONIC ACID
Structure	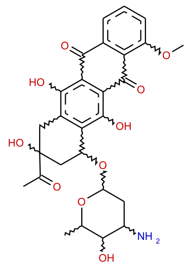	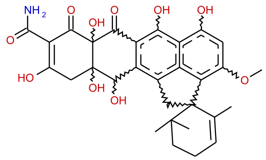	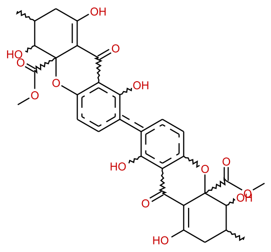
Actual Endpoint (-log C)	3.196	3.666	4.463
Predicted Endpoint (-log C)	3.6117	3.0269	3.37815
Distance	0.329	0.669	0.680
Reference	YKYUA6 25;573;74	TXAPA9 24;507;73	TXAPA9 48;A14;79

Model Applicability

Unknown features are fingerprint features in the query molecule, but not found or appearing too infrequently in the training set.

1. All properties and OPS components are within expected ranges.
2. Unknown FCFP_6 feature: 16: [*]:[cH]:[*]
3. Unknown FCFP_6 feature: 1618154665: [*]:[cH]:[cH]:[cH]:[*]
4. Unknown FCFP_6 feature: -1549669478: [*]:[c](:[*])C(=O)[c](:[*]):[*]
5. Unknown FCFP_6 feature: 74595001: [*]:[cH]:[c](O):[cH]:[*]
6. Unknown FCFP_6 feature: 1186333723: [*]CC(O[*])[c](:[*]):[*]
7. Unknown FCFP_6 feature: -549108873: [*]:[c](:[*])O

Feature Contribution

Top features for positive contribution			
Fingerprint	Bit/Smiles	Feature Structure	Score

ECFP_6	642810091	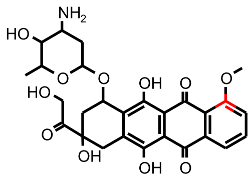 [*]:[c](:[*]):[*]	0.281
FCFP_6	136627117	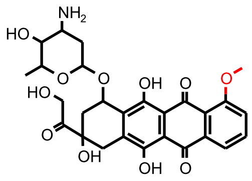 [*]OC	0.17
ECFP_6	-1074141656	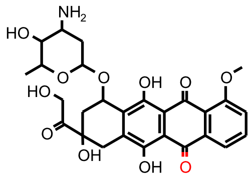 [*]=O	0.142

Top Features for negative contribution

Fingerprint	Bit/Smiles	Feature Structure	Score
ECFP_6	2106656448	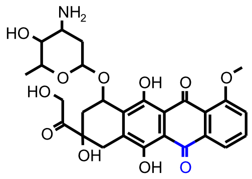 [*]C(=O)[*]	-0.352
ECFP_6	683445015	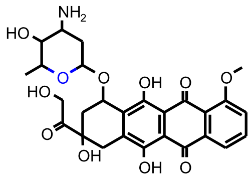 [*]O[*]	-0.266

ECFP_6	734603939	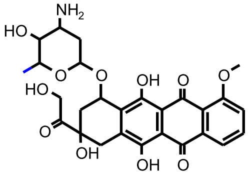<br>[*]C	-0.201

In silico ADMET analysis

ADMET descriptors (absorption, distribution, metabolism, excretion, and toxicity) of the compounds were determined using Discovery studio 4.0. At first, the CHARMM force field was applied then the compounds were prepared and minimized according to the preparation of small molecule protocol. Then ADMET descriptors protocol was applied to carry out these studies

Toxicity studies

The toxicity parameters of the synthesized compounds were calculated using Discovery studio 4.0. Doxorubicin was used as a reference drug. At first, the CHARMM force field was applied then the compounds were prepared and minimized according to the preparation of small molecule protocol. Then different parameters were calculated from toxicity prediction (extensible) protocol.

In silico ADMET analysis

ADMET descriptors (absorption, distribution, metabolism, excretion, and toxicity) of the compounds were determined using Discovery studio 4.0. At first, the CHARMM force field was applied then the compounds were prepared and minimized according to the preparation of small molecule protocol. Then ADMET descriptors protocol was applied to carry out these studies

Toxicity studies

The toxicity parameters of the synthesized compounds were calculated using Discovery studio 4.0. Doxorubicin was used as a reference drug. At first, the CHARMM force field was applied then the compounds were prepared and minimized according to the preparation of small molecule protocol. Then different parameters were calculated from toxicity prediction (extensible) protocol.

4.2.1. In vitro anti-proliferative activity against MCF-7, HepG-2 and HCT-116

The cells were cultured in RPMI-1640 medium supplemented with 10% fetal bovine serum (FBS), penicillin (100 unit /mL) and streptomycin sulphate (100µg/mL) at 37 oC in a 5% CO2 incubator. Then, cells were dropped in 96-well plates at a density of 3-8 × 103 cells/well and incubated for two days in a 5% CO2 incubator at 37 °C. Then, the cells were treated with the synthesized compounds and the cell cultures were continued incubated for 24 h. However, Different concentrations of the compound under test (0.0, 5.0, 12.5, 25.0 and 50 mg/ml) were added to the cell monolayer. Then, (3-[4,5-dimethylthiazol-2-yl]- 2,5-diphenyltetrazolium bromide) MTT solution (20 µl, 5mg/mL) was added to each well and incubated for additional 4 h. The formed purple crystals of MTT-formazan were dissolved in 100 µl DMSO each well; the absorbance of each well was measured at 570 nm using a plate reader (EXL 800, USA). All of the compounds were tested three times. The relative cell viability in percentage was calculated. The results for IC_{50} values of the active compounds are summarized in Table 1. The data represented the mean of three independent experiments in triplicate and were expressed as means ± SD. The IC_{50} value was defined as the concentration at which 50% of the cells could survive.

4.2.2. DNA intercalation assay (DNA/methyl green colorimetric assay)

Affinity of some compounds for DNA-binding was examined *in vitro* using DNA/methyl green assay using doxorubicin as a positive control. In this test, a mixture of methyl green (20 mg) and Calf thymus DNA (10 mg) (Sigma– Aldrich) were suspended in 0.05 M TrisHCl buffer (100 mL, pH 7.5) containing 7.5 mM MgSO4. This mixture was stirred continuously for 24 h at 37 °C. Then, ethanolic solution of the test compounds was pitted into the wells of a 96-well microtiter tray at a concentration of 10, 100 and 1000 µM. The excess solvent was removed from each well under vacuum, with subsequent addition of 200 µL of the DNA/methyl green solution. The test samples were incubated for 24 h in dark at ambient temperature. After that, absorbance of each sample was determined at 642.5–645 nm. In this test, the methyl green dye reversibly binds to DNA to form persistent colored complex of DNA/methyl green. This color still stables at neutral pH. When the DNA intercalators were added, the methyl green was displaced from DNA with addition of H2O molecule to the dye resulting in formation of the colorless carbinol leading to a decrease in spectrophotometric absorbance. ΔA value (the difference between DNA/methyl green complex and free cabinol) provides the simplest means for detecting the DNA-binding affinity and relative binding strength. IC50′s were determined by linear regression of data plotted on a semi-log scale and the data were compared with doxorubicin as standard DNA intercalator.

4.2.3. Measurement of topoisomerase II activity

Compounds (8a, 8b, 9a, 9c, 9d, 12a, and 12b) were further evaluated to assess their Topo II inhibitory activities. In this test, Topo II drug screening kit (TopoGEN, Inc., Columbus) was utilized to determine the Topo II activity. Doxorubicin was used as a reference drug in this test. A typical enzyme reaction contained a mixture of human Topo II (2 µL), substrate super coiled pHot1 DNA (0.25 µg), 50 µg/mL test compound (2 µL), and assay buffer (4 µL). The reaction started upon incubation of the mixture in 37 °C for 30 min. The reaction was terminated by addition of 10% sodium dodecylsulphate (2 µL) and proteinase K (50 µg/mL) at 37 °C for 15 min. followed by incubation for 15 min at 37 °C. Then, the DNA was run on 1% agarose gel in BioRad gel electrophoresis system for 1–2 h followed by staining with GelRedTM stain for 2 h and destained for 15 min with TAE buffer. The gel was imaged Via BioRad's Gel DocTMEZ system. Both supercoiled and linear strands DNA were incorporated in the gel as markers for DNA-Topo II intercalators. The results of IC50 values were calculated using the GraphPad Prism version 5.0. Each reaction was performed in duplicate, and at least three independent determinations of each IC_{50} were made.
